# Biocatalytic characterization of an alcohol dehydrogenase variant deduced from *Lactobacillus kefir* in asymmetric hydrogen transfer

**DOI:** 10.1038/s42004-023-01013-1

**Published:** 2023-10-12

**Authors:** Aleksandra Rudzka, Beata Zdun, Natalia Antos, Lia Martínez Montero, Tamara Reiter, Wolfgang Kroutil, Paweł Borowiecki

**Affiliations:** 1 Laboratory of Biocatalysis and Biotransformation, Department of Drugs Technology and Biotechnology, Faculty of Chemistry, Warsaw University of Technology, Koszykowa 75, 00–662, Warsaw, Poland; 2 Institute of Chemistry, University of Graz, NAWI Graz, BioTechMed Graz, Field of Excellence BioHealth, Heinrichstrasse 28, 8010 Graz, Austria

**Keywords:** Biocatalysis, Oxidoreductases, Screening, Biocatalysis, Asymmetric catalysis

## Abstract

Hydrogen transfer biocatalysts to prepare optically pure alcohols are in need, especially when it comes to sterically demanding ketones, whereof the bioreduced products are either essential precursors of pharmaceutically relevant compounds or constitute APIs themselves. In this study, we report on the biocatalytic potential of an anti-Prelog (*R*)-specific *Lactobacillus kefir* ADH variant (Lk-ADH-E145F-F147L-Y190C, named Lk-ADH Prince) employed as *E. coli*/ADH whole-cell biocatalyst and its characterization for stereoselective reduction of prochiral carbonyl substrates. Key enzymatic reaction parameters, including the reaction medium, evaluation of cofactor-dependency, organic co-solvent tolerance, and substrate loading, were determined employing the drug pentoxifylline as a model prochiral ketone. Furthermore, to tap the substrate scope of Lk-ADH Prince in hydrogen transfer reactions, a broad range of 34 carbonylic derivatives was screened. Our data demonstrate that *E. coli/*Lk-ADH Prince exhibits activity toward a variety of structurally different ketones, furnishing optically active alcohol products at the high conversion of 65–99.9% and in moderate-to-high isolated yields (38–91%) with excellent anti-Prelog (*R*)-stereoselectivity (up to >99% ee) at substrate concentrations up to 100 mM.

## Abstract (web summary)

Alcohol dehydrogenases (ADH) are known to be efficient and stereoselective biocatalysts for reducing prochiral carbonyl compounds to chiral alcohols; however, their catalytic performances remain limited for application. Here, the authors demonstrate the wide substrate scope of a *L. kefir* ADH variant with anti-Prelog specificity to generate (*R*)-alcohols.

## Introduction

**Fig. 1:**
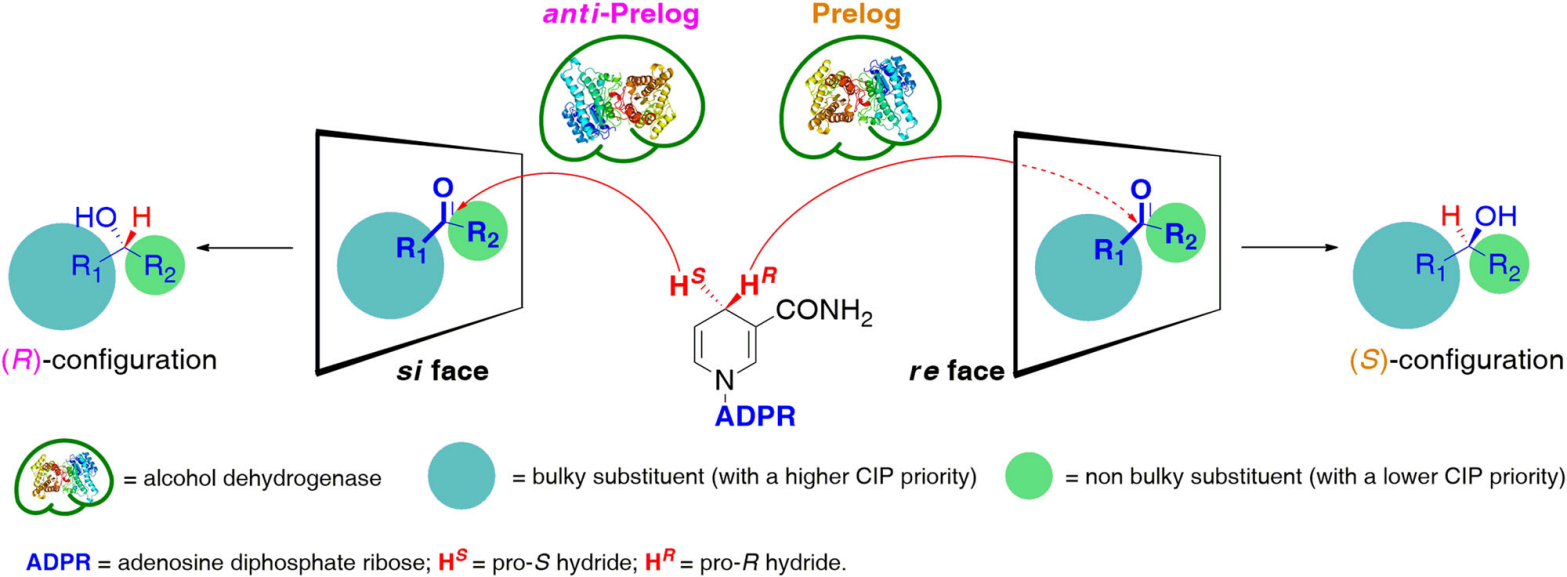
The Prelog’s rule for predicting the stereochemical outcome of ADH-catalyzed asymmetric reduction of prochiral carbonyl compounds. The dashed red line means below the plane.

Alcohol dehydrogenases (ADHs; EC 1.1.1.1–42), also known as ketoreductases (KREDs) or carbonyl reductases (CRs/CREDs), are considered as efficient and selective biocatalysts for reducing prochiral carbonyl compounds to chiral alcohols in a stereoselective manner^1–4^. The most common alcohol dehydrogenases applied in the biocatalytic synthesis of non-racemic alcohols (i.e., mono-alcohols, diols, halohydrins, amino alcohols, hydroxy acids/esters, etc.) include short-chain ADHs isolated from yeast (YADH)^5–7^, horse liver (HLADH)^8–12^, *Thermoanaerobacter pseudoethanolicus* (formerly *T. ethanolicus*; TeSADH or ADH-TH)^13–18^, *Thermoanaerobacter brockii* (TbSADH)^19–22^, *Rhodococcus erythropolis* (ReADH)^23,24^, *Rhodococcus ruber* (ADH-A)^25–28^, *Candida parapsilosis* (CpADH)^29,30^, *Pichia glucozyma* (PgluADH)^31–34^, *Bacillus subtilis* (BYueD)^35,36^, *Gluconobacter oxydans* (GoADH or GoCR)^37–40^, *Aromatoleum aromaticum* (*S*-HPED)^41–44^, *Flavobacterium psychrofilium* (FpADH)^45^, etc. All these enzymes catalyze the transfer of the hydride (H^−^) of the NAD(P)H cofactors from the *re*-face of prochiral carbonyl compounds, thus furnishing the so-called Prelog chiral alcohols, such as (*S*)-1-phenylethanol from acetophenone (Fig. 1). In turn, anti-Prelog (*R*)-stereospecific enzymes originated, i.e., from *Lactobacillus brevis* (Lb-ADH)^46–50^, *Lactobacillus kefir* (Lk-ADH)^51–53^, *Leifsonia* sp. (LSADH)^54–56^, *Pseudomonas* sp. (PED)^57,58^, *Aromatoleum aromaticum* (*R*-HPED)^59–61^, *Sporobolomyces salmonicolor* (SsADH)^62–64^, or *Flavobacterium* sp. (FsADH)^45^ deliver the hydride ion of NAD(P)H from the *si*-face of prochiral carbonyl substrates, yielding the corresponding (*R*)-alcohols in all the cases when the Cahn–Ingold–Prelog (CIP) priority rules are well conserved. Interestingly, among anti-Prelog ADHs, only LSADH, PED, and *R*-HPED are NADH-dependent.

Although NAD(P)^+^-dependent alcohol dehydrogenases have been widely in use since the mid-1990s, novel ADHs, including wild-type^65–70^ and engineered variants^71–77^ with improved catalytic performances, operational stability, and tailored substrate/cofactor specificity, are developed each year, showing that the potential of this area is far from being fully exploited. In that respect, recent developments in the molecular biology field, including directed evolution and site-directed mutagenesis, allowed the development of various valuable mutations of ADHs leading to efficient biocatalysts that can be applied toward, i.e., ‘bulky–bulky’ prochiral substrates, which are hardly accepted by the wild-type enzymes^78–82^. The recombinant proteins deserving special attention, which can stereoselectively reduce challenging ‘bulky–bulky’ ketones, include ADHs from *Ralstonia* sp. (RasADH)^83^, *Sphingobium yanoikuyae* (SyADH)^84,85^, *Paracoccus pantotrophus* (PpADH)^86^, *Kluyveromyces polyspora* (KpADH)^87,88^, etc. Moreover, the last decade showed that ADHs are important biocatalysts commonly applied in relatively novel synthetic concepts, including biocatalytic cascades^89–93^ and chemoenzymatic linear cascades^94–96^.

All the ADHs mentioned above and innovative biocatalytic strategies relying on them enable excellent synthetic tools for faster and cost-effective drug discovery. In this context, several blockbuster pharmaceuticals containing at least one chiral alcohol moiety (i.e., montelukast, atorvastatin, and crizotinib) have been devised on an industrial scale using ADHs as the key biocatalysts^97,98^. However, since many small-molecule chiral APIs are produced from (*R*)-configurated synthons, thus in general, anti-Prelog-type ADHs displaying (*R*)‐stereoselectivity (*R*-ADHs) are highly required in the pharmaceutical industry. Unfortunately, the wild-type ADHs exhibiting anti-Prelog stereoselectivity are rare in nature, and what is even more challenging is that their high substrate specificity restricts their applications in organic synthesis^99^. Therefore, in this study, we aimed at tapping the potential of a variant of the *L. kefir* ADH, namely Lk-ADH-E145F-F147L-Y190C, described previously for radical dehalogenation of lactones^100^, which we named Lk-ADH Prince, that turned here out to exhibit anti-Prelog specificity and excellent stereoselectivity toward a wide scope of carbonyl substrates, being drug-like molecules and/or their chiral precursors.

## Results and discussion

### Enzyme preparation

The reported Lk-ADH Prince enzyme was initially prepared by site saturation mutagenesis strategy using the 22-c-trick protocol from Acevedo et al.^101^. The following positions of *L. kefir* ADH were saturated: A94, E145, F147, D150, L153, Y190, L195, V196, L199, A202. The most beneficial mutations in terms of the improved substrate specificity and enzyme’s selectivity of action included the replacement of amino acids (AA) possessing sterically hindered aromatic residues (i.e., F147 and Y190), which are located in the vicinity of the entrance to the enzyme active site, with less bulky aliphatic AA (i.e., L147 and C190). In turn, the introduction of F instead of E into the 145-position of the protein structure sequence was made with the hope of establishing additional π − π, π−anion, and/or π−alkyl interactions with the bound substrates in close proximity toward the catalytic triad (S143-Y156-L160). Therefore, we envisioned that the optimized structure of the engineered Lk-ADH variant, especially by elimination of the narrowed parts of the entrance tunnel, would enhance transport pathways for bulky–bulky substrates into binding site, usually not acceptable by wild-type Lk-ADH.

### Reaction optimizations

**Fig. 2:**
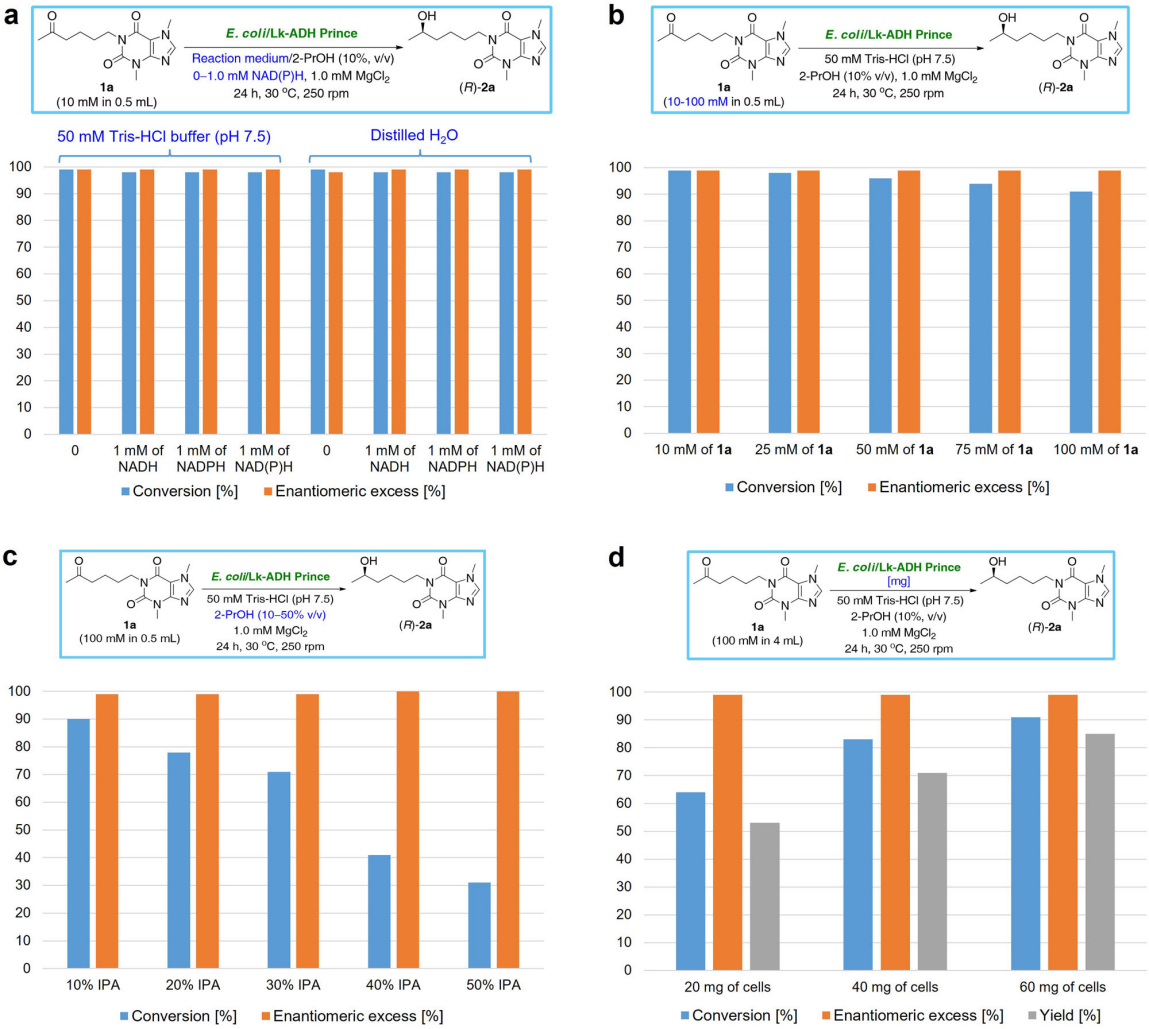
Optimization of the reaction conditions for the *E*. *coli*/Lk-ADH Prince-catalyzed asymmetric bioreduction of the model pentoxifylline (**1a**). **a** Effect of the type of an aqueous reaction medium and addition of the external NAD(P)H cofactors. **b** Effect of the substrate concentration. **c** Effect of the propan-2-ol (2-PrOH) concentration. **d** Reaction scale-up and impact of the amount of *E. coli*/Lk-ADH Prince cells. All the reactions were conducted for 24 h at 30 °C, 250 rpm (orbital shaker). The “IPA” abbreviation states for propan-2-ol.

**Fig. 3:**
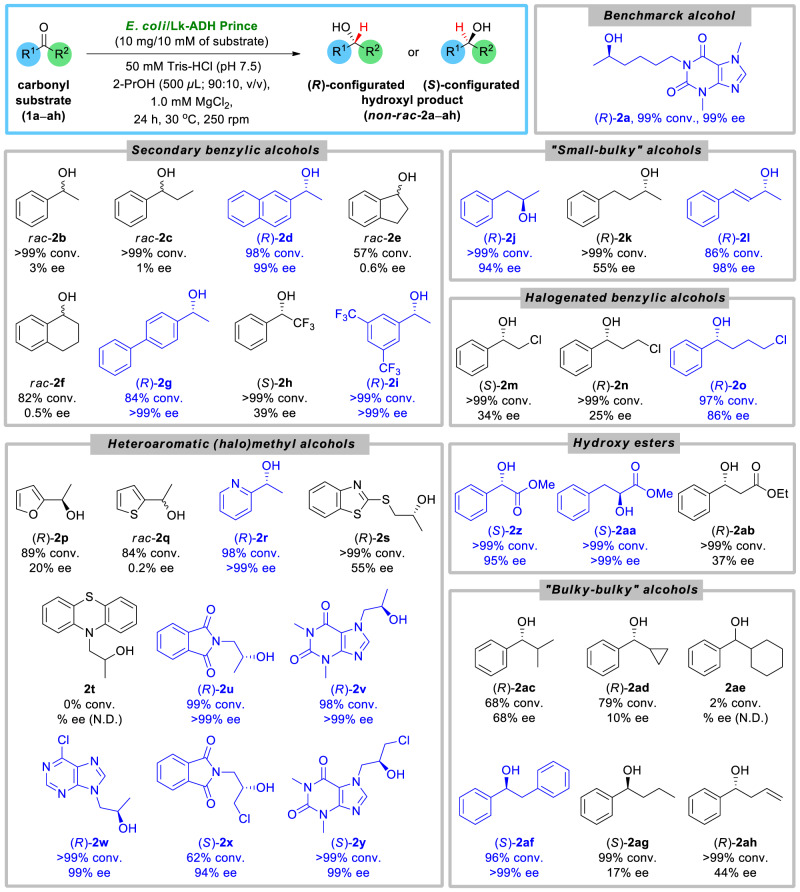
Substrate scope for *E. coli*/Lk-ADH Prince-catalyzed bioreduction of carbonyl compounds **1a**–**ah**. With blue color were highlighted products obtained with high-to-excellent enantiopurity (>80% ee).

The first hints at the applicability of freeze-dried whole *E. coli* cells containing the Lk-ADH Prince enzyme (*E. coli*/Lk-ADH Prince) for preparative-scale redox reactions was recently shown in the synthesis of enantiomerically pure (*R*)-lisofylline^102^ and two other xanthine-like derivatives^103^. In the current study, we employ pentoxifylline (**1a**) as a model substrate to evaluate the effects of various factors [i.e., reaction medium, type of external cofactor, substrate concentration, the amounts of propan-2-ol (2-PrOH) and *E. coli* cells] on the outcome of enzymatic reduction (Fig. 2), and further extend the optimal conditions toward the broader scope of the carbonyl substrates to show the full biocatalytic potential of Lk-ADH Prince (Fig. 3).

The initial analytical-scale *E. coli*/Lk-ADH Prince-catalyzed bioreductions of **1a** (Fig. 2a or Supplementary Table 1 in Supplementary Information) were carried out using 10 mM of the prochiral substrate and 10 mg of freeze-dried *E. coli* cells (20 mg/mL) containing the overexpressed enzyme suspended in 450 μL of 50 mM Tris–HCl buffer (pH 7.5) or distilled water in the presence of 1.0 mM of the nicotinamide cofactors (NADH and/or its phosphate counterpart NADPH) or without supplemental NAD(P)H, respectively. The reactions were also supplied with 1.0 mM of MgCl_2_ since, in the case of ADHs isolated from *L. kefir*, it was found that the addition of Mg^2+^ ions is beneficial to prevent rapid deactivation of the enzyme^104^. Moreover, to improve the solubility of the ketone **1a** in the aqueous media and allow *in celulo* regeneration of the NAD(P)H cofactors, we supplied each reaction mixture with 10% v/v of 2-PrOH (50 μL) as the cheap sacrificial co-substrate. All the biotransformations were carried out for 24 h at 30 °C using a gentle stirring (an orbital shaker, 250 rpm) and without access to air.

Preliminary experiments indicate that bioreduction rates of the model ketone **1a** and the optical purity of the alcohol (*R*)-**2a** produced were similar in almost all cases, suggesting that the catalytic activity and stereoselectivity of *E. coli*/Lk-ADH Prince toward **1a** are less dependent on a given set of conditions. Interestingly, the most promising results in terms of the substrate conversion (>99% conv.) and stereochemical outcome (99% ee) were obtained when the biocatalytic reduction of **1a** was performed in the buffered solution at pH 7.5 and without adding additional NAD(P)H cofactors. This can be explained as lyophilized whole cells were applied containing already the cofactor^105,106^. Notably, the amount of intracellular NAD(P)H cofactors in lyophilized cells may be limited, i.e., due to degradation during lyophilization or storage. However, our experiments showed that the employed recombinant *E. coli* cells allowed continuous regeneration of the cofactors.

After identifying suitable conditions for *E. coli*/Lk-ADH Prince-catalyzed bioreduction of **1a**, we aimed to increase the substrate concentration from 10 mM to 25 mM, 50 mM, 75 mM, and 100 mM (Fig. 2b or Supplementary Table 2 in Supplementary Information), respectively. Noteworthy, complete conversion was achieved only with the lowest concentration (10 mM) at the conditions employed. Nevertheless, with 100 mM final conc. of prochiral ketone, the product (*R*)-**2a** was still obtained with 91% conv. Since the drop in the substrate conversion was negligible in the range of all tested concentrations and should not affect overall volumetric productivity, we decided to use 100 mM final conc. of **1a** in the subsequent attempts toward the asymmetric biosynthesis of the target product (*R*)-**2a**.

Since it is preferable to use a single ADH with auxiliary alcohol (i.e., 2-PrOH, EtOH) as the hydrogen donor for cofactor recycling, in the next step, we aimed to scrutinize the effect of increased 2-PrOH concentration under optimal reaction conditions (Fig. 2c or Supplementary Table 3 in Supplementary Information). We conducted these experiments motivated by the fact that in the best-case scenario of the bioreduction mode, the elevated 2-PrOH-concentrations not only shift a thermodynamic equilibrium toward the product formation but also increase the solubility of lipophilic substrates in the monophasic aqueous/organic medium, thus ensuring enhanced reaction rates^107^. In this context, we analyzed substrate conversion catalyzed by *E. coli*/Lk-ADH Prince by testing different percentages of the co-solvent additive from 10% v/v to 50% v/v. However, increasing 2-PrOH concentrations had a negative effect on the substrate conversion and resulted in a 3-fold drop of its value in the case of 50% v/v of co-solvent. These results might be due to the instability of the Lk-ADH Prince toward 2-PrOH and the in situ formed acetone, which is known as an even more harmful compound to the enzymes’ catalytic activity than short-chain alcohols^4^. The other explanation for the drop in the substrate conversion is the change in the ratio of ketone/2-PrOH, which affects the reaction’s equilibrium for a given set of bioreduction conditions. Of note, the whole-cell biocatalyst turned out to be relatively stable towards elevated co-substrate concentrations, thus allowing the use of 2-PrOH for cofactor regeneration at 30% v/v. Nevertheless, to preserve the catalytic system’s highest possible productivity, we continued our studies using a 10% v/v 2-PrOH supply. Interestingly, although higher concentrations of 2-PrOH (40–50% v/v) gave worse results in terms of substrate conversion (31–41%), we found them beneficial for enantiomeric excess of (*R*)-**2a** as it was obtained in optically pure form (>99% ee).

In the next step, to evaluate the general applicability of the elaborated method on a semi-preparative-scale, stereoselective hydrogen-transfer bioreduction of **1a** catalyzed by *E. coli*/Lk-ADH Prince was scaled up to 0.4 mmol [100 mM final conc. in 4 mL (total reaction volume)] of the prochiral ketone (Fig. 2d or Supplementary Table 4 in Supplementary Information). Noteworthy, substrate **1a** was subjected to the optimized biocatalytic conditions with the preservation of the linear enlargement of the buffer volume and the amounts of co-solvent and the MgCl_2_ additive. The studied effect of the altered quantities of *E. coli* cells on the reaction outcome revealed that to achieve a similar % conv. value as in the analytical scale, at least 60 mg of the lyophilized cells had to be applied. Lower cell dosage might be insufficient to produce (*R*)-**2a** with a concentration beyond 100 mM. In turn, higher cell dosage might increase substrate conversion and reaction velocity, but we assumed that it would also increase the cost of the process. Hence, 15 mg/mL lyophilized cells were selected for further studies.

### Substrate scope

In order to gain a deeper understanding of the substrate specificity and stereoselectivity of *E. coli*/Lk-ADH Prince, we next aimed to investigate a plethora of other than benchmark pentoxifylline (**1a**) carbonyl compounds **1b**–**ah** (Fig. 3).

This set of prochiral compounds included benzylic ketones **1b**–**i**, “small-bulky” ketones **1j**–**l**, halogenated benzylic ketones **1m**–**o**, heteroaromatic (halo)methyl ketones **1p**–**y**, α- and β-keto esters **1z**–**ab**, and “bulky–bulky” ketones **1ac**–**ah**. Especially the last group of the mentioned substrates **1ac**–**ah** seems very interesting from the viewpoint of asymmetric bioreduction as the sterically demanding carbonyl compounds are “hard-to-be-discriminated” by enzymes due to the low difference in the size of the substituents located around the prochiral center. Noteworthy, among the tested prochiral substrates **1a**–**ah** were compounds, which after bioreduction of their carbonyl group, provide either pharmaceuticals directly, as in the case of lisofylline [(*R*)-**2a**] and proxyphylline [(*R*)-**2v**], or lead to crucial chiral synthons, which can be further used in the synthesis of natural products and/or APIs that exhibit a myriad of pharmacological properties (for details, see Supplementary Table 10 appended in Supplementary Information).

Incipiently, enzymatic reactions were carried out on an analytical scale using 10 mM of the respective prochiral substrate **1b**–**ah** and 10 mg of *E. coli* cells suspended in aqueous Tris–HCl buffer (50 mM, pH 7.5) in the presence of 1.0 mM of MgCl_2_ and 2-PrOH (10% v/v) as a hydrogen donor and without additional cofactors. All the biotransformations were performed at 30 °C and arbitrarily terminated after 24 h. Detailed examination of the obtained results revealed that *E. coli*/Lk-ADH Prince showed broad substrate scope and stereoselectivity to produce chiral alcohols with excellent enantiomeric excesses. An unprecedented substrate scope and near-to-absolute stereoselectivity (98–99.9% ee) toward 12 out of a total 34 tested prochiral carbonyl compounds proved that this variant of (*R*)-specific ADH from *L. kefir* is a very interesting enzyme for preparative applications. In this context, *E. coli*/Lk-ADH Prince showcases excellent stereoselectivity toward carbonyl substrates, which possess a small methyl group located nearby the prochiral center and large hetero(aromatic) substituent on the other side of the carbon atom of carbonyl group, such as in the case of pentoxifylline (**1a**), 1-(naphthalen-2-yl)ethenone (**1d**), 1-([1,1’-biphenyl]-4-yl)ethenone (**1g**), 1-(3,5-bis(trifluoromethyl)phenyl)ethenone (**1i**), 1-(pyridin-2-yl)ethenone (**1r**), and 2-(2-oxopropyl)isoindoline-1,3-dione (**1u**). Interestingly, it was observed that *E. coli*/Lk-ADH Prince also exhibits superb catalytic activity toward almost all the aforementioned substrates leading to desired alcohols (*R*)-**2a**, (*R*)-**2d**, (*R*)-**2i**, (*R*)-**2r**, and (*R*)-**2u** with excellent 98–99.9% conv., except **1g**, which was functionalized toward (*R*)-**2g** with 84% conv. To our surprise, single derivatives from the series of α-chloro ketones [i.e., 7-(3-chloro-2-oxopropyl)-1,3-dimethyl-1*H*-purine-2,6(3*H*,7*H*)-dione (**1y**)], α-keto esters [i.e., methyl 2-oxo-3-phenylpropanoate (**1aa**)], and “bulky–bulky” ketones [i.e., 1,2-diphenylethanone (**1af**)] were found to be reduced to the respective optically pure alcohols (*S*)-**2y**, (*S*)-**2aa**, and (*S*)-**2af** with 96–99.9% conv. In turn, relatively high stereoselectivity (88–98% ee) was observed for (*E*)-4-phenylbut-3-en-2-one (**1l**), 1-phenylpropan-2-one (**1j**), 2-(3-chloro-2-oxopropyl)isoindoline-1,3-dione (**1x**), methyl 2-oxo-2-phenylacetate (**1z**), and 4-chloro-1-phenylbutan-1-one (**1o**). Interestingly, **1o** was the only substrate from halogenated benzylic ketones **1m**–**o** that was reduced with >80% ee. In contrast, the other two derivatives from this series, namely 2-chloro-1-phenylethanone (**1m**) and 3-chloro-1-phenylpropan-1-one (**1n**), were reduced with moderate stereoselectivity, thus allowing to obtain corresponding alcohols (*S*)-**2m** and (*R*)-**2n** in 25–34% ee, respectively. In turn, low stereoselectivity of *E. coli*/Lk-ADH Prince toward substrates that exhibit steric limitations disabling the acceptance into the enzyme active site and the subsequent transformation were observed in the case of 2-methyl-1-phenylpropan-1-one (**1ac**), cyclopropyl(phenyl)methanone (**1ad**), 1-phenylbutan-1-one (**1ag**), and 1-phenylbut-3-en-1-one (**1ah**). Another crucial finding was observed during the investigations with keto esters **1z**–**ab**, which all showed very high reactivity in the bioreductions catalyzed by *E. coli*/Lk-ADH Prince, thus affording the corresponding products (*S*)-**2z**–**aa** and (*R*)-**2ab** with >99% conv. However, in the case of the substrate **1ab** with an oxo group at the β-position to ester moiety, the titled biocatalysts turned out to be moderately stereoselective, allowing to obtain (*R*)-**2ab** in only 37% ee. The most interesting result in terms of the stereoselectivity outcome was noticed in the case of two bulky–bulky ketones **1af** and **1ag**, which were reduced in the presence of *E. coli*/Lk-ADH Prince into the corresponding alcohols (*S*)-**2af** and (*S*)-**2ag** with reversed absolute configuration on the stereogenic centers. Interestingly, substrates possessing similar aliphatic chain to **1ag**, but with terminal alkene moiety as in the case of **1ah**, or the attached chlorine atom as for **1n**–**o**, furnished (*R*)-alcohols.

In contrast to the wild-type ADH from *L. kefir*, the engineered biocatalyst exhibits in principal no stereoselectivity toward acetophenone (**1b**), propiophenone (**2c**), 1-indanone (2,3-dihydro-1*H*-inden-1-one, **2e**), α-tetralone (3,4-dihydronaphthalen-1(2*H*)-one, **2f**), and 2-acetylthiophene (1-(thiophen-2-yl)ethenone, **2q**), yielding the respective racemic mixtures as the bioreduction products. Additionally, the data concerning the stereopreference for the substrates clarified that *E. coli*/Lk-ADH Prince produced (*R*)-alcohols in all the cases except (*S*)-**2h**, (*S*)-**2m**, (*S*)-**2x**, (*S*)-**2y**, (*S*)-**2z**, and (*S*)-**2aa**, which is due to a switch in CIP priority. Moreover, although the reaction system was not optimized for each substrate, *E. coli*/Lk-ADH Prince has been found to catalyze the bioreduction of 32 carbonyl substrates, indicating a pretty broad substrate spectrum. It turned out that only two ketones **1t** and **1ae** failed to be reduced by the titled biocatalyst, as no reaction in these cases was detected. For a schematic overview of the optical purity spectrum for the tested *E. coli*/Lk-ADH Prince toward carbonyl substrates, please see Supplementary Fig. 1 deposited in Supplementary Information. The absolute configuration of the chiral products was determined based on the elution order of the HPLC peaks separated by chiral columns compared to literature data and/or commercial analytical standards (for details, please see Supplementary Table 13 in Supplementary Information).

### Reaction scale-up and further optimizations

**Fig. 4:**
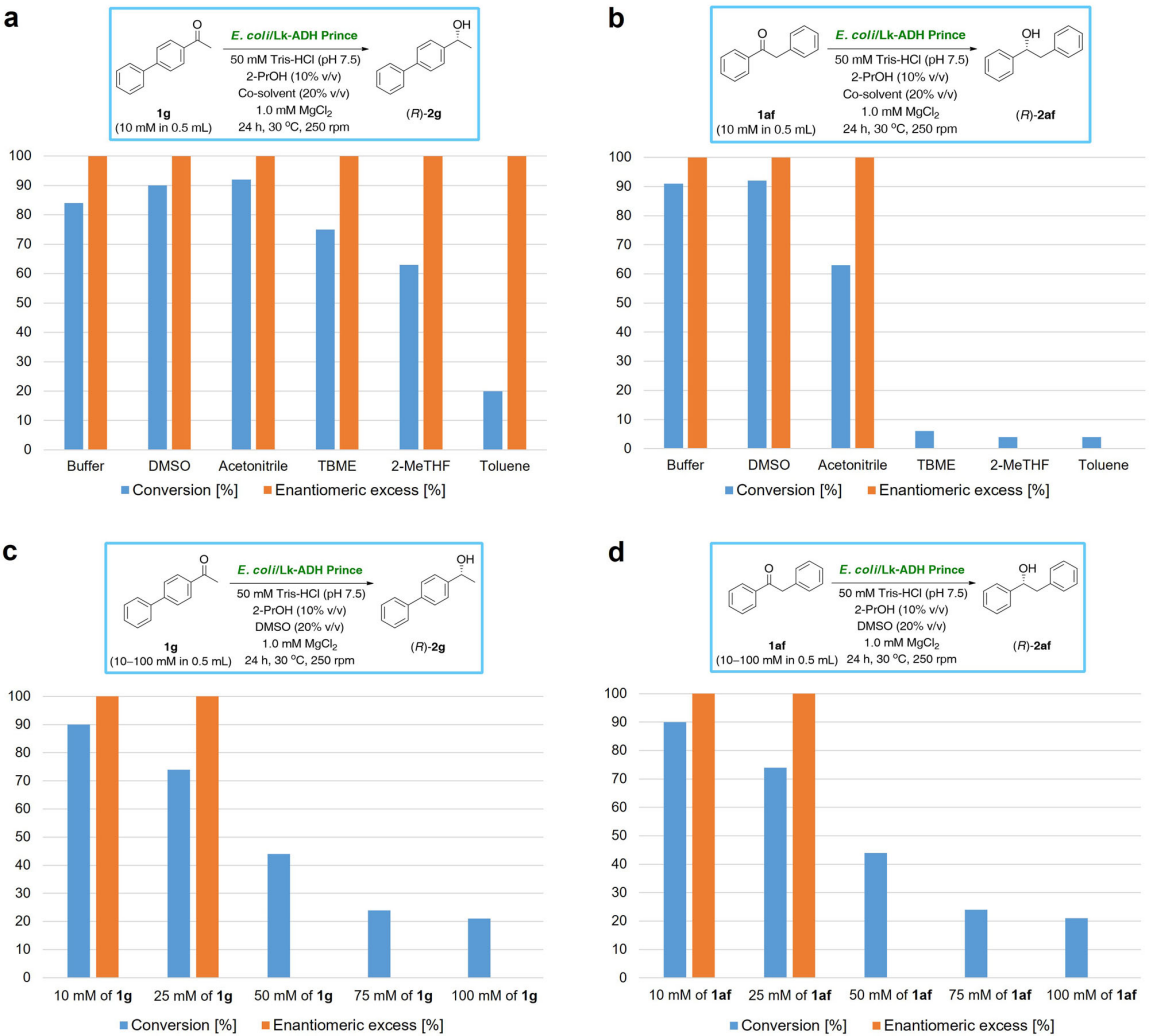
Optimization of the reaction conditions for the *E*. *coli*/Lk-ADH Prince-catalyzed asymmetric bioreduction of 1-(biphenyl-4-yl)ethanone (**1g**) and 1,2-diphenylethanone (**1af**). **a** and **b** Effect of the organic co-solvent (20% v/v) on the conversion of the *E*. *coli*/Lk-ADH Prince-catalyzed reduction of **1g** (**a**) or **1af** (**b**) in the presence of 2-propanol (10% v/v). **c** and **d** Effect of the concentration of substrate **1g** (**c**) or **1af** (**d**) in the presence of DMSO (20% v/v) and 2-propanol (10% v/v) as co-solvents. All the reactions were conducted with **1g** or **1af** (10 mM final conc. in 0.5 mL total volume), lyophilized *E*. *coli*/Lk-ADH Prince (10 mg), and 1.0 mM MgCl_2_ for 24 h at 30 °C, 250 rpm (orbital shaker).

In order to demonstrate the preparative application of *E. coli*/Lk-ADH Prince, the enzyme preparation was further used for the stereoselective bioreduction of carbonyl substrates, for giving the alcohols with high-to-excellent enantiopurity (>80% ee) in the previous step (see Fig. 3). With optimal reaction conditions in hand, we have scaled up the reactions to 0.4 mmol of the respective substrate (100 mM final conc. in 4 mL total volume). However, some of the examined derivatives **1d,** **1g,** **1l,** **1u,** **1v,** **1x**, and **1af** failed to form homogenous solutions within the reaction medium and/or to react at 100 mM conc. Therefore, prior to the preparative-scale investigations, we performed bioreductions of two randomly selected, poorly water-soluble ketone substrates, namely 1-(biphenyl-4-yl)ethanone (**1g**) and 1,2-diphenylethanone (**1af**), in mono- or biphasic organic−aqueous systems composed of 50 mM Tris–HCl buffer (pH 7.5), 2-PrOH (10% v/v), and different organic co-solvents (20% v/v) displaying a broad range of hydrophobicity (Fig. 4a, b or Supplementary Table 5 and Supplementary Table 6 in Supplementary Information). Noteworthy, studying the effects of reaction media on the catalytic performance and robustness of ADHs is critical since the activity, stability, and selectivity of enzymes can differ significantly according to the organic solvents’ nature^108–114^. The solvent screening revealed that in both cases, monophasic aqueous–organic systems using polar aprotic solvents, such as DMSO and CH_3_CN, are more suitable for *E. coli*/Lk-ADH Prince-catalyzed bioreductions leading to higher conversions of **1g** (90–92% conv.) and **1af** (63–92% conv.) than in biphasic solvent systems with water-immiscible solvents, such as methyl *tert*-butyl ether (MTBE), 2-methyltetrahydorfuran (2-MeTHF), and toluene (PhCH_3_), which gave 20–75% conv. in the case of **1g** and 4–6% conv. in the case of **1af**. Gratifyingly, the co-solvents did not affect stereoselectivity; thus, for both studied substrates, the reactions led to the optically pure products (*R*)-**2g** and (*S*)-**2af** in >99% ee. Finally, the outcome of the reaction conducted in the presence of DMSO was comparable to that achieved in buffer solution supplemented only with 2-PrOH; however, since the conversions improved, we used this co-solvent in the following attempts.

Subsequently, we evaluated the effect of the substrate concentration on *E. coli*/Lk-ADH Prince-catalyzed bioreduction of **1g** and **1af** (Fig. 4c, d or Supplementary Table 7 and Supplementary Table 8 in Supplementary Information). The conversions were measured under established conditions at varied concentrations of **1** **g** and **1af** (10 mM, 25 mM, 50 mM, 75 mM, and 100 mM) after 24 h.

Our results showed that the conversions in the reduction of both ketones were acceptably high at 10–25 mM final conc., and reached 74–90%. In contrast, a significant decrease in conversion was detected when the substrate concentrations exceeded 50 mM, which led to diminished values (<44% conv.). The negative effect of the increased concentrations of ketones on enzyme catalytic activity indicates that substrate inhibition occurs in these cases.

Considering all the abovementioned findings, our next step was to examine the outcome of preparative-scale *E. coli*/Lk-ADH Prince-catalyzed asymmetric bioreduction of the selected carbonyl compounds (Table 1).

**Table 1:** Preparative-scale *E. coli*/Lk-ADH Prince-catalyzed asymmetric bioreduction of ketones^a^.


Entry	Substrate		Substrate conc. [mM]	DMSO [%, v/v]	Conv.^b^ [%]	Yield^c^ [%]	ee^d^ [%] / (conf.)^e^
	Structure	Annotation					
1		**1a**	100	0	91	86	99 / (*R*)
2		**1d**	100	5	65	51	99 / (*R*)
3		**1g**	25	20	66	66	>99 / (*R*)
4		**1i**	100	0	91	73	>99 / (*R*)
5		**1j**	100	0	96	62	93 / (*R*)
6		**1l**	100	5	52	34	98 / (*R*)
7			25	5	69	67	98 / (*R*)
8		**1o**	100	0	82	41	88 / (*R*)
9		**1r**	100	0	99	79	96 / (*R*)
10		**1u**	100	20	99	76	>99 / (*R*)
11		**1v**	100	5	51	30	>99 / (*R*)
12			25	5	>99	67	>99 / (*R*)
13		**1w**	100	0	>99	91	>99 / (*R*)
14		**1x**	25	20	>99	38	95 / (*S*)
15		**1y**	100	0	59	26	99 / (*S*)
16			25	0	95	59	99 / (*S*)
17		**1z**	100	0	>99	68	94 / (*S*)
18		**1aa**	100	0	>99	67	>99 / (*S*)
19		**1af**	25	20	66	40	>99 / (*S*)

^a^Reaction conditions: carbonyl substrate **1a**–**af** (0.1–0.4 mmol, 25–100 mM final conc. in 4 mL final volume), *E. coli*/Lk-ADH Prince (60 mg), 1.0 mM MgCl_2_, 50 mM Tris–HCl (pH 7.5), 2-PrOH (10, v/v), DMSO (0–20, v/v), 24 h, 30 °C, 250 rpm.

^b^Conversion values (%) determined by GC analyses using calibration curves.

^c^Isolated yield after column chromatography.

^d^Enantiomeric excess determined for non**-**racemic hydroxyl products using HPLC analysis on chiral stationary phases.

^e^Absolute configurations of the optically active products were established by comparing HPLC peaks elution order with commercial enantiomeric standards or literature data (for details, see Supplementary Table 13 in Supplementary Information). The major enantiomer is shown in parentheses.

Noteworthy, those substrates, which probably inhibited or denatured Lk-ADH Prince, were used at lower concentrations (25 mM), whereas those with limited solubility in an aqueous reaction medium were supplemented with DMSO (5–20% v/v), respectively. Concerning the optical purity of the isolated products, the results were in line with our previous observations obtained during analytical-scale studies, except for **1r**, in which case bioreduction led to a slight decrease in enantiomeric excess of (*R*)-**2r** (from >99% to 96%). In terms of the isolated yield, excellent results (>90%) were achieved in the case of **1w**; very good (80–90%) in the case of **1a**; good (70–80%) in the case of **1i,** **1r**, and **1** **u**; moderate (40–70%) in the case of **1d,** **1g,** **1j,** **1l,** **1o,** **1v,** **1y,** **1z,** **1aa**, and **1af**; low (<40%) in the case of **1x**.

**Fig. 5:**
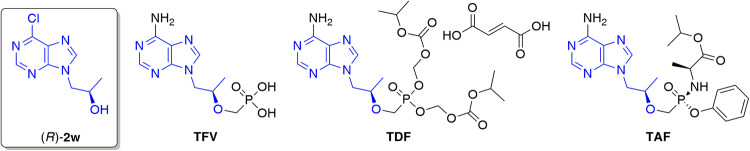
Chemical structures of tenofovir (TFV), tenofovir 5′-disoproxil fumarate (TDF), tenofovir 5′-alafenamide (TAF), and their key precursor – (2*R*)-1-(6-chloro-9*H*-purin-9-yl)propan-2-ol [(*R*)-**2w**]. With blue color were highlighted common core structure.

After examining all the data sets, it was clear that the most satisfied synthesis efficiency was reached in the case of **1w**. This ketone was stereoselectively reduced in the presence of *E*. *coli*/Lk-ADH Prince at the highest possible substrate concentration (100 mM; 1 mmol/mL) without using DMSO as co-solvent, thus furnishing optically pure (*R*)-**2w** (>99% ee) with >99% conv. in 91% isolated yield. Since (*R*)-**2w** is the key precursor in the asymmetric synthesis of blockbuster antiretroviral tenofovir (**TFV**), and its lipophilic prodrugs, including tenofovir 5′-disoproxil fumarate (**TDF**, Viread^®^) and tenofovir 5′-alafenamide (**TAF**, Vemlidy^®^) (Fig. 5)^115^, *E*. *coli*/Lk-ADH Prince can be considered as a valuable biocatalyst from the viewpoint of medicinal chemistry.

### Computational studies

**Fig. 6:**
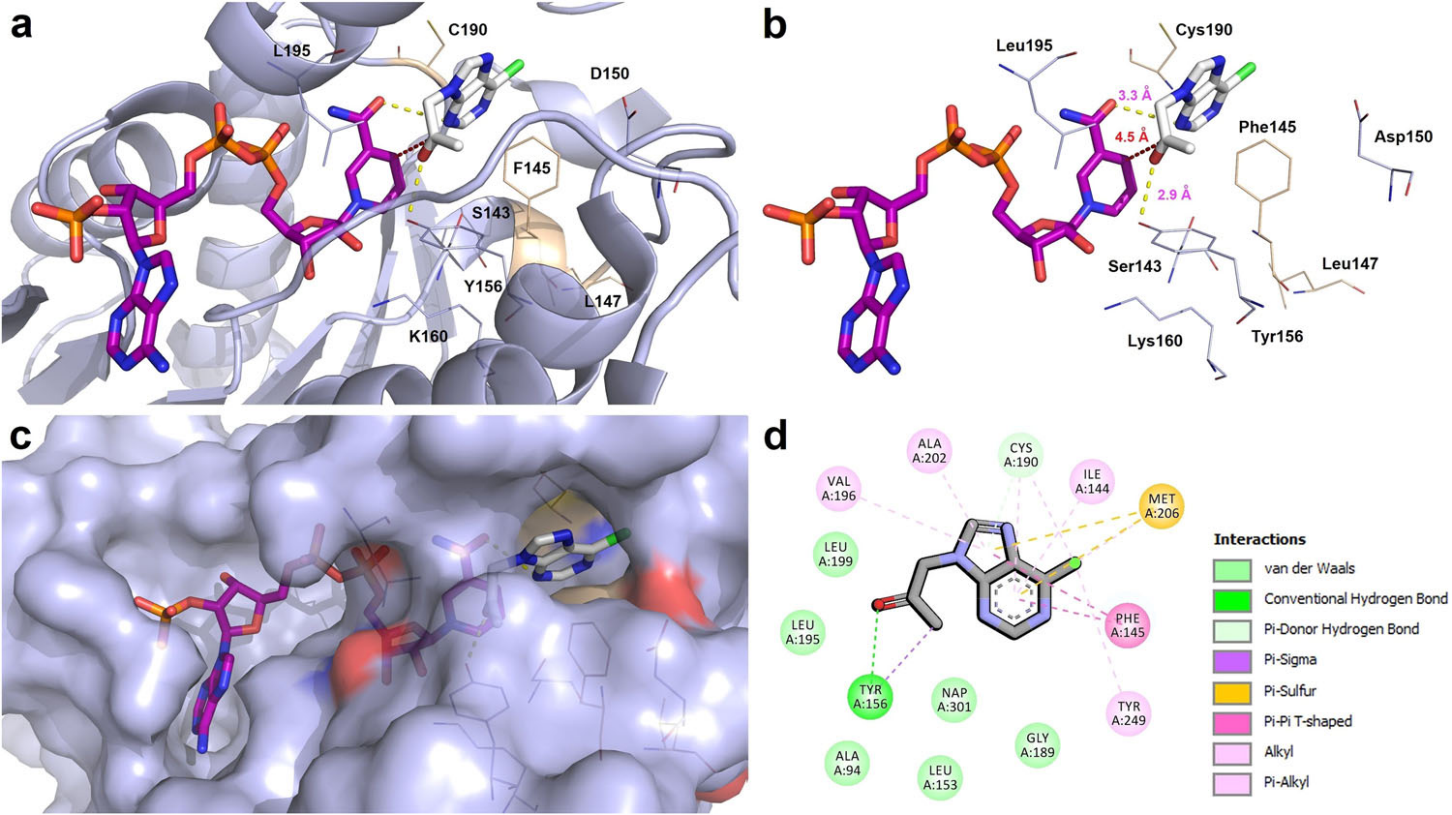
Binding mode of 1-(6-chloro-9*H*-purin-9-yl)propan-2-one (**1w**) with Lk-ADH Prince prepared from Lk-ADH (PDB code: 4RF2), with close contacts to amino acid residues and NADPH cofactor located in the active site. The docked ligand **1w** and the cofactor are shown as *sticks* representation, where **1w** is white, and NADPH is violet. The overall receptor structure is shown as a semi-transparent light-blue *cartoon* (**a**) or *sphere* diagram (**c**), respectively. The most significant amino acid (AA) residues contributing to the stabilization of the ketone **1w** in the complex with Lk-ADH Prince are shown in light-blue (for conserved AA residues) and gold (for mutated AA residues) *lines* representations. Nitrogen atoms are presented with blue color, oxygen atoms with red color, chlorine atoms with green color, whereas phosphorus atoms with orange color. All the hydrogens were omitted for clarity. The formation of intermolecular hydrogen bonds is represented by yellow dashed lines, whereas the plausible trajectory of the hydride transfer from NADPH to a carbon atom of the carbonyl group is shown as a red dashed line. Mutual distances between the amino acid residues and the respective ligand’s atoms are given in Ångström (**b**). The figures shown in panels **a**–**c** were prepared using the program PyMOL (http://www.pymol.org/). **d** The 2D protein-ligand interactions map was generated using the program BIOVIA Discovery Studio Visualizer 20.1.0.19295 (Dassault Systèmes Biovia Corp.; https://www.3ds.com). In this case, polar contacts between receptor-ligand including conventional hydrogen bonds (green) and π−donor hydrogen bonds (green-pastel), as well as non-polar contacts between receptor-ligand including π–σ interactions (purple/violet), π–alkyl (light pink), π–π T-shaped (pink), and π–sulfur (gold) are represented by dashed lines. Intermolecular Van der Waals forces are displayed in light-green spoked arcs.

Since ketone **1w** was a perfect substrate for Lk-ADH Prince, we found it interesting to verify how this compound accommodates in the enzyme active site and interacts with amino acid residues to form catalytically productive pose leading to anti-Prelog product (*R*)-**2w**. For this purpose, prochiral ketone (*R*)-**2w** was docked with Lk-ADH Prince, which was prepared from Lk-ADH from *Lactobacillus kefir* (PDB code: 4RF2)^116^ using standard UCSF Chimera mutations/rotamers options according to dynameomics backbone-independent rotamer library elaborated by A.D. Scouras and V. Dagget^117^ (For details, see ‘Supplementary Methods 4. Molecular docking’ deposited in Supplementary Information). The visualization of the selected top-scoring docking pose of **1w** to Lk-ADH Prince with close contact with amino acid residues located in the engineered cavity (with mutated positions shown as gold *lines* representation) is presented in Fig. 6.

**Fig. 7:**
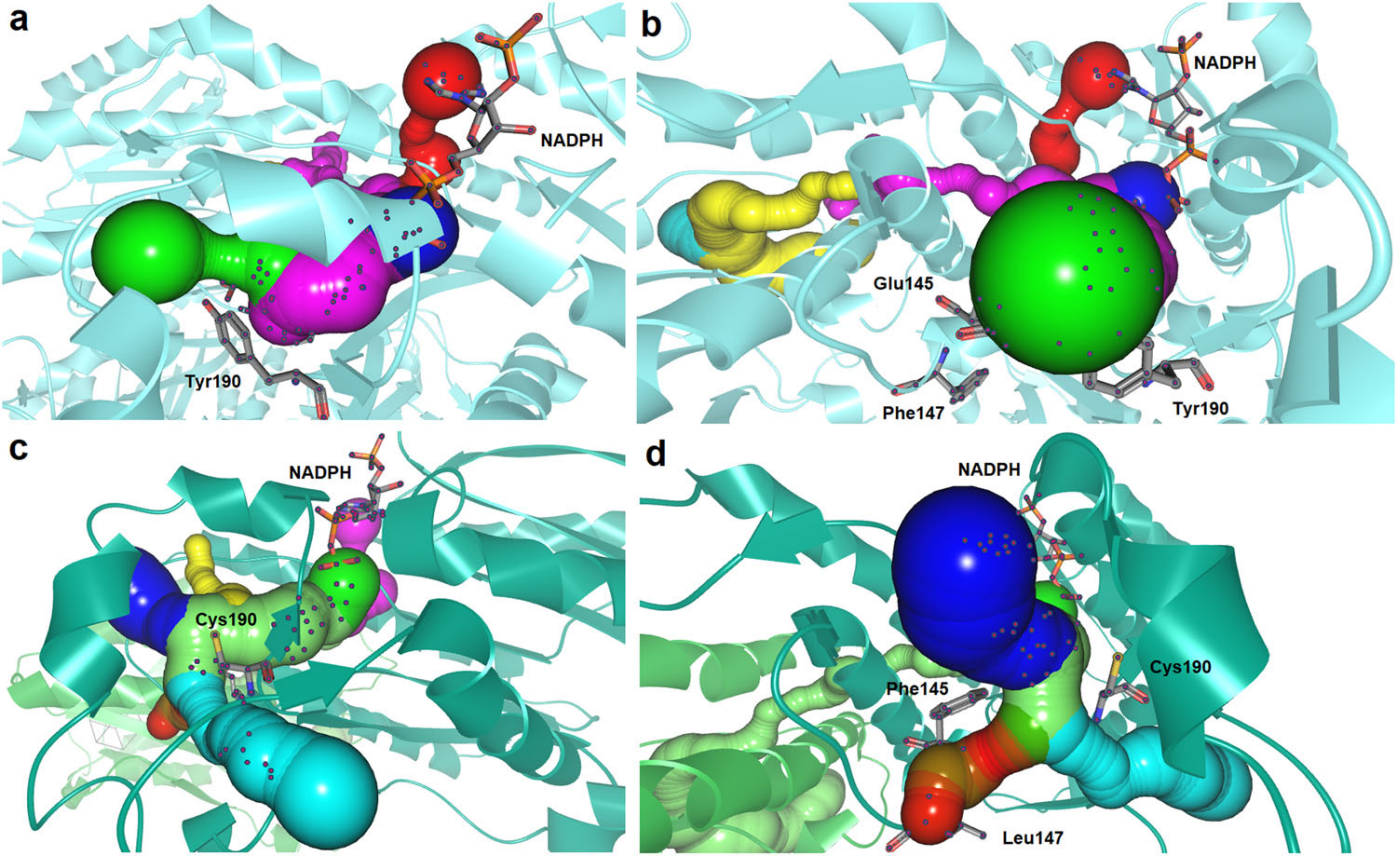
Visualization of the tunnels in wild-type Lk-ADH (panel a and b) and Lk-ADH Prince (panel c and d) represented as a set of intersecting spheres using CAVER Analyst 2.0 software. Indicates the access pathways to buried active sites in Lk-ADH and Lk-ADH Prince, respectively. The overall enzyme structures are shown as a semi-transparent cyan (**a**, **b**) or green (**c**, **d**) *cartoon*, respectively. The most significant amino acid residues responsible for the formation of the tunnels and bottlenecks, as well as the NADPH cofactor, are represented by *sticks*. For details concerning tunnel statistics, see Supplementary Table 9 deposited in Supplementary Information.

The hypothetical complex of **1w** with Lk-ADH Prince suggests that the ketone accommodates inside the substrate-binding pocket with a pose that promotes the (*R*)-stereoselective reduction of the prochiral carbonyl group. In this regard, the three-dimensional architecture of the engineered active site of Lk-ADH Prince enforces the orientation of the 6-chloro-9*H*-purine moiety of the substrate perpendicular to NADPH; thus, the methyl group is pulled out into the smaller binding cleft near the catalytic triad consisting of Ser143-Tyr156-Lys160. Moreover, a strong 2.9 Å-long hydrogen bond formed between the oxygen atom of the prochiral carbonyl group of **1w** and Tyr156, as well as an additional 3.3 Å-long H-bonding between the *N*-3 atom of the purine ring and the amide group of the cofactor’s nicotinamide moiety favored the orientation of the ketone with *si*-face exposed toward the reactive hydride of the NADPH. A deeper inspection of the 2D protein-ligand interactions map (Fig. 6d) revealed that mutated Phe145 forms π–π T-shaped interaction with a purine moiety of **1w**. This interaction allows anchoring the purine moiety in the broader part of the active site cavity while leaving the carbon atom of the carbonyl group closer to the NADPH cofactor. On the other hand, the peripheral Phe147 to Leu147 and Tyr190 to Cys190 mutations allow for avoiding unfavorable steric clashes between the substrates with larger (hetero)aromatic substituents (i.e., **1a,** **1d,** **1g,** **1i,** **1r,** **1u**, and **1v**) and the amino acid residues located at the entrance to the Lk-ADH Prince active site, which is in line with the experimental results. Accordingly, a non-polar substrate channel with an open entrance is also essential for the catalytic performance of Lk-ADH Prince toward “bulky–bulky” ketones, including **1y,** **1o,** **1x,** **1z,** **1aa**, and **1af**. This hypothesis was confirmed by additional in silico calculations using CAVER Analyst 2.0 software^118^, which allowed mapping and in-depth analysis of the 3D architecture of wild-type and engineered enzymes’ tunnels and entrances to the binding sites (Fig. 7 or Supplementary Fig. 2 deposited in Supplementary Information).

The comparison of the access pathways to buried active sites in both studied proteins revealed that altering the Tyr190 with Cys190 significantly enlarges the radius of a central tunnel in the mutated protein (Fig. 7c, d, blue color sphere) and, thus, potentially enhances the accessibility of Lk-ADH Prince for various bulky–bulky substrates. Moreover, such mutation also promotes the creation of an alternative tunnel leading from the protein surface to the core (Fig. 7c, d, magenta color sphere), which might additionally influence the substrate and product diffusion. The average bottleneck radius for the central tunnel and the supplemental tunnel are 1.9 Å and 1.3 Å, respectively. In turn, when comparing the shape of the wild-type Lk-ADH tunnel with the mutated variant, it is clear that its middle part is narrowed by Tyr190 residue to form a bottleneck of 1.3 Å average radius, thus hindering the efficient passage of more bulky substrate molecules into the active site (Fig. 7a, b, green color sphere). For details concerning tunnels’ statistics, please see Supplementary Table 9 deposited in Supplementary Information. Considering these facts, we believe that further studies on the rational re-design of amino acid residues located in the vicinity of the respective tunnels are required to obtain functional (tailored) Lk-ADH variants with facilitated transport pathways for small molecules and other structurally demanding substrates.

## Conclusions

In conclusion, the substrate scope of an engineered variant of the alcohol dehydrogenase from *Lactobacillus kefir* E145F-F147L-Y190C, named Lk-ADH Prince, was investigated. The enzyme was heterologously overexpressed in *E. coli* cells and used as lyophilized whole-cell preparation catalyzing the asymmetric transfer biohydrogenation of a broad range of carbonyl compounds to afford alcohol products in high isolated yield (up to 91%) and with excellent enantiomeric excesses (up to >99% ee) without the requirement of expensive exogenous NAD(P)H cofactor addition. Noteworthy, the studied biocatalyst tolerates substrate loadings up to 100 mM conc. and organic solvent concentrations (up to 30% v/v) without compromising the conversion and stereoselectivity of the obtained optically active products. Moreover, experimental work revealed that replacing sterically hindered aromatic amino acids (Phe147 and Tyr190) with those possessing smaller aliphatic residues (Lys147 and Cys190) located at the entrance to the active site of the wild-type Lk-ADH allowed more bulky substrates to be accessible for the engineered enzyme. These data demonstrate that *E. coli*/Lk-ADH Prince can be an excellent biocatalyst for the asymmetric synthesis of industrially valuable enantiomers of chiral alcohols, including difficult-to-obtain sterically demanding compounds. Taken altogether, we are highly convinced that using *E. coli*/Lk-ADH Prince will blaze new trials for other beneficial biosynthetic campaigns and provide preparative (bio)organic chemists with a practical tool to be included in the route scouting.

## Methods

### Overexpression of Lk-ADH Prince

#### Cloning

The corresponding gene was ordered from BioCat GmbH as the optimized sequence for overexpression in *E. coli* BL21(DE3) and cloned into a pASK-IBA5Plus vector with N-terminal Strep-II tag by restriction enzyme cloning (restriction sites BsaI). Successful cloning was confirmed by sequencing (see below).

#### Protein expression

For expression of the Lk-ADH-Prince in *E. coli* BL21(DE3) (New England Biolabs) LB medium (Luria Broth) supplemented with ampicillin (100 μg mL^-1^ final concentration) and MgCl_2_ (1 mM final concentration) was inoculated with 1% overnight culture (15 mL LB medium supplemented with ampicillin) and incubated at 37 °C and 120 rpm to an OD600 of 0.6. Expression was induced by the addition of AHTC (anhydrotetracycline, 0.4 μM final concentration) and conducted at 20 °C, 120 rpm overnight. The cells were harvested by centrifugation (20 min, 4 °C, 4000 rpm) and freeze-dried using a lyophilizer to obtain *E. coli*/Lk-ADH Prince preparation.

### DNA and protein sequences of Lk-ADH Prince

#### Native amino acid sequence

MTDRLKGKVAIVTGGTLGIGLAIADKFVEEGAKVVITGRHADVGEKAAKSIGGTDVIRFVQHDASDEAGWTKLFDTTEEAFGPVTTVVNNAGIAVSKSVEDTTTEEWRKLLSVNLDGVFFGTRLGIQRMKNKGLGASIINMSSIFGLVGDPTLGAYNASKGAVRIMSKSAALDCALKDYDVRVNTVHPGCIKTPLVDDLEGAEEMMSQRTKTPMGHIGEPNDIAWICVYLASDESKFATGAEFVVDGGYTAQ.

#### Amino acid sequence including Strep-Tag as used in this study

MASWSHPQFEKGAETMTDRLKGKVAIVTGGTLGIGLAIADKFVEEGAKVVITGRHADVGEKAAKSIGGTDVIRFVQHDASDEAGWTKLFDTTEEAFGPVTTVVNNAGIAVSKSVEDTTTEEWRKLLSVNLDGVFFGTRLGIQRMKNKGLGASIINMSSIFGLVGDPTLGAYNASKGAVRIMSKSAALDCALKDYDVRVNTVHPGCIKTPLVDDLEGAEEMMSQRTKTPMGHIGEPNDIAWICVYLASDESKFATGAEFVVDGGYTAQ.

#### Codon-optimized DNA sequence used

atggctagctggagccacccgcagttcgaaaaaggcgccgagaccATGACCGATCGTCTGAAAGGTAAAGTTGCAATTGTTACCGGTGGCACCTTAGGTATTGGTCTGGCAATTGCAGATAAATTTGTTGAAGAAGGTGCCAAAGTTGTTATTACCGGTCGTCATGCAGATGTTGGTGAAAAAGCAGCAAAAAGCATTGGTGGCACCGATGTTATTCGTTTTGTTCAGCATGATGCAAGTGATGAAGCAGGTTGGACCAAACTGTTTGATACCACCGAAGAAGCATTTGGTCCGGTTACCACCGTTGTTAATAATGCAGGTATTGCAGTTAGCAAGAGCGTTGAAGATACCACCACAGAAGAATGGCGTAAACTGCTGAGCGTTAATCTGGATGGTGTTTTTTTTGGCACCCGTCTGGGTATTCAGCGTATGAAAAACAAAGGTCTGGGTGCCAGCATTATCAATATGAGCAGCATTTTTGGTCTGGTTGGTGATCCGACACTGGGTGCATATAATGCAAGCAAAGGTGCAGTTCGTATTATGAGCAAAAGCGCAGCACTGGATTGTGCACTGAAAGATTATGATGTTCGTGTGAATACCGTTCATCCGGGTTGTATTAAAACACCGCTGGTTGATGATCTGGAAGGTGCCGAAGAAATGATGAGCCAGCGTACCAAAACACCGATGGGTCATATTGGTGAACCGAATGATATTGCCTGGATTTGTGTTTATCTGGCCAGTGATGAAAGTAAATTTGCGACCGGTGCCGAATTTGTTGTTGATGGTGGTTATACCGCACAGTAAggtctctgatatctaactaagcttgacctg.

### General procedure for *E. coli*/Lk-ADH Prince-catalyzed bioreductions of prochiral carbonyl substrates 1a, 1d, 1g, 1i, 1j, 1l, 1o, 1r, 1u, 1v, 1w, 1x, 1y, 1z, 1aa, and 1af

#### General procedure A (0.40 mmol-scale)

*E. coli*/Lk-ADH Prince (60 mg) was suspended in 50 mM Tris–HCl buffer (2.4–3.6 mL, pH 7.5) containing 1.0 mM MgCl_2_ and preincubated for 30 min at 30 °C. Then, the respective ketone **1a,** **1d,** **1i,** **1j,** **1o,** **1r,** **1u,** **1z**, or **1aa** (0.40 mmol, 100 mM final concentration), 2-propanol (400 μL, 10% v/v) and DMSO (0–20% v/v) were added to the mixture. The reaction was shaken (250 rpm) at 30 °C for 24 h and then stopped by extraction with EtOAc (3 × 15 mL). The organic layers were combined and dried over anhydrous MgSO_4_. After filtering off the drying agent and evaporating the volatiles, the crude residue was purified by column chromatography on SiO_2_ gel using a mixture of the appropriate eluent (see section ‘Supplementary physico-chemical data of the products’ deposited in Supplementary Information) for each derivative to afford the desired optically active products: (*R*)-**2a** (96 mg, 86% yield, 99% ee); (*R*)-**2d** (35 mg, 51% yield, 99% ee); (*R*)-**2i** (75 mg, 73% yield, >99% ee); (*R*)-**2j** (34 mg, 62% yield, 93% ee); (*R*)-**2o** (30 mg, 41% yield, 88% ee); (*R*)-**2r** (39 mg, 79% yield, 96% ee); (*R*)-**2u** (62 mg, 76% yield, >99% ee); (*S*)-**2z** (45 mg, 68% yield, 94% ee); (*S*)-**2aa** (48 mg, 67% yield, >99% ee). For details, see Table 1.

#### General procedure B (0.10 mmol-scale)

*E. coli*/Lk-ADH Prince (60 mg) was suspended in 50 mM Tris–HCl buffer (2.4–3.6 mL, pH 7.5) containing 1.0 mM MgCl_2_ and preincubated for 30 min at 30 °C. Then, the respective ketone **1g,** **1l,** **1v,** **1x,** **1y**, or **1af** (0.10 mmol, 25 mM final concentration), 2-propanol (400 μL, 10% v/v), and DMSO (0–20% v/v) were added to the mixture. The reaction was shaken (250 rpm) at 30 °C for 24 h and then stopped by extraction with EtOAc (3 × 15 mL). The organic layers were combined and dried over anhydrous MgSO_4_. After filtering off the drying agent and evaporating the volatiles, the crude residue was purified by column chromatography on SiO_2_ gel using a mixture of the appropriate eluent (see section ‘Supplementary physico-chemical data of the products’ deposited in Supplementary Information) for each derivative to afford the desired optically active products: (*R*)-**2g** (13 mg, 66% yield, >99% ee); (*R*)-**2l** (10 mg, 67% yield, 98% ee); (*R*)-**2v** (16 mg, 67% yield, >99% ee); (*S*)-**2x** (9 mg, 38% yield, 95% ee); (*S*)-**2y** (16 mg, 59% yield, 99% ee); (*S*)-**2af** (8 mg, 40% yield, >99% ee). For details, see Table 1.

#### General procedure C (0.40 mmol-scale with modified work up for the product strongly soluble in H_2_O)

*E. coli*/Lk-ADH Prince (60 mg) was suspended in 50 mM Tris–HCl buffer (3.6 mL, pH 7.5) containing 1.0 mM MgCl_2_ and preincubated for 30 min at 30 °C. Then, a ketone **1w** (0.40 mmol, 100 mM final concentration) and 2-propanol (400 μL, 10% v/v) were added to the mixture. The reaction was shaken (250 rpm) at 30 °C for 24 h and then stopped by azeotropic evaporation of H_2_O with PhCH_3_ (25 ml). The crude residue was dissolved in CH_2_Cl_2_ and dried over anhydrous MgSO_4_. After filtering off the drying agent and evaporating the volatiles, the crude residue was purified by column chromatography on SiO_2_ gel using a mixture of CH_2_Cl_2_/MeOH (95:5, v/v) as an eluent to afford the desired optically active (*R*)-**2w** (77 mg, 91% yield, >99% ee). For details, see Table 1.

### Reporting summary

Further information on research design is available in the Nature Portfolio Reporting Summary linked to this article.

### Supplementary information

Supplementary Information

Description of Additional Supplementary Files

Supplementary Data 1

Supplementary Data 2

Supplementary Data 3

Supplementary Data 4

Reporting Summary

## Data Availability

All data generated or analyzed during this study are included in this published article (and its supplementary information files). The data that support the findings of this study i.e., supplementary methods, detailed experimental procedures and characterizations of new compounds, synthetic procedures for optimization studies of the enzymatic reaction parameters, protocols for molecular docking studies, physicochemical data of the products, as well as analytical separation conditions of the studied compounds by GC and HPLC analytical methods, are available in the Supplementary Information. Analytical separation conditions of studied compounds by GC, see Supplementary Table 11 in the Supplementary Information. HPLC analytical separation conditions of racemates by chiral columns, see Supplementary Table 12 in the Supplementary Information. The origin of the chemicals used in this study, see Supplementary Table 14 in the Supplementary Information. For NMR, FTMS, and ATR-FTIR spectra, see Supplementary Data 1 (Supplementary Figs. 3–140). The list of the Cartesian coordinates for the optimized structure of 1-(6-chloro-9*H*-purin-9-yl)propan-2-one (**1w**), see Supplementary Data 2. Protein and DNA sequences of alcohol dehydrogenase variant deduced from *Lactobacillus kefir* (Lk-ADH Prince), see Supplementary Data 3. For copies of HPLC chromatograms, see Supplementary Data 4 (i.e., Supplementary Figs. 141–215).
