# Supplementary material for: Biocatalytic characterization of an alcohol dehydrogenase variant deduced from Lactobacillus kefir in asymmetric hydrogen transfer
Source: Commun Chem. 2023 Oct 12;6:217. doi: 10.1038/s42004-023-01013-1 (PMC10570314; doi:10.1038/s42004-023-01013-1)
Supplement: Supplementary file 1 — Supplementary Information [file 42004_2023_1013_MOESM1_ESM.pdf]

Supplementary Information

Biocatalytic Characterization of an Alcohol Dehydrogenase Variant Deduced from *Lactobacillus kefir* in Asymmetric Hydrogen Transfer

Aleksandra Rudzka,[1] Beata Zdun,[1] Natalia Antos,[1] Lia Martínez Montero,[2] Tamara Reiter,[2] Wolfgang Kroutil[2] and Paweł Borowiecki[1,]*

[1] Laboratory of Biocatalysis and Biotransformation, Department of Drugs Technology and Biotechnology, Faculty of Chemistry, Warsaw University of Technology, Koszykowa 75, 00–662 Warsaw, Poland.

[2] Institute of Chemistry, University of Graz, NAWI Graz, BioTechMed Graz, Field of Excellence BioHealth, Heinrichstrasse 28, 8010 Graz, Austria.

*Corresponding author: Dr. Paweł Borowiecki (Email: pawel.borowiecki@pw.edu.pl; Website: http://lbb-wut-borowiecki.ch.pw.edu.pl/)

Table of Contents

1. Supplementary Methods. General information ……………………………………………………... 3–4
2. Supplementary Methods 1. Overexpression of Lk-ADH Prince ……………..………………... 5–6
3. Supplementary Methods 2. Synthetic procedures for the obtained compounds ………......… 7–12
4. Supplementary Methods 3. Optimization of the reaction parameters ……………………..…. 13–16
Supplementary Table 1. Screening conditions for the *E. coli*/Lk-ADH Prince-catalyzed asymmetric bioreduction of the model pentoxifylline (**1a**) after 24 h …………………..………………..….. 13
Supplementary Table 2. *E. coli*/Lk-ADH Prince-catalyzed bioreduction of pentoxifylline (**1a**) after 24 h – effect of the substrate concentration …………………..……………………………..…..... 13
Supplementary Table 3. *E. coli*/Lk-ADH Prince-catalyzed bioreduction of pentoxifylline (**1a**) after 24 h – effect of the 2-PrOH concentration ……………………………………..…………….……….. 14
Supplementary Table 4. Preparative-scale *E. coli*/Lk-ADH Prince-catalyzed bioreduction of pentoxifylline (**1a**) after 24 h – effect of the amount of *E. coli*/Lk-ADH Prince cells …..………..….. 14
Supplementary Table 5. *E. coli*/Lk-ADH Prince-catalyzed bioreduction of 1-(biphenyl-4-yl)ethanone (**1g**) after 24 h – effect of the co-solvent ……………………..………..……………….…….. 15
Supplementary Table 6. *E. coli*/Lk-ADH Prince-catalyzed bioreduction of 1,2-diphenylethanone (**1af**) after 24 h – effect of the co-solvent ……………………………..……..…………………..…….. 15
Supplementary Table 7. *E. coli*/Lk-ADH Prince-catalyzed bioreduction of 1-(biphenyl-4-yl)ethanone (**1g**) after 24 h – effect of the substrate concentration ……………….……..………………..….. 16
Supplementary Table 8. *E. coli*/Lk-ADH Prince-catalyzed bioreduction of pentoxifylline (**1af**) after 24 h – effect of the substrate concentration ……………………..…………..………………..….. 16
Supplementary Figure 1. Grouping optical purity spectrum for the tested *E. coli*/Lk-ADH Prince toward carbonyl substrates ……….……………………….…………………………….……..…... 17
5. Supplementary Methods 4. Molecular docking …………….……..…………………..…... 18–19
Supplementary Figure 2. Visualization of the tunnels in wild-type Lk-ADH (panel a and b) and Lk-ADH Prince (panel c and d) represented as a set of intersecting spheres using CAVER Analyst 2.0 software ……………………………………………..………………..……………………... 20
Supplementary Table 9. Tunnel statistics calculated with CAVER Analyst 2.0 software ……......... 20
Supplementary Table 10. Chiral building blocks (obtained in this work) useful for the synthesis of APIs and/or natural products …………………………………….……….………….……….... 21–22
6. Supplementary physico-chemical data of the products ……..……………………….……….. 23–33
Supplementary Table 11. Analytical separation conditions of studied compounds by GC column ………………...……………………………………….……………………..…...…… 34–37
Supplementary Table 12. HPLC analytical separation conditions of racemates by chiral columns ……………………..…………………………………………………………………..….. 38–40
Supplementary Table 13. Determination of the absolute configuration based on the elution order of the HPLC peaks separated by chiral columns ………………….. 41–43
Supplementary Table 14. The origin of the chemicals used in this study …........................….... 44–46
7. Supplementary references ……………………………....……..………………………….……...... 47

1. Supplementary Methods.

General information.

β-Nicotinamide adenine dinucleotide, disodium salt, hydrate, 95+%, reduced form (NADH) was purchased from Across Organics (Cat. No.: 271100010); β-nicotinamide adenine dinucleotide 2′-phosphate reduced tetrasodium salt (NADPH) was purchased from AmBeed (Cat. No.: A341469). Chromatography grade *n*-hexane and 2-propanol (2-PrOH) used in high-performance liquid chromatography (HPLC) were purchased from Avantor Performance Materials Poland S.A. (formerly POCH Polish Chemicals Reagents). All other commercially available reagents, including starting materials (ketones) **1a**–**ah** and optically active alcohols {i.e., (*S*)-1-phenyl ethanol [(*S*)-**2b**], (*S*)-1-phenyl-1-propanol [(*S*)-**2c**], (*S*)-α-methyl-2-naphtalenemethanol [(*S*)-**2d**], (*R*)-1-indanol [(*R*)-**2e**], (*R*)-1,2,3,4-tetrahydro-1-napthol [(*R*)-**2f**], (*R*)-1-phenyl-2-propanol [(*R*)-**2j**], (*R*)-1-phenyl-3-butanol [(*R*)-**2k**], (*S*)-1-(2-furyl)ethanol [(*R*)-**2p**], and (*R*)-1-phenyl-1-butanol [(*R*)-**2ag**]} used as analytical standards for chiral HPLC [purchased from Merck KGaA or MERCK Schuchardt (Darmstadt, Germany), TCI (Tokyo Chemical Industry), Thermo Fisher (Kandel) GmbH (Kandel, Germany), Alfa Aesar (Kandel, Germany), Fluorochem Ltd. (Hadfield Derbyshire, United Kingdom), AmBeed (Arlington Hts, IL 60004, USA), Angene Chemical (London, England), Santa Cruz Biotechnology, Inc. (Heidelberg, Germany), BLD Pharmatech (India) Pvt Ltd. (Hajdarabad, India), and Enamine Ltd. (Kyiv, Ukraine)] were used without further purification. The details concerning the manufacturers and catalog numbers of the respective ketones **1a**–**ah** are given in **Supplementary Table 14**.

Analytical thin-layer chromatography was carried out on TLC aluminum plates with silica gel Kieselgel 60 F_{254} (Merck, Germany) (0.2 mm thickness film containing a fluorescence indicator green 254 nm (F254) using UV light as a visualizing agent.

Preparative separations were carried out by column chromatography using Merck silica gel 60 (230–400 mesh), with grain size 40–63 μm.

The gas chromatography (GC) analyses were performed with an Agilent Technologies 6890N instrument (Maryland, United States) equipped with a flame ionization detector (FID) and fitted with HP-50+ (30 m) semi-polar column (50 % phenyl–50 % methylpolysiloxane); the GC injector was maintained at 250 °C; Helium (2 mL/min) was used as carrier gas; retention times (t_R) are given in minutes under these conditions; column temperature programs are given in **Supplementary Table 11**.

The enantiomeric excesses (% ee) of biocatalytic reduction products were determined by high-performance liquid chromatography (HPLC) analyses performed on Shimadzu Nexera-*i* (LC-2040C 3D) equipped with a photodiode array detector (PAD) using columns packed with chiral stationary phases as follows: Chiralpak AD-H, Chiralcel OD-H, or Chiralcel OJ-H (4.6 mm × 250 mm, coated on 5 μm silica gel grain size, from Daicel Chemical Ind., Ltd., Japan) or Lux i-Cellulose-5 (Phenomenex®, USA) equipped with dedicated pre-columns (4 mm × 10 mm, 5 μm); the respective mixtures of *n*-hexane/2-PrOH or *n*-hexane/2-PrOH/diethylamine (DEA)

were used as mobile phases in the appropriate ratios; the HPLC analyses were executed in an isocratic, and isothermal (25 °C or 30 °C) mode; flow (*f*) is given in mL/min; the method conditions for the resolution of the appropriate racemates are shown in **Supplementary Table 12**.

^{1}H NMR (500 MHz), $^{13}C\{^{1}H\}$ NMR (126 MHz), and $^{19}F\{^{1}H\}$ NMR (470 MHz) spectra were recorded on a Varian NMR System 500 MHz spectrometer; The ^{1}H, $^{13}C\{^{1}H\}$, and $^{19}F\{^{1}H\}$ chemical shifts (δ) are reported in parts per million (ppm) relative to the (i) solvent signals: $CDCl_3$, δ_H (residual $CHCl_3$) 7.26 ppm, δ_C 77.16 ppm or (ii) internal $CFCl_3$ reference set at 0 ppm in case of δ_F. Chemical shifts are quoted as s (singlet), d (doublet), dd (doublet of doublets), t (triplet), q (quartet), m (multiplet), and br. s (broad singlet); coupling constants (*J*) are reported in Hertz (Hz). Raw spectroscopic data have been processed with MestReNova (Product version: 6.0.2-5475).

Fourier Transform Mass Spectrometry (FTMS) spectra were recorded on Q Exactive Hybrid Quadrupole-Orbitrap Mass Spectrometer, ESI source: electrospray with spray voltage 4.00 kV; all samples were prepared by dilution with MeOH (0.5 mL) and addition of a mixture of $CH_3CN/MeOH/H_2O$ (50:25:25, v/v/v) + 0.5% formic acid (HCOOH) each.

Fourier transform infrared (FTIR) spectra of neat samples were recorded on an IRSpirit™ Fourier Transform Infrared Spectrophotometer Instrument with a DLATGS detector from Shimadzu Corp. equipped with the QATR™-S single-reflection attenuated total reflectance (ATR) measurement attachment with a monolithic diamond crystal stage (high-throughput) as prism material and a pressure clamp; the incident angle is 45 degrees with 4 cm^{-1} resolution and accumulation of 32 scans, 4000–400 cm^{-1} recording range; absorption maxima (ν_{max})/wavenumber (frequency, ν) are quoted in cm^{-1}; apodization Happ-Genzel. The abbreviation "br" denotes broad.

2. Supplementary Methods 1. Overexpression of Lk-ADH Prince.

2.1. DNA and protein sequences of Lk-ADH Prince Lactobacillus kefir

Abbr.	Organism	Vector	Restriction site	Tag	pEG-Number[a]
**Lk-ADH Prince**	*L. kefir*	IBA5Plus	BsaI, BsaI	N-term. Strep-II	pEG 476

[a] Internal plasmid number; for inquiries about plasmids, please refer to this number.

2.2. Lk-ADH Prince Lactobacillus kefir

Native amino acid sequence:
MTDRLKGKVAIVTGGTLGIGLAIADKFVEEGAKVVITGRHADVGEKAAKSIGGTDVI
RFVQHDASDEAGWTKLFDTTEEAFGPVTTVVNNAGIAVSKSVEDTTTEEWRKLLSV
NLDGVFFGTRLGIQRMKNKGLGASIINMSSIFGLVGDPTLGAYNASKGAVRIMSKSA
ALDCALKDYDVRVNTVHPGCIKTPLVDDLEGAEEMMSQRTKTPMGHIGEPNDIAWI
CVYLASDESKFATGAEFVVDGGYTAQ

Amino acid sequence including Strep-Tag as used in this study:
MASWSHPQFEKGAETMTDRLKGKVAIVTGGTLGIGLAIADKFVEEGAKVVITGRHA
DVGEKAAKSIGGTDVIRFVQHDASDEAGWTKLFDTTEEAFGPVTTVVNNAGIAVSKS
VEDTTTEEWRKLLSVNLDGVFFGTRLGIQRMKNKGLGASIINMSSIFGLVGDPTLGAY
NASKGAVRIMSKSAALDCALKDYDVRVNTVHPGCIKTPLVDDLEGAEEMMSQRTKT
PMGHIGEPNDIAWICVYLASDESKFATGAEFVVDGGYTAQ

Codon-optimized DNA sequence used:
atggctagctggagccacccgcagttcgaaaaaggcgccgagaccATGACCGATCGTCTGAAAGGTAAAG
TTGCAATTGTTACCGGTGGCACCTTAGGTATTGGTCTGGCAATTGCAGATAAATTT
GTTGAAGAAGGTGCCAAAGTTGTTATTACCGGTCGTCATGCAGATGTTGGTGAAA
AAGCAGCAAAAAGCATTGGTGGCACCGATGTTATTCGTTTTGTTCAGCATGATGC
AAGTGATGAAGCAGGTTGGACCAAACTGTTTGATACCACCGAAGAAGCATTTGGT
CCGGTTACCACCGTTGTTAATAATGCAGGTATTGCAGTTAGCAAGAGCGTTGAAG
ATACCACCACAGAAGAATGGCGTAAACTGCTGAGCGTTAATCTGGATGGTGTTTT
TTTTGGCACCCGTCTGGGTATTCAGCGTATGAAAAACAAAGGTCTGGGTGCCAGC
ATTATCAATATGAGCAGCATTTTTGGTCTGGTTGGTGATCCGACACTGGGTGCAT
ATAATGCAAGCAAAGGTGCAGTTCGTATTATGAGCAAAAGCGCAGCACTGGATT
GTGCACTGAAAGATTATGATGTTCGTGTGAATACCGTTCATCCGGGTTGTATTAA
AACACCGCTGGTTGATGATCTGGAAGGTGCCGAAGAAATGATGAGCCAGCGTAC
CAAAACACCGATGGGTCATATTGGTGAACCGAATGATATTGCCTGGATTTGTGTT
TATCTGGCCAGTGATGAAAGTAAATTTGCGACCGGTGCCGAATTTGTTGTTGATG
GTGGTTATACCGCACAGTAAggtctctgatatctaactaagcttgacctg

2.3. Cloning

The enzyme was selected based on a literature search. The corresponding gene was ordered from BioCat GmbH as an optimized sequence for overexpression in *E. coli* BL21(DE3) and cloned into a pASK-IBA5Plus vector with N-terminal Strep-II tag by restriction enzyme cloning (restriction sites BsaI). Successful cloning was confirmed by sequencing.

2.4. Protein expression

For expression of the Lk-ADH-Prince in *E. coli* BL21(DE3) (New England Biolabs) LB medium (Luria Broth) supplemented with ampicillin (100 µg mL^{-1} final concentration) and $MgCl_2$ (1 mM final concentration) was inoculated with 1% overnight culture (15 mL LB medium supplemented with ampicillin) and incubated at 37 °C and 120 rpm to an OD600 of 0.6. Expression was induced by the addition of AHTC (anhydrotetracycline, 0.4 µM final concentration) and conducted at 20 °C, 120 rpm overnight. The cells were harvested by centrifugation (20 min, 4 °C, 4000 rpm) and freeze-dried using a lyophilizer to obtain *E. coli*/Lk-ADH Prince preparation.

2.5. SDS-Page

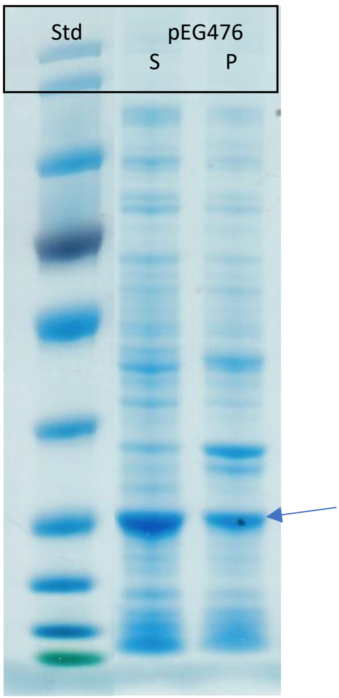

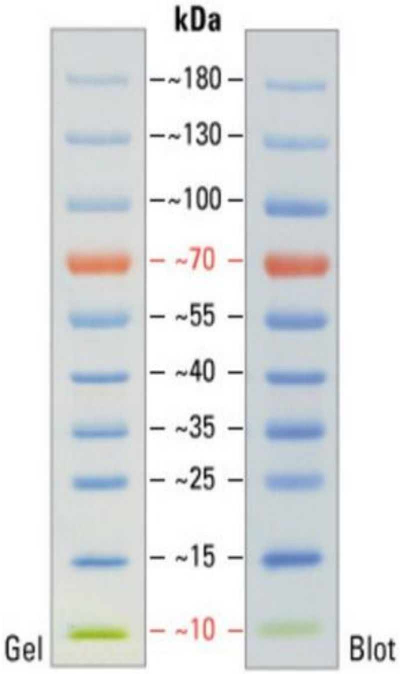

S : supernatant ; P : pellet

3. Supplementary Methods 2. Synthetic procedures for the obtained compounds.

3.1. General procedure for the synthesis of racemic alcohols rac-2a–ah

In a round bottom flask, the appropriate carbonyl compound **1a**–**ah** (2.0 mmol) was dissolved in MeOH (10 mL) and cooled to 0–5 °C with an ice bath. Next, $NaBH_4$ (1.0 mmol) was added portion-wise, and after the addition, the reaction mixture was allowed to warm to room temperature and stirred for 2 h. Subsequently, the reaction was quenched with a saturated aqueous solution of NH_4Cl (10 mL), and the methanol was removed using the rotary evaporator. The remaining solution was extracted with EtOAc (3 × 15 mL), and the collected organic phases were rinsed with brine (20 mL). After drying the organic layer over anhydrous Na_2SO_4, filtration of the drying agent, and evaporation of the solvent, the crude oil was subjected to column chromatography on SiO_2 gel using the respective *n*-hexane/EtOAc mixture or petroleum ether/Et_2O as eluent to afford the desired *rac*-**2a**–**ah**.

3.2.1. Stereoselective bioreduction of pentoxyphylline (1a) – Screening procedures

Method A (Screening conditions):

E. coli/Lk-ADH Prince (10 mg) was suspended in 50 mM Tris–HCl buffer (450 µL, pH 7.5; see entries 1–4 in **Supplementary Table 1**) or distilled H_2O (450 µL; see entries 5–8 in **Table 1**) containing 0.5 mM NADH (entries 2 and 6 in **Supplementary Table 1**) or 0.5 mM NADPH (entries 3 and 7 in **Supplementary Table 1**), or 0.5 mM NADH and 0.5 mM NADPH (entries 4 and 8 in **Supplementary Table 1**) or without cofactors (entries 1 and 5 in **Supplementary Table 1**), 1 mM $MgCl_2$ preincubated for 30 min at 30 °C. Then, a ketone **1a** (10 mM final concentration) and 2-propanol (50 µL, 10% v/v) were added to the mixture. Biotransformations were conducted in a final volume of 500 µL in glass vials (V = 1.5 mL) without air access for 24 h at 30 °C using a laboratory shaker (250 rpm). After this time, each reaction was stopped by extracting the content of the vial with EtOAc (3 × 1 mL), the combined organic phase was dried over anhydrous $MgSO_4$, and the filtrate was transferred into a separate HPLC vial and concentrated under a vacuum. The oil residue in one of the vials was used to determine % conv. by using GC analysis after derivatization of the crude mixture with BSA (see protocol ***3.2.2.***), and the other oil residue (ca. 2 mg) was redissolved in HPLC-grade 2-PrOH (250 µL), passed through a short pad of silica gel loaded into a Pasteur pipette (in order to remove residuals of the cell components), and analyzed directly by HPLC on a chiral stationary phase to establish

enantiomeric excesses (% ee) of optically active alcohol (*R*)-**2a**. For additional data, see also **Supplementary Table 1**.

Method B (Effect of the substrate concentration):

E. coli/Lk-ADH Prince (10 mg) was suspended in 50 mM Tris–HCl buffer (450 μL, pH 7.5) containing 1.0 mM $MgCl_2$ and preincubated for 30 min at 30 °C. Then, a ketone **1a** (25 mM, 50 mM, 75 mM, or 100 mM final concentration) and 2-propanol (50 μL, 10% v/v) were added to the mixture. Biotransformations were conducted in a final volume of 500 μL in glass vials (*V* = 1.5 mL) without air access for 24 h at 30 °C using a laboratory shaker (250 rpm). After this time, each reaction was stopped by extracting the content of the vial with EtOAc (3 × 1 mL), the combined organic phase was dried over anhydrous $MgSO_4$, and the filtrate was transferred into a separate HPLC vial and concentrated under a vacuum. The oil residue in one of the vials was used to determine % conv. by using GC analysis after derivatization of the crude mixture with BSA (see protocol ***3.2.2.***), and the other oil residue (ca. 2 mg) was redissolved in HPLC-grade 2-PrOH (250 μL), passed through a short pad of silica gel loaded into a Pasteur pipette (in order to remove residuals of the cell components), and analyzed directly by HPLC on a chiral stationary phase to establish enantiomeric excesses of optically active alcohol (*R*)-**2a**. For additional data, see also **Supplementary Table 2**.

Method C (Effect of the 2-PrOH concentration):

E. coli/Lk-ADH Prince (10 mg) was suspended in 50 mM Tris–HCl buffer (pH 7.5) containing 1.0 mM $MgCl_2$ and preincubated for 30 min at 30 °C. Then, a solution of ketone **1a** (100 mM final concentration) in 2-propanol (50–250 μL, 10–50% v/v) was added to the mixture. Biotransformations were conducted in a final volume of 500 μL in glass vials (*V* = 1.5 mL) without air access for 24 h at 30 °C using a laboratory shaker (250 rpm). After this time, each reaction was stopped by extracting the content of the vial with EtOAc (3 × 1 mL), the combined organic phase was dried over anhydrous $MgSO_4$, and the filtrate was transferred into a separate HPLC vial and concentrated under a vacuum. The oil residue in one of the vials was used to determine % conv. by using GC analysis after derivatization of the crude mixture with BSA (see protocol ***3.2.2.***), and the other oil residue (ca. 2 mg) was redissolved in HPLC-grade 2-PrOH (250 μL), passed through a short pad of silica gel loaded into a Pasteur pipette (in order to remove residuals of the cell components), and analyzed directly by HPLC on a chiral stationary phase to establish enantiomeric excesses of optically active alcohol (*R*)-**2a**. For additional data, see also **Supplementary Table 3**.

Method D (Effect of the amount of E. coli/Lk-ADH Prince cells):

E. coli/Lk-ADH Prince (20–60 mg) was suspended in 50 mM Tris–HCl buffer (3.6 mL; pH 7.5) containing 1.0 mM $MgCl_2$ and preincubated for 30 min at 30 °C. Then, a ketone **1a** (111 mg, 0.40 mmol, 100 mM final concentration) and 2-propanol (400 μL, 10% v/v) were added to the mixture. The reaction was shaken (250 rpm) at 30 °C for 24 h and then stopped by extraction with EtOAc (3 × 15 mL). The organic layers were combined and dried over anhydrous $MgSO_4$. After filtering off the drying agent and evaporating the volatiles, the crude residue was purified by column chromatography on SiO_2 gel using a mixture of $CHCl_3$/MeOH (95:5, v/v) as an eluent yielding desired optically active alcohol (*R*)-**2a**. For additional data, see also **Supplementary Table 4**.

3.2.2. Derivatization of the samples for GC analyses with BSA as silylation reagent

To a vial containing oil residue after enzymatic reactions, a solution of *N,O*-bis(trimethylsilyl)acetamide (BSA, 15 mg, 71.3 μmol, 18 μL) in CH_2Cl_2 (100 μL) was added in one portion. After 20 min of vigorous vortexing of the reaction mixture at room temperature, the aliquot of the sample was directly analyzed using GC.

3.2.3. General procedure for the analytical scale bioreductions of prochiral carbonyl substrates 1a–ah (except 1m, 1p, 1q, and 1w) using E. coli/Lk-ADH Prince – Substrate scope

Method E: *E. coli*/Lk-ADH Prince (10 mg) was suspended in 50 mM Tris–HCl buffer (pH 7.5) containing 1.0 mM $MgCl_2$ and preincubated for 30 min at 30 °C. Then, the respective ketone **1a**–**ah** (10 mM, final concentration) and 2-propanol (50 μL, 10% v/v) were added to the mixture. Biotransformations were conducted in a final volume of 500 μL in glass vials (V = 1.5 mL) without air access for 24 h at 30 °C using a laboratory shaker (250 rpm). After this time, each reaction was stopped by extracting the content of the vial with EtOAc (3 × 1 mL), the combined organic phase was dried over anhydrous $MgSO_4$, and the filtrate was transferred into a separate HPLC vial and concentrated under a vacuum. The oil residue in one of the vials was used to determine % conv. by using GC analysis (derivatization of the crude mixture with BSA if required, see protocol ***3.2.2.***), and the other oil residue (ca. 2 mg) was redissolved in HPLC-grade 2-PrOH (250 μL), passed through a short pad of silica gel loaded into a Pasteur pipette (in order to remove residuals of the cell components), and analyzed directly by HPLC on a chiral stationary phase to establish enantiomeric excesses of optically active alcohols *non-rac*-**1a**–**ah** [except (*S*)-**1m**, (*R*)-**1p**, (*R*)-**1q**, and (*R*)-**1w**]. For additional data, see **Scheme 1** in the main manuscript.

Method F (For products' not-extractable' with EtOAc): *E. coli*/Lk-ADH Prince (10 mg) was suspended in 50 mM Tris–HCl buffer (pH 7.5) containing 1.0 mM $MgCl_2$ and preincubated for 30 min at 30 °C. Then, the respective ketone **1m**, **1p**, or **1q** (10 mM, final concentration), and 2-propanol (50 μL, 10% v/v) were added to the mixture. Biotransformations were conducted in a final volume of 500 μL in glass vials (*V* = 1.5 mL) without air access for 24 h at 30 °C using a laboratory shaker (250 rpm). After this time, each reaction was stopped by extracting the content of the vial with *n*-hexane (3 × 1 mL), the combined organic phase was dried over anhydrous $MgSO_4$, the filtrate was transferred into a vial and filtrated by syringe filter and analyzed directly by GC and HPLC on a chiral stationary phase to establish enantiomeric excesses of optically active alcohols (*S*)-**2m**, (*R*)-**2p**, or (*R*)-**2q**. For additional data, see **Scheme 1** in the main manuscript.

Method G (For the product strongly soluble in H_2O): *E. coli*/Lk-ADH Prince (10 mg) was suspended in 50 mM Tris–HCl buffer (pH 7.5) containing 1.0 mM $MgCl_2$ and preincubated for 30 min at 30 °C. Then, a ketone **1w** (10 mM, final concentration) and 2-propanol (50 μL, 10% v/v) were added to the mixture. Biotransformations were conducted in a final volume of 500 μL in glass vials (*V* = 1.5 mL) without air access for 24 h at 30 °C using a laboratory shaker (250 rpm). After incubation, the enzymatic reaction was stopped by filtering off the cells using a syringe equipped with a hydrophobic PTFE filter (0.45 μM). Next, a portion of $PhCH_3$ (5 mL) was added to the permeate, and the water was azeotropically evaporated using a rotavap. The crude oil residue was diluted with HPLC-grade 2-PrOH and additionally passed through short-pad column chromatography (*vide* Pasteur pipette terminated with cotton wool and filled with SiO_2 gel) using HPLC-grade 2-PrOH (100%) as eluent, thus obtaining desired optically active alcohol (*R*)-**2w**. For additional data, see **Scheme 1** in the main manuscript.

Method H (Effect of the organic solvent):

E. coli/Lk-ADH Prince (10 mg) was suspended in 50 mM Tris–HCl buffer (300 μL, pH 7.5) containing 1.0 mM $MgCl_2$ and preincubated for 30 min at 30 °C. Then, the respective ketone **1g** or **1af** (10 mM, final concentration), 2-propanol (50 μL, 10% v/v), and organic solvent (i.e., DMSO, CH_3CN, TBME, 2-MeTHF or $PhCH_3$, 100 μL, 20% v/v) were added to the mixture. Biotransformations were conducted in a final volume of 500 μL in glass vials (*V* = 1.5 mL) without air access for 24 h at 30 °C using a laboratory shaker (250 rpm). After this time, each reaction was stopped by extracting the content of the vial with EtOAc (3 × 1 mL). The combined organic phase was dried over anhydrous $MgSO_4$, and the filtrate was transferred into

a separate HPLC vial and concentrated under a vacuum. The oil residue in one of the vials was used to determine % conv. by using GC analysis, and the other oil residue (ca. 2 mg) was redissolved in HPLC-grade 2-PrOH (250 μL), passed through a short pad of silica gel loaded into a Pasteur pipette (in order to remove residuals of the cell components), and analyzed directly by HPLC on a chiral stationary phase to establish enantiomeric excesses of optically active alcohols (*R*)-**2g** or (*S*)-**2af**. For additional data, see also **Supplementary Tables 5–6**.

Method I (Effect of the substrate concentration):

E. coli/Lk-ADH Prince (10 mg) was suspended in 50 mM Tris–HCl buffer (300 μL, pH 7.5) containing 1.0 mM $MgCl_2$ and preincubated for 30 min at 30 °C. Then, the respective ketone **1g** or **1af** (25 mM, 50 mM, 75 mM, or 100 mM final concentration), 2-propanol (50 μL, 10% v/v), and DMSO (100 μL, 20% v/v) were added to the mixture. Biotransformations were conducted in a final volume of 500 μL in glass vials (V = 1.5 mL) without air access for 24 h at 30 °C using a laboratory shaker (250 rpm). After this time, each reaction was stopped by extracting the content of the vial with EtOAc (3 × 1 mL), the combined organic phase was dried over anhydrous $MgSO_4$, and the filtrate was transferred into a separate HPLC vial and concentrated under a vacuum. The oil residue in one of the vials was used to determine % conv. by using GC analysis, and the other oil residue (ca. 2 mg) was redissolved in HPLC-grade 2-PrOH (250 μL), passed through a short pad of silica gel loaded into a Pasteur pipette (in order to remove residuals of the cell components), and analyzed directly by HPLC on a chiral stationary phase to establish enantiomeric excesses of optically active alcohols (*R*)-**2g** or (*S*)-**2af**. For additional data, see also **Supplementary Tables 7–8**.

3.2.3. General procedure for the semi-preparative scale bioreductions of prochiral carbonyl substrates 1a, 1d, 1g, 1i, 1j, 1l, 1o, 1r, 1u, 1w, 1v, 1x, 1y, 1z, 1aa, and 1af using E. coli/Lk-ADH Prince

Method J (0.40 mmol-scale): *E. coli*/Lk-ADH Prince (60 mg) was suspended in 50 mM Tris–HCl buffer (2.4–3.6 mL, pH 7.5) containing 1.0 mM $MgCl_2$ and preincubated for 30 min at 30 °C. Then, the respective ketone **1a**, **1d**, **1i**, **1j**, **1o**, **1r**, **1u**, **1z**, or **1aa** (0.40 mmol, 100 mM final concentration), 2-propanol (400 μL, 10% v/v) and DMSO (0–20% v/v) were added to the mixture. The reaction was shaken (250 rpm) at 30 °C for 24 h and then stopped by extraction with EtOAc (3 × 15 mL). The organic layers were combined and dried over anhydrous $MgSO_4$. After filtering off the drying agent and evaporating the volatiles, the crude residue was purified by column chromatography on SiO_2 gel using a mixture of the appropriate eluent (see below in

section ***3***) for each derivative to afford the desired optically active products: (*R*)-**2a** (96 mg, 86% yield, 99% ee); (*R*)-**2d** (35 mg, 51% yield, 99% ee); (*R*)-**2i** (75 mg, 73% yield, >99% ee); (*R*)-**2j** (34 mg, 62% yield, 93% ee); (*R*)-**2o** (30 mg, 41% yield, 88% ee); (*R*)-**2r** (39 mg, 79% yield, 96% ee); (*R*)-**2u** (62 mg, 76% yield, >99% ee); (*S*)-**2z** (45 mg, 68% yield, 94% ee); (*S*)-**2aa** (48 mg, 67% yield, >99% ee). For details, see **Table 2** in the main manuscript.

Method K (0.10 mmol-scale): *E. coli*/Lk-ADH Prince (60 mg) was suspended in 50 mM Tris–HCl buffer (2.4–3.6 mL, pH 7.5) containing 1.0 mM $MgCl_2$ and preincubated for 30 min at 30 °C. Then, the respective ketone **1g**, **1l**, **1v**, **1x**, **1y**, or **1af** (0.10 mmol, 25 mM final concentration), 2-propanol (400 µL, 10% v/v), and DMSO (0–20% v/v) were added to the mixture. The reaction was shaken (250 rpm) at 30 °C for 24 h and then stopped by extraction with EtOAc (3 × 15 mL). The organic layers were combined and dried over anhydrous $MgSO_4$. After filtering off the drying agent and evaporating the volatiles, the crude residue was purified by column chromatography on SiO_2 gel using a mixture of the appropriate eluent (see below in section ***3***) for each derivative to afford the desired optically active products: (*R*)-**2g** (13 mg, 66% yield, >99% ee); (*R*)-**2l** (10 mg, 67% yield, 98% ee); (*R*)-**2v** (16 mg, 67% yield, >99% ee); (*S*)-**2x** (9 mg, 38% yield, 95% ee); (*S*)-**2y** (16 mg, 59% yield, 99% ee); (*S*)-**2af** (8 mg, 40% yield, >99% ee). For details, see **Table 2** in the main manuscript.

Method L (0.40 mmol-scale with modified work up for the product strongly soluble in H_2O): *E. coli*/Lk-ADH Prince (60 mg) was suspended in 50 mM Tris–HCl buffer (3.6 mL, pH 7.5) containing 1.0 mM $MgCl_2$ and preincubated for 30 min at 30 °C. Then, a ketone **1w** (0.40 mmol, 100 mM final concentration) and 2-propanol (400 µL, 10% v/v) were added to the mixture. The reaction was shaken (250 rpm) at 30 °C for 24 h and then stopped by azeotropic evaporation of H_2O with $PhCH_3$ (25 ml). The crude residue was dissolved in CH_2Cl_2 and dried over anhydrous $MgSO_4$. After filtering off the drying agent and evaporating the volatiles, the crude residue was purified by column chromatography on SiO_2 gel using a mixture of CH_2Cl_2/MeOH (95:5, v/v) as an eluent to afford the desired optically active (*R*)-**2w** (77 mg, 91% yield, >99% ee). For details, see **Table 2** in the main manuscript.

4. Supplementary Methods 3. Optimization of the reaction parameters.

Supplementary Table 1. Screening conditions for the *E. coli*/Lk-ADH Prince-catalyzed asymmetric bioreduction of the model pentoxifylline (**1a**) after 24 h.

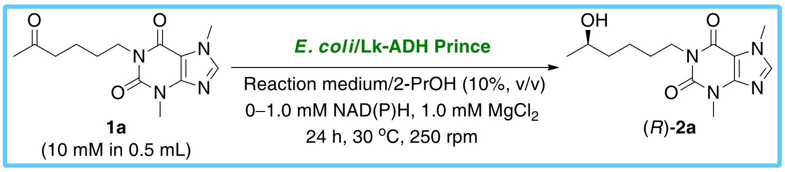

Entry	Reaction medium[a]	NADH [mM]	NADPH [mM]	Conv.[b] [%]	ee_p[c] [%]
1	50 mM Tris-HCl buffer (pH 7.5)	–	–	99	99
2		1.0	–	98	99
3		–	1.0	98	99
4		1.0	1.0	98	99
5	Distilled H_2O	–	–	99	98
6		1.0	–	98	99
7		–	1.0	98	99
8		1.0	1.0	98	99

[a] Reaction conditions: **1a** (10 mM final conc.), lyophilized biocatalyst (10 mg), 1.0 mM NADH (or without), 1.0 mM NADPH (or without), 1.0 mM $MgCl_2$, reaction medium/2-PrOH (500 μL; 90:10, v/v), 24 h, 30 °C, 250 rpm (laboratory shaker).
[b] Conversion values (%) (i.e., consumption of substrate **1a**) were determined by GC analyses after derivatization of crude mixture with *N*,*O*-bis(trimethylsilyl)acetamide (BSA) as a silylating reagent.
[c] Determined for (*R*)-**2a** by chiral HPLC analysis.

Supplementary Table 2. *E. coli*/Lk-ADH Prince-catalyzed bioreduction of pentoxifylline (**1a**) after 24 h – effect of the substrate concentration.

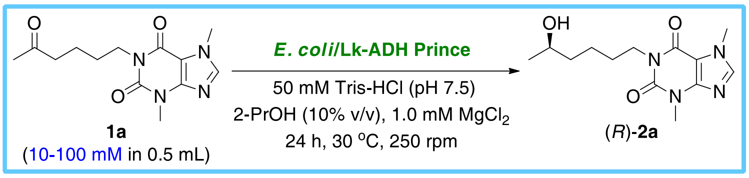

Entry	Substrate conc.[a] [mM]	Conv.[b] [%]	ee_p[c] [%]
1	10	99	99
2	25	98	99
3	50	96	99
4	75	94	99
5	100	91	99

[a] Reaction conditions: **1a** (10–100 mM final conc.), lyophilized biocatalyst (10 mg), 1.0 mM $MgCl_2$, 50 mM Tris-HCl (pH 7.5)/2-PrOH (500 μL; 90:10, v/v), 24 h, 30 °C, 250 rpm.
[b] Conversion values (%) were determined by GC analyses after derivatization of crude mixture with BSA as a silylating reagent.
[c] Determined for (*R*)-**2a** by chiral HPLC analysis.

Supplementary Table 3. *E. coli*/Lk-ADH Prince-catalyzed bioreduction of pentoxifylline (**1a**) after 24 h – effect of the 2-PrOH concentration.

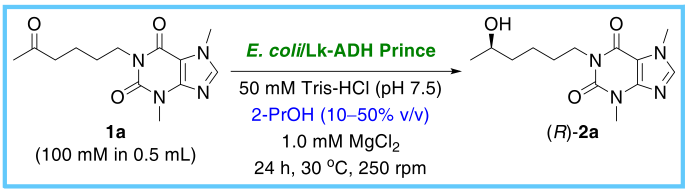

Entry	2-PrOH[a] [%, v/v]	Conv.[b] [%]	ee_p[c] [%]
1	10	90	99
2	20	78	99
3	30	71	99
4	40	41	>99
5	50	31	>99

[a] Reaction conditions: **1a** (100 mM final conc.), lyophilized biocatalyst (10 mg), 1.0 mM $MgCl_2$, 50 mM Tris-HCl (pH 7.5), 2-PrOH (10–50%, v/v), 0.5 final volume, 24 h, 30 ºC, 250 rpm.
[b] Conversion values (%) were determined by GC analyses after derivatization of crude mixture with BSA as a silylating reagent.
[c] Determined for (*R*)-**2a** by chiral HPLC analysis.

Supplementary Table 4. Preparative-scale *E. coli*/Lk-ADH Prince-catalyzed bioreduction of pentoxifylline (**1a**) after 24 h – effect of the amount of *E. coli*/Lk-ADH Prince cells.

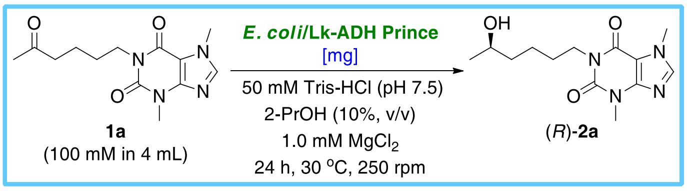

Entry	*E. coli* cells [mg][a]	*E. coli* cells [mg/mL]	Conv.[b] [%]	Yield[c] [%]	ee_p[d] [%]
1	20	5	64	53	99
2	40	10	83	71	99
3	60	15	91	85	99

[a] Reaction conditions: **1a** (111 mg, 0.4 mmol, 100 mM final conc.), lyophilized whole-cell biocatalyst (20–60 mg), 1.0 mM $MgCl_2$, 50 mM Tris-HCl (pH 7.5)/2-PrOH (4 mL; 90:10, v/v), 24 h, 30 ºC, 250 rpm.
[b] Conversion values (%) were determined by GC analyses after derivatization of crude mixture with BSA as a silylating reagent.
[c] Isolated yield after column chromatography.
[d] Determined for (*R*)-**2a** by chiral HPLC analysis.

Supplementary Table 5. *E. coli*/Lk-ADH Prince-catalyzed bioreduction of 1-(biphenyl-4-yl)ethanone (**1g**) after 24 h – effect of the co-solvent.

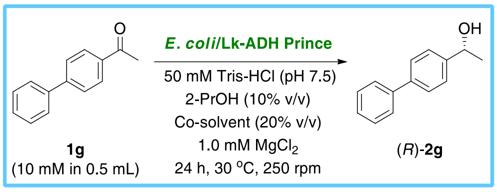

Entry	Co-solvent[a]	Conv.[b] [%]	ee_p[c] [%]
1	–	84	>99
2	DMSO	90	>99
3	CH_3CN	92	>99
4	TBME	75	>99
5	2-MeTHF	63	>99
6	$PhCH_3$	20	>99

[a] Reaction conditions: **1g** (10 mM final conc.), lyophilized biocatalyst (10 mg), 1.0 mM $MgCl_2$, 50 mM Tris-HCl (pH 7.5)/2-PrOH/co-solvent (500 µL; 70:10:20, v/v/v), 24 h, 30 ºC, 250 rpm.
[b] Conversion values (%) were determined by GC analyses using calibration curve.
[c] Determined for (*R*)-**2g** by chiral HPLC analysis.

Supplementary Table 6. *E. coli*/Lk-ADH Prince-catalyzed bioreduction of 1,2-diphenylethanone (**1af**) after 24 h – effect of the co-solvent.

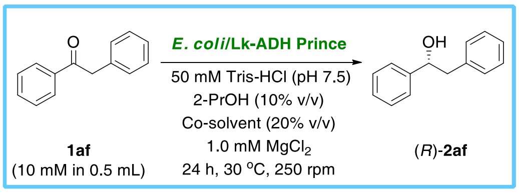

Entry	Co-solvent[a]	Conv.[b] [%]	ee_p[c] [%]
1	–	91	>99
2	DMSO	92	>99
3	CH_3CN	63	>99
4	TBME	6	–
5	2-MeTHF	4	–
6	$PhCH_3$	4	–

[a] Reaction conditions: **1af** (10 mM final conc.), lyophilized biocatalyst (10 mg), 1.0 mM $MgCl_2$, 50 mM Tris-HCl (pH 7.5)/2-PrOH/co-solvent (500 µL; 70:10:20, v/v/v), 24 h, 30 ºC, 250 rpm.
[b] Conversion values (%) were determined by GC analyses using calibration curve.
[c] Determined for (*S*)-**2af** by chiral HPLC analysis.

Supplementary Table 7. *E. coli*/Lk-ADH Prince-catalyzed bioreduction of 1-(biphenyl-4-yl)ethanone (**1g**) after 24 h – effect of the substrate concentration.

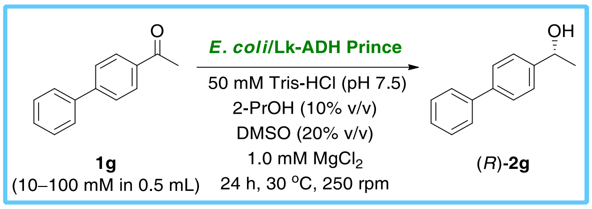

Entry	Substrate conc.[a] [mM]	Conv.[b] [%]	ee_p[c] [%]
1	10	90	>99
2	25	74	>99
3	50	44	–
4	75	24	–
5	100	21	–

[a] Reaction conditions: **1g** (10–100 mM final conc.), lyophilized biocatalyst (10 mg), 1.0 mM $MgCl_2$, 50 mM Tris-HCl (pH 7.5)/2-PrOH/DMSO (500 µL; 70:10:20, v/v/v), 24 h, 30 ºC, 250 rpm.
[b] Conversion values (%) were determined by GC analyses using calibration curve.
[c] Determined for (*R*)-**2g** by chiral HPLC analysis.

Supplementary Table 8. *E. coli*/Lk-ADH Prince-catalyzed bioreduction of pentoxifylline (**1af**) after 24 h – effect of the substrate concentration.

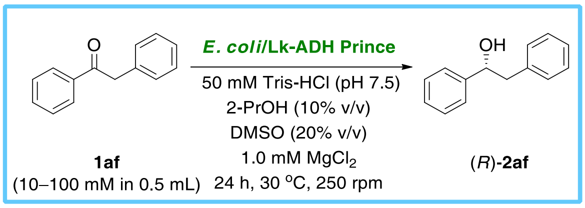

Entry	Substrate conc.[a] [mM]	Conv.[b] [%]	ee_p[c] [%]
1	10	92	>99
2	25	85	>99
3	50	43	–
4	75	24	–
5	100	16	–

[a] Reaction conditions: **1af** (10–100 mM final conc.), lyophilized biocatalyst (10 mg), 1.0 mM $MgCl_2$, 50 mM Tris-HCl (pH 7.5)/2-PrOH/DMSO (500 µL; 70:10:20, v/v/v), 24 h, 30 ºC, 250 rpm.
[b] Conversion values (%) were determined by GC analyses using calibration curve.
[c] Determined for (*S*)-**2af** by chiral HPLC analysis.

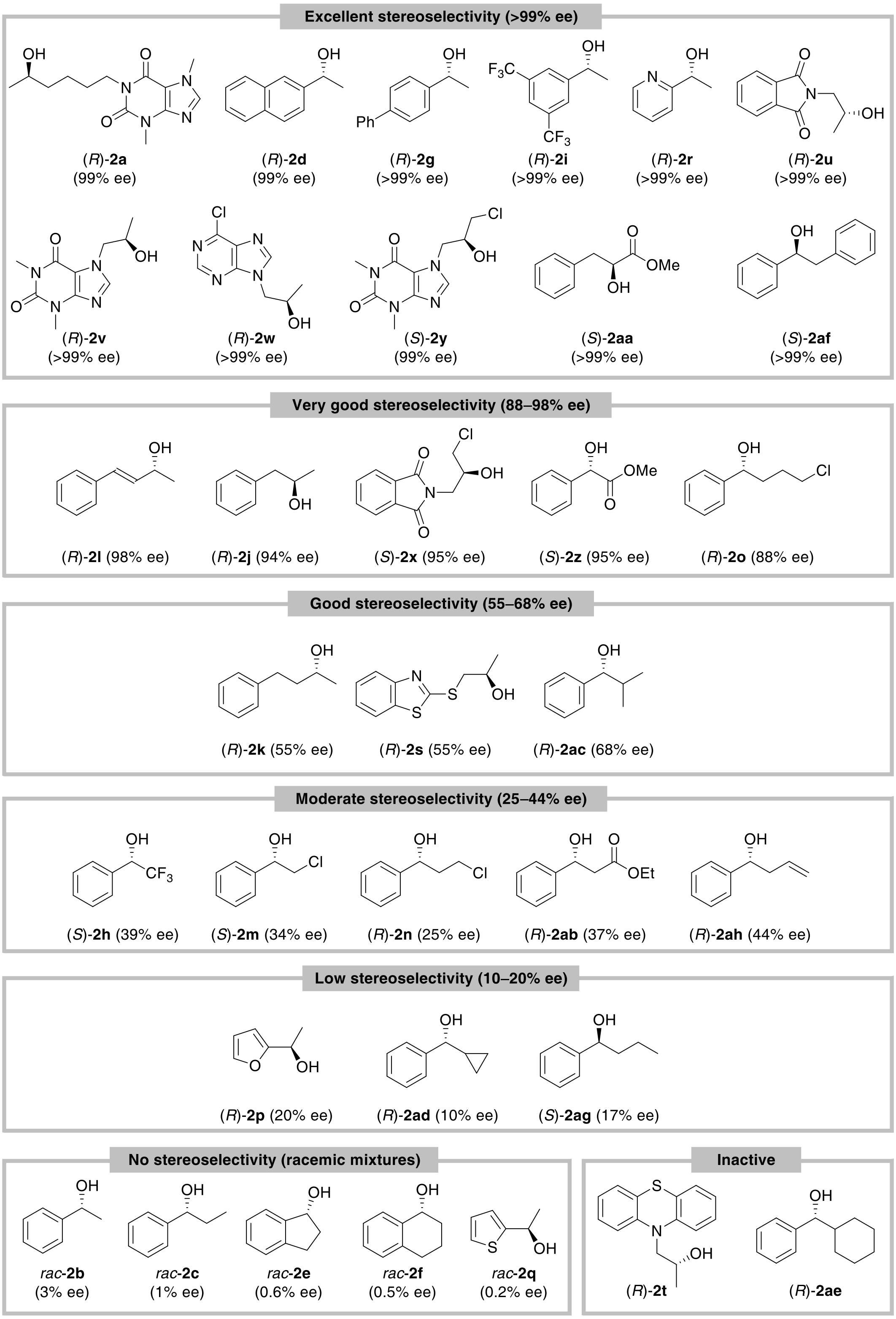

Supplementary Figure 1. Grouping optical purity spectrum for the tested *E. coli*/Lk-ADH Prince toward carbonyl substrates.

5. Supplementary Methods 4. Molecular docking.

5.1. Molecular docking preparation

Molecular docking simulations to establish favorable ligand binding geometry for 1-(6-chloro-9*H*-purin-9-yl)propan-2-one (**1w**) in Lk-ADH Prince were carried out on a 24 CPUs-based desktop PC computer equipped with AMD Ryzen™ 9 3900X 12-Core Processor 3800 MHz and 32 GB of RAM on a Microsoft Windows 11 Professional 64-bit operating system using AutoDock Vina vs. 1.1.2 program for Windows (http://autodock.scripps.edu/) [1].

5.1.1 Ligand preparation

At first, the respective ligand molecule **1w** in non-ionizable form was prepared with ChemAxon MarvinSketch vs. 14.9.1.0 (http://www.chemaxon.com/marvin/) using the general 'Cleaning in 3D' option to assign with proper 3D orientation and then calculating conformers with MMFF94 force field parameters and saved as .pdb file. To obtain the minimum energy conformation for docking studies, the initial geometries of the afore-pretreated ligand were additionally optimized in Avogadro vs. 1.2.0. (http://avogadro.cc/) using MMFF94 force field with 500 steps and Steepest Descent Algorithm, after adding all the hydrogens to the structure, and saved as .mol2 files. Next, the Gasteiger partial charges were calculated with AutoDock Tools vs. 1.5.6 (ADT, S3 http://mgltools.scripps.edu/). In contrast, all torsion angles for **1w** were considered flexible, and all the possible rotatable bonds and non-polar hydrogens were determined (the number of rotatable bonds found in **1w** were 2 out of 32). The final ligand **1w** file was saved as .pdbqt.

5.1.2 Receptor preparation

Lk-ADH Prince (Lk-ADH-E145F-F147L-Y190C) triple mutant was prepared using the crystal structure of alcohol dehydrogenase Lk-ADH from *Lactobacillus kefir* (PDB code: 4RF2) [2] downloaded from Brookhaven RCSB Protein Data Bank (PDB database, http://www.rcsb.org/pdb/). To avoid steric clashes within the model, the crude target protein .pdb file 4RF2 was prepared by UCSF Chimera vs. 1.11.2 package (http://www.cgl.ucsf.edu/chimera/) [3] by removing all crystal waters (HOH). Nicotinamide-adenine-dinucleotide phosphate (NADP+, denoted as NAP) was left to determine the relevant interactions between ligand-receptor. Next, triple mutation (Phe instead of Glu145; Leu instead of Phe147; Cys instead of Tyr190) was performed using standard UCSF Chimera tools (i.e., tools → structure editing → rotamers) set on dynameomics backbone-independent rotamer

library according to A.D. Scouras and V. Dagget [4]. Afterward, the polar hydrogen atoms were added, and Gasteiger charges were calculated with AutoDock Tools 1.5.6 package using its standard utility scripts, and then the final protein file was saved as .pdbqt. A searching 'grid box' was set by using the AutoGrid function to perform docking in a (10 × 10 × 10 Å)-unit grid box centered on a catalytic cavity located in the Lk-ADH Prince (center_x = 25.365; center_y = −31.938; center_z = 29.345) as target coordinates with a grid spacing of 0.325 Å, respectively.

5.2. Molecular docking procedure

Docking was performed with an exhaustiveness level of 96 concerning global search. Number of 100 independent runs were performed for ligand molecule **1w** using the Lamarckian Genetic Algorithm (GA) with at most 106 energy evaluations and a maximum number of generations of >27 000 $Å^3$ (the search space volume). The rest of the docking parameters, including the remaining Lamarckian GA parameters, were set as default using the standard values for genetic Vina algorithms (the posed dockings were below 5.00 Å rmsd). The docking modes of the studied ligand **1w** were clustered and ranked based on a mutual ligand–protein affinity expressed as absolute free binding energies [ΔG_{calc} (kcal/mol)] as well as the values of root mean square deviation (rmsd) in both modes regarding rmsd lower bound (l.b.), and rmsd upper bound (u.b.), respectively. The rmsd-values were computed referring to the input structure submitted to docking simulations. The used random seed amounted to –657606464. The best nine poses (modes) were selected according to AutoDock Vina scoring functions based on binding energies showing mutual ligand–protein affinity (kcal/mol). The results generated by AutoDock Vina, including optimized binding poses of **1w** in hypothetical complexes with Lk-ADH Prince and critical polar contacts between the respective atoms of the ligand and receptor molecules, were visualized using The PyMOL Molecular Graphics System software, vs. 1.3, Schrödinger, LLC (https://www.pymol.org/) (**Figure 6A−C** in main text). Two-dimensional (2D) visualization of the Lk-ADH Prince binding interfaces for top-scoring pose of the ligand **1w** molecule was computed by BIOVIA Discovery Studio Visualizer 20.1.0.19295 software (**Figure 6D** in main text).

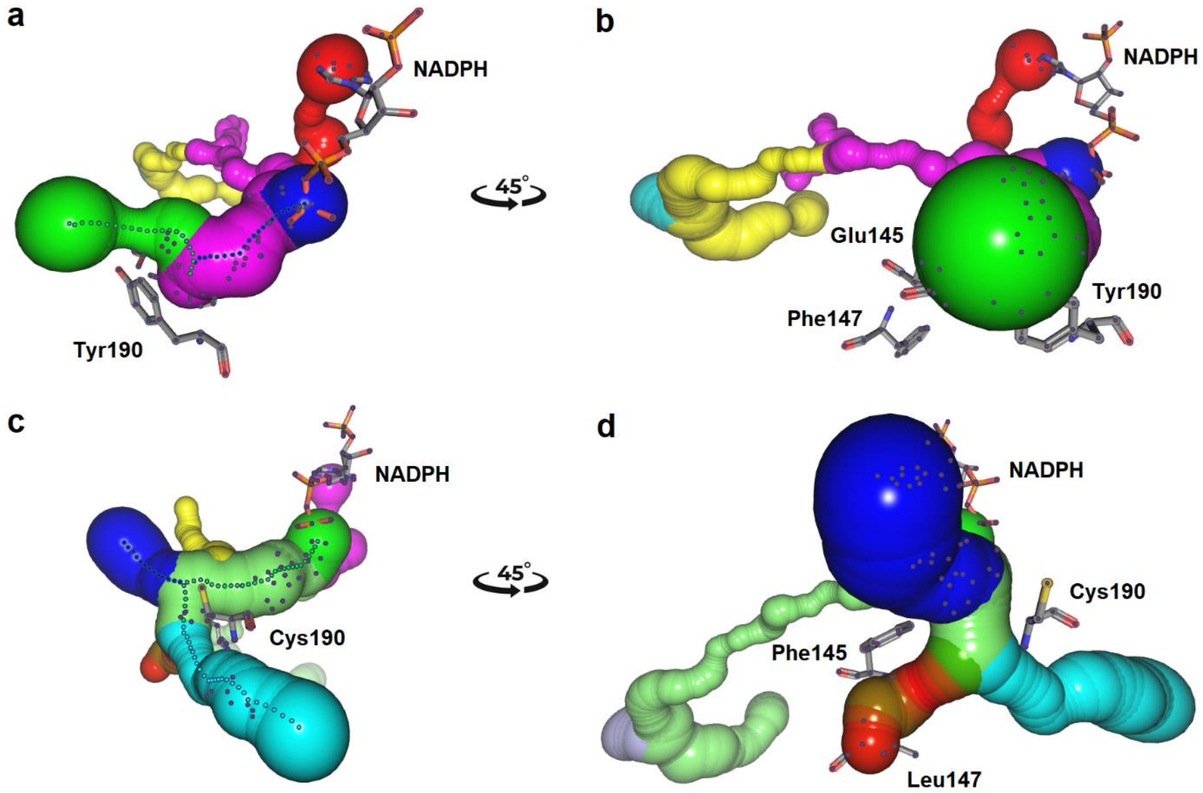

Supplementary Figure 2. Visualization of the tunnels in wild-type Lk-ADH (panel a and b) and Lk-ADH Prince (panel c and d) represented as a set of intersecting spheres using CAVER Analyst 2.0 software [5].

Supplementary Table 9. Tunnel statistics calculated with CAVER Analyst 2.0 software.

Protein	ID[a]	No[b]	No_snaps[c]	AvgBR [Å][d]	MaxBR [Å][e]	AvgL [Å][f]	AvgC[g]	Prior[h]	Avg_throug[i]
Lk-ADH	tun_cl_1	1	1	2.216	2.220	10.583	1.216	0.823	0.823
	tun_cl_2	1	1	1.280	1.280	10.157	1.221	0.723	0.723
Lk-ADH Prince	tun_cl_1	1	1	1.854	1.850	9.690	1.210	0.815	0.815
	tun_cl_2	1	1	1.854	1.850	16.032	1.309	0.731	0.731
	tun_cl_3	1	1	1.310	1.310	12.134	1.115	0.643	0.643

[a] Identification of a given tunnel cluster; ranks a given cluster based on their priority.

[b] Total number of tunnels belonging to a given cluster.

[c] Number of snapshots with at least one tunnel with a radius >= parameter min_probe_radius.

[d] Average bottleneck radius (i.e., the narrowest part, of a given tunnel).

[e] Maximum bottleneck radius.

[f] Average tunnel length.

[g] Average tunnel curvature.

[h] Tunnel priority calculated by averaging tunnel throughputs over all snapshots (zero value used for snapshots without tunnels).

[i] Average tunnel throughput (where throughput = e^{-cost}).

Supplementary Table 10. Chiral building blocks (obtained in this work) useful for the synthesis of APIs and/or natural products.

Building block	APIs	Natural products
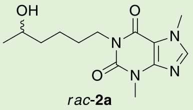 *rac*-**2a**	lisofylline	-
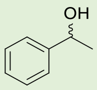 *rac*-**2b**	crizotinib, mavacamten, etomidate, fendiline	trail pheromone of *Aphaenogaster cockerelli*
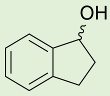 *rac*-**2e**	rasagiline	-
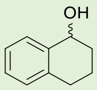 *rac*-**2f**	sertraline	-
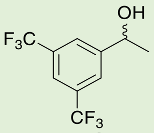 *rac*-**2i**	aprepitant	-
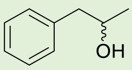 *rac*-**2j**	selegiline, dextroamphetamine and amphetamine prodrugs (i.e., amfetaminil, fenethylline, etc.)	-
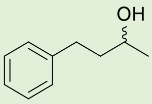 *rac*-**2k**	labetalol, emepronium	-
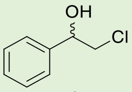 *rac*-**2m**	apremilast, nifenalol, satolol, medroxalol, salmeterol, denopamine, tembamide, aegeline, isoproterenol	-
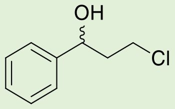 *rac*-**2n**	fluoxetine, dapoxetine, nisoxetine, atomoxetine, levamisole, tolterodine	-
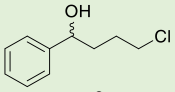 *rac*-**2o**	sertraline, terfenadine, fexofenadine, trecetilide, flutroline	(+)-cryptophycin A, (+)-cryptophycin 52, (–)-centrolobine

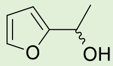 *rac*-**2p**	-	(−)-cassine, (−)-spectaline, (−)-carnavaline, prosafrine, (−)-prosafrinine
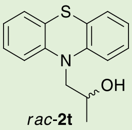 *rac*-**2t**	ethopropazine (profenamine), promethazine	-
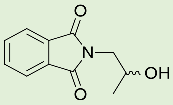 *rac*-**2u**	tenofovir, dextromoramide	-
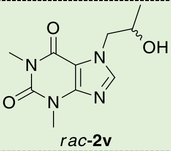 *rac*-**2v**	proxyphylline	-
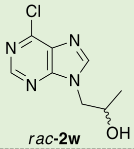 *rac*-**2w**	tenofovir	-
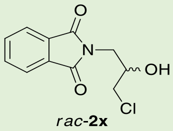 *rac*-**2x**	rivaroxaban, β-blockers (i.e., propranolol, alprenolol, pindolol, carazolol, moprolol, metoprolol, etc.)	-
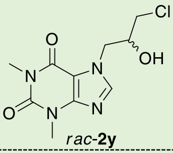 *rac*-**2y**	xanthinol, diprophylline	-
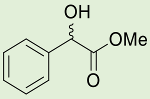 *rac*-**2z**	pemoline, eflucimibe, cyclandelate, homotropine, clopidogrel, amoxicillin	-
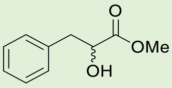 *rac*-**2aa**	saroglitazar, AZD 4619	danshensu, oscilarina, aeruginosin 298A
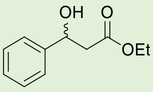 *rac*-**2ab**	fluoxetine, dapoxetine, nisoxetine, atomoxetine, levamisole, tolterodine	-
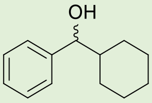 *rac*-**2ae**	oxybutynin	-
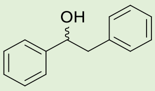 *rac*-**2af**	-	(*R*)-combrestatin

6. Supplementary physico-chemical data of the products.

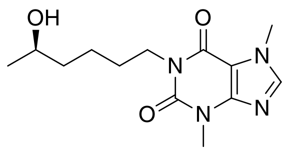

(*R*)-**2a**

R_f [$CHCl_3$/MeOH (95:5, v/v)] 0.37; ^1H NMR (500 MHz, $CDCl_3$) δ 7.49 (s, 1H), 4.00 (t, J = 7.5 Hz, 2H), 3.97 (s, 3H), 3.79 (dd, J = 12.0, 5.8 Hz, 1H), 3.56 (s, 3H), 1.80 (br. s, 1H), 1.73–1.61 (m, 3H), 1.56–1.35 (m, 4H), 1.17 (d, J = 6.2 Hz, 3H); $^{13}C\{^1H\}$ NMR (126 MHz, $CDCl_3$) δ 155.5, 151.6, 148.9, 141.5, 107.8, 68.0, 41.3, 38.9, 33.7, 29.8, 28.0, 23.6, 23.1; FTMS (ESI-TOF) *m/z*: $[M+H]^+$ Calcd for $C_{13}H_{21}N_4O_3^+$ m/z: 281.16082, Found 281.16079; ATR-FTIR (neat): ν_{max} = 3361 (br, $\nu_{O\text{-}H}$), 1695 ($\nu_{O\text{-}H}$), 1651 ($\delta_{N\text{-}H}$); GC [260 (10 °C/min)]: t_R = 9.47 min; HPLC [*n*-hexane-*i*-PrOH-DEA (78:22:0.1, v/v); f = 1.0 mL/min (p = 5.5 MPa); λ = 273 nm (Chiralpak AD-H)]: t_R = 31.082 min (*R*-isomer) and 34.118 min (*S*-isomer).

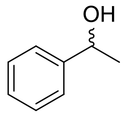

rac-**2b**

R_f [*n*-hexane/AcOEt (90:10, v/v)] 0.22; ^1H NMR (500 MHz, $CDCl_3$) δ 7.61–7.09 (m, 5H), 4.87 (q, J = 6.5 Hz, 1H), 2.29 (br. s, 1H), 1.49 (d, J = 6.5 Hz, 3H); $^{13}C\{^1H\}$ NMR (126 MHz, $CDCl_3$) δ 146.1, 128.7, 127.7, 125.7, 70.6, 25.4; FTMS (ESI-TOF) *m/z*: $[M+H]^+$ Calcd for $C_8H_{11}O^+$ m/z: 123.08044, Not Found; $[M+H–H_2O]^+$ Calcd for $C_8H_9^+$ m/z: 105.06988, Found 105.07040; ATR-FTIR (neat): ν_{max} = 3350 (br, $\nu_{O\text{-}H}$), 744 ($\gamma_{C\text{-}H}$), 697 ($\gamma_{C\text{-}H}$); GC [100–260 (10 °C/min)]: t_R = 2.71 min; HPLC [*n*-hexane-*i*-PrOH (97:3, v/v); f = 1.0 mL/min (p = 4.4 MPa); λ = 210 nm (Chiralcel OD-H)]: t_R = 10.839 min (*R*-isomer) and 12.638 min (*S*-isomer).

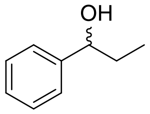

rac-**2c**

R_f [*n*-hexane/AcOEt (90:10, v/v)] 0.24; ^1H NMR (500 MHz, $CDCl_3$) δ 7.39–7.29 (m, 4H), 7.29–7.22 (m, 1H), 4.56 (dd, J = 8.6, 4.6 Hz, 1H), 2.12 (br. s, 1H), 1.87–1.68 (m, 2H), 0.90 (t, J = 7.4 Hz, 3H); $^{13}C\{^1H\}$ NMR (126 MHz, $CDCl_3$) δ 144.6, 128.4, 127.5, 126.0, 76.0, 31.9, 10.2; FTMS (ESI-TOF) *m/z*: $[M+H]^+$ Calcd for $C_9H_{13}O^+$ m/z: 137.09609, Found 137.05973; $[M+H–H_2O]^+$ Calcd for $C_9H_{11}^+$ m/z: 119.08553, Found Not Found; ATR-FTIR (neat): ν_{max} = 3387 (br, $\nu_{O\text{-}H}$), 1453 ($\nu_{C\text{-}O}$), 759 ($\gamma_{C\text{-}H}$), 699 ($\gamma_{C\text{-}H}$); GC [100–260 (10 °C/min)]: t_R = 3.50 min HPLC [*n*-hexane-*i*-PrOH (98:2, v/v); f = 1.0 mL/min (p = 4.4 MPa); λ = 220 nm (Chiralcel OD-H)]: t_R = 12.738 min (*R*-isomer) and 13.633 min (*S*-isomer).

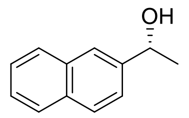

(*R*)-**2d**

R_f [*n*-hexane/AcOEt (90:10, v/v)] 0.20; ^{1}H NMR (500 MHz, $CDCl_3$) δ 7.92–7.72 (m, 4H), 7.56–7.42 (m, 3H), 5.05 (q, J = 6.5 Hz, 1H), 2.15 (br. s, 1H), 1.58 (d, J = 6.5 Hz, 1H); $^{13}C\{^1H\}$ NMR (126 MHz, $CDCl_3$) δ 143.3, 133.4, 133.0, 128.4, 128.0, 127.8, 126.2, 125.9, 123.9, 123.9, 70.6, 25.2; FTMS (ESI-TOF) *m/z*: $[M+H]^+$ Calcd for $C_{12}H_{13}O^+$ m/z: 173.09609, Not Found; $[M+H–H_2O]^+$ Calcd for $C_{12}H_{11}^+$ m/z: 155.08553, Found 155.08556; ATR-FTIR (neat): ν_{max} = 3291 (br, $\nu_{O\text{-}H}$), 1599 ($\gamma_{C\text{-}H}$), 1599 ($\gamma_{C\text{-}H}$), 1072 ($\nu_{C\text{-}O}$), 823 ($\gamma_{C\text{-}H}$), 740 ($\gamma_{C\text{-}H}$); GC [150–260 (10 °C/min)]: t_R = 5.28 min; HPLC [*n*-hexane-*i*-PrOH (90:10, v/v); f = 1.0 mL/min (p = 4.5 MPa); λ = 222 nm (Chiralcel OJ-H)]: t_R = 13.786 min (*S*-isomer) and 17.834 min (*R*-isomer).

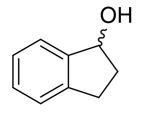

rac-**2e**

R_f [*n*-hexane/AcOEt (90:10, v/v)] 0.11; ^{1}H NMR (500 MHz, $CDCl_3$) δ 7.40 (d, J = 6.8 Hz, 1H), 7.33–7.17 (m, 3H), 5.30–5.11 (m, 1H), 3.07–2.84 (m, 1H), 2.90–2.73 (m, 1H), 2.58–2.37 (m, 2H), 2.01–1.84 (m, 1H); $^{13}C\{^1H\}$ NMR (126 MHz, $CDCl_3$) δ 145.1, 143.3, 128.3, 126.7, 124.9, 124.3, 76.3, 35.9, 29.8; FTMS (ESI-TOF) *m/z*: $[M+H]^+$ Calcd for $C_9H_{11}O^+$ m/z: 135.08044, Not Found; $[M+H–H_2O]^+$ Calcd for $C_9H_9^+$ m/z: 117.06988, Found 117.07030; ATR-FTIR (neat): ν_{max} = 3213 (br, $\nu_{O\text{-}H}$), 1456 ($\gamma_{C\text{-}H}$), 1326 ($\gamma_{C\text{-}H}$), 1055 ($\nu_{C\text{-}O}$), 762 ($\gamma_{C\text{-}H}$), 737 ($\gamma_{C\text{-}H}$); GC [150–260 (10 °C/min)]: t_R = 2.17 min; HPLC [*n*-hexane-*i*-PrOH (98:2, v/v); f = 1.0 mL/min (p = 4.4 MPa); λ = 205 nm (Chiralcel OD-H)]: t_R = 15.539 min (*S*-isomer) and 17.650 min (*R*-isomer).

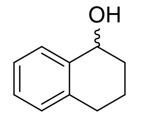

rac-**2f**

R_f [*n*-hexane/AcOEt (90:10, v/v)] 0.13; ^{1}H NMR (500 MHz, $CDCl_3$) δ 7.43 (dd, J = 5.7, 3.3 Hz, 1H), 7.31–7.16 (m, 2H), 7.15–7.04 (m, 1H), 4.86–4.70 (m, 1H), 2.93–2.63 (m, 2H), 2.07–1.86 (m, 3H), 1.86–1.73 (m, 2H); $^{13}C\{^1H\}$ NMR (126 MHz, $CDCl_3$) δ 138.9, 137.2, 129.1, 128.6, 127.7, 126.3, 68.3, 32.4, 29.4, 18.9; FTMS (ESI-TOF) *m/z*: $[M+H]^+$ Calcd for $C_{10}H_{13}O^+$ m/z: 149.09609, Not Found; $[M+H–H_2O]^+$ Calcd for $C_{10}H_{11}^+$ m/z: 131.08553, Found 131.08574; ATR-FTIR (neat): ν_{max} = 3274 (br, $\nu_{O\text{-}H}$), 1452 ($\gamma_{C\text{-}H}$), 1267 ($\gamma_{C\text{-}H}$), 1067 ($\nu_{C\text{-}O}$), 963 ($\gamma_{C\text{-}H}$), 776 ($\gamma_{C\text{-}H}$); GC [150–260 (10 °C/min)]: t_R = 3.03 min; HPLC [*n*-hexane-*i*-PrOH (90:10, v/v); f = 0.8 mL/min (p = 3.6 MPa); λ = 220 nm (Chiralcel OJ-H)]: t_R = 7.871 min (*S*-isomer) and 9.170 min (*R*-isomer).

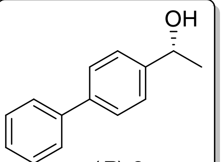

(*R*)-**2g**

R_f [*n*-hexane/AcOEt (30:10, v/v)] 0.38; ^{1}H NMR (500 MHz, $CDCl_3$) δ 7.64–7.55 (m, 4H), 7.49–7.41 (m, 4H), 7.39–7.32 (m, 1H), 4.96 (d, J = 6.5 Hz, 1H), 1.90 (s, 1H), 1.55 (d, J = 6.5 Hz, 3H); $^{13}C\{^1H\}$ NMR (126 MHz, $CDCl_3$) δ 145.0, 141.0, 140.6, 128.9, 127.4, 127.2, 126.0, 70.3, 25.30;

FTMS (ESI-TOF) *m/z*: $[M+H]^+$ Calcd for $C_{14}H_{15}O^+$ m/z: 199.11174, Not Found; $[M+H–H_2O]^+$ Calcd for $C_{14}H_{13}^+$ m/z: 181.10118, Found 181.10133; ATR-FTIR (neat): ν_{max} = 3302 (br, $\nu_{O\text{-}H}$), 1482 ($\gamma_{C\text{-}H}$), 1085 ($\gamma_{C\text{-}H}$), 1067 ($\nu_{C\text{-}O}$), 833 ($\gamma_{C\text{-}H}$), 759 ($\gamma_{C\text{-}H}$), 686 ($\gamma_{C\text{-}H}$); GC [170–260 (10 °C/min)]: t_R = 5.71 min; HPLC [*n*-hexane-*i*-PrOH (98:2, v/v); f = 0.8 mL/min (*p* = 3.5 MPa); λ = 210 nm (Chiralcel OD-H)]: t_R = 34.797 min (*S*-isomer) and 37.752 min (*R*-isomer).

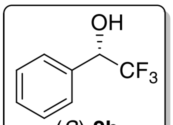

(*S*)-**2h**

R_f [*n*-hexane/AcOEt (90:10, v/v)] 0.27; ^{1}H NMR (500 MHz, $CDCl_3$) δ 7.51–7.45 (m, 2H), 7.44–7.40 (m, 3H), 5.03–4.99 (m, 1H), 2.84 (d, *J* = 4.5 Hz, 1H); ^{13}C{^{1}H} NMR (126 MHz, $CDCl_3$) δ 134.1, 129.7, 128.8, 127.8, 127.6, 124.4 (q, *J* = 282.4 Hz), 72.8 (q, *J* = 32.2 Hz); ^{19}F{^{1}H} NMR (470 MHz, $CDCl_3$) δ –83.6 (d, *J* = 6.7 Hz, 3F); FTMS (ESI-TOF) *m/z*: $[M+H]^+$ Calcd for $C_9H_{13}O^+$ m/z: 177.05217, Not Found; $[M+H–OH]^+$ Calcd for $C_8H_6F_3^+$ m/z: 159.04161, Found 159.04173; $[M–H]^–$ Calcd for $C_8H_6F_3O^–$ m/z: 175.03653, Found 175.03656; ATR-FTIR (neat): ν_{max} = 3406 (br, $\nu_{O\text{-}H}$), 1456 ($\gamma_{C\text{-}H}$), 1121 ($\gamma_{C\text{-}H}$), 1059 ($\nu_{C\text{-}O}$), 865 ($\gamma_{C\text{-}H}$), 759 ($\gamma_{C\text{-}H}$), 701 ($\gamma_{C\text{-}H}$), 631 ($\gamma_{C\text{-}H}$); GC [100–260 (10 °C/min)]: t_R = 2.32 min; HPLC [*n*-hexane-*i*-PrOH (95:5, v/v); f = 0.9 mL/min (*p* = 4.1 MPa); λ = 210 nm (Chiralcel OD-H)]: t_R = 12.395 min (*R*-isomer) and 13.249 min (*S*-isomer).

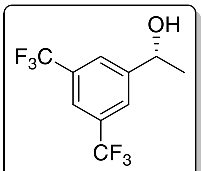

(*R*)-**2i**

R_f [*n*-hexane/AcOEt (90:10, v/v)] 0.20; ^{1}H NMR (500 MHz, $CDCl_3$) δ 7.84 (s, 2H), 7.79 (s, 1H), 5.04 (q, *J* = 6.5 Hz, 1H), 2.05 (br. s, 1H), 1.55 (d, *J* = 6.5 Hz, 3H); ^{13}C{^{1}H} NMR (126 MHz, $CDCl_3$) δ 148.4, 131.9 (q, *J* = 33.6 Hz), 125.8, 123.5 (q, *J* = 273.2 Hz), 121.4, 69.4, 25.7; ^{19}F{^{1}H} NMR (470 MHz, $CDCl_3$) δ –68.2 (s, 6F); FTMS (ESI-TOF) *m/z*: $[M+H]^+$ Calcd for $C_{10}H_9F_6O^+$ m/z: 259.05521, Found 259.18987; $[M+H–H_2O]^+$ Calcd for $C_{10}H_7F_6^+$ m/z: 241.04465, Found 241.04414; $[M–H]^–$ Calcd for $C_{10}H_7F_6O^–$ m/z: 257.04066, Found 257.04205; ATR-FTIR (neat): ν_{max} = 3254 (br, $\nu_{O\text{-}H}$), 1373 ($\gamma_{C\text{-}H}$), 1274 ($\gamma_{C\text{-}H}$), 1112 ($\nu_{C\text{-}O}$), 895 ($\gamma_{C\text{-}H}$), 841 ($\gamma_{C\text{-}H}$), 704 ($\gamma_{C\text{-}H}$), 681 ($\gamma_{C\text{-}H}$); GC [100–260 (10 °C/min)]: t_R = 1.84 min; HPLC [*n*-hexane-*i*-PrOH (98:2, v/v); f = 0.8 mL/min (*p* = 3.5 MPa); λ = 210 nm (Chiralcel OD-H)]: t_R = 8.737 min (*S*-isomer) and 9.774 min (*R*-isomer).

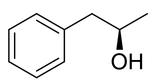

(*R*)-**2j**

R_f [Petroleum ether/Et_2O (20:10, v/v)] 0.38; ^{1}H NMR (500 MHz, $CDCl_3$) δ 7.34–7.31 (m, 2H), 7.28–7.19 (m, 3H), 4.10–3.95 (m, 1H), 2.75 (ddd, *J* = 21.4, 13.5, 6.2 Hz, 2H), 1.59 (d, *J* = 3.9 Hz, 1H), 1.25 (d, *J* = 6.2 Hz, 3H); ^{13}C{^{1}H} NMR (126 MHz, $CDCl_3$) δ 138.6, 129.5, 128.7, 126.6, 69.0, 45.9, 22.9; FTMS (ESI-TOF) *m/z*: $[M+H]^+$ Calcd for $C_9H_{13}O^+$ m/z: 137.09609, Not Found; $[M+H–H_2O]^+$ Calcd for $C_9H_{11}^+$ m/z:

119.08553, Found 119.08585; ATR-FTIR (neat): ν_{max} = 3348 (br, $\nu_{O\text{-}H}$), 1452 ($\gamma_{C\text{-}H}$), 111577 ($\gamma_{C\text{-}H}$), 1077 ($\nu_{C\text{-}O}$), 938 ($\gamma_{C\text{-}H}$), 740 ($\gamma_{C\text{-}H}$), 697 ($\gamma_{C\text{-}H}$), 599 ($\gamma_{C\text{-}H}$); GC [100–260 (10 °C/min)]: t_R = 3.33 min; HPLC [*n*-hexane-*i*-PrOH (99:1, v/v); f = 0.8 mL/min (p = 3.5 MPa); λ = 205 nm (Chiralcel OD-H)]: t_R = 17.631 min (*S*-isomer) and 19.877 min (*R*-isomer).

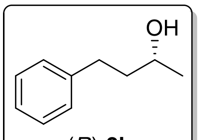

(*R*)-**2k**

R_f [*n*-hexane/AcOEt (90:10, v/v)] 0.11; ^{1}H NMR (500 MHz, $CDCl_3$) δ 7.33–7.27 (m, 2H), 7.24–7.17 (m, 3H), 3.84 (dd, J = 12.4, 6.2 Hz, 1H), 2.81–2.73 (m, 1H), 2.68–2.65 (m, 1H), 1.85–1.71 (m, 2H), 1.54 (s, 1H), 1.24 (d, J = 6.2 Hz, 3H); $^{13}C\{^1H\}$ NMR (126 MHz, $CDCl_3$) δ 142.2, 128.5, 125.9, 67.7, 41.0, 32.3, 23.8; FTMS (ESI-TOF) *m/z*: $[M+H]^+$ Calcd for $C_{10}H_{15}O^+$ m/z: 151.11174, Not Found; $[M+H–H_2O]^+$ Calcd for $C_{10}H_{13}^+$ m/z: 133.10118, Found 133.10141; ATR-FTIR (neat): ν_{max} = 3354 (br, $\nu_{O\text{-}H}$), 1454 ($\gamma_{C\text{-}H}$), 1126 ($\gamma_{C\text{-}H}$), 1053 ($\nu_{C\text{-}O}$), 855 ($\gamma_{C\text{-}H}$), 743 ($\gamma_{C\text{-}H}$), 696 ($\gamma_{C\text{-}H}$); GC [100–260 (10 °C/min)]: t_R = 4.71 min; HPLC [*n*-hexane-*i*-PrOH (90:10, v/v); f = 0.8 mL/min (p = 3.7 MPa); λ = 210 nm (Chiralcel OD-H)]: t_R = 7.799 min (*R*-isomer) and 10.264 min (*S*-isomer).

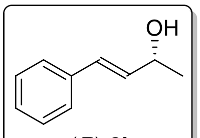

(*R*)-**2l**

R_f [Petroleum ether/AcOEt (20:10, v/v)] 0.44; ^{1}H NMR (500 MHz, $CDCl_3$) δ 7.38 (dt, J = 2.6, 1.7 Hz, 2H), 7.35–7.30 (m, 2H), 7.27–7.22 (m, 1H), 6.57 (d, J = 15.9 Hz, 1H), 6.27 (dd, J = 15.9, 6.4 Hz, 1H), 4.53–4.46 (m, 1H), 1.77 (s, 1H), 1.38 (d, J = 6.4 Hz, 3H); $^{13}C\{^1H\}$ NMR (126 MHz, $CDCl_3$) δ 136.8, 133.7, 129.5, 128.7, 127.8, 126.6, 69.1, 23.6; FTMS (ESI-TOF) *m/z*: $[M+H]^+$ Calcd for $C_{10}H_{13}O^+$ m/z: 149.09609, Not Found; $[M+H–H_2O]^+$ Calcd for $C_{10}H_{11}^+$ m/z: 131.08553, Found 131.08568; ATR-FTIR (neat): ν_{max} = 3421 (br, $\nu_{O\text{-}H}$), 1359 ($\gamma_{C\text{-}H}$), 1141 ($\gamma_{C\text{-}H}$), 1057 ($\nu_{C\text{-}O}$), 963 ($\gamma_{C\text{-}H}$), 934 ($\gamma_{C\text{-}H}$), 747 ($\gamma_{C\text{-}H}$), 690 ($\gamma_{C\text{-}H}$); GC [150–260 (10 °C/min)]: t_R = 2.52 min; HPLC [*n*-hexane-*i*-PrOH (95:5, v/v); f = 0.9 mL/min (p = 4.1 MPa); λ = 210 nm (Chiralcel OD-H)]: t_R = 15.259 min (*R*-isomer) and 24.625 min (*S*-isomer).

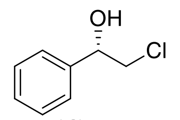

(*S*)-**2m**

R_f [*n*-hexane/AcOEt (90:10, v/v)] 0.2; ^{1}H NMR (500 MHz, $CDCl_3$) δ 7.42–7.36 (m, 4H), 7.35–7.33 (m, 1H), 4.90 (dd, J = 8.8, 3.4 Hz, 1H), 3.74 (dd, J = 11.3, 3.4 Hz, 1H), 3.65 (dd, J = 11.3, 8.8 Hz, 1H), 2.76 (br. s, 1H); $^{13}C\{^1H\}$ NMR (126 MHz, $CDCl_3$) δ 140.0, 128.8, 128.6, 126.2, 74.2, 51.0; FTMS (ESI-TOF) *m/z*: $[M+H]^+$ Calcd for $C_8H_{10}ClO^+$ m/z: 157.04147, Not Found; $[M+H–H_2O]^+$ Calcd for $C_8H_8Cl^+$ m/z: 139.03090, Found 139.03070; ATR-FTIR (neat): ν_{max} = 3368 (br, $\nu_{O\text{-}H}$), 1454 ($\gamma_{C\text{-}H}$), 1062 ($\nu_{C\text{-}O}$), 763 ($\gamma_{C\text{-}H}$), 720 ($\gamma_{C\text{-}H}$), 696 ($\gamma_{C\text{-}H}$), 612 ($\gamma_{C\text{-}H}$); GC [100–260 (10 °C/min)]: t_R

= 5.25 min; HPLC [*n*-hexane-*i*-PrOH (98:2, v/v); f = 1.0 mL/min (p = 4.4 MPa); λ = 220 nm (Chiralcel OD-H)]: t_R = 18.214 min (*S*-isomer) and 20.209 min (*R*-isomer).

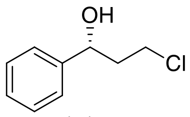

(*R*)-**2n**

R_f [*n*-hexane/AcOEt (80:20, v/v)] 0.42; ^{1}H NMR (500 MHz, $CDCl_3$) δ 7.41–7.34 (m, 4H), 7.33–7.27 (m, 1H), 4.94 (dd, J = 8.5, 4.7 Hz, 1H), 3.76–3.71 (m, 1H), 3.58–3.54 (m, 1H), 2.26–2.21 (m, 1H), 2.10–2.07 (m, 2H), 1.63 (br. s, 0.1H); ^{13}C{^{1}H} NMR (126 MHz, $CDCl_3$) δ 143.8, 128.8, 129.1, 125.9, 71.5, 41.8, 41.6; FTMS (ESI-TOF) *m/z*: $[M+H]^+$ Calcd for $C_9H_{12}ClO^+$ m/z: 171.05712, Not Found; $[M+H–Cl]^+$ Calcd for $C_9H_{11}O^+$ m/z: 135.08044, Found 135.08037; ATR-FTIR (neat): ν_{max} = 3213 (br, $\nu_{O\text{-}H}$), 1454 ($\gamma_{C\text{-}H}$), 1062 ($\nu_{C\text{-}O}$), 763 ($\gamma_{C\text{-}H}$), 720 ($\gamma_{C\text{-}H}$), 696 ($\gamma_{C\text{-}H}$), 612 ($\gamma_{C\text{-}H}$); GC [120–260 (10 °C/min)]: t_R = 5.28 min; HPLC [*n*-hexane-*i*-PrOH (95:5, v/v); f = 0.9 mL/min (p = 4.1 MPa); λ = 210 nm (Chiralcel OD-H)]: t_R = 11.156 min (*R*-isomer) and 13.505 min (*S*-isomer).

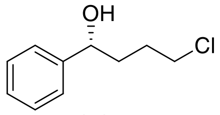

(*R*)-**2o**

R_f [petroleum ether/Et_2O (60:10, v/v)] 0.12; ^{1}H NMR (500 MHz, $CDCl_3$) δ 7.39–7.31 (m, 4H), 7.31–7.26 (m, 1H), 4.75–4.63 (m, 1H), 3.65–3.48 (m, 2H), 2.02 (br. s, 1H), 1.97–1.75 (m, 4H); ^{13}C{^{1}H} NMR (126 MHz, $CDCl_3$) δ 144.4, 128.7, 127.9, 125.9, 74.0, 45.1, 36.3, 29.1; FTMS (ESI-TOF) *m/z*: $[M+H]^+$ Calcd for $C_{10}H_{14}ClO^+$ m/z: 185.07277, Found 185.05717; ATR-FTIR (neat): ν_{max} = 3357 (br, $\nu_{O\text{-}H}$), 1452 ($\gamma_{C\text{-}H}$), 1059 ($\nu_{C\text{-}O}$), 757 ($\gamma_{C\text{-}H}$), 699 ($\gamma_{C\text{-}H}$), 648 ($\gamma_{C\text{-}H}$); GC [120–260 (10 °C/min)]: t_R = 6.74 min; HPLC [*n*-hexane-*i*-PrOH (98:2, v/v); f = 1.0 mL/min (p = 4.4 MPa); λ = 210 nm (Chiralcel OD-H)]: t_R = 23.391 min (*R*-isomer) and 26.377 min (*S*-isomer).

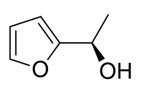

(*R*)-**2p**

R_f [Petroleum ether/AcOEt (95:5, v/v)] 0.33; ^{1}H NMR (500 MHz, $CDCl_3$) δ 7.36 (dd, J = 1.8, 0.8 Hz, 1H), 6.32 (dd, J = 3.2, 1.8 Hz, 1H), 6.22 (dt, J = 3.2, 0.8 Hz, 1H), 4.87 (d, J = 6.2 Hz, 1H), 2.14 (br. s, 1H), 1.53 (d, J = 6.6 Hz, 3H); ^{13}C{^{1}H} NMR (126 MHz, $CDCl_3$) δ 157.7, 142.0, 110.2, 105.2, 63.7, 21.4; FTMS (ESI-TOF) *m/z*: $[M+H]^+$ Calcd for $C_6H_9O_2^+$ m/z: 113.05970, Not Found; $[M+H–OH]^+$ Calcd for $C_6H_7O^+$ m/z: 95.04914, Found 95.04976; ATR-FTIR (neat): ν_{max} = 3337 (br, $\nu_{O\text{-}H}$), 1228 ($\gamma_{C\text{-}H}$), 1066 ($\nu_{C\text{-}O}$), 1007 ($\gamma_{C\text{-}H}$), 877 ($\gamma_{C\text{-}H}$), 809 ($\gamma_{C\text{-}H}$), 733 ($\gamma_{C\text{-}H}$); GC [100–260 (10 °C/min)]: t_R = 1.40 min; HPLC [*n*-hexane-*i*-PrOH (95:5, v/v); f = 0.8 mL/min (p = 3.4 MPa); λ = 220 nm (Chiralcel OJ-H)]: t_R = 11.332 min (*S*-isomer) and 12.586 min (*R*-isomer).

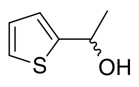

rac-**2q**

R_f [*n*-hexane/AcOEt (80:20, v/v)] 0.34; ^{1}H NMR (500 MHz, $CDCl_3$) δ 7.28–7.20 (m, 1H), 6.99–6.95 (m, 2H), 5.13 (q, J = 6.3 Hz, 1H), 2.09 (br. s, 1H), 1.60 (d, J = 6.4 Hz, 3H); $^{13}C\{^1H\}$ NMR (126 MHz, $CDCl_3$) δ 150.0, 126.8, 124.6, 123.3, 66.4, 25.4; FTMS (ESI-TOF) *m/z*: $[M+H]^+$ Calcd for $C_6H_9OS^+$ m/z: 129.03686, Not Found, Found 124.07590; ATR-FTIR (neat): ν_{max} = 3328 (br, $\nu_{O\text{-}H}$), 1435 ($\gamma_{C\text{-}H}$), 1065 ($\nu_{C\text{-}O}$), 1004 ($\gamma_{C\text{-}H}$), 849 ($\gamma_{C\text{-}H}$), 693 ($\gamma_{C\text{-}H}$); GC [120–260 (10 °C/min)]: t_R = 2.02 min; HPLC [*n*-hexane-*i*-PrOH (95:5, v/v); f = 0.8 mL/min (p = 3.4 MPa); λ = 233 nm (Chiralcel OJ-H)]: t_R = 13.836 min (*S*-isomer) and 17.395 min (*R*-isomer).

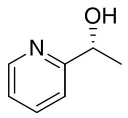

(*R*)-**2r**

R_f [*n*-hexane/AcOEt (50:50, v/v)] 0.32; ^{1}H NMR (500 MHz, $CDCl_3$) δ 8.55–8.47 (m, 1H), 7.69–7.66 (m, 1H), 7.30–7.24 (m, 1H), 7.22–7.14 (m, 1H), 4.88 (q, J = 6.4 Hz, 1H), 4.35 (br. s, 1H), 1.49 (d, J = 6.6 Hz, 3H); $^{13}C\{^1H\}$ NMR (126 MHz, $CDCl_3$) δ 163.2, 148.3, 136.9, 122.3, 119.9, 69.0, 24.4; FTMS (ESI-TOF) *m/z*: $[M+H]^+$ Calcd for $C_7H_{10}NO^+$ m/z: 124.07569, Found 124.07593; ATR-FTIR (neat): ν_{max} = 3372 (br, $\nu_{O\text{-}H}$), 1594 ($\gamma_{C\text{-}H}$), 1435 ($\gamma_{C\text{-}H}$), 1080 ($\nu_{C\text{-}O}$), 902 ($\gamma_{C\text{-}H}$), 785 ($\gamma_{C\text{-}H}$), 749 ($\gamma_{C\text{-}H}$), 605 ($\gamma_{C\text{-}H}$); GC [120–260 (10 °C/min)]: t_R = 2.09 min; HPLC [*n*-hexane-*i*-PrOH (99:1, v/v); f = 1.0 mL/min (p = 3.7 MPa); λ = 258 nm (Chiralpak Lux i-Cellulose 5)]: t_R = 43.482 min (*S*-isomer) and 46.830 min (*R*-isomer).

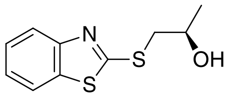

(*R*)-**2s**

R_f [*n*-hexane/AcOEt (80:20, v/v)] 0.22; ^{1}H NMR (500 MHz, $CDCl_3$) δ 7.87–7.81 (m, 1H), 7.76–7.71 (m, 1H), 7.41 (ddd, J = 8.3, 7.3, 1.2 Hz, 1H), 7.30 (ddd, J = 8.4, 7.4, 1.2 Hz, 1H), 4.28 (br. s, 1H), 4.28– 4.22 (m, 1H), 3.51 (dd, J = 14.4, 3.0 Hz, 1H), 3.34 (dd, J = 14.3, 6.9 Hz, 1H), 1.36 (d, J = 6.2 Hz, 3H); $^{13}C\{^1H\}$ NMR (126 MHz, $CDCl_3$) δ 167.8, 152.6, 135.5, 126.4, 124.7, 121.5, 121.2, 67.6, 42.3, 22.7; FTMS (ESI-TOF) *m/z*: $[M+H]^+$ Calcd for $C_{10}H_{12}NOS_2^+$ m/z: 226.03548, Found 226.03508; ATR-FTIR (neat): ν_{max} = 3327 (br, $\nu_{O\text{-}H}$), 1455 ($\gamma_{C\text{-}H}$), 1423 ($\gamma_{C\text{-}H}$), 1076 ($\nu_{C\text{-}O}$), 993 ($\gamma_{C\text{-}H}$), 752 ($\gamma_{C\text{-}H}$), 724 ($\gamma_{C\text{-}H}$); GC [220–260 (10 °C/min)]: t_R = 3.89 min; HPLC [*n*-hexane-*i*-PrOH (95:5, v/v); f = 0.7 mL/min (p = 3.1 MPa); λ = 225 nm (Chiralcel OD-H)]: t_R = 22.202 min (*R*-isomer) and 24.051 min (*S*-isomer).

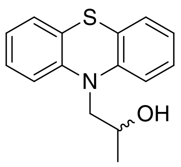

rac-**2t**

R_f [*n*-hexane/AcOEt (80:20, v/v)] 0.18; ^{1}H NMR (500 MHz, $CDCl_3$) δ 7.25–7.14 (m, 4H), 7.03–6.87 (m, 4H), 4.25–4.13 (m, 1H), 3.98 (dd, J = 13.5, 3.4 Hz, 1H), 3.79 (dd, J = 13.5, 8.9 Hz, 1H), 2.45 (br. s, 1H), 1.29 (d, J = 6.2 Hz, 3H); $^{13}C\{^1H\}$ NMR (126 MHz, $CDCl_3$) δ 145.5, 127.9, 127.5, 126.8,

123.2, 116.3, 63.6, 55.3, 20.0; FTMS (ESI-TOF) *m/z*: $[M+H]^+$ Calcd for $C_{15}H_{16}NOS^+$ m/z: 258.09471, Found 258.09438; ATR-FTIR (neat): ν_{max} = 3500 ($\nu_{O\text{-}H}$), 1590 ($\gamma_{C\text{-}H}$), 1451 ($\gamma_{C\text{-}H}$), 1250 ($\gamma_{C\text{-}H}$), 1033 ($\nu_{C\text{-}O}$), 943 ($\gamma_{C\text{-}H}$), 744 ($\gamma_{C\text{-}H}$), 732 ($\gamma_{C\text{-}H}$), 625 ($\gamma_{C\text{-}H}$); GC [260 (const.)]: t_R = 5.08 min; HPLC [*n*-hexane-*i*-PrOH (90:10, v/v); f = 0.8 mL/min (p = 3.7 MPa); λ = 254 nm (Chiralcel OD-H)]: t_R = 8.650 min (*R*-isomer) and 9.501 min (*S*-isomer).

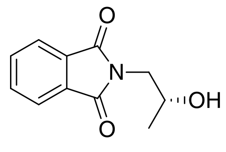

(*R*)-**2u**

R_f [$CHCl_3$/Acetone (98:2, v/v)] 0.24; ^{1}H NMR (500 MHz, $CDCl_3$) δ 7.88–7.81 (m, 2H), 7.72 (td, J = 5.3, 2.1 Hz, 2H), 4.11 (ddd, J = 12.2, 6.3, 3.9 Hz, 1H), 3.74 (qd, J = 14.2, 5.6 Hz, 2H), 2.43 (d, J = 5.7 Hz, 1H), 1.25 (d, J = 6.3 Hz, 3H); ^{13}C{^{1}H} NMR (126 MHz, $CDCl_3$) δ 169.1, 134.2, 132.1, 123.6, 66.9, 45.7, 21.2; FTMS (ESI-TOF) *m/z*: $[M+H]^+$ Calcd for $C_{11}H_{12}NO_3^+$ m/z: 206.08117, Found 206.08109; ATR-FTIR (neat): ν_{max} = 3480 (br, $\nu_{O\text{-}H}$), 1765 ($\gamma_{C=O}$), 1698 ($\delta_{N\text{-}H}$), 1390 ($\gamma_{C\text{-}H}$), 1037 ($\nu_{C\text{-}O}$), 918 ($\gamma_{C\text{-}H}$), 835 ($\gamma_{C\text{-}H}$), 795 ($\gamma_{C\text{-}H}$), 723 ($\gamma_{C\text{-}H}$), 711 ($\gamma_{C\text{-}H}$); GC [220–260 (10 °C/min)]: t_R = 2.69 min; HPLC [*n*-hexane-*i*-PrOH (90:10, v/v); f = 0.8 mL/min (p = 3.7 MPa); λ = 216 nm (Chiralcel OD-H)]: t_R = 17.310 min (*R*-isomer) and 18.748 min (*S*-isomer).

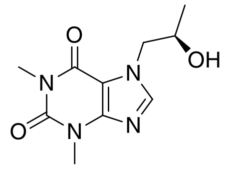

(*R*)-**2v**

R_f [$CHCl_3$/MeOH (98:2, v/v)] 0.22; ^{1}H NMR (500 MHz, $CDCl_3$) δ 7.60 (s, 1H), 4.47 (dd, J = 13.9, 3.0 Hz, 1H), 4.20–4.19 (m, 1H), 4.09 (dd, J = 13.9, 7.5 Hz, 1H), 3.55 (s, 3H), 3.37 (s, 3H), 3.01 (d, J = 4.7 Hz, 1H), 1.26 (d, J = 6.3 Hz, 3H); ^{13}C{^{1}H} NMR (126 MHz, $CDCl_3$) δ 156.0, 151.6, 149.0, 142.3, 107.3, 66.8, 53.7, 30.0, 28.2, 20.7; FTMS (ESI-TOF) *m/z*: $[M+H]^+$ Calcd for $C_{10}H_{15}N_4O_3^+$ m/z: 239.11387, Found 239.11373; ATR-FTIR (neat): ν_{max} = 3488 (br, $\nu_{O\text{-}H}$), 1698 ($\gamma_{C=O}$), 1656 ($\delta_{N\text{-}H}$), 1547 ($\delta_{N\text{-}H}$), 1400 ($\gamma_{C\text{-}H}$), 1023 ($\nu_{C\text{-}O}$), 971 ($\gamma_{C\text{-}H}$), 759 ($\gamma_{C\text{-}H}$), 746 ($\gamma_{C\text{-}H}$); GC [260 (const.)]: t_R = 4.01 min; HPLC [*n*-hexane-*i*-PrOH (78:22, v/v); f = 1.0 mL/min (p = 5.5 MPa); λ = 273 nm (Chiralpak AD-H)]: t_R = 7.621 min (*S*-isomer) and 8.877 min (*R*-isomer).

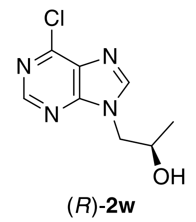

(*R*)-**2w**

R_f [CH_2Cl_2/MeOH (95:5, v/v)] 0.33; ^{1}H NMR (500 MHz, $CDCl_3$) δ 8.68 (s, 1H), 8.18 (s, 1H), 4.42 (dd, J = 14.1, 2.7 Hz, 1H), 4.33–4.29 (m, 1H), 4.12 (dd, J = 14.1, 8.1 Hz, 1H), 3.51 (br. s, 1H), 1.31 (d, J = 6.3 Hz, 3H); ^{13}C{^{1}H} NMR (126 MHz, $CDCl_3$) δ 152.0, 151.9, 150.8, 146.6, 131.2, 66.1, 51.7, 21.0; FTMS (ESI-TOF) *m/z*: $[M+H]^+$ Calcd for $C_8H_{10}ClN_4O^+$ m/z: 213.05377, Found 213.05363; ATR-FTIR (neat): ν_{max} = 3256 (br, $\nu_{O\text{-}H}$), 1594 ($\gamma_{C=O}$), 1563 ($\delta_{N\text{-}}$

H), 1400 (γ_{C-H}), 1334 (γ_{C-H}), 1215 (γ_{C-H}), 1075 (ν_{C-O}), 930 (γ_{C-H}), 792 (γ_{C-H}), 763 (γ_{C-H}), 631 (γ_{C-H}); GC [260 (const.)]: t_R = 2.81 min; HPLC [*n*-hexane-*i*-PrOH (90:10, v/v); f = 1.0 mL/min (p = 4.5 MPa); λ = 264 nm (Chiralcel OJ-H)]: t_R = 16.210 min (*S*-isomer) and 17.868 min (*R*-isomer).

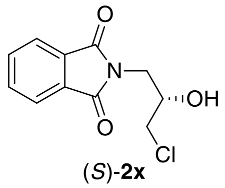
(*S*)-**2x**

R_f [$CHCl_3$/Acetone (98:2, v/v)] 0.73; ^1H NMR (500 MHz, $CDCl_3$) δ 7.92–7.82 (m, 2H), 7.78–7.67 (m, 2H), 4.19–4.14 (m, 1H), 3.95 (dd, J = 14.3, 7.5 Hz, 1H), 3.91–3.83 (m, 1H), 3.67 (dd, J = 11.4, 4.7 Hz, 1H), 3.61 (dd, J = 11.4, 5.4 Hz, 1H), 2.93 (d, J = 6.5 Hz, 1H); $^{13}C\{^1H\}$ NMR (126 MHz, $CDCl_3$) δ 168.8, 134.3, 132.0, 123.7, 69.9, 47.4, 41.7; FTMS (ESI-TOF) *m/z*: $[M+H]^+$ Calcd for $C_{11}H_{11}ClNO_3^+$ m/z: 240.04220, Found 240.04208; ATR-FTIR (neat): ν_{max} = 3462 (br, ν_{O-H}), 1772 ($\gamma_{C=O}$), 1689 (δ_{N-H}), 1395 (γ_{C-H}), 1304 (γ_{C-H}), 1077 (γ_{C-H}), 1037 (ν_{C-O}), 845 (γ_{C-H}), 716 (γ_{C-H}), 688 (γ_{C-H}); GC [220–260 (10 °C/min)]: t_R = 4.25 min; HPLC [*n*-hexane-*i*-PrOH (90:10, v/v); f = 0.8 mL/min (p = 3.7 MPa); λ = 220 nm (Chiralcel OD-H)]: t_R = 22.862 min (*R*-isomer) and 30.254 min (*S*-isomer).

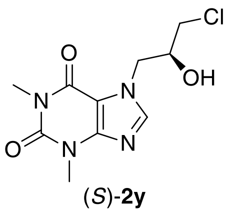
(*S*)-**2y**

R_f [$CHCl_3$/MeOH (98:2, v/v)] 0.30; ^1H NMR (500 MHz, $CDCl_3$) δ 7.66 (s, 1H), 4.60 (dd, J = 14.4, 3.1 Hz, 1H), 4.41 (dd, J = 14.4, 6.9 Hz, 1H), 4.27–4.19 (m, 1H), 4.08 (d, J = 5.1 Hz, 1H), 3.63–3.49 (m, 5H), 3.38 (s, 3H); $^{13}C\{^1H\}$ NMR (126 MHz, $CDCl_3$) δ 156.3, 151.5, 149.3, 142.7, 107.3, 70.3, 49.7, 45.8, 30.0, 28.3; FTMS (ESI-TOF) *m/z*: $[M+H]^+$ Calcd for $C_{10}H_{14}ClN_4O_3^+$ m/z: 273.07489, Found 273.07439; ATR-FTIR (neat): ν_{max} = 3421 (br, ν_{O-H}), 1693 ($\gamma_{C=O}$), 1649 ($\gamma_{C=O}$), 1547 (γ_{C-H}), 1431 (γ_{C-H}), 1095 (ν_{C-O}), 1026 (ν_{C-O}), 763 (γ_{C-H}), 746 (γ_{C-H}), 661 (γ_{C-H}); GC [260 (const.)]: t_R = 6.56 min; HPLC [*n*-hexane-*i*-PrOH (78:22, v/v); f = 0.3 mL/min (p = 1.6 MPa); λ = 273 nm (Chiralpak AD-H)]: t_R = 35.534 min (*R*-isomer) and 38.908 min (*S*-isomer).

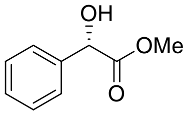
(*S*)-**2z**

R_f [*n*-hexane/AcOEt (80:20, v/v)] 0.25; ^1H NMR (500 MHz, $CDCl_3$) δ 7.45–7.40 (m, 2H), 7.39–7.31 (m, 3H), 5.18 (s, 1H), 3.76 (s, 3H), 3.53 (br. s, 1H); $^{13}C\{^1H\}$ NMR (126 MHz, $CDCl_3$) δ 174.2, 138.4, 128.7, 128.6, 126.7, 73.0, 53.1; FTMS (ESI-TOF) *m/z*: $[M+H]^+$ Calcd for $C_9H_{11}O_3^+$ m/z: 167.07027, Not Found; $[M+H–H_2O]^+$ Calcd for $C_9H_9O_2^+$ m/z: 149.05970, Found 149.05981; ATR-FTIR (neat): ν_{max} = 3436 (br, ν_{O-H}), 1736 ($\gamma_{C=O}$), 1187 (γ_{C-H}), 1095 (ν_{C-O}), 1067 (ν_{C-O}), 980 (γ_{C-H}), 732 (γ_{C-H}), 694 (γ_{C-H}); GC [120–260 (10 °C/min)]: t_R = 4.17 min; HPLC [*n*-hexane-*i*-PrOH (90:10,

v/v); f = 0.8 mL/min (p = 3.7 MPa); λ = 225 nm (Chiralcel OD-H)]: t_R = 9.274 min (*S*-isomer) and 15.083 min (*R*-isomer).

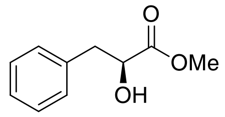

(*S*)-**2aa**

R_f [*n*-hexane/AcOEt (90:10, v/v)] 0.29; ^{1}H NMR (500 MHz, $CDCl_3$) δ 7.32–7.29 (m, 2H), 7.28–7.19 (m, 3H), 4.46 (dd, *J* = 6.8, 4.4 Hz, 1H), 3.77 (s, 3H), 3.13 (dd, *J* = 13.9, 4.4 Hz, 1H), 2.97 (dd, *J* = 13.9, 6.8 Hz, 1H), 2.76 (br. s, 1H); ^{13}C{^{1}H} NMR (126 MHz, $CDCl_3$) δ 174.7, 136.4, 129.6, 128.6, 127.0, 71.4, 52.6, 40.7; FTMS (ESI-TOF) *m/z*: $[M+H]^+$ Calcd for $C_{10}H_{13}O_3^+$ m/z: 181.08592, Found 181.08582; $[M+Na]^+$ Calcd for $C_{10}H_{12}NaO_3^+$ m/z: 203.06787, Found 203.06787; ATR-FTIR (neat): ν_{max} = 3262 (br, $\nu_{O\text{-}H}$), 1746 ($\gamma_{C=O}$), 1429 ($\gamma_{C\text{-}H}$), 1277 ($\gamma_{C\text{-}H}$), 1210 ($\gamma_{C\text{-}H}$), 1100 ($\nu_{C\text{-}O}$), 1077 ($\nu_{C\text{-}O}$), 1011 ($\nu_{C\text{-}O}$), 756 ($\gamma_{C\text{-}H}$), 699 ($\gamma_{C\text{-}H}$); GC [120–260 (10 °C/min)]: t_R = 4.91 min; HPLC [*n*-hexane-*i*-PrOH (90:10, v/v); f = 0.5 mL/min (p = 2.2 MPa); λ = 210 nm (Chiralcel OJ-H)]: t_R = 26.511 min (*R*-isomer) and 29.606 min (*S*-isomer).

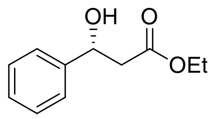

(*R*)-**2ab**

R_f [*n*-hexane/AcOEt (80:20, v/v)] 0.36; ^{1}H NMR (500 MHz, $CDCl_3$) δ 7.41–7.32 (m, 4H), 7.29–7.27 (m, 1H), 5.13 (dt, *J* = 8.9, 3.7 Hz, 1H), 4.18 (q, *J* = 7.2 Hz, 2H), 3.33 (d, *J* = 3.5 Hz, 1H), 2.73 (qd, *J* = 16.3, 6.4 Hz, 2H), 1.26 (t, *J* = 7.2 Hz, 3H); ^{13}C{^{1}H} NMR (126 MHz, $CDCl_3$) δ 172.5, 142.7, 128.6, 127.9, 125.8, 70.4, 61.0, 43.5, 14.3; FTMS (ESI-TOF) *m/z*: $[M+H]^+$ Calcd for $C_{11}H_{15}O_3^+$ m/z: 195.10157, Not Found; $[M+Na]^+$ Calcd for $C_{11}H_{14}NaO_3$+ m/z: 217.08352, Found 217.08341; ATR-FTIR (neat): ν_{max} = 3462 (br, $\nu_{O\text{-}H}$), 1716 ($\gamma_{C=O}$), 1296 ($\gamma_{C\text{-}O}$), 1194 ($\gamma_{C\text{-}O}$), 1036 ($\gamma_{C\text{-}O}$), 759 ($\gamma_{C\text{-}H}$), 699 ($\gamma_{C\text{-}H}$); GC [150–260 (10 °C/min)]: t_R = 3.92 min; HPLC [*n*-hexane-*i*-PrOH (90:10, v/v); f = 0.8 mL/min (p = 3.7 MPa); λ = 210 nm (Chiralcel OD-H)]: t_R = 9.150 min (*S*-isomer) and 11.599 min (*R*-isomer).

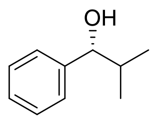

(*R*)-**2ac**

R_f [*n*-hexane/AcOEt (30:10, v/v)] 0.51; ^{1}H NMR (500 MHz, $CDCl_3$) δ 7.38–7.30 (m, 4H), 7.29–7.25 (m, 1H), 4.36 (d, *J* = 6.9 Hz, 1H), 1.96 (dq, *J* = 13.5, 6.8 Hz, 1H), 1.89 (br. s, 1H), 1.01 (d, *J* = 6.8 Hz, 3H), 0.80 (d, *J* = 6.8 Hz, 3H); ^{13}C{^{1}H} NMR (126 MHz, $CDCl_3$) δ 143.8, 128.3, 127.5, 126.7, 80.2, 35.4, 19.1, 18.4; FTMS (ESI-TOF) *m/z*: $[M+H]^+$ Calcd for $C_{10}H_{15}O^+$ m/z: 151.11174, Not Found; $[M+H–H_2O]^+$ Calcd for $C_{10}H_{13}^+$ m/z: 133.10118, Found 133.10118; ATR-FTIR (neat): ν_{max} = 3387 (br, $\nu_{O\text{-}H}$), 1452 ($\gamma_{C\text{-}H}$), 1019 ($\nu_{C\text{-}O}$), 759 ($\gamma_{C\text{-}H}$), 699 ($\gamma_{C\text{-}H}$); GC [120–260 (10 °C/min)]: t_R = 2.82 min; HPLC [*n*-hexane-*i*-PrOH (98:2, v/v); f = 1.0 mL/min (p = 4.4 MPa); λ = 210 nm (Chiralcel OD-H)]: t_R = 9.755 min (*S*-isomer) and 12.093 min (*R*-isomer).

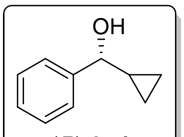

(R)-**2ad**

R_f [n-hexane/AcOEt (30:10, v/v)] 0.42; ^{1}H NMR (500 MHz, $CDCl_3$) δ 7.44–7.42 (m, 2H), 7.39–7.33 (m, 2H), 7.32–7.27 (m, 1H), 4.02 (d, J = 8.3 Hz, 1H), 2.00 (d, J = 2.3 Hz, 1H), 1.63 (br. s, 0.27H), 1.29–1.16 (m, 1H), 0.68–0.61 (m, 1H), 0.60–0.52 (m, 1H), 0.48 (dt, J = 9.5, 5.1 Hz, 1H), 0.38 (dt, J = 14.9, 4.7 Hz, 4H); ^{13}C{^{1}H} NMR (126 MHz, $CDCl_3$) δ 143.9, 128.5, 127.7, 126.2, 78.7, 19.3, 3.7, 3.0; FTMS (ESI-TOF) m/z: [M+H]$^+$ Calcd for $C_{10}H_{13}O^+$ m/z: 149.09609, Not Found; [M+H–H_2O]$^+$ Calcd for $C_{10}H_{11}^+$ m/z: 131.08553, Found 131.08567; ATR-FTIR (neat): ν_{max} = 3357 (br, $\nu_{O\text{-}H}$), 1452 1024 ($\nu_{C\text{-}O}$), 697 ($\gamma_{C\text{-}H}$); GC [120–260 (10 °C/min)]: t_R = 3.98 min; HPLC [n-hexane-i-PrOH (98:2, v/v); f = 1.0 mL/min (p = 4.3 MPa); λ = 204 nm (Chiralcel OJ-H)]: t_R = 21.053 min (S-isomer) and 24.549 min (R-isomer).

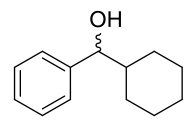

rac-**2ae**

R_f [n-hexane/AcOEt (80:20, v/v)] 0.65; ^{1}H NMR (500 MHz, $CDCl_3$) δ 7.38–7.24 (m, 5H), 4.36 (d, J = 7.2 Hz, 1H), 2.03–1.95 (m, 1H), 1.86 (s, 1H), 1.81–1.73 (m, 1H), 1.71–1.56 (m, 3H), 1.43–1.34 (m, 1H), 1.29–0.87 (m, 5H); ^{13}C{^{1}H} NMR (126 MHz, $CDCl_3$) δ 143.8, 128.3, 127.5, 126.8, 79.5, 45.1, 29.5, 29.0, 26.6, 26.2, 26.2; FTMS (ESI-TOF) m/z: [M+H]$^+$ Calcd for $C_{13}H_{19}O^+$ m/z: 191.14304, Not Found; [M+H–H_2O]$^+$ Calcd for $C_{13}H_{17}^+$ m/z: 173.13248, Found 173.13250; ATR-FTIR (neat): ν_{max} = 3436 (br, $\nu_{O\text{-}H}$), 1006 ($\nu_{C\text{-}O}$), 765 ($\gamma_{C\text{-}H}$), 693 ($\gamma_{C\text{-}H}$); GC [150–260 (10 °C/min)]: t_R = 4.92 min; HPLC [n-hexane-i-PrOH (95:5, v/v); f = 0.9 mL/min (p = 4.1 MPa); λ = 210 nm (Chiralcel OD-H)]: t_R = 7.461 min (S-isomer) and 9.032 min (R-isomer).

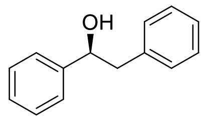

(S)-**2af**

R_f [n-hexane/AcOEt (90:10, v/v)] 0.27; ^{1}H NMR (500 MHz, $CDCl_3$) δ 7.43–7.14 (m, 10H), 4.91 (dd, J = 8.4, 4.9 Hz, 1H), 3.08–2.98 (m, 2H), 1.99 (d, J = 1.9 Hz, 1H), 1.57 (br. s, 0.18H); ^{13}C{^{1}H} NMR (126 MHz, $CDCl_3$) δ 143.9, 138.2, 129.6, 128.6, 128.5, 127.7, 126.7, 126.0, 75.5, 46.2; FTMS (ESI-TOF) m/z: [M+H]$^+$ Calcd for $C_{14}H_{15}O^+$ m/z: 199.11174, Not Found; [M+H–H_2O]$^+$ Calcd for $C_{14}H_{13}^+$ m/z: 181.10118, Found 181.10107; ATR-FTIR (neat): ν_{max} = 3299 (br, $\nu_{O\text{-}H}$), 1495 1026 ($\nu_{C\text{-}O}$), 742 ($\gamma_{C\text{-}H}$), 694 ($\gamma_{C\text{-}H}$); GC [150–260 (10 °C/min)]: t_R = 6.34 min; HPLC [n-hexane-i-PrOH (95:5, v/v); f = 0.9 mL/min (p = 4.1 MPa); λ = 210 nm (Chiralcel OD-H)]: t_R = 12.139 min (R-isomer) and 13.611 min (S-isomer).

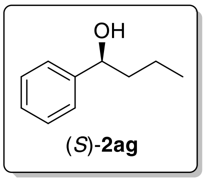

R_f [*n*-hexane/AcOEt (30:10, v/v)] 0.53; ^{1}H NMR (500 MHz, $CDCl_3$) δ 7.40–7.32 (m, 12H), 7.29–7.26 (m, 3H), 4.68 (dd, J = 7.6, 5.8 Hz, 1H), 1.87 (br. s, 1H), 1.84–1.75 (m, 1H), 1.69 (ddt, J = 13.6, 10.1, 5.8 Hz, 1H), 1.50–1.38 (m, 1H), 1.37–1.25 (m, 1H), 0.94 (t, J = 7.4 Hz, 3H); ^{13}C{^{1}H} NMR (126 MHz, $CDCl_3$) δ 145.1, 128.6, 127.6, 126.0, 74.6, 41.4, 19.2, 14.1; FTMS (ESI-TOF) *m/z*: $[M+H]^+$ Calcd for $C_{10}H_{15}O^+$ m/z: 151.11174, Not Found; $[M+H–OH]^+$ Calcd for $C_{10}H_{13}^+$ m/z: 133.10118, Found 133.10131; ATR-FTIR (neat): ν_{max} = 3348 (br, $\nu_{O\text{-}H}$), 1454 ($\gamma_{C\text{-}H}$), 1026 ($\nu_{C\text{-}O}$), 697 ($\gamma_{C\text{-}H}$); GC [120–260 (10 °C/min)]: t_R = 3.17 min; HPLC [*n*-hexane-*i*-PrOH (97:3, v/v); f = 1.0 mL/min (p = 4.3 MPa); λ = 220 nm (Chiralcel OJ-H)]: t_R = 8.585 min (*R*-isomer) and 9.251 min (*S*-isomer).

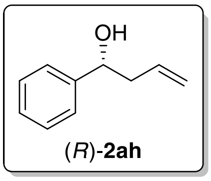

R_f [*n*-hexane/AcOEt (95:5, v/v)] 0.24; ^{1}H NMR (500 MHz, $CDCl_3$) δ 7.39–7.33 (m, 4H), 7.32–7.26 (m, 1H), 5.93–5.73 (m, 1H), 5.25–5.08 (m, 2H), 4.74 (dd, J = 7.8, 5.2 Hz, 1H), 2.63–2.43 (m, 2H), 2.07 (br. s, 1H); ^{13}C{^{1}H} NMR (126 MHz, $CDCl_3$) δ 144.0, 134.6, 128.6, 127.7, 125.9, 118.6, 73.4, 44.0; FTMS (ESI-TOF) *m/z*: $[M+H]^+$ Calcd for $C_{10}H_{13}O^+$ m/z: 149.09609, Not Found; $[M+H–H_2O]^+$ Calcd for $C_{10}H_{11}^+$ m/z: 131.08553, Not Found; $[M+H–OH]^+$ Calcd for $C_{10}H_{11}^+$ m/z: 131.08553, Found 131.08551; ATR-FTIR (neat): ν_{max} = 3377 (br, $\nu_{O\text{-}H}$), 1454 ($\gamma_{C\text{-}H}$), 1043 ($\nu_{C\text{-}O}$), 1027 ($\nu_{C\text{-}O}$), 912 ($\gamma_{C\text{-}H}$), 697 ($\gamma_{C\text{-}H}$); GC [120–260 (10 °C/min)]: t_R = 3.15 min; HPLC [*n*-hexane-*i*-PrOH (98:2, v/v); f = 1.0 mL/min (p = 4.4 MPa); λ = 220 nm (Chiralcel OD-H)]: t_R = 12.694 min (*S*-isomer) and 13.864 min (*R*-isomer).

Supplementary Table 11. Analytical separation conditions of studied compounds by GC column.

Compound	Temperature program [°C]	Retention time [min]
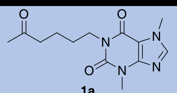 **1a**	260 (const.)	9.75
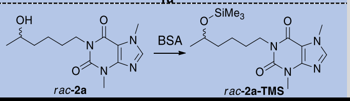 *rac*-**2a** → *rac*-**2a-TMS**		9.47 or 7.28 (silylated derivative)
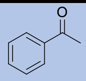 **1b**	100–260 (10 °C/min)	2.92
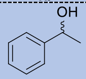 *rac*-**2b**		2.71
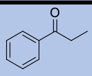 **1c**	100–260 (10 °C/min)	3.81
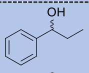 *rac*-**2c**		3.50
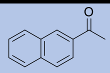 **1d**	150–260 (10 °C/min)	5.52
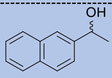 *rac*-**2d**		5.38
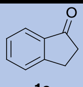 **1e**	150–260 (10 °C/min)	2.66
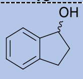 *rac*-**2e**		2.17
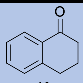 **1f**	150–260 (10 °C/min)	3.36
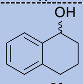 *rac*-**2f**		3.03
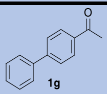 **1g**	170–260 (10 °C/min)	5.94
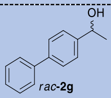 *rac*-**2g**		5.71
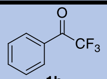 **1h**	100–260 (10 °C/min)	1.22
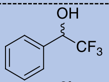 *rac*-**2h**		2.32
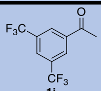 **1i**	100–260 (10 °C/min)	1.27
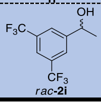 *rac*-**2i**		1.84

Supplementary Table 11. Analytical separation conditions of studied compounds by GC (cont.).

Compound	Temperature program [°C]	Retention time [min]
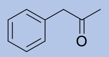 **1j**	100–260 (10 °C/min)	3.57
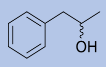 *rac*-**2j**		3.33
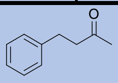 **1k**	100–260 (10 °C/min)	4.83
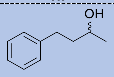 *rac*-**2k**		4.71
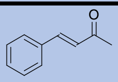 **1l**	150–260 (10 °C/min)	2.88
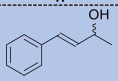 *rac*-**2l**		2.52
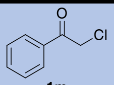 **1m**	100–260 (10 °C/min)	5.75
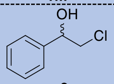 *rac*-**2m**		5.25
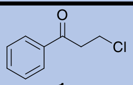 **1n**	120–260 (10 °C/min)	5.38
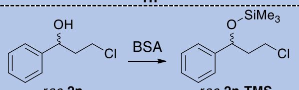 *rac*-**2n** → *rac*-**2n-TMS**		5.28 or 4.15 (silylated derivative)
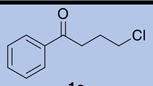 **1o**	120–260 (10 °C/min)	6.61
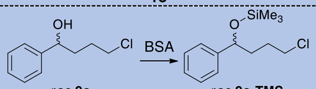 *rac*-**2o** → *rac*-**2o-TMS**		6.74 or 5.45 (silylated derivative)
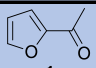 **1p**	100–260 (10 °C/min)	1.69
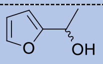 *rac*-**2p**		1.40
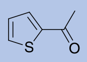 **1q**	120–260 (10 °C/min)	2.43
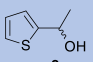 *rac*-**2q**		2.02
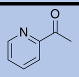 **1r**	120–260 (10 °C/min)	1.88
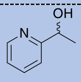 *rac*-**2r**		2.09

Supplementary Table 11. Analytical separation conditions of studied compounds by GC (cont.).

Compound	Temperature program [°C]	Retention time [min]
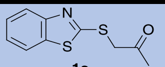 **1s**	220–260 (10 °C/min)	3.90
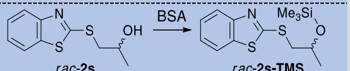 *rac*-**2s** → *rac*-**2s-TMS**		3.89 or 3.34 (silylated derivative)
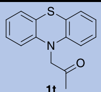 **1t**	260 (const.)	5.57
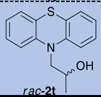 *rac*-**2t**		5.08
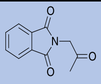 **1u**	220–260 (10 °C/min)	2.80
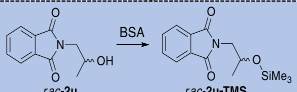 *rac*-**2u** → *rac*-**2u-TMS**		2.69 or 2.37 (silylated derivative)
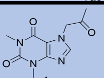 **1v**	260 (const.)	3.95
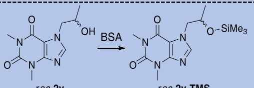 *rac*-**2v** → *rac*-**2v-TMS**		4.01 or 2.64 (silylated derivative)
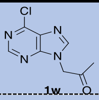 **1w**	260 (const.)	2.67
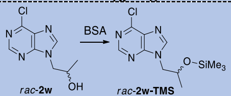 *rac*-**2w** → *rac*-**2w-TMS**		2.81 or 1.99 (silylated derivative)
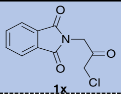 **1x**	220–260 (10 °C/min)	4.04
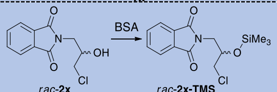 *rac*-**2x** → *rac*-**2x-TMS**		4.25 or 3.67 (silylated derivative)
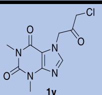 **1y**	260 (const.)	6.41
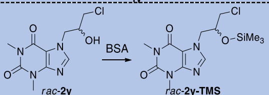 *rac*-**2y** → *rac*-**2y-TMS**		6.56 or 4.39 (silylated derivative)
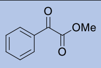 **1z**	120–260 (10 °C/min)	4.24
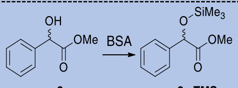 *rac*-**2z** → *rac*-**2z-TMS**		4.17 or 5.13 (silylated derivative)
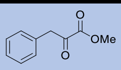 **1aa**	120–260 (10 °C/min)	4.79
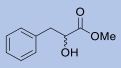 *rac*-**2aa**		4.91

Supplementary Table 11. Analytical separation conditions of studied compounds by GC (cont.).

Compound	Temperature program [°C]	Retention time [min]
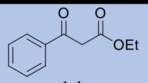 **1ab**	150–260 (10 °C/min)	Decomp: 4.31, 3.35, 1.63, 1.41
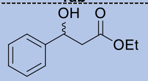 *rac*-**2ab**		3.92
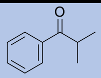 **1ac**	120–260 (10 °C/min)	2.85
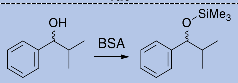 *rac*-**2ac** *rac*-**2ac-TMS**		2.82 or 2.25 (silylated derivative)
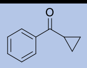 **1ad**	120–260 (10 °C/min)	4.02
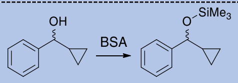 *rac*-**2ad** *rac*-**2ad-TMS**		3.98 or 3.23 (silylated derivative)
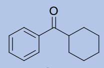 **1ae**	150–260 (10 °C/min)	4.92
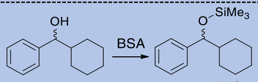 *rac*-**2ae** *rac*-**2ae-TMS**		4.92 or 3.73 (silylated derivative)
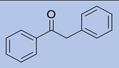 **1af**	150–260 (10 °C/min)	6.85
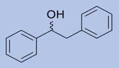 *rac*-**2af**		6.34
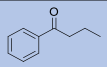 **1ag**	120–260 (10 °C/min)	3.34
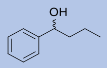 *rac*-**2ag**		3.17
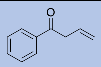 **1ah**	120–260 (10 °C/min)	3.43
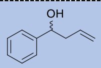 *rac*-**2ah**		3.15

Supplementary Table 12. HPLC analytical separation conditions of racemates by chiral columns.

Compound	HPLC Column	Mobile Phase<br>*n*-Hexane/IPA/DEA [v/v/v][a]	Flow Rate [mL/min] / Pressure [MPa]	Detection [nm] / Temperature [°C]	Retention Time [min]
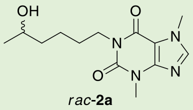<br>*rac*-**2a**	Chiralpak AD-H	78:22:0.1	1.0 / 5.5	273 / 25	31.082 (*R*) and 34.118 (*S*)
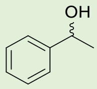<br>*rac*-**2b**	Chiralcel OD-H	97:3:0	1.0 / 4.4	210 / 30	10.839 (*R*) and 12.638 (*S*)
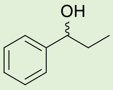<br>*rac*-**2c**	Chiralcel OD-H	98:2:0	1.0 / 4.4	220 / 30	12.738 (*R*) and 13.633 (*S*)
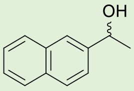<br>*rac*-**2d**	Chiralcel OJ-H	90:10:0	1.0 / 4.5	222 / 30	13.786 (*S*) and 17.834 (*R*)
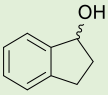<br>*rac*-**2e**	Chiralcel OD-H	98:2:0	1.0 / 4.4	205 / 30	15.539 (*S*) and 17.650 (*R*)
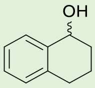<br>*rac*-**2f**	Chiralcel OJ-H	90:10:0	0.8 / 3.6	220 / 30	7.871 (*S*) and 9.170 (*R*)
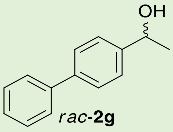<br>*rac*-**2g**	Chiralcel OD-H	98:2:0	0.8 / 3.5	210 / 30	34.797 (*S*) and 37.752 (*R*)
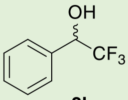<br>*rac*-**2h**	Chiralcel OD-H	95:5:0	0.9 / 4.1	210 / 30	12.395 (*R*) and 13.249 (*S*)
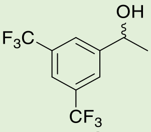<br>*rac*-**2i**	Chiralcel OD-H	98:2:0	0.8 / 3.5	210 / 30	8.737(*S*) and 9.774 (*R*)
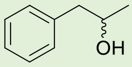<br>*rac*-**2j**	Chiralcel OD-H	99:1:0	0.8 / 3.5	205 / 30	17.631 (*S*) and 19.877 (*R*)

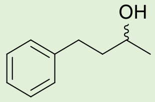 *rac*-**2k**	Chiralcel OD-H	90:10:0	0.8 / 3.7	210 / 30	7.799 (*R*) and 10.264 (*S*)
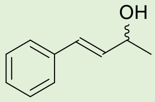 *rac*-**2l**	Chiralcel OD-H	95:5:0	0.9 / 4.1	210 / 30	15.259 (*R*) and 24.625 (*S*)
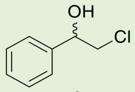 *rac*-**2m**	Chiralcel OD-H	98:2:0	1.0 / 4.4	220 / 30	18.214 (*R*) and 20.209 (*S*)
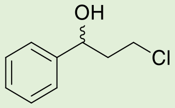 *rac*-**2n**	Chiralcel OD-H	95:5:0	0.9 / 4.1	210 / 30	11.156 (*R*) and 13.505 (*S*)
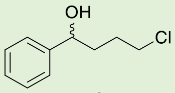 *rac*-**2o**	Chiralcel OD-H	98:2:0	1.0 / 4.4	210 / 30	23.391 (*R*) and 26.377 (*S*)
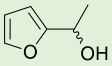 *rac*-**2p**	Chiralcel OJ-H	95:5:0	0.8 / 3.4	220 / 30	11.332 (*S*) and 12.586 (*R*)
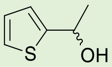 *rac*-**2q**	Chiralcel OJ-H	95:5:0	0.8 / 3.4	233 / 30	13.836 (*S*) and 17.395 (*R*)
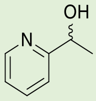 *rac*-**2r**	Lux i-Cellulose 5[b]	99:1:0	1.0 / 3.7	258 / 30	43.482 (*S*) and 46.830 (*R*)
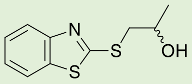 *rac*-**2s**	Chiralcel OD-H	95:5:0	0.7 / 3.1	225 / 30	22.202 (*R*) and 24.051 (*S*)
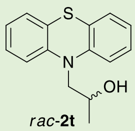 *rac*-**2t**	Chiralcel OD-H	90:10:0	0.8 / 3.7	254 / 30	8.650 (*R*) and 9.501 (*S*)
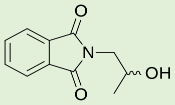 *rac*-**2u**	Chiralcel OD-H	90:10:0	0.8 / 3.7	216 / 30	17.310 (*R*) and 18.748 (*S*)
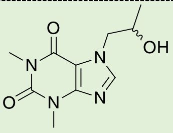 *rac*-**2v**	Chiralpak AD-H	78:22:0	1.0 / 5.5	273 / 25	7.621 (*S*) and 8.877 (*R*)

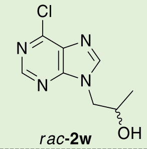 *rac*-**2w**	Chiralcel OJ-H	90:10:0	1.0 / 4.5	264 / 30	16.210 (*S*) and 17.868 (*R*)
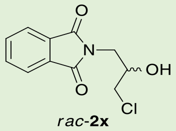 *rac*-**2x**	Chiralcel OD-H	90:10:0	0.8 / 3.7	220 / 30	22.862 (*S*) and 30.254 (*R*)
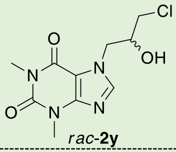 *rac*-**2y**	Chiralpak AD-H	78:22:0	0.3 / 1.6	273 / 25	35.534 (*R*) and 38.908 (*S*)
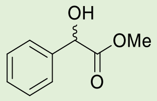 *rac*-**2z**	Chiralcel OD-H	90:10:0	0.8 / 3.7	225 / 30	9.213 (*S*) and 14.990 (*R*)
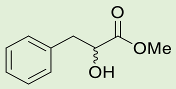 *rac*-**2aa**	Chiralcel OJ-H	90:10:0	0.5 / 2.2	210 / 30	26.551 (*R*) and 29.606 (*S*)
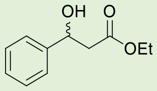 *rac*-**2ab**	Chiralcel OD-H	90:10:0	0.8 / 3.7	210 / 30	9.150 (*S*) and 11.599 (*R*)
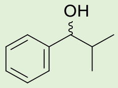 *rac*-**2ac**	Chiralcel OD-H	98:2:0	1.0 / 4.4	210 / 30	9.755 (*S*) and 12.093 (*R*)
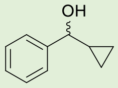 *rac*-**2ad**	Chiralcel OJ-H	98:2:0	1.0 / 4.3	204 / 30	21.053 (*S*) and 24.549 (*R*)
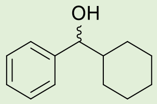 *rac*-**2ae**	Chiralcel OD-H	95:5:0	0.9 / 4.1	210 / 30	7.461 (*S*) and 9.032 (*R*)
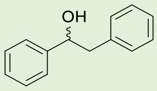 *rac*-**2af**	Chiralcel OD-H	95:5:0	0.9 / 4.1	210 / 30	12.139 (*R*) and 13.611 (*S*)
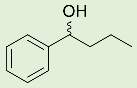 *rac*-**2ag**	Chiralcel OJ-H	97:3:0	1.0 / 4.3	220 / 30	8.585 (*S*) and 9.251 (*R*)
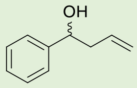 *rac*-**2ah**	Chiralcel OD-H	98:2:0	1.0 / 4.4	220 / 30	12.694 (*S*) and 13.864 (*R*)

[a] IPA states for 2-PrOH (propan-2-ol); DEA states for diethylamine. [b] Sample was dissolved in EtOAc.

Supplementary Table 13. Determination of the absolute configuration based on the elution order of the HPLC peaks separated by chiral columns.

Compound	HPLC Column	The elution order of the HPLC peaks	Literature reference or analytical standard
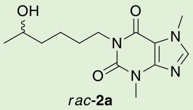 *rac*-**2a**	Chiralpak AD-H	1st peak: (*R*)-enantiomer<br>2nd peak: (*S*)-enantiomer	[6]
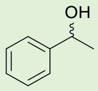 *rac*-**2b**	Chiralcel OD-H	1st peak: (*R*)-enantiomer<br>2nd peak: (*S*)-enantiomer	See the HPLC chromatogram recorded for (*S*)-**2b** as an analytical standard
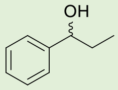 *rac*-**2c**	Chiralcel OD-H	1st peak: (*R*)-enantiomer<br>2nd peak: (*S*)-enantiomer	See the HPLC chromatogram recorded for (*S*)-**2c** as an analytical standard
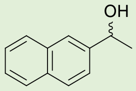 *rac*-**2d**	Chiralcel OJ-H	1st peak: (*S*)-enantiomer<br>2nd peak: (*R*)-enantiomer	See the HPLC chromatogram recorded for (*S*)-**2d** as an analytical standard
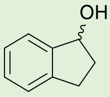 *rac*-**2e**	Chiralcel OD-H	1st peak: (*S*)-enantiomer<br>2nd peak: (*R*)-enantiomer	See the HPLC chromatogram recorded for (*R*)-**2e** as an analytical standard
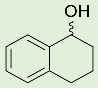 *rac*-**2f**	Chiralcel OJ-H	1st peak: (*S*)-enantiomer<br>2nd peak: (*R*)-enantiomer	See the HPLC chromatogram recorded for (*R*)-**2f** as an analytical standard
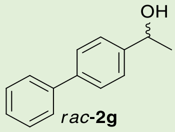 *rac*-**2g**	Chiralcel OD-H	1st peak: (*S*)-enantiomer<br>2nd peak: (*R*)-enantiomer	[7]
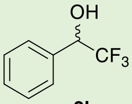 *rac*-**2h**	Chiralcel OD-H	1st peak: (*R*)-enantiomer<br>2nd peak: (*S*)-enantiomer	[8]
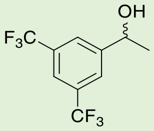 *rac*-**2i**	Chiralcel OD-H	1st peak: (*S*)-enantiomer<br>2nd peak: (*R*)-enantiomer	[9]
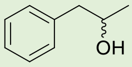 *rac*-**2j**	Chiralcel OD-H	1st peak: (*S*)-enantiomer<br>2nd peak: (*R*)-enantiomer	See the HPLC chromatogram recorded for (*R*)-**2j** as an analytical standard

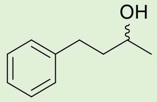<br>*rac*-**2k**	Chiralcel OD-H	1st peak: (*R*)-enantiomer<br>2nd peak: (*S*)-enantiomer	See the HPLC chromatogram recorded for (*R*)-**2k** as an analytical standard
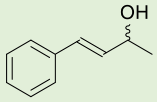<br>*rac*-**2l**	Chiralcel OD-H	1st peak: (*R*)-enantiomer<br>2nd peak: (*S*)-enantiomer	[10]
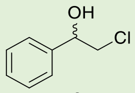<br>*rac*-**2m**	Chiralcel OD-H	1st peak: (*R*)-enantiomer<br>2nd peak: (*S*)-enantiomer	[8]
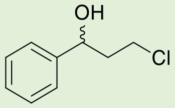<br>*rac*-**2n**	Chiralcel OD-H	1st peak: (*R*)-enantiomer<br>2nd peak: (*S*)-enantiomer	[8]
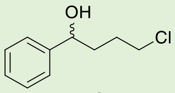<br>*rac*-**2o**	Chiralcel OD-H	1st peak: (*R*)-enantiomer<br>2nd peak: (*S*)-enantiomer	[8]
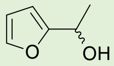<br>*rac*-**2p**	Chiralcel OJ-H	1st peak: (*S*)-enantiomer<br>2nd peak: (*R*)-enantiomer	See the HPLC chromatogram recorded for (*R*)-**2p** as an analytical standard
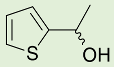<br>*rac*-**2q**	Chiralcel OJ-H	1st peak: (*S*)-enantiomer<br>2nd peak: (*R*)-enantiomer	[9]
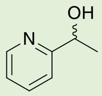<br>*rac*-**2r**	Lux i-Cellulose 5	1st peak: (*S*)-enantiomer<br>2nd peak: (*R*)-enantiomer	[11]
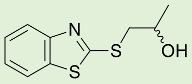<br>*rac*-**2s**	Chiralcel OD-H	1st peak: (*R*)-enantiomer<br>2nd peak: (*S*)-enantiomer	[12]
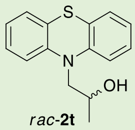<br>*rac*-**2t**	Chiralcel OD-H	1st peak: (*R*)-enantiomer<br>2nd peak: (*S*)-enantiomer	[13]
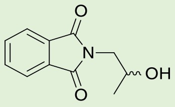<br>*rac*-**2u**	Chiralcel OD-H	1st peak: (*R*)-enantiomer<br>2nd peak: (*S*)-enantiomer	[14]
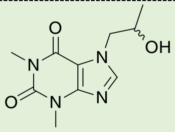<br>*rac*-**2v**	Chiralpak AD-H	1st peak: (*S*)-enantiomer<br>2nd peak: (*R*)-enantiomer	[15]

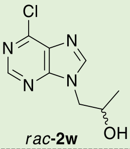 *rac*-**2w**	Chiralcel OJ-H	1st peak: (*S*)-enantiomer<br>2nd peak: (*R*)-enantiomer	[16]
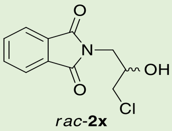 *rac*-**2x**	Chiralcel OD-H	1st peak: (*S*)-enantiomer<br>2nd peak: (*R*)-enantiomer	[17]
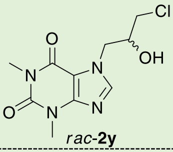 *rac*-**2y**	Chiralpak AD-H	1st peak: (*R*)-enantiomer<br>2nd peak: (*S*)-enantiomer	[15]
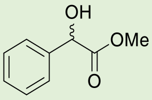 *rac*-**2z**	Chiralcel OD-H	1st peak: (*S*)-enantiomer<br>2nd peak: (*R*)-enantiomer	[18]
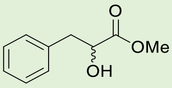 *rac*-**2aa**	Chiralcel OJ-H	1st peak: (*R*)-enantiomer<br>2nd peak: (*S*)-enantiomer	[19]
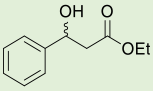 *rac*-**2ab**	Chiralcel OD-H	1st peak: (*S*)-enantiomer<br>2nd peak: (*R*)-enantiomer	[18]
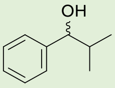 *rac*-**2ac**	Chiralcel OD-H	1st peak: (*S*)-enantiomer<br>2nd peak: (*R*)-enantiomer	[20]
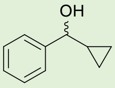 *rac*-**2ad**	Chiralcel OJ-H	1st peak: (*S*)-enantiomer<br>2nd peak: (*R*)-enantiomer	[21]
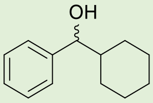 *rac*-**2ae**	Chiralcel OD-H	1st peak: (*S*)-enantiomer<br>2nd peak: (*R*)-enantiomer	[20]
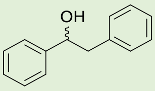 *rac*-**2af**	Chiralcel OD-H	1st peak: (*S*)-enantiomer<br>2nd peak: (*R*)-enantiomer	[22 or 23]
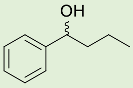 *rac*-**2ag**	Chiralcel OJ-H	1st peak: (*R*)-enantiomer<br>2nd peak: (*S*)-enantiomer	See the HPLC chromatogram recorded for (*R*)-**2ag** as an analytical standard
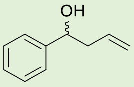 *rac*-**2ah**	Chiralcel OD-H	1st peak: (*S*)-enantiomer<br>2nd peak: (*R*)-enantiomer	[24]

Supplementary Table 14. The origin of the chemicals used in this study.

Compound	Manufacturer	Cat. Number
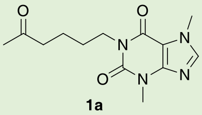 1a	Sigma-Aldrich	P1784
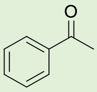 1b	Sigma-Aldrich	P51605
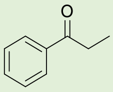 1c	Sigma-Aldrich	P51605
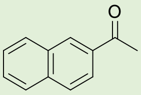 1d	Sigma-Aldrich	134775
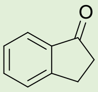 1e	Sigma-Aldrich	I2304
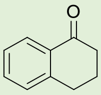 1f	Sigma-Aldrich	T19003
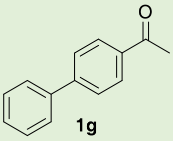 1g	TCI	A1025
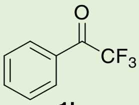 1h	Acros Organics	148350050
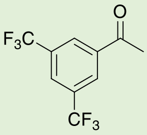 1i	AmBeed	A107409
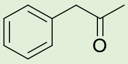 1j	MERCK Schuchardt	801523
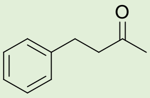 1k	Sigma-Aldrich	B16003

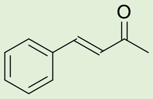 **1l**	Sigma-Aldrich	147885
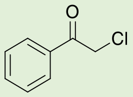 **1m**	Sigma-Aldrich	C19686
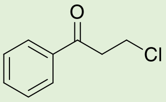 **1n**	Sigma-Aldrich	335614
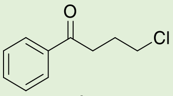 **1o**	Sigma-Aldrich	363448
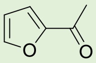 **1p**	Sigma-Aldrich	A16254
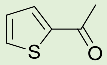 **1q**	Sigma-Aldrich	A22602
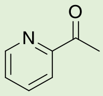 **1r**	Sigma-Aldrich	A21002
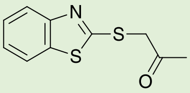 **1s**	AmBeed	A723024
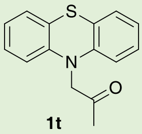 **1t**	Biosynth	FM25924
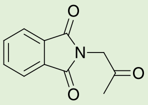 **1u**	BLD Pharmatech	BL3H160B9574
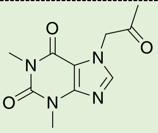 **1v**	Sigma-Aldrich	R865796
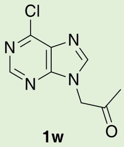 **1w**	Enamine	BBV-51271384

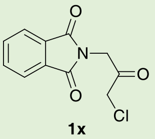 **1x**	Sigma-Aldrich	S242225
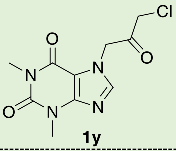 **1y**	[A]	[A]
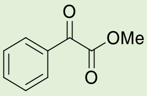 **1z**	Sigma-Aldrich	M30507
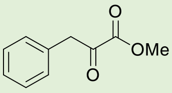 **1aa**	AmBeed	A515597
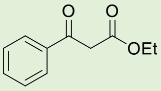 **1ab**	Alfa Aesar	L05030
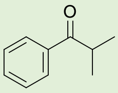 **1ac**	TCI	I0113
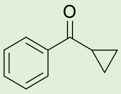 **1ad**	AmBeed	A642851
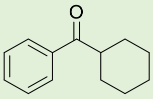 **1ae**	TCI	C1345
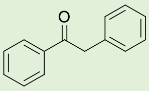 **1af**	TCI	B0435
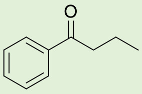 **1ag**	Sigma-Aldrich	124338
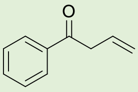 **1ah**	AmBeed	A1135421

[A] Synthesized according to the method reported by us previously (see Ref. [15]).

7. Supplementary references.

[1] Trott, O.; Olson, A. J. *J. Comput. Chem.* **2010**, *31*, 455–461.

[2] Noey, E. L.; Tibrewal, N.; Jiménez-Osés, G.; Osuna, S.; Park, J.; Bond, C. M.; Cascio, D.; Liang, J.; Zhang, X.; Huisman, G. W.; Tang, Y.; Houk, K. N.*Proceed. Nat. Acad. Sci.* **2015**, *112*, E7065–E7072.

[3] Pettersen, E. F.; Goddard, T. D.; Huang, C. C.; Couch, G. S.; Greenblatt, D. M.; Meng, E. C.; Ferrin, T. E. *J. Comput. Chem.* **2004**, *25*, 1605−1612.

[4] Lountos, G. T.; Austin, B. P.; Nallamsetty, S.; Waugh, D. S. *Protein. Sci.* **2009**, *18*, 467–474.

[5] Jurcik, A.; Bednar, D.; Byska, J.; Marques, S. M.; Furmanova, K.; Daniel, L.; Kokkonen, P.; Brezovsky, J.; Strnad, O.; Stourac, J.; Pavelka, A.; Manak, M.; Damborsky, J.; Kozlikova, B. *Bioinformatics* **2018**, *34*, 3586–3588.

[6] Borowiecki, P.; Rudzka, A.; Reiter, T.; Kroutil, W. *Catal. Sci. Technol.* **2022**, *12*, 4312–4324.

[7] Łowicki, D.; Bezłada, A.; Mlynarski, J. *Adv. Synth. Catal.* **2014**, *356*, 591–595.

[8] Xu, G.C.; Shang, Y.P.; Yu, H.L.; Xu, J.H. *Chem. Commun.* **2015**, *51*, 15728–15731.

[9] Tian, C.; Gong, L.; Meggers, E. *Chem. Commun.* **2016**, *52*, 4207–4210.

[10] Cheng, Y.N.; Wu, H.L.; Wu, P.Y.; Shen, Y.Y.; Uang, B.J. *Chem.–Asian J.* **2012**, *7*, 2921–2924.

[11] Zheng, L.S.; Llopis, Q.; Echeverria, P.G.; Ferard, C.; Guillamot, G.; Phansavath, P.; Ratovelomanana-Vidal, V. *J. Org. Chem.* **2017**, *82*, 5607–5615.

[12] Borowiecki, P.; Włoczewska, M.; Ochal, Z. *J. Mol. Catal. B: Enzym.* **2014**, *109*, 9–16.

[13] Borowiecki, P.; Paprocki, D.; Dranka, M. *Beilstein J. Org. Chem.* **2014**, *10*, 3038–3055.

[14] Xu, Z.; Li, Y.; Liu, J.; Wu, N.; Li, K.; Zhu, S.; Zhang, R.; Liu, Y. *Org. Biomol. Chem.* **2015**, *13*, 7513–7516.

[15] Borowiecki, P.; Rudzka, A.; Reiter, T.; Kroutil, W. *Bioorg. Chem.* **2022**, *127*, 105967.

[16] Zdun, B.; Reiter, T.; Kroutil, W.; Borowiecki, P. *J. Org. Chem.* **2023**, *88*, 11045−11055.

[17] Borowiecki, P.; Zdun, B.; Popow, N.; Wiklinska, M.; Reiter, T.; Kroutil, W. *RSC Adv.* **2022**, *12*, 22150–22160.

[18] Jiang, W.; Zhao, Q.; Tang, W. *Chinese J. Chem.* **2018**, *36*, 153–156.

[19] Chinthapally, K.; Baskaran, S. *Org. Biomol. Chem.* **2014**, *12*, 4305–4309.

[20] Ling, F.; Chen, J.; Nian, S.; Hou, H.; Yi, X.; Wu, F.; Xu, M.; Zhong, W. *Synlett* **2020**, *31*, 285–289.

[21] Zhang, Z.; Hu, X. *Angew. Chem.* **2021**, *60*, 22833–22838.

[22] Cao, W.; Tan, D.; Lee, R.; Tan, C.H. *J. Am. Chem. Soc.* **2018**, *140*, 1952–1955.

[23] Kitanosono, T.; Xu, P.; Kobayashi, S. *Chem.–Asian J.* **2014**, *9*, 179–188.

[24] Ghosh, D.; Sahu, D.; Saravanan, S.; Abdi, S.H.; Ganguly, B.; Khan, N.U.; Kureshy, R.I.; Bajaj, H.C. *Org. Biomol. Chem.* **2013**, *11*, 3451–3460.
