# Supplementary material for: Biocatalytic characterization of an alcohol dehydrogenase variant deduced from Lactobacillus kefir in asymmetric hydrogen transfer
Source: Commun Chem. 2023 Oct 12;6:217. doi: 10.1038/s42004-023-01013-1 (PMC10570314; doi:10.1038/s42004-023-01013-1)
Supplement: Supplementary file 2 — Description of Additional Supplementary Files [file 42004_2023_1013_MOESM2_ESM.pdf]

Description of Additional Supplementary Files

File name: Supplementary Data 1

Description: The copies of NMR, FTMS, and ATR-FTIR spectra for alcohols.

File name: Supplementary Data 2

Description: The list of the Cartesian coordinates for the optimized structure of 1-(6-chloro-9*H*-purin-9-yl)propan-2-one (**1w**).

File name: Supplementary Data 3

Description: Protein and DNA sequences of alcohol dehydrogenase variant deduced from Lactobacillus kefir (Lk-ADH Prince).

File name: Supplementary Data 4

Description: The copies of HPLC chromatograms.
