# Supplementary material for: Biocatalytic characterization of an alcohol dehydrogenase variant deduced from Lactobacillus kefir in asymmetric hydrogen transfer
Source: Commun Chem. 2023 Oct 12;6:217. doi: 10.1038/s42004-023-01013-1 (PMC10570314; doi:10.1038/s42004-023-01013-1)

Spectral data (copies of NMR, FTMS, and ATR-FTIR spectra)

1-(5-Hydroxyhexyl)-3,7-dimethyl-3,7-dihydro-1H-purine-2,6-dione (2a)

Supplementary Figure 3. ^{1}H NMR spectrum of **2a** (500 MHz, $CDCl_3$)

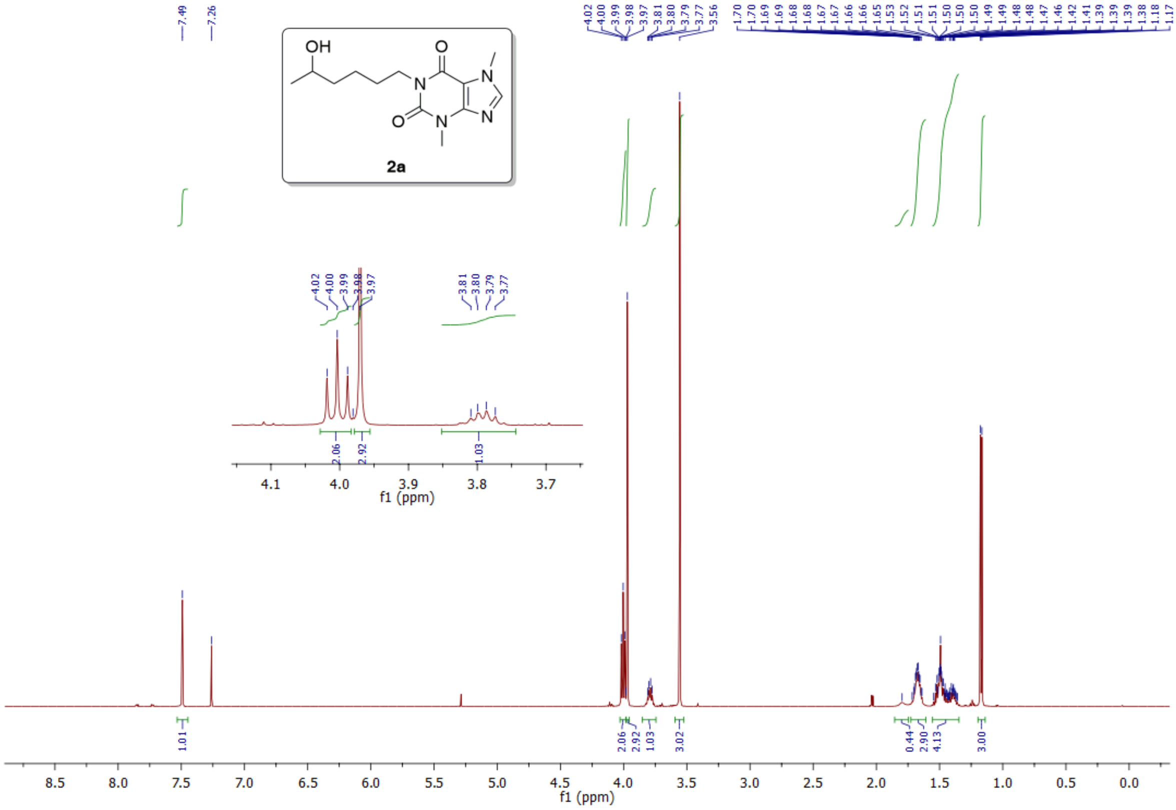

Supplementary Figure 4. $^{13}C\{^{1}H\}$ NMR spectrum of **2a** (126 MHz, $CDCl_3$)

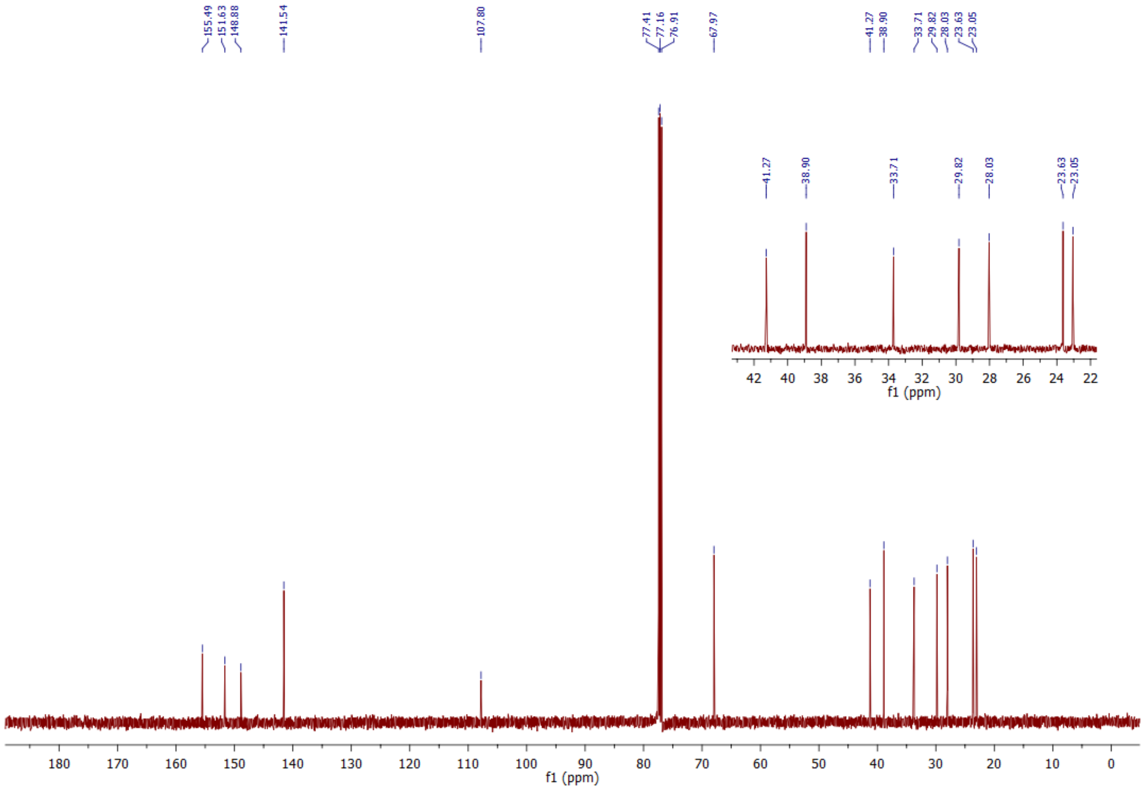

Supplementary Figure 5. FTMS spectrum of **2a** (ESI-TOF)

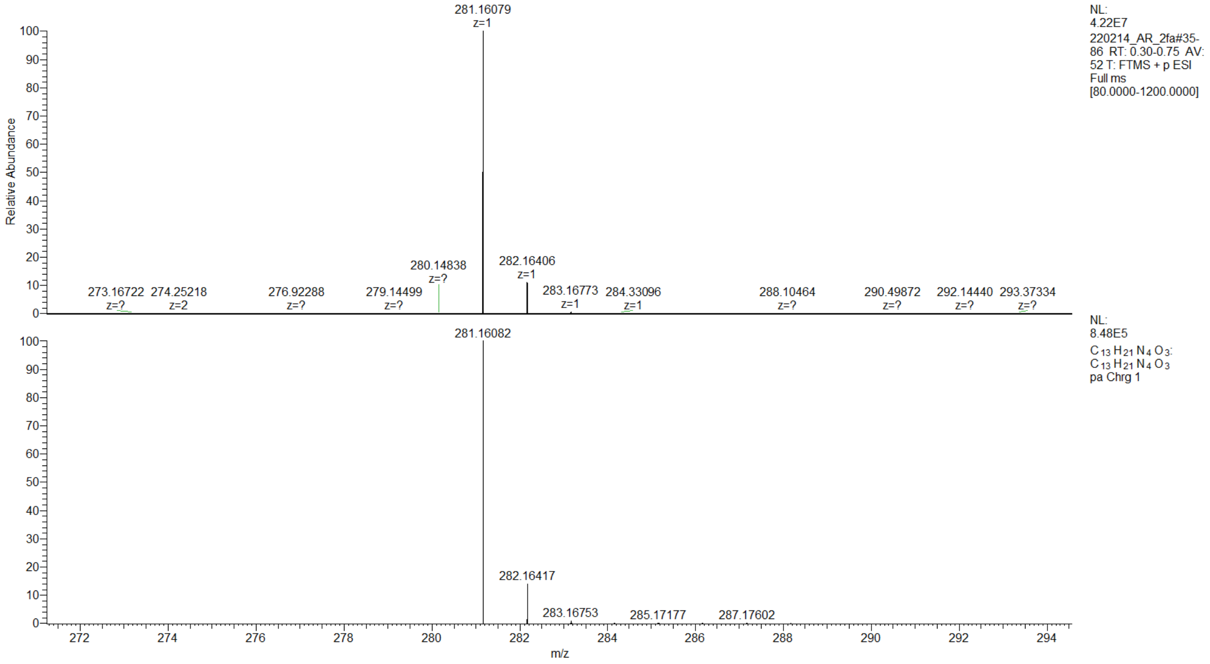

Supplementary Figure 6. ATR-FTIR spectrum of **2a** (neat)

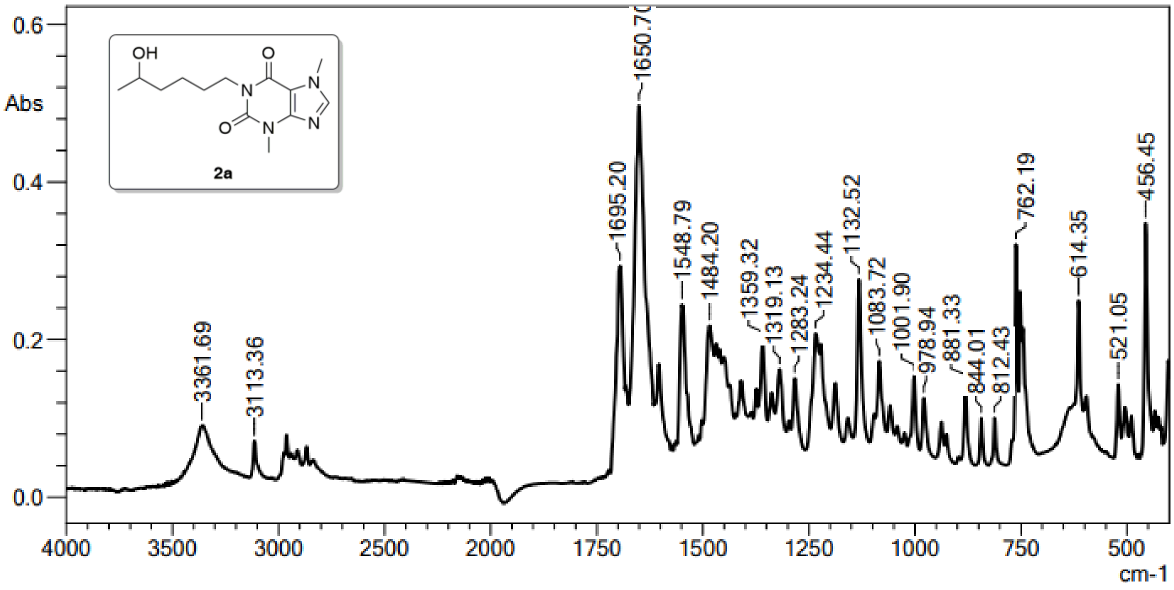

1-Phenylethanol (2b)

Supplementary Figure 7. ^{1}H NMR spectrum of **2b** (500 MHz, $CDCl_3$)

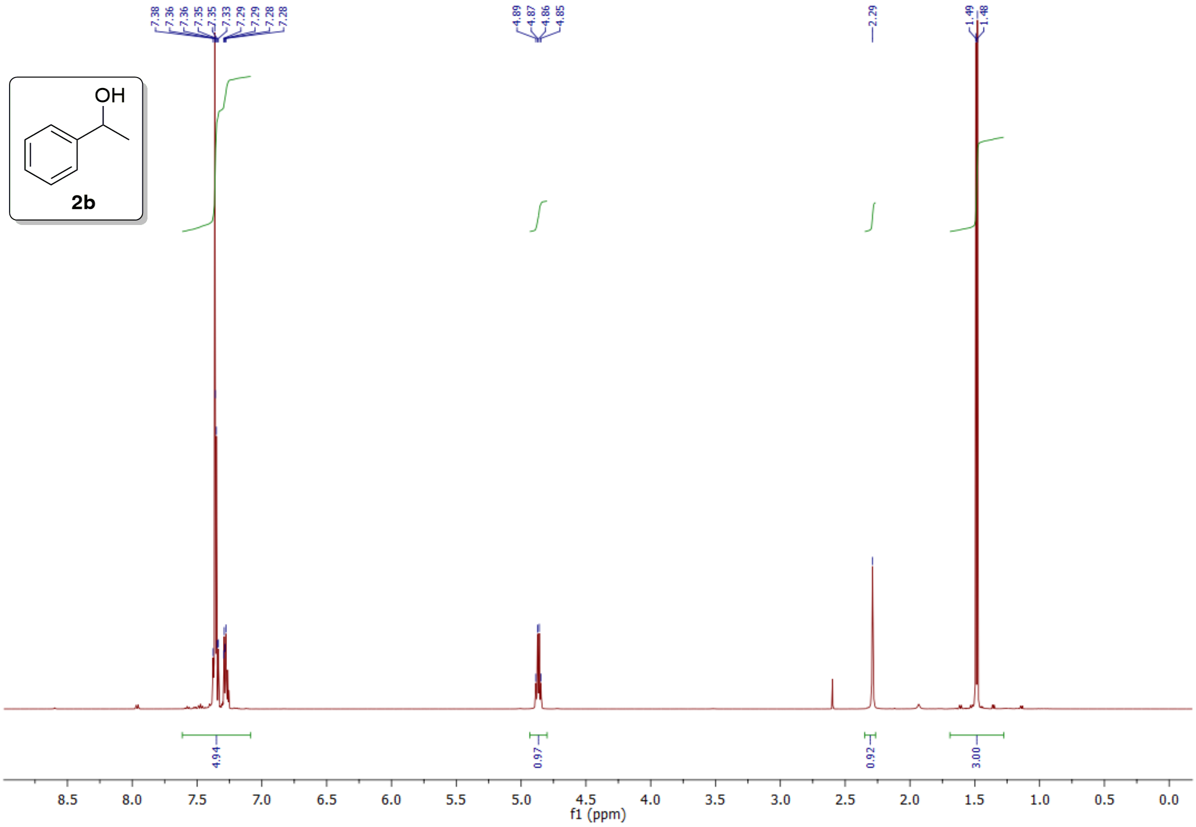

Supplementary Figure 8. $^{13}C\{^{1}H\}$ NMR spectrum of **2b** (126 MHz, $CDCl_3$)

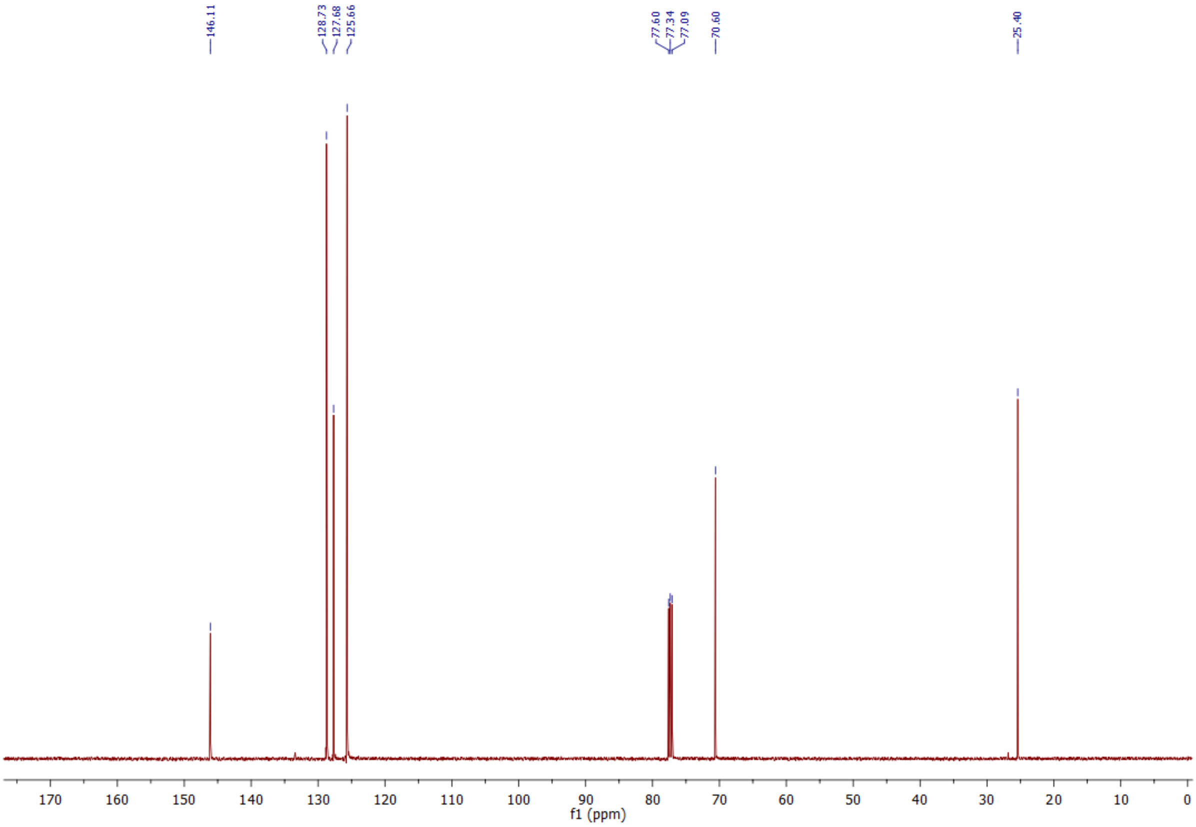

Supplementary Figure 9. FTMS spectrum of **2b** (ESI-TOF)

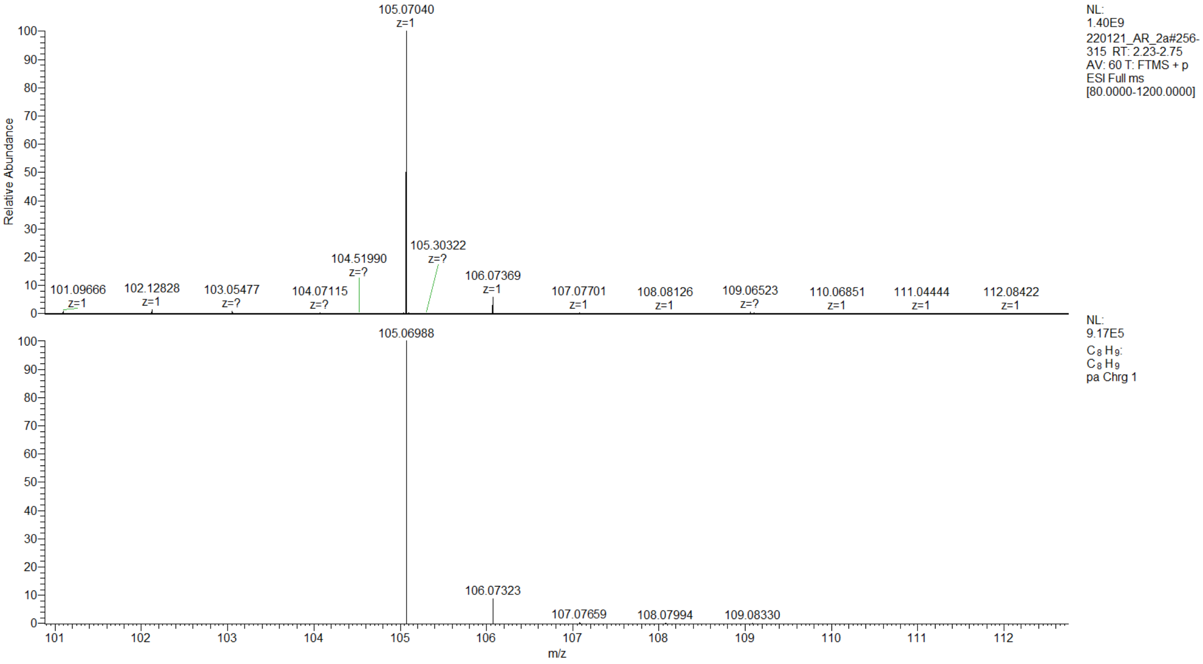

Supplementary Figure 10. ATR-FTIR spectrum of **2b** (neat)

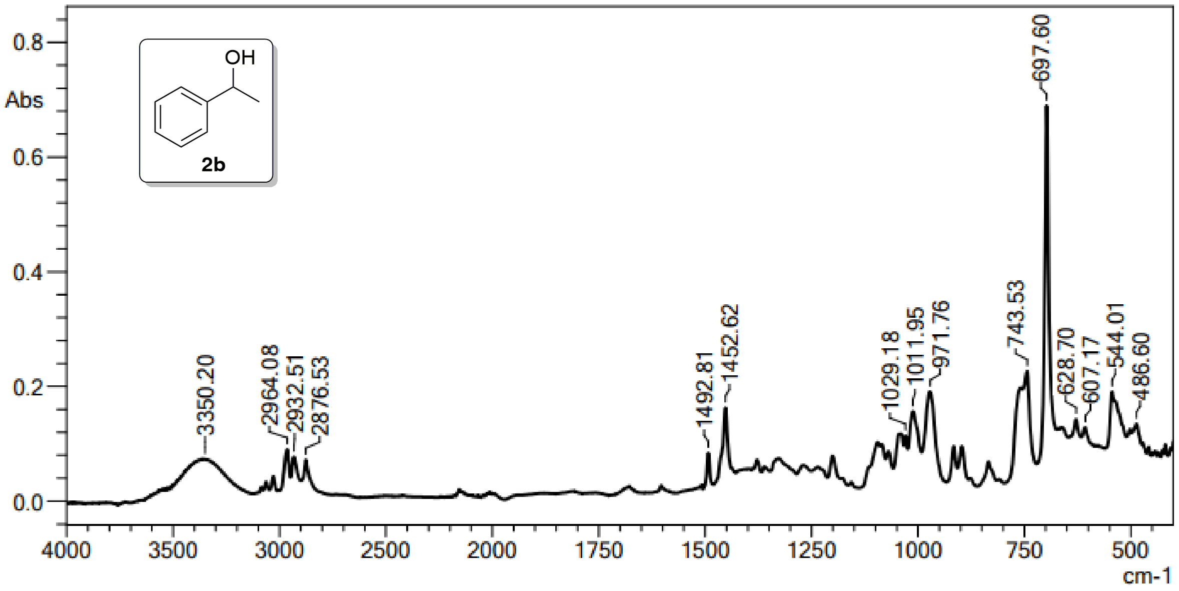

1-Phenylpropan-1-ol (2c)

Supplementary Figure 11. ^{1}H NMR spectrum of **2c** (500 MHz, $CDCl_3$)

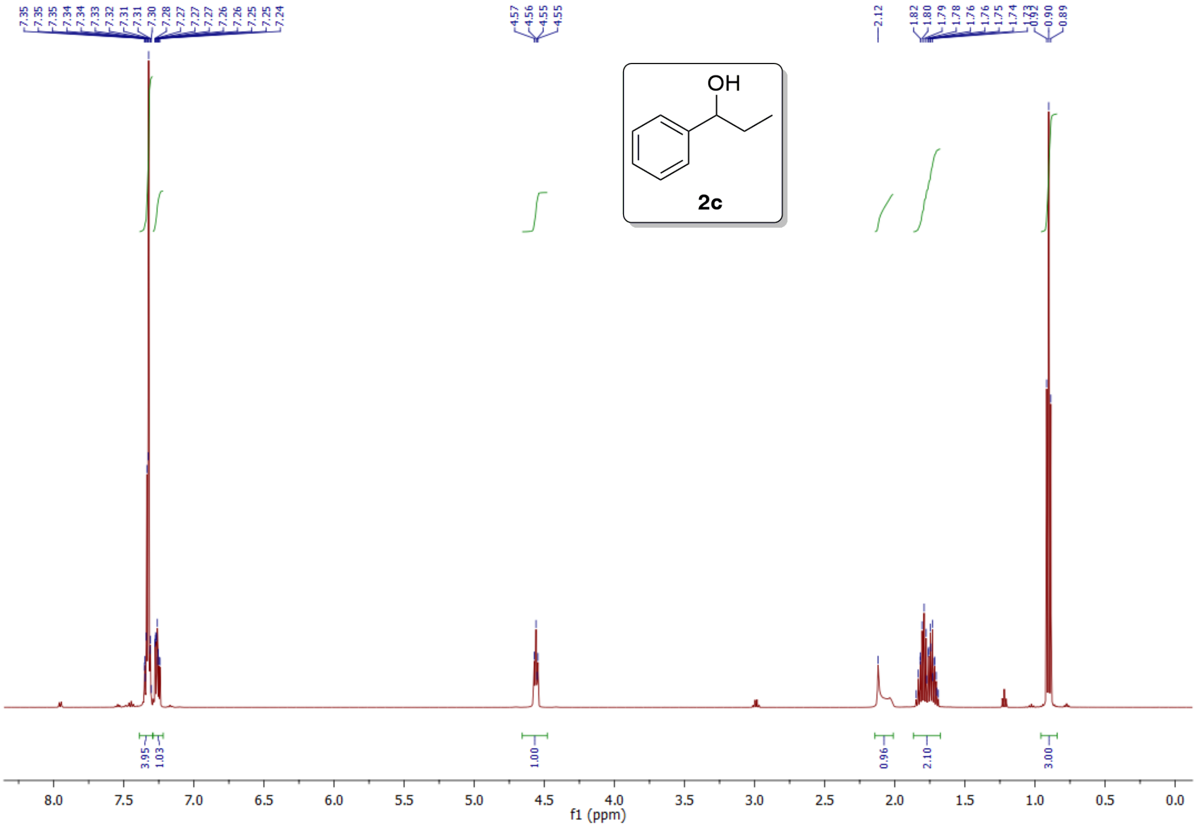

Supplementary Figure 12. $^{13}C\{^{1}H\}$ NMR spectrum of **2c** (126 MHz, $CDCl_3$)

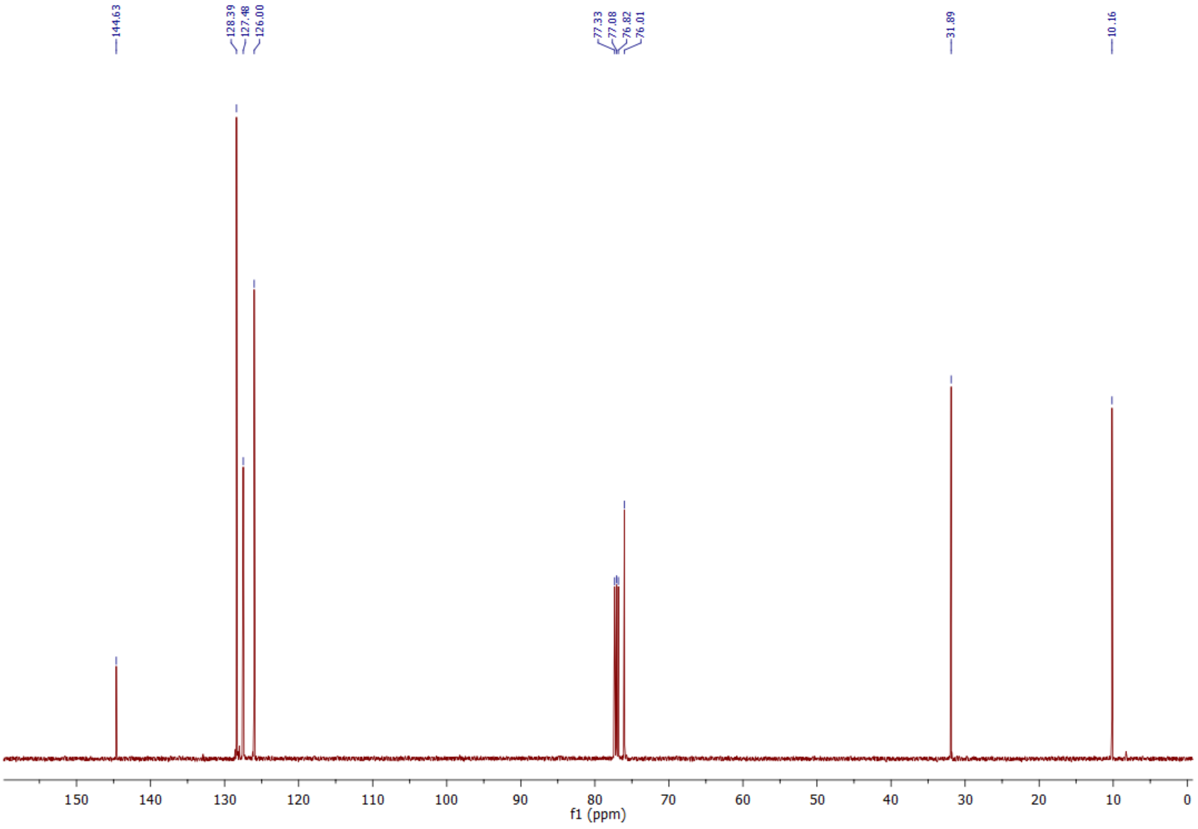

Supplementary Figure 13. FTMS spectrum of **2c** (ESI-TOF)

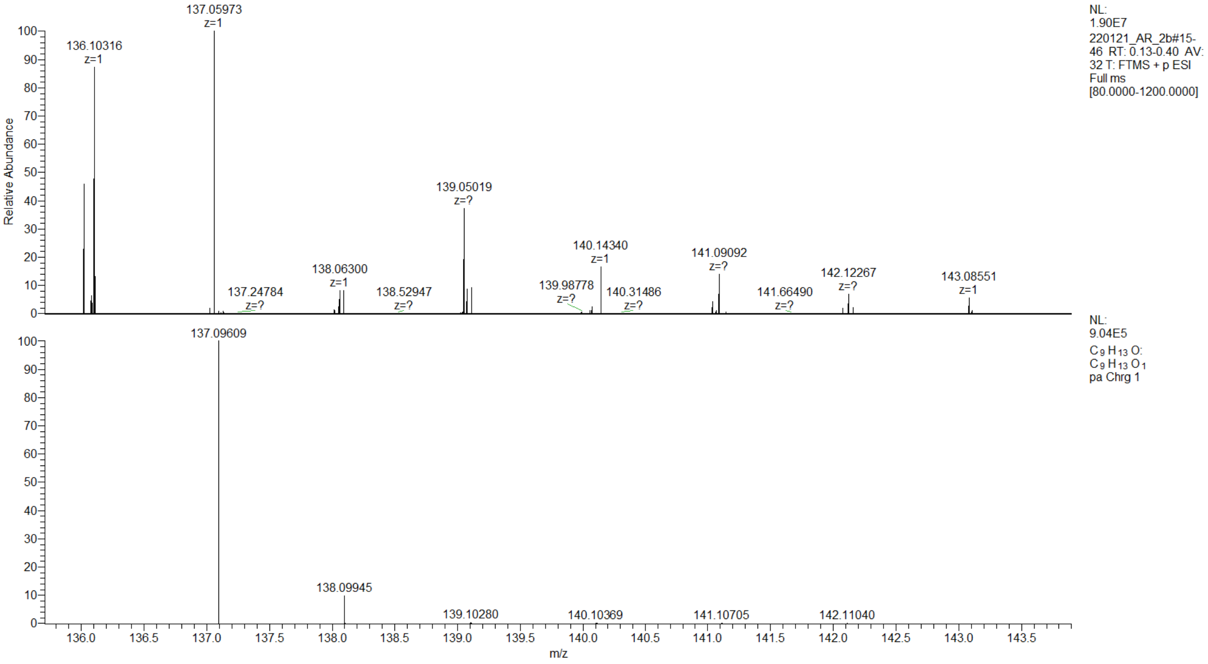

Supplementary Figure 14. ATR-FTIR spectrum of **2c** (neat)

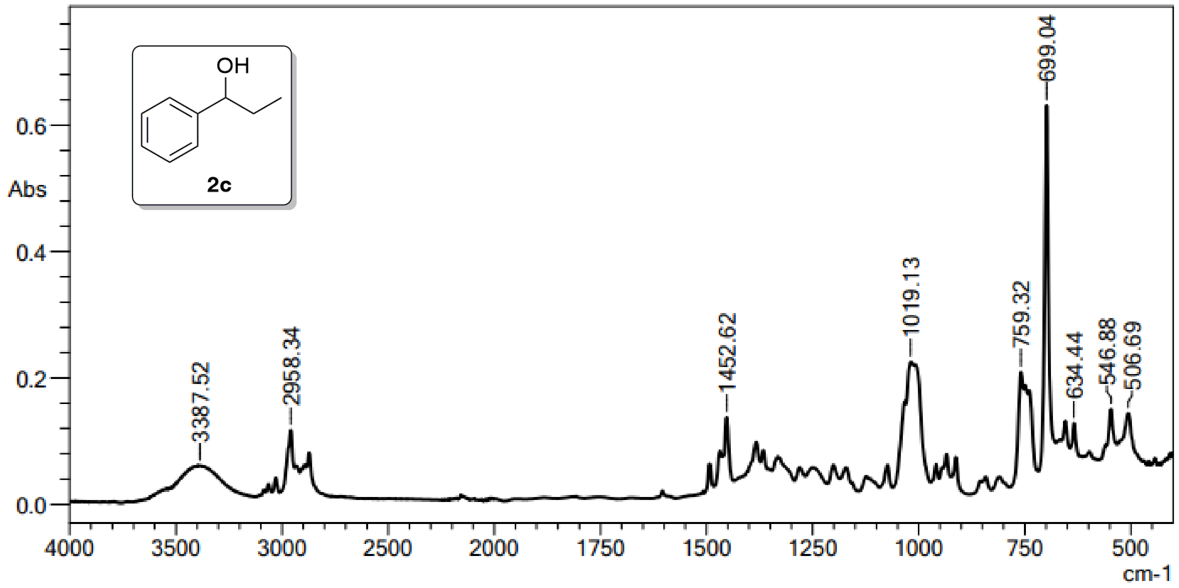

1-(Naphthalen-2-yl)ethanol (2d)

Supplementary Figure 15. ^{1}H NMR spectrum of **2d** (500 MHz, $CDCl_3$)

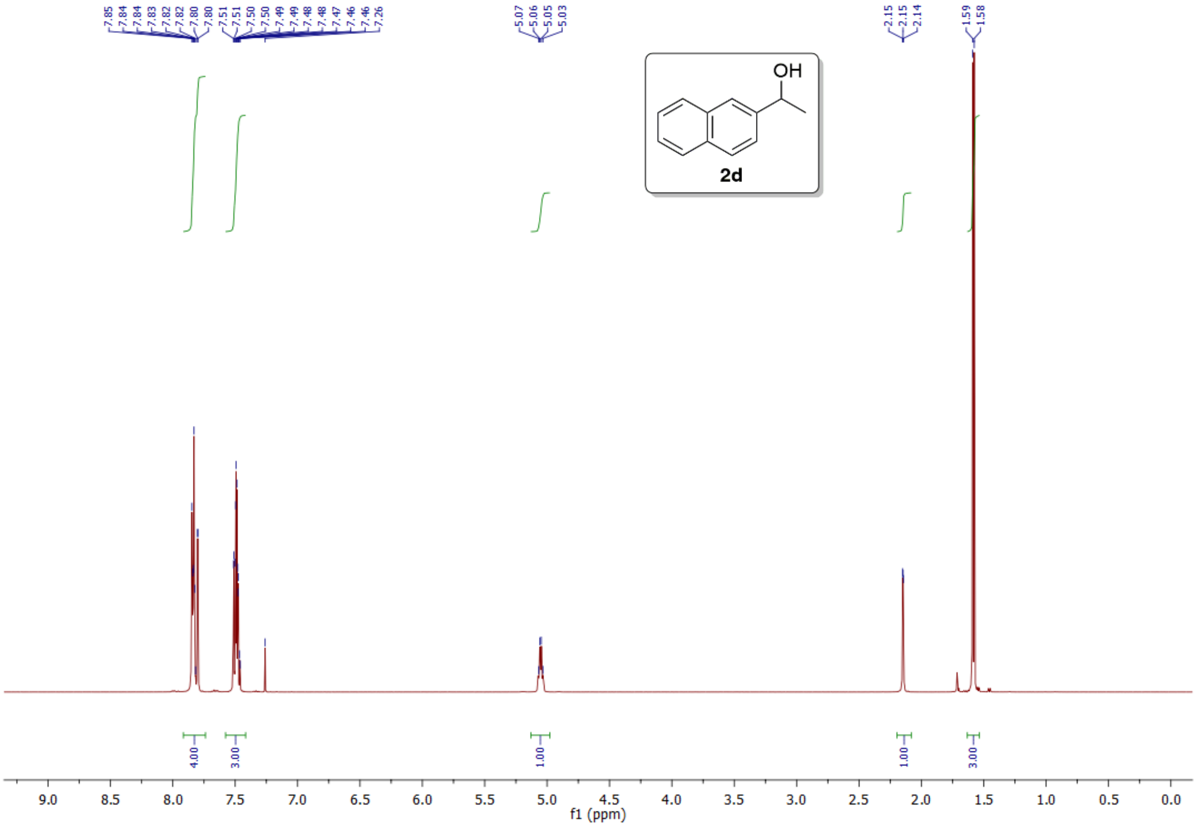

Supplementary Figure 16. $^{13}C\{^1H\}$ NMR spectrum of **2d** (126 MHz, $CDCl_3$)

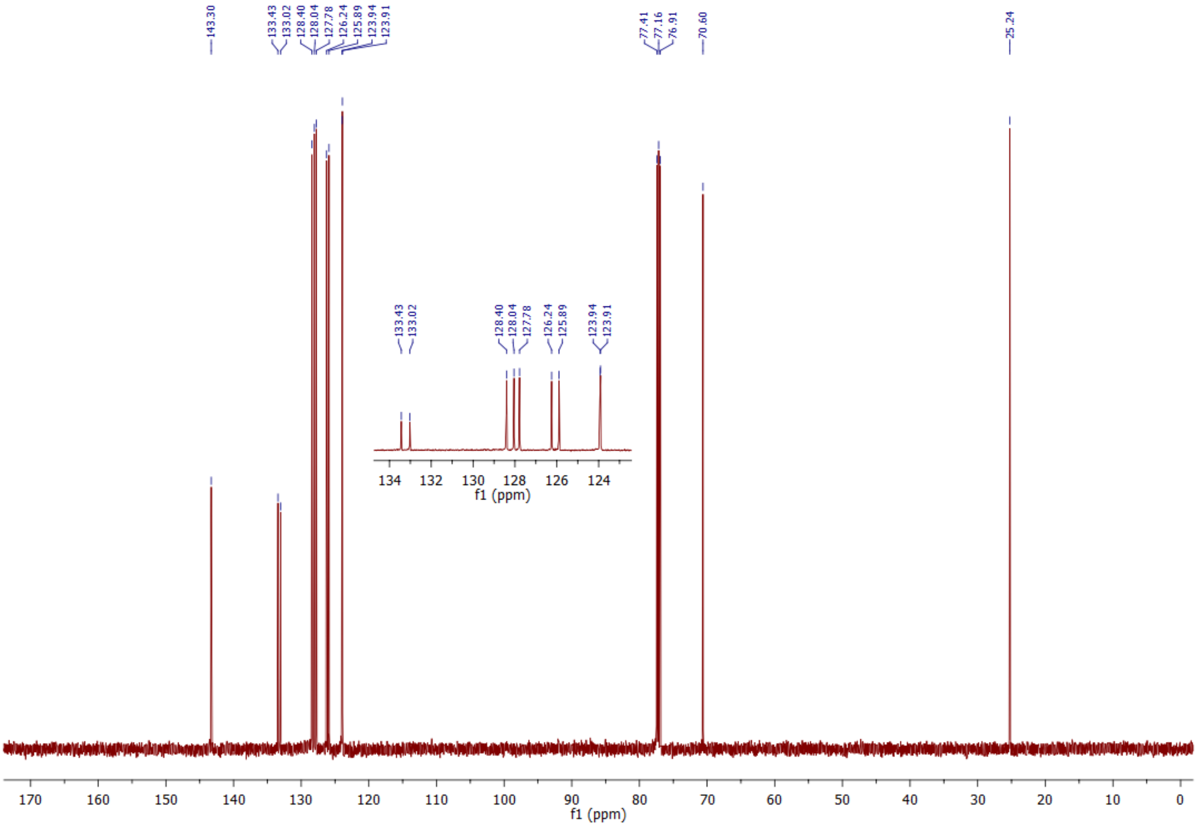

Supplementary Figure 17. FTMS spectrum of **2d** (ESI-TOF)

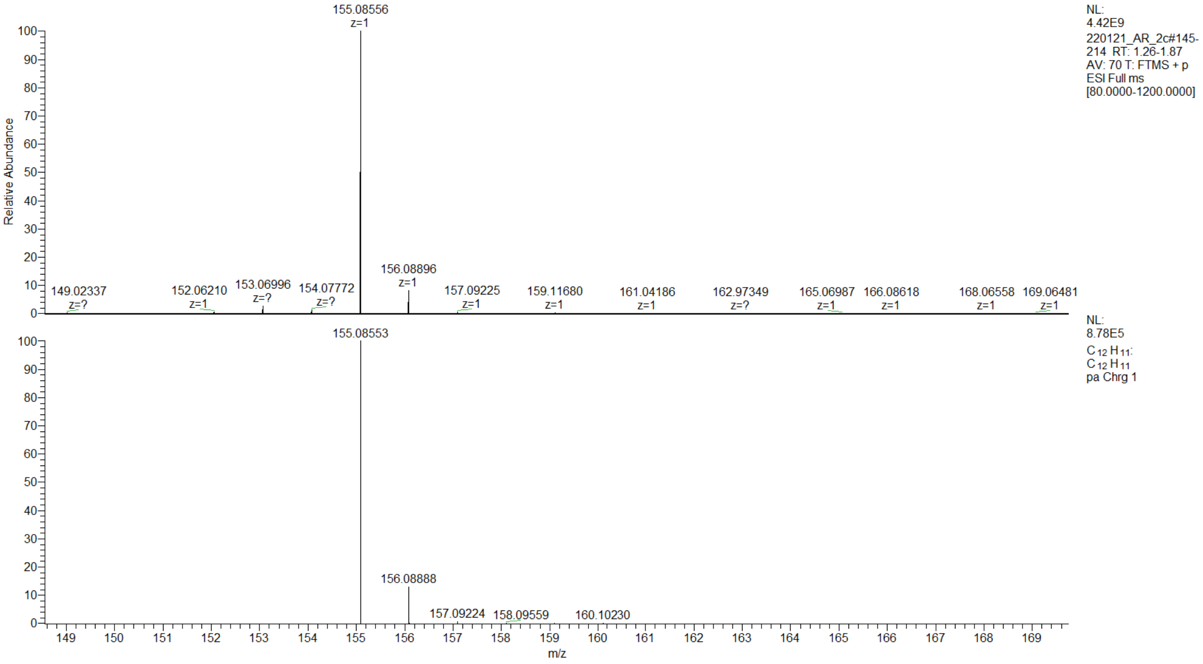

Supplementary Figure 18. ATR-FTIR spectrum of **2d** (neat)

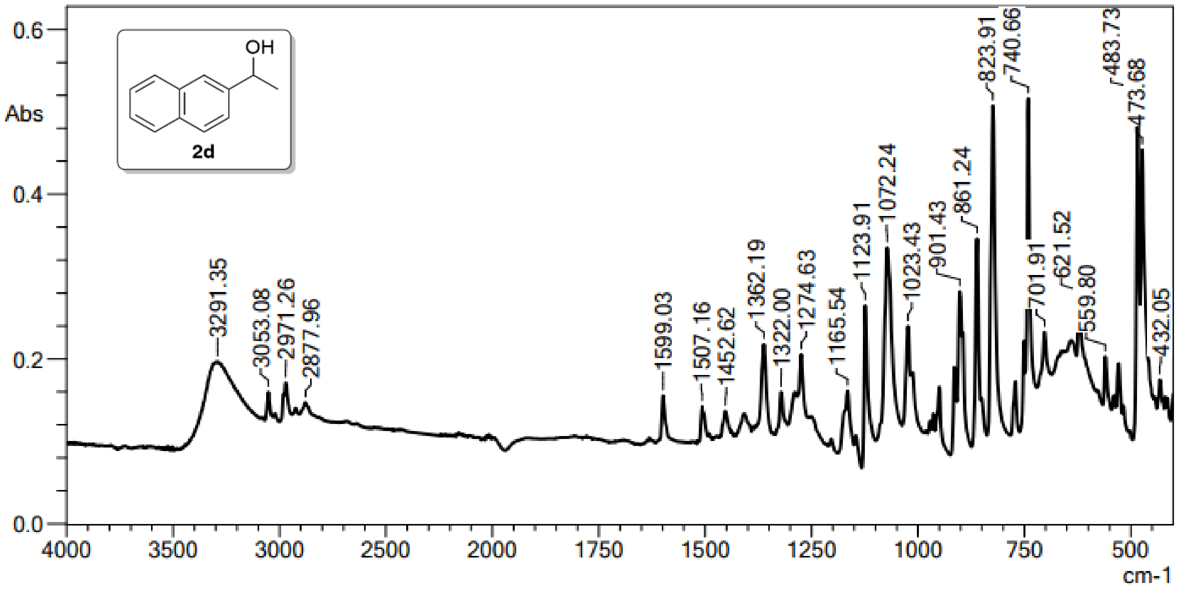

2,3-Dihydro-1H-inden-1-ol (2e)

Supplementary Figure 19. ^{1}H NMR spectrum of **2e** (500 MHz, $CDCl_3$)

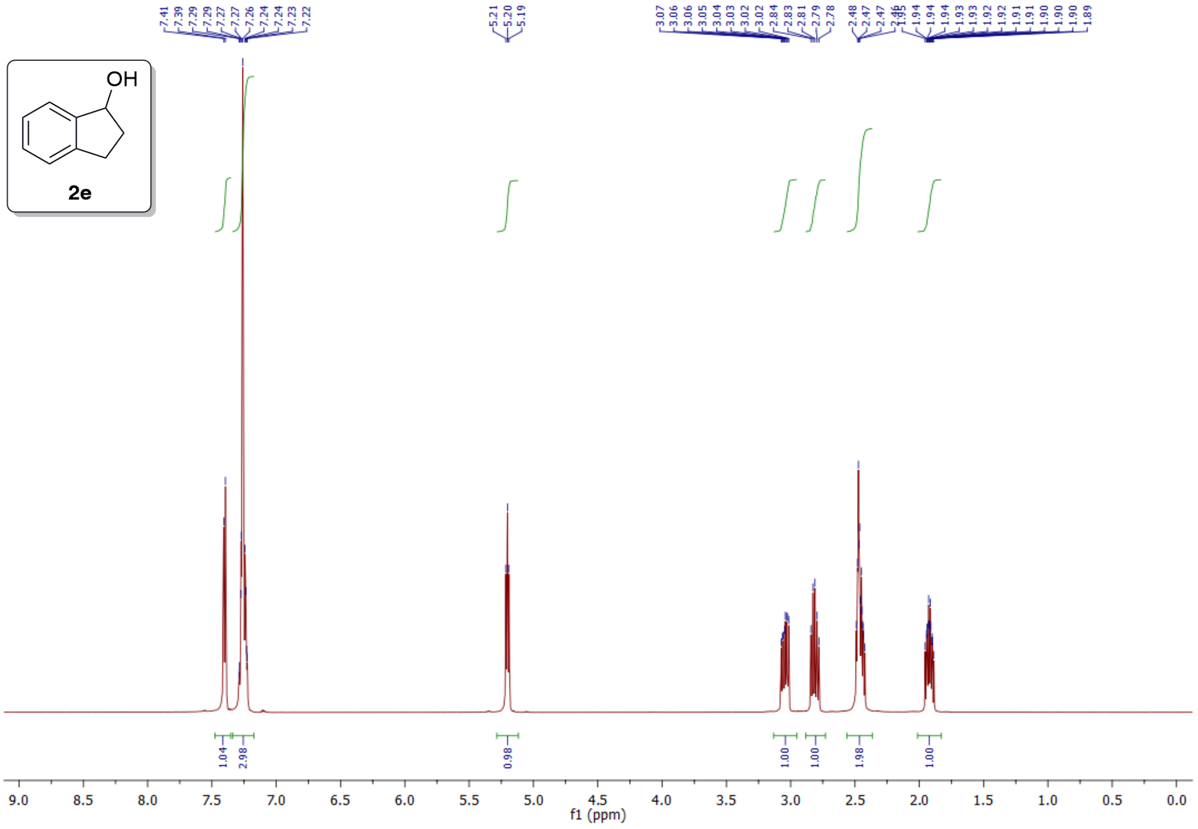

Supplementary Figure 20. ^{13}C{^{1}H} NMR spectrum of **2e** (126 MHz, $CDCl_3$)

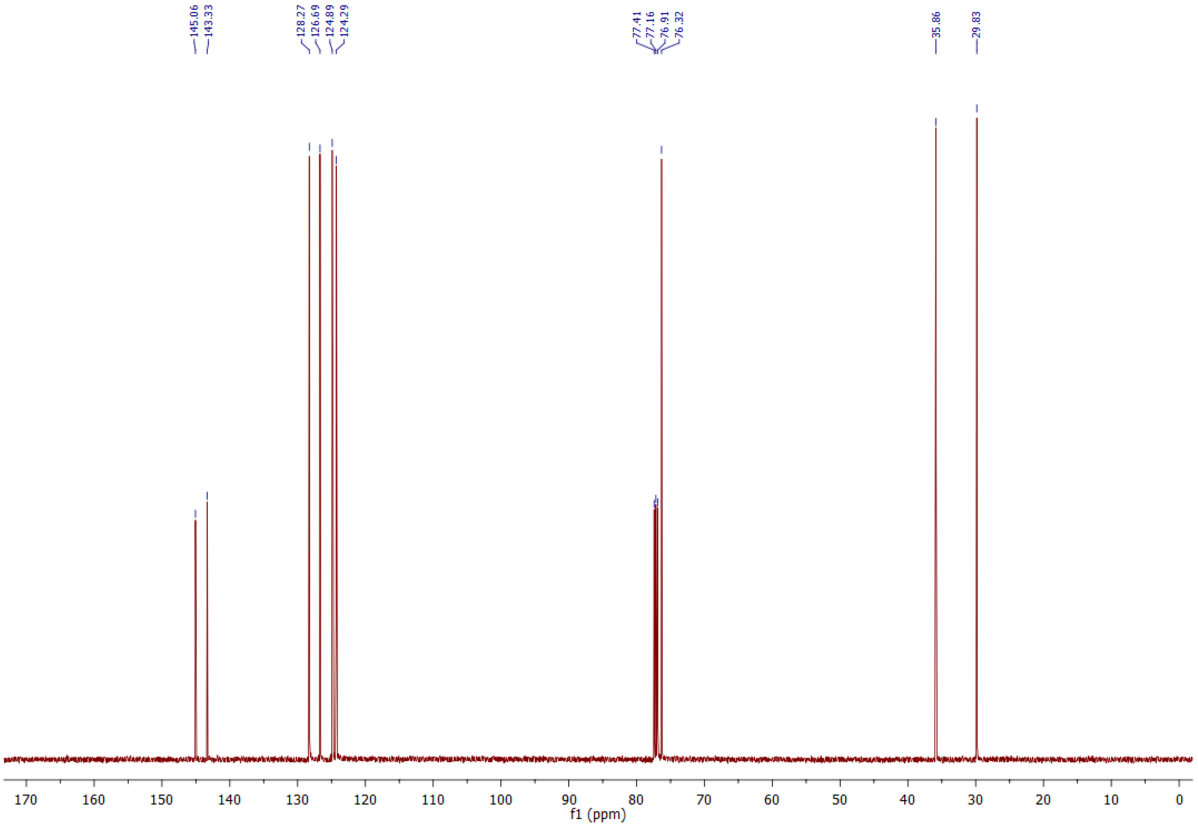

Supplementary Figure 21. FTMS spectrum of **2e** (ESI-TOF)

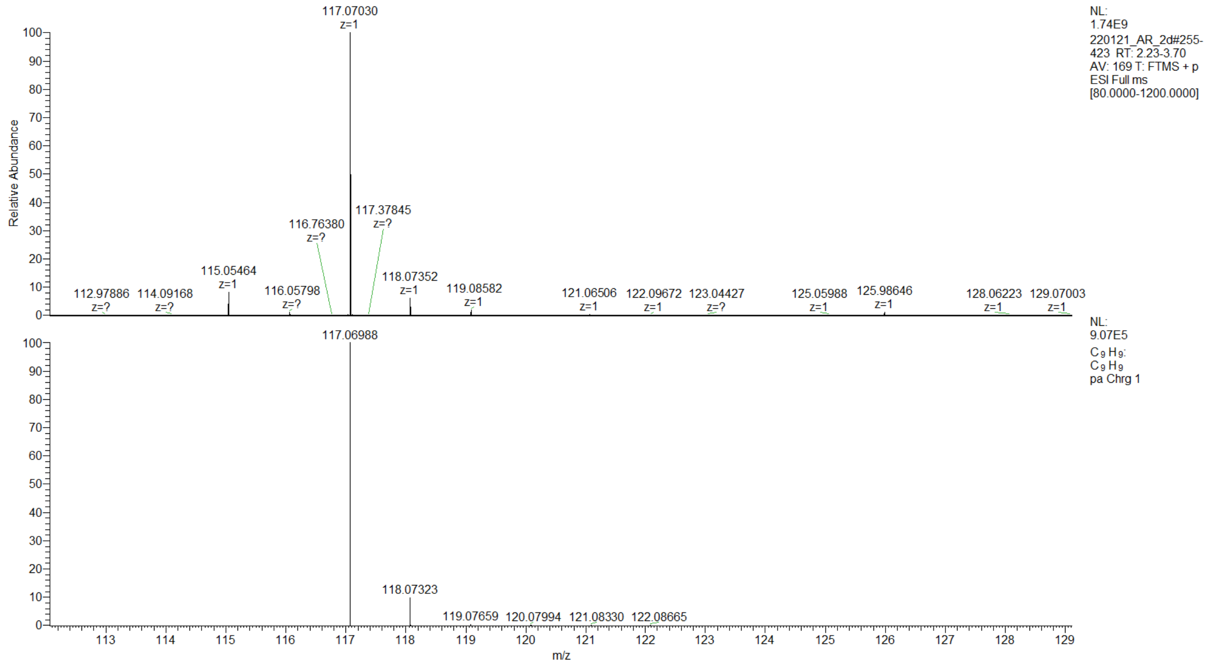

Supplementary Figure 22. ATR-FTIR spectrum of **2e** (neat)

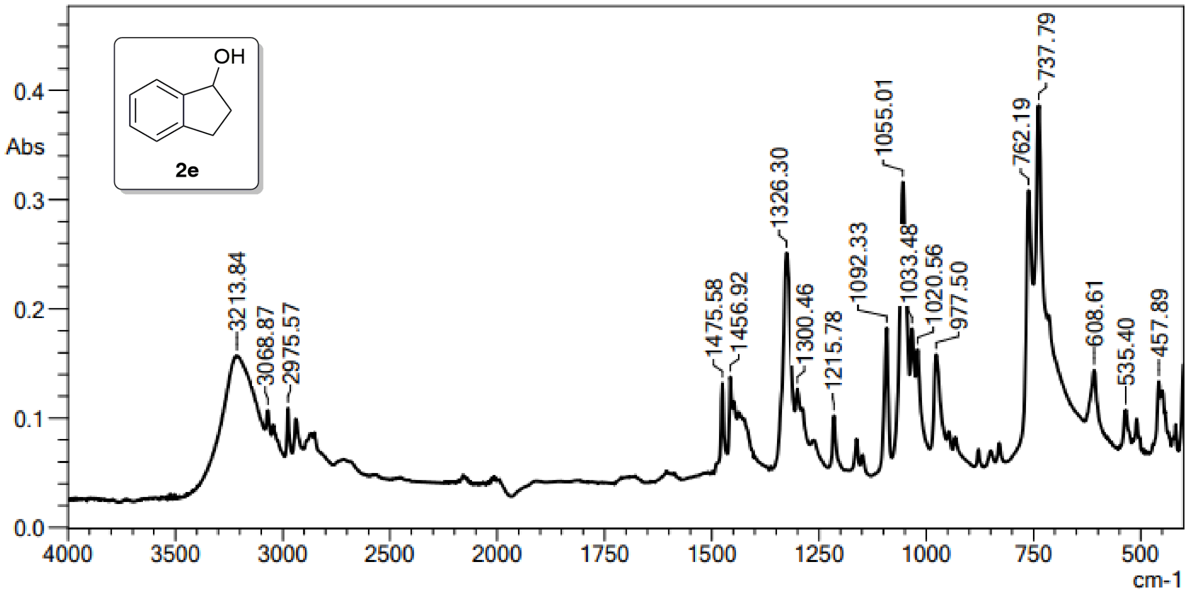

1,2,3,4-Tetrahydronaphthalen-1-ol (2f)

Supplementary Figure 23. ^{1}H NMR spectrum of **2f** (500 MHz, $CDCl_3$)

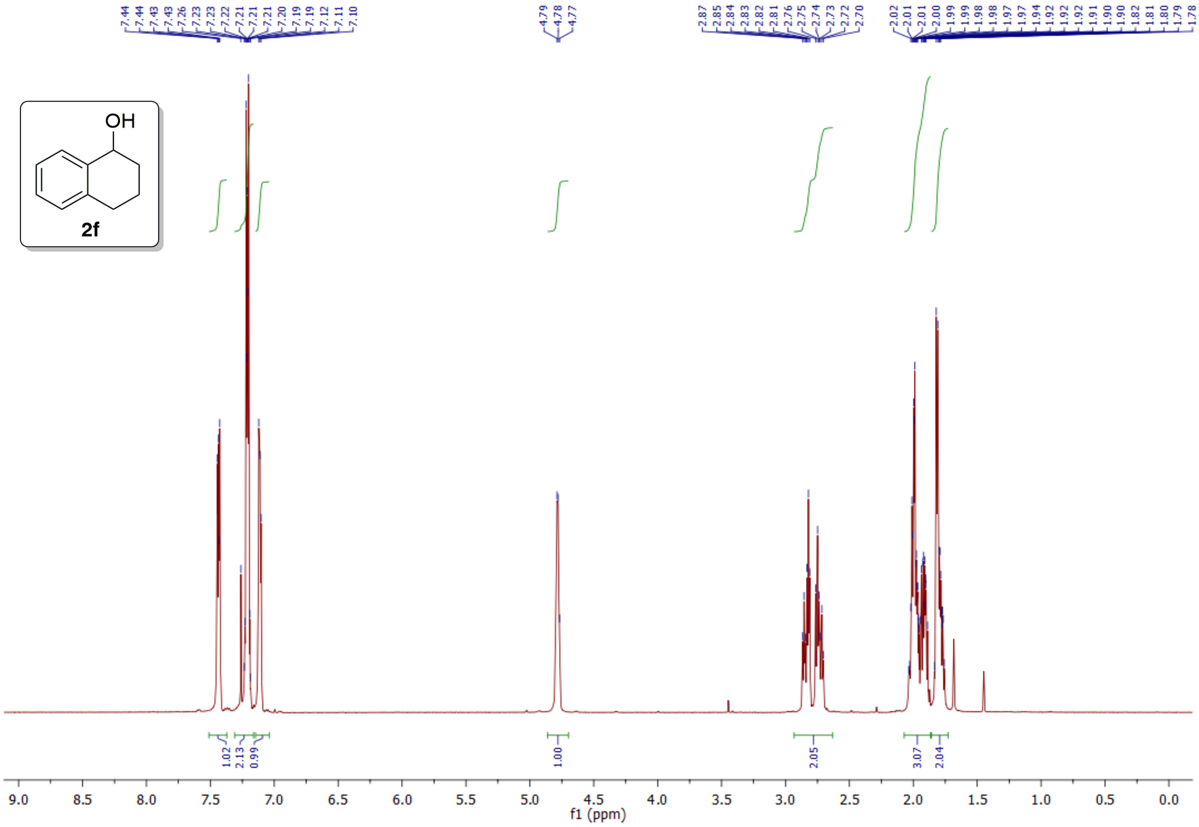

Supplementary Figure 24. $^{13}C\{^{1}H\}$ NMR spectrum of **2f** (126 MHz, $CDCl_3$)

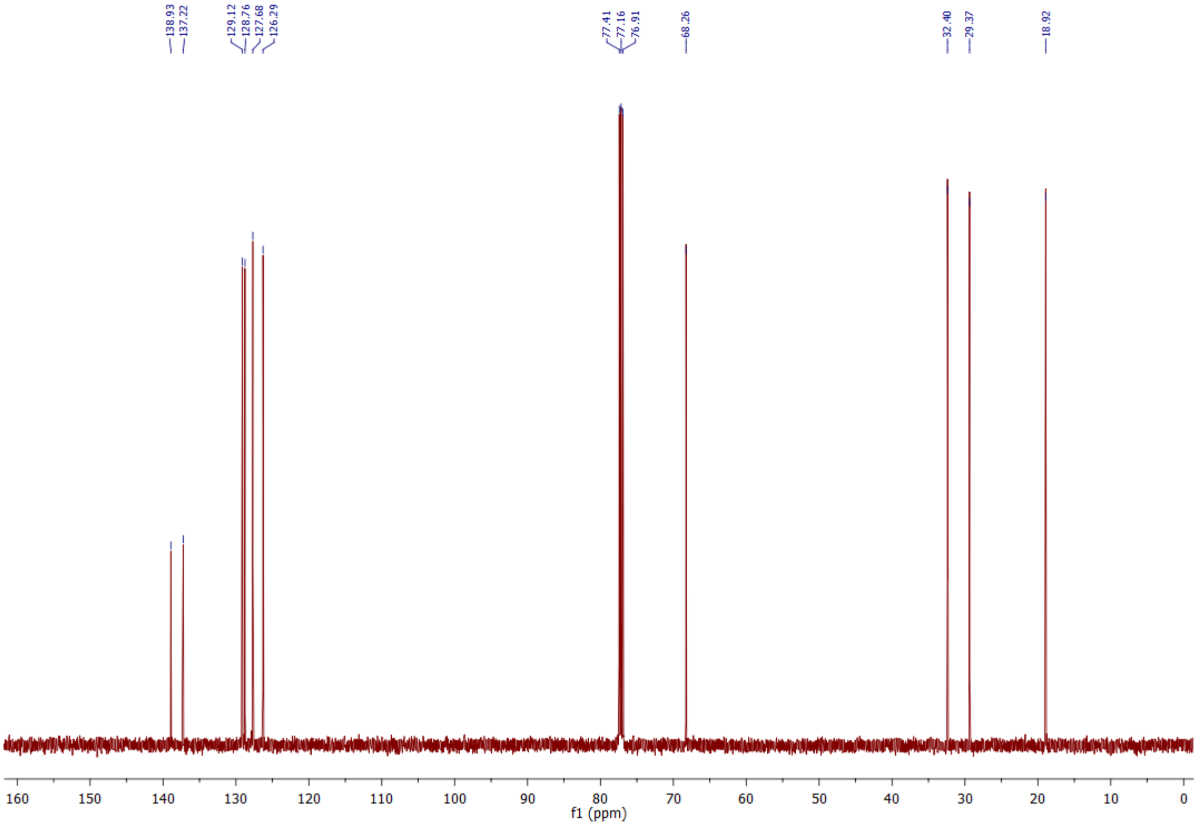

Supplementary Figure 25. FTMS spectrum of **2f** (ESI-TOF)

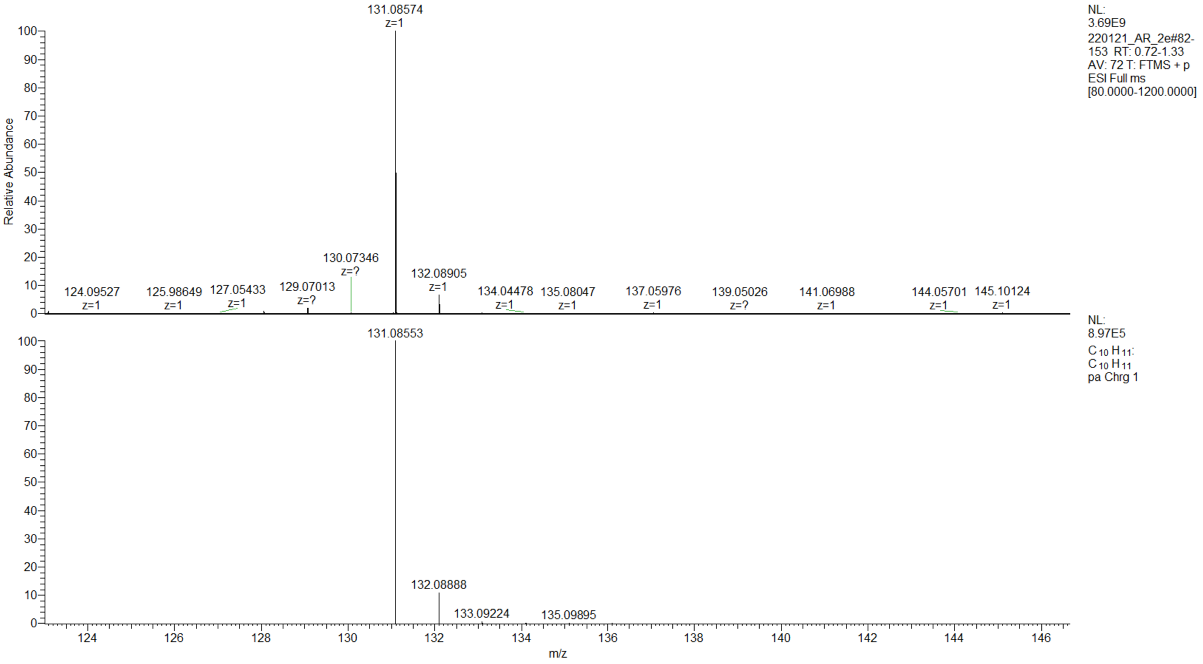

Supplementary Figure 26. ATR-FTIR spectrum of **2f** (neat)

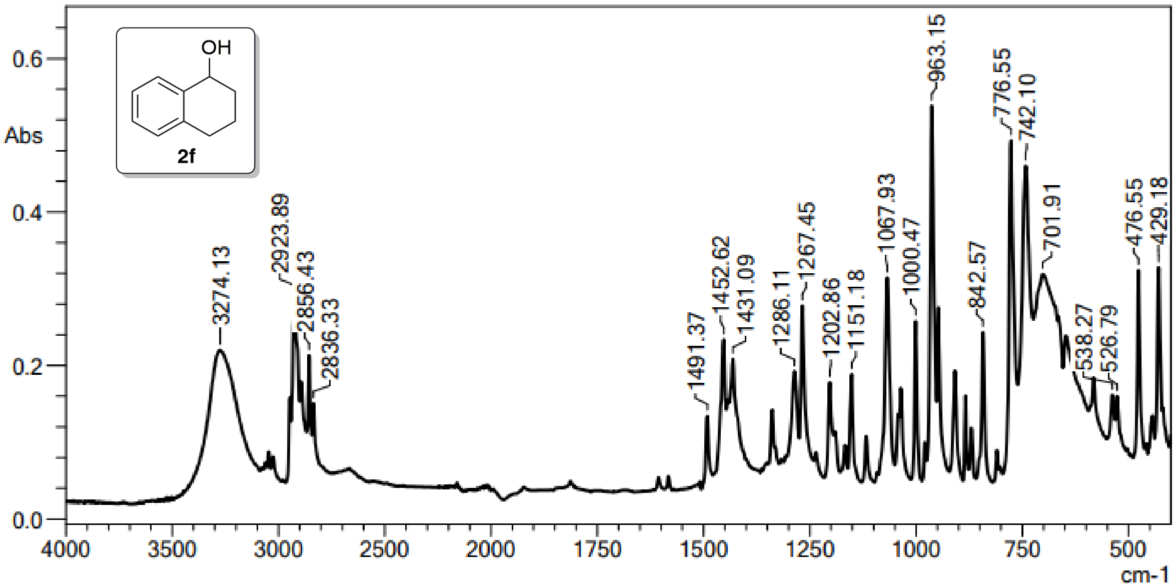

1-(Biphenyl-4-yl)ethanol (2g)

Supplementary Figure 27. ^{1}H NMR spectrum of **2g** (500 MHz, $CDCl_3$)

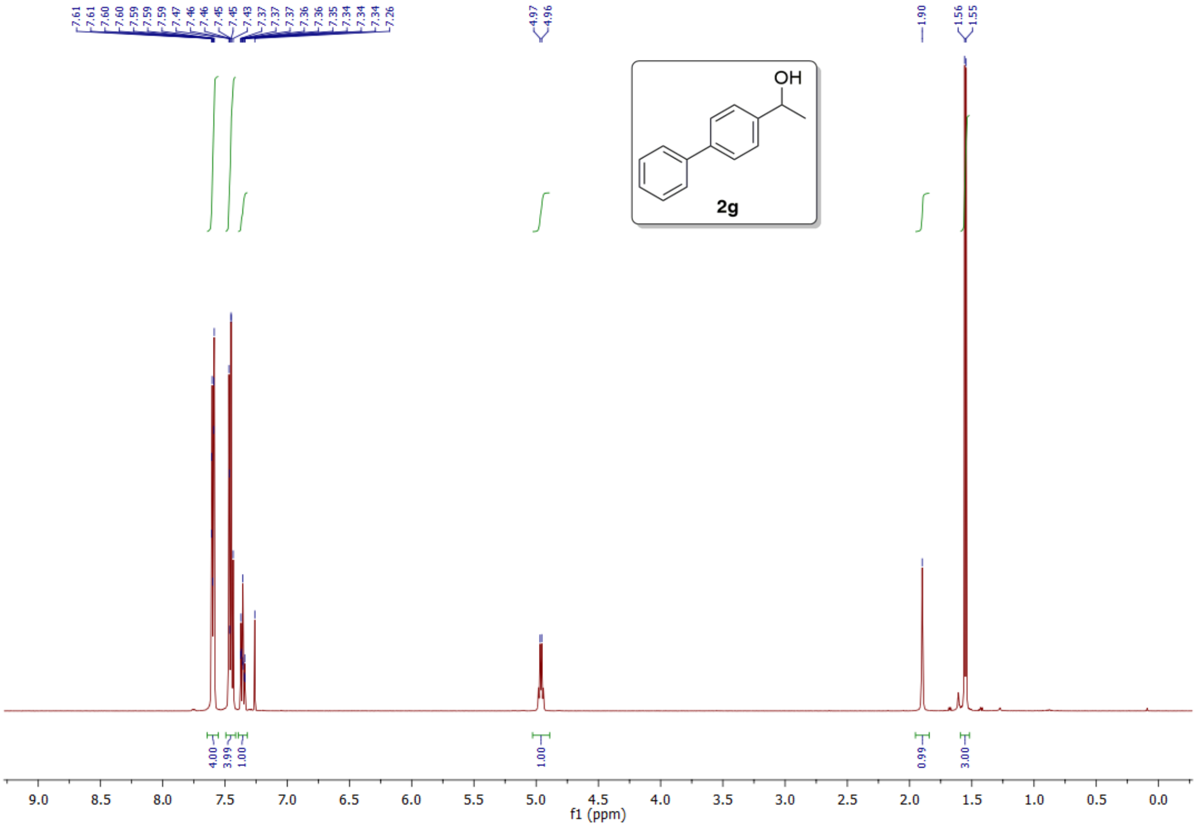

Supplementary Figure 28. $^{13}C\{^{1}H\}$ NMR spectrum of **2g** (126 MHz, $CDCl_3$)

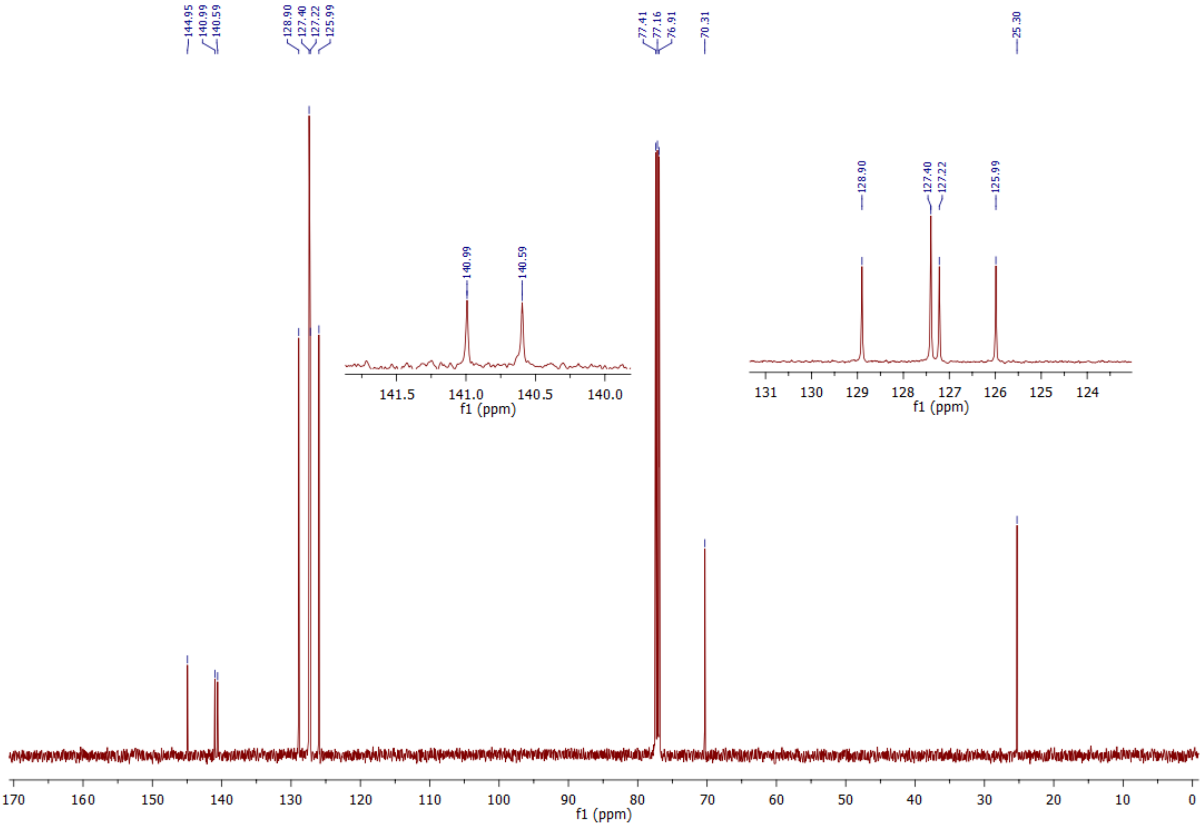

Supplementary Figure 29. FTMS spectrum of **2g** (ESI-TOF)

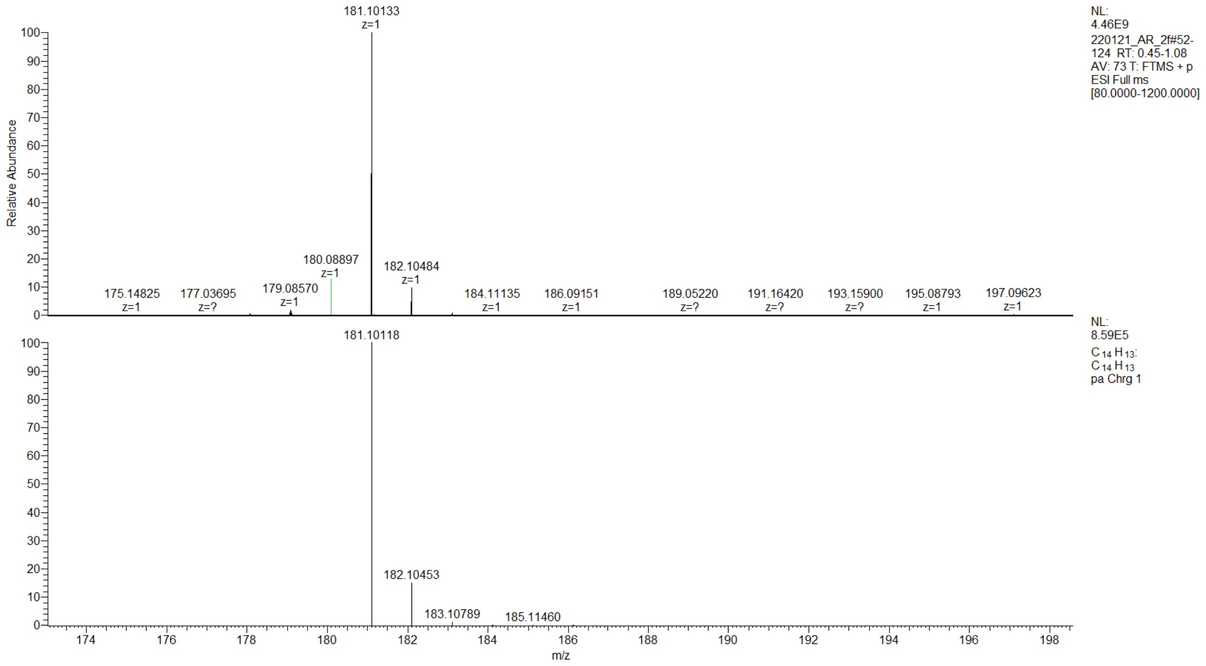

Supplementary Figure 30. ATR-FTIR spectrum of **2g** (neat)

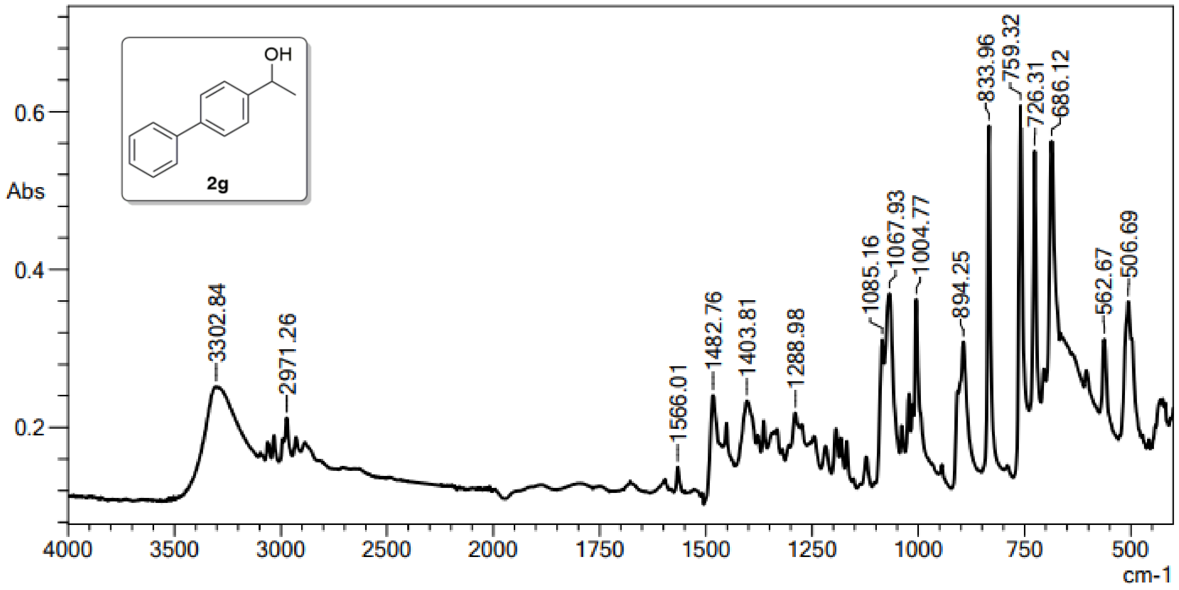

2,2,2-Trifluoro-1-phenylethanol (2h)

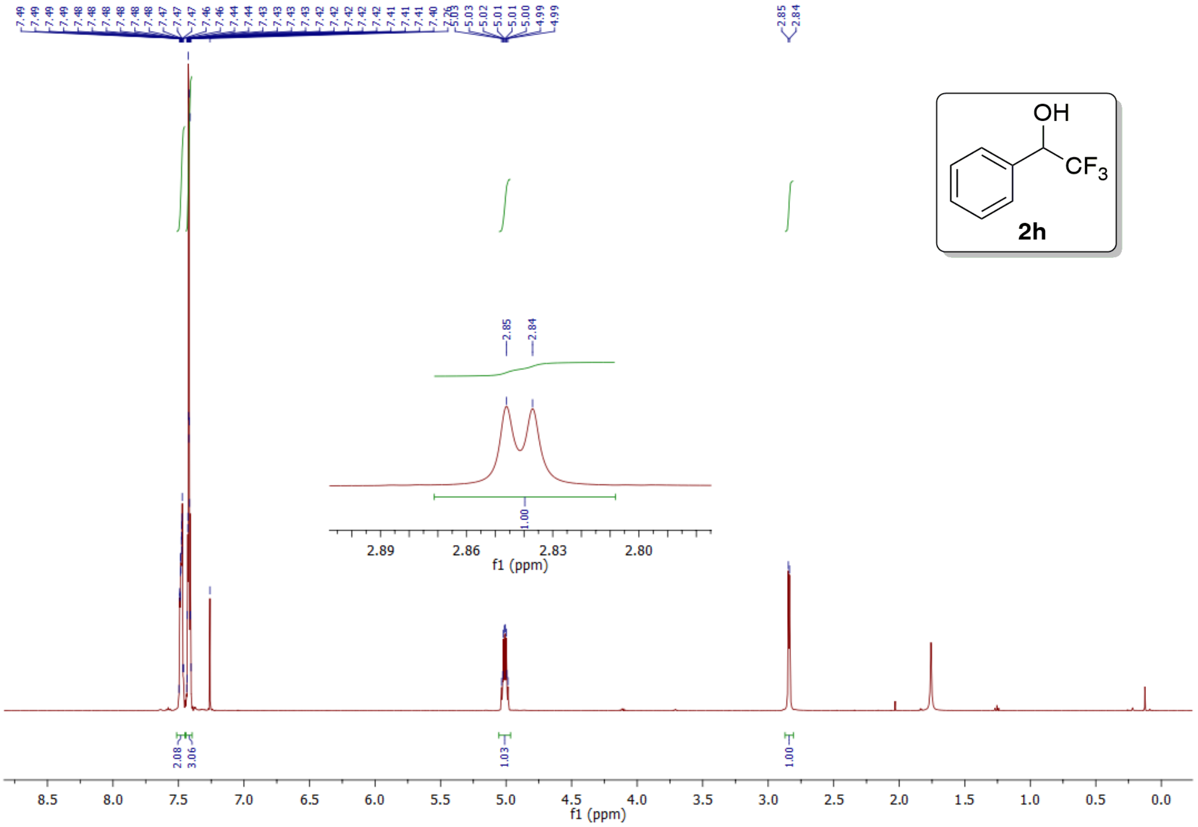

Supplementary Figure 31. ^{1}H NMR spectrum of **2h** (500 MHz, $CDCl_3$)

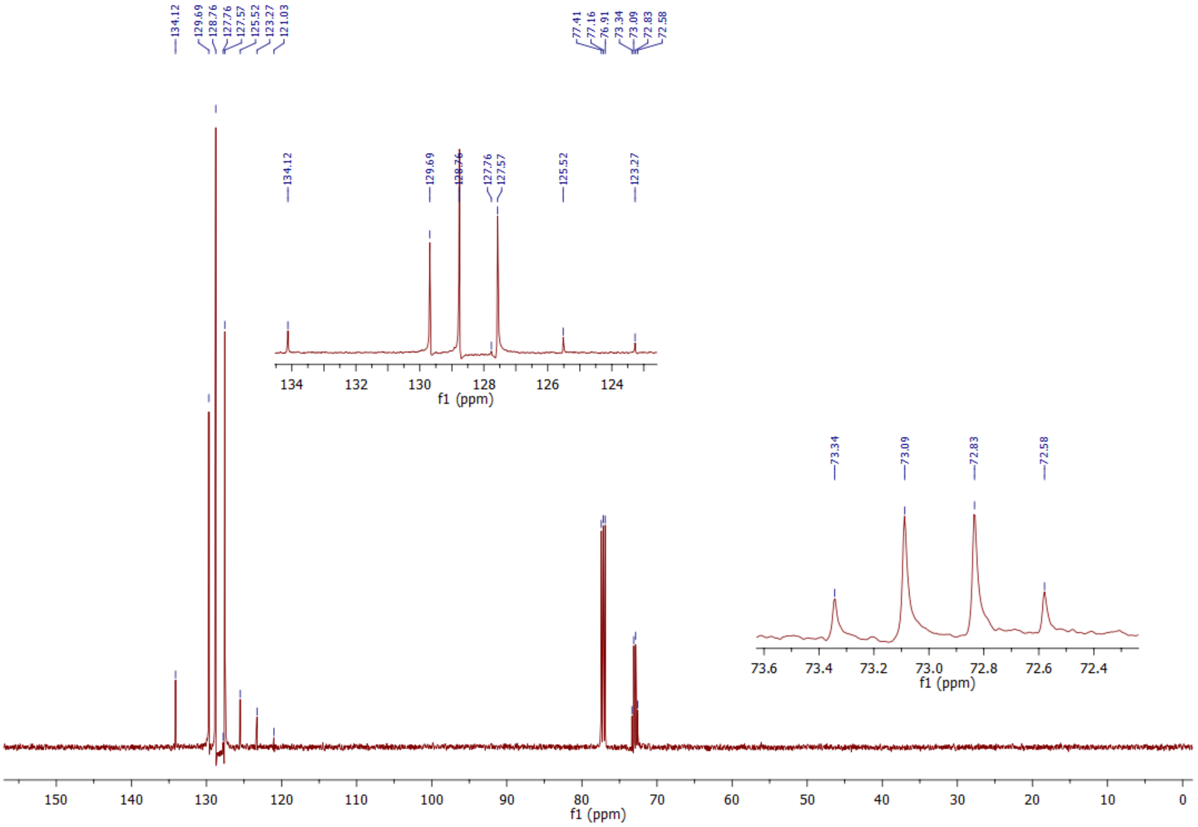

Supplementary Figure 32. $^{13}C\{^{1}H\}$ NMR spectrum of **2h** (126 MHz, $CDCl_3$)

Supplementary Figure 33. $^{19}F\{^{1}H\}$ NMR spectrum of **2h** (470 MHz, $CDCl_3$)

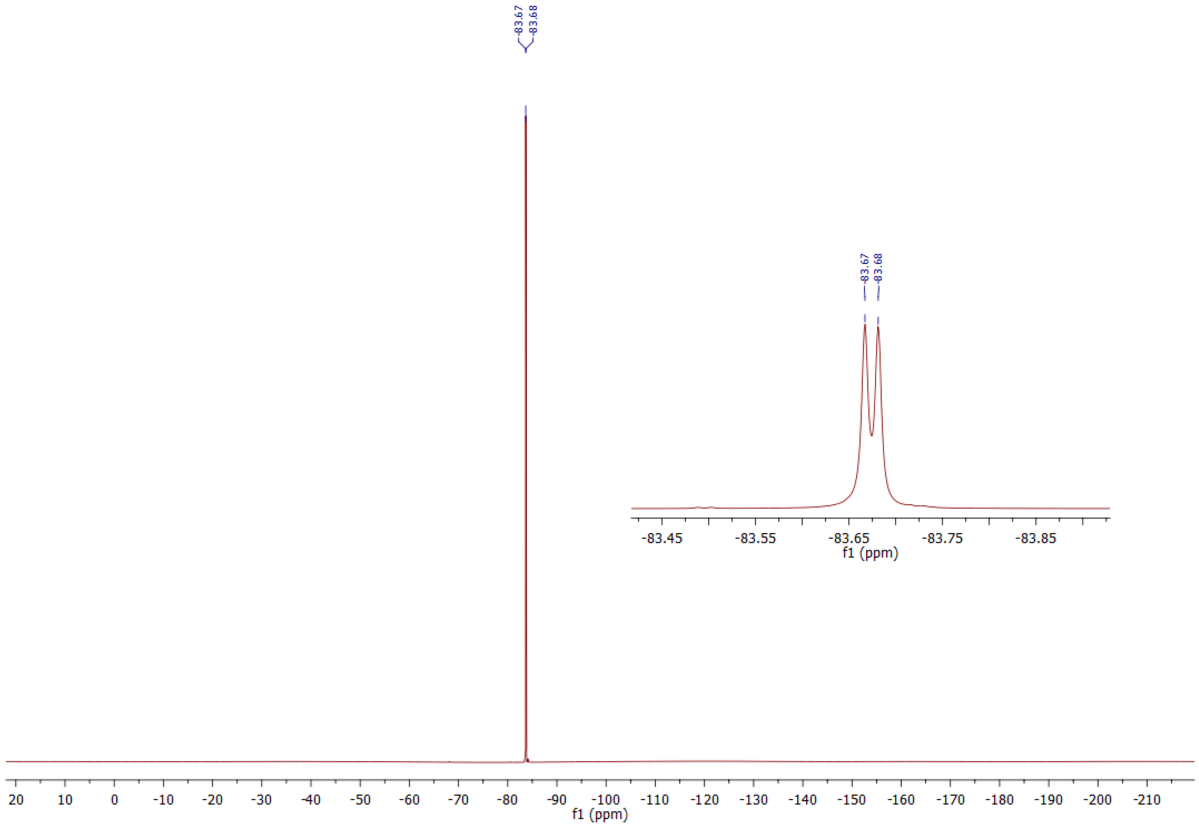

Supplementary Figure 34a. FTMS spectrum of **2h** (ESI-TOF)

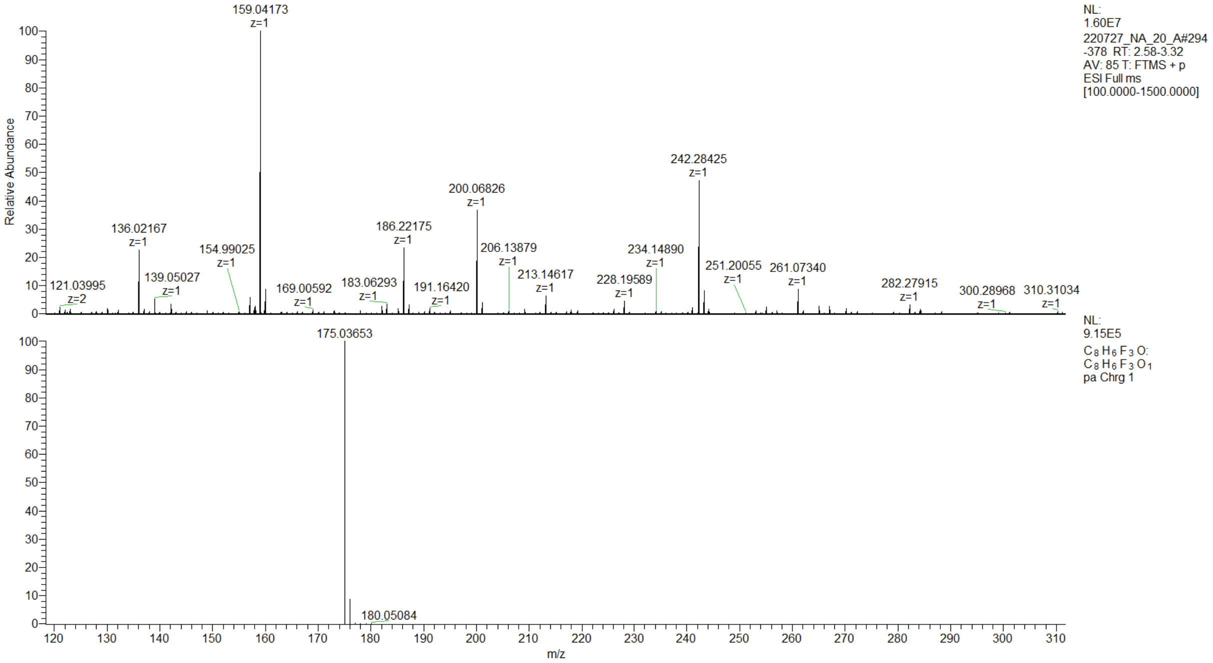

Supplementary Figure 34b. FTMS spectrum of **2h** (ESI-TOF)

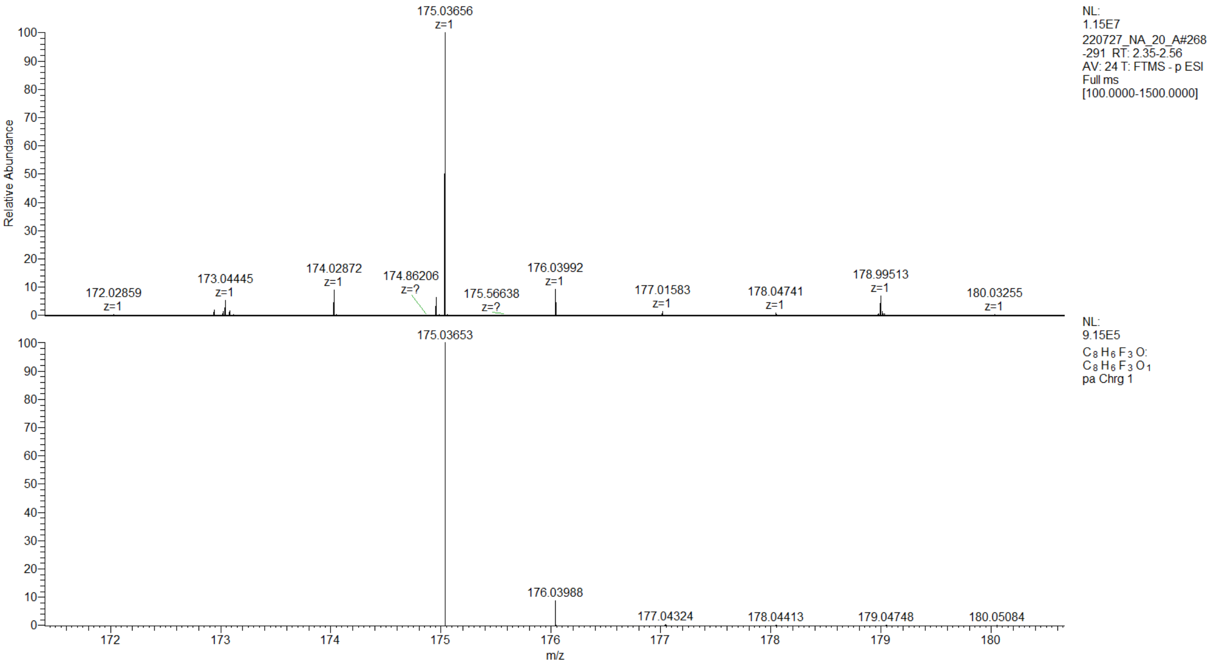

Supplementary Figure 35. ATR-FTIR spectrum of **2h** (neat)

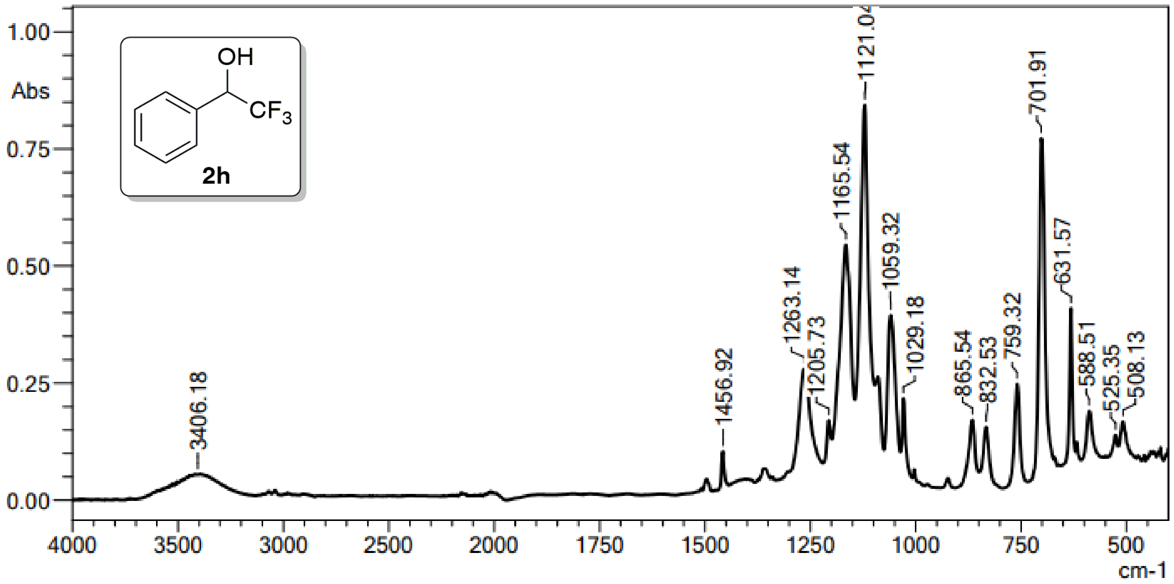

1-[3,5-bis(Trifluoromethyl)phenyl]ethanol (2i)

Supplementary Figure 36. ^{1}H NMR spectrum of **2i** (500 MHz, $CDCl_3$)

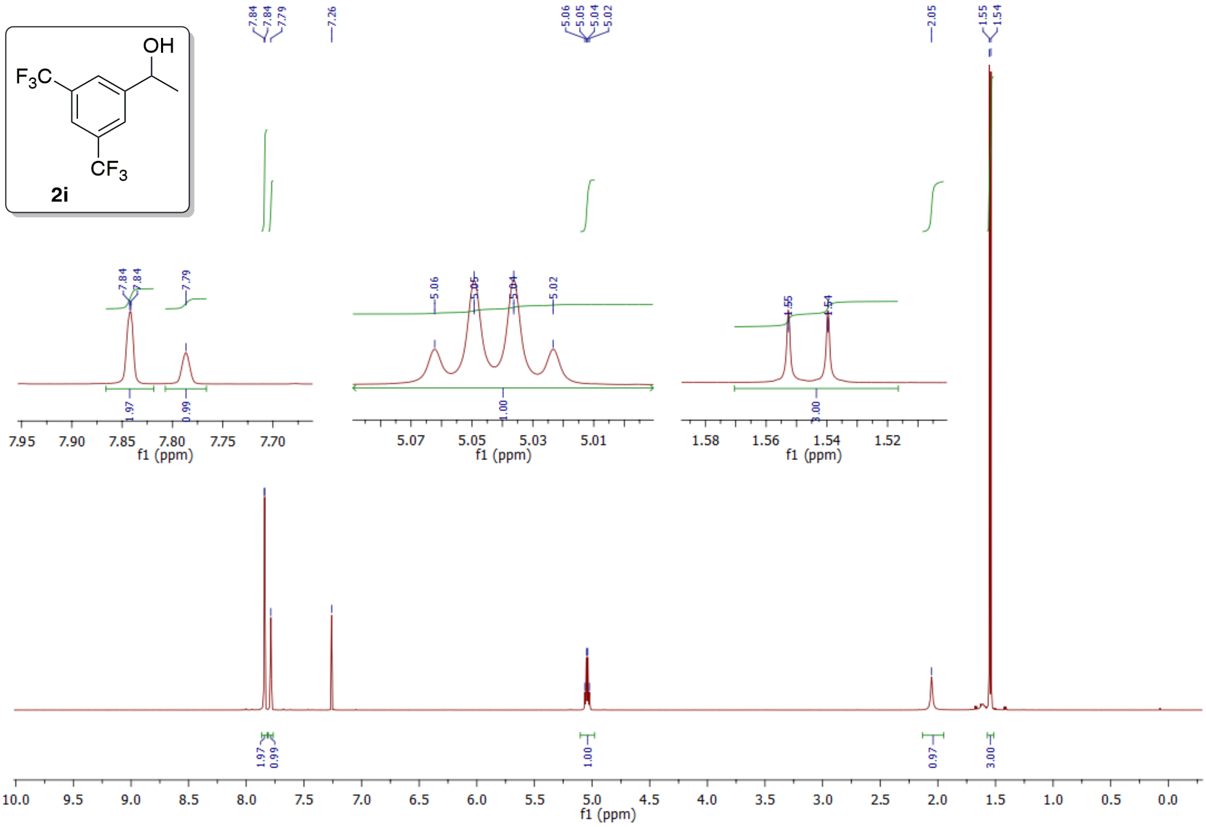

Supplementary Figure 37. ^{13}C{^{1}H} NMR spectrum of **2i** (126 MHz, $CDCl_3$)

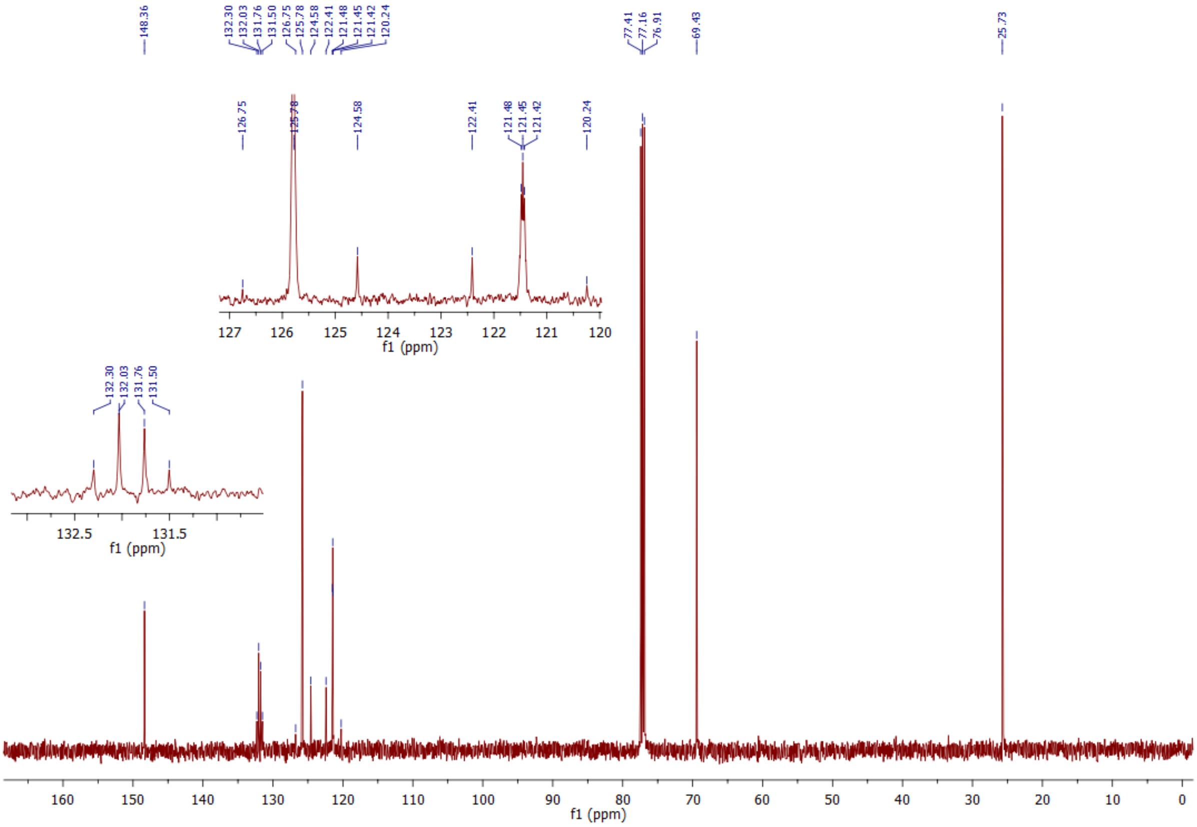

Supplementary Figure 38. $^{19}F\{^{1}H\}$ NMR spectrum of **2i** (470 MHz, $CDCl_3$)

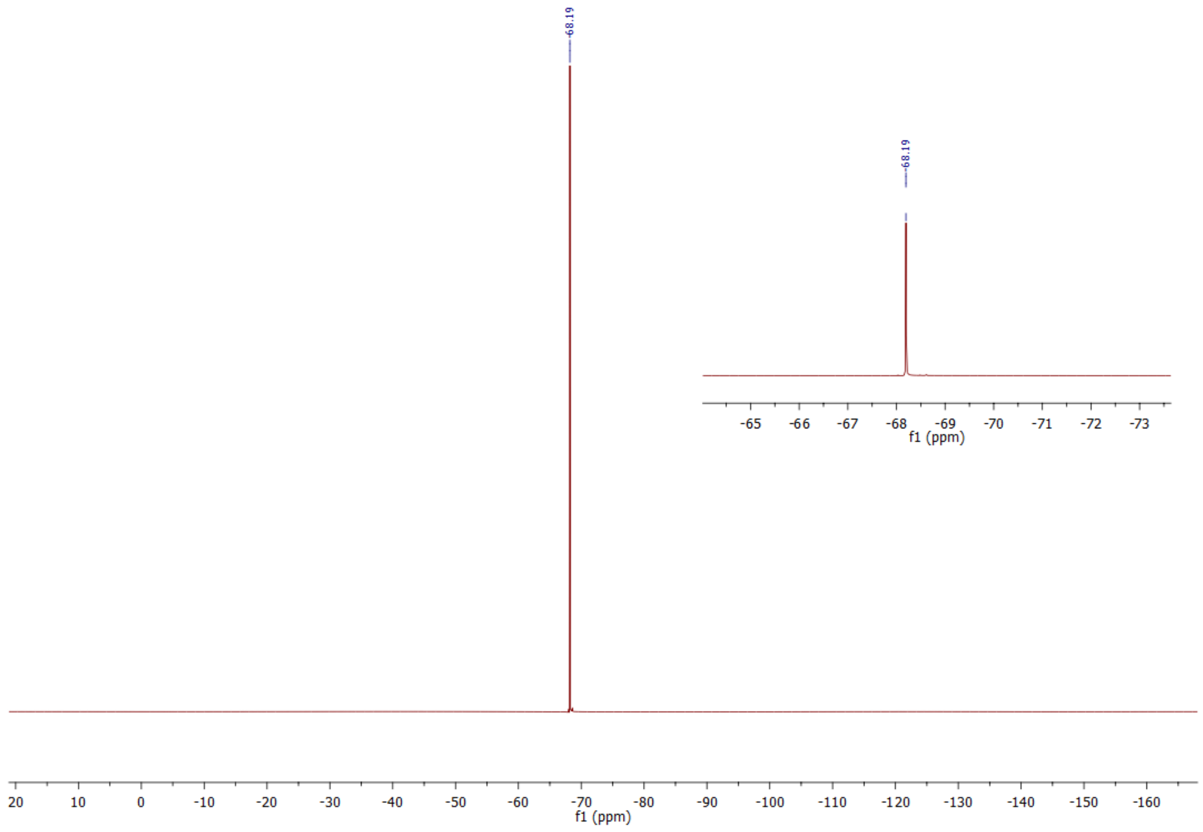

Supplementary Figure 39a. FTMS spectrum of **2i** (ESI-TOF)

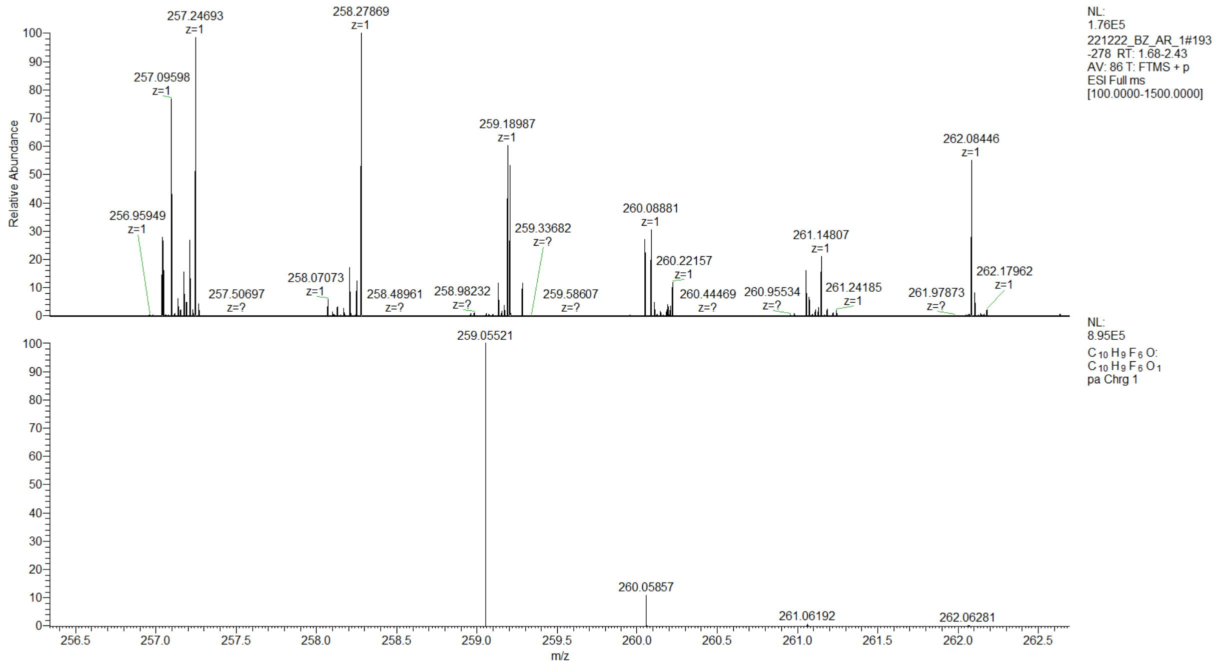

Supplementary Figure 39b. FTMS spectrum of **2i** (ESI-TOF)

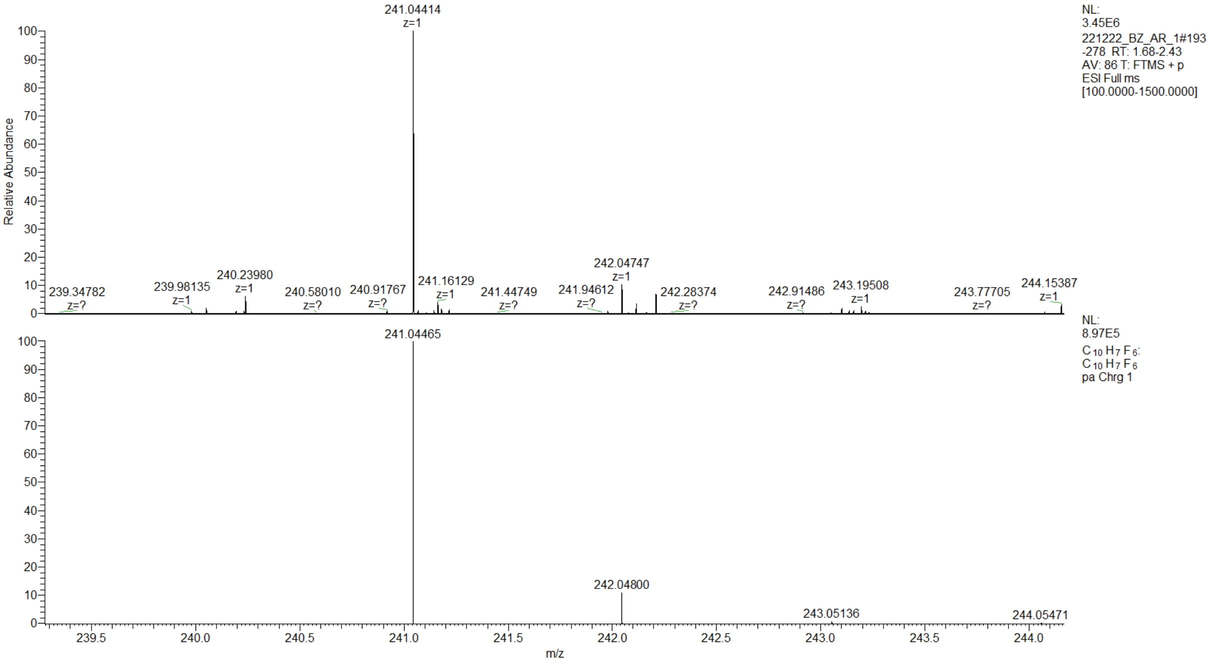

Supplementary Figure 40. ATR-FTIR spectrum of **2i** (neat)

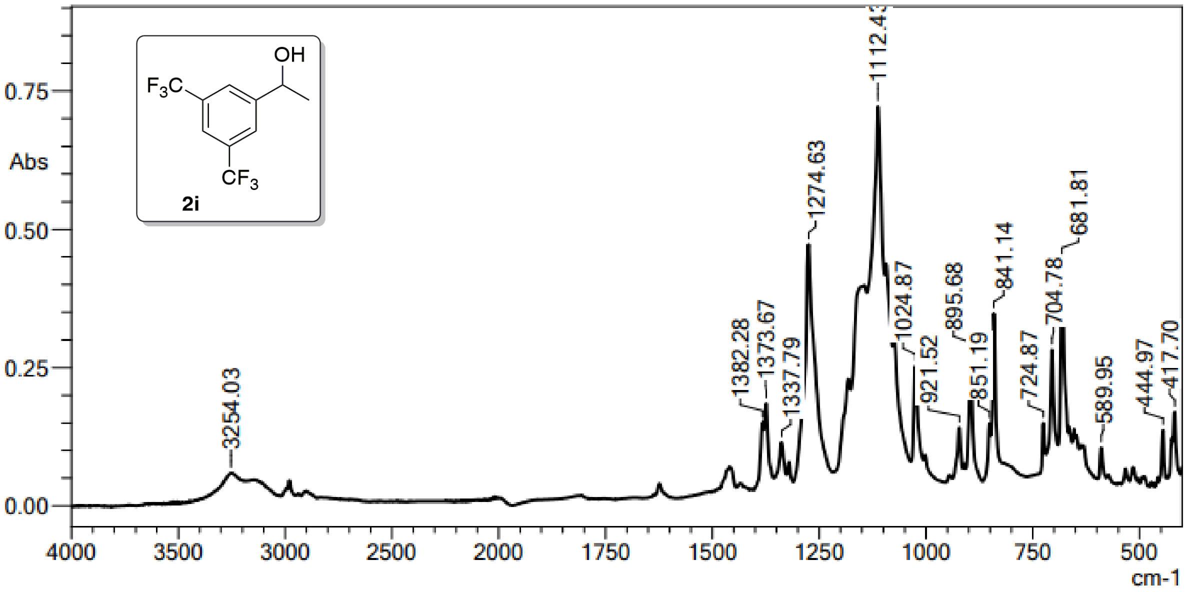

1-Phenylpropan-2-ol (2j)

Supplementary Figure 41. ^{1}H NMR spectrum of **2j** (500 MHz, $CDCl_3$)

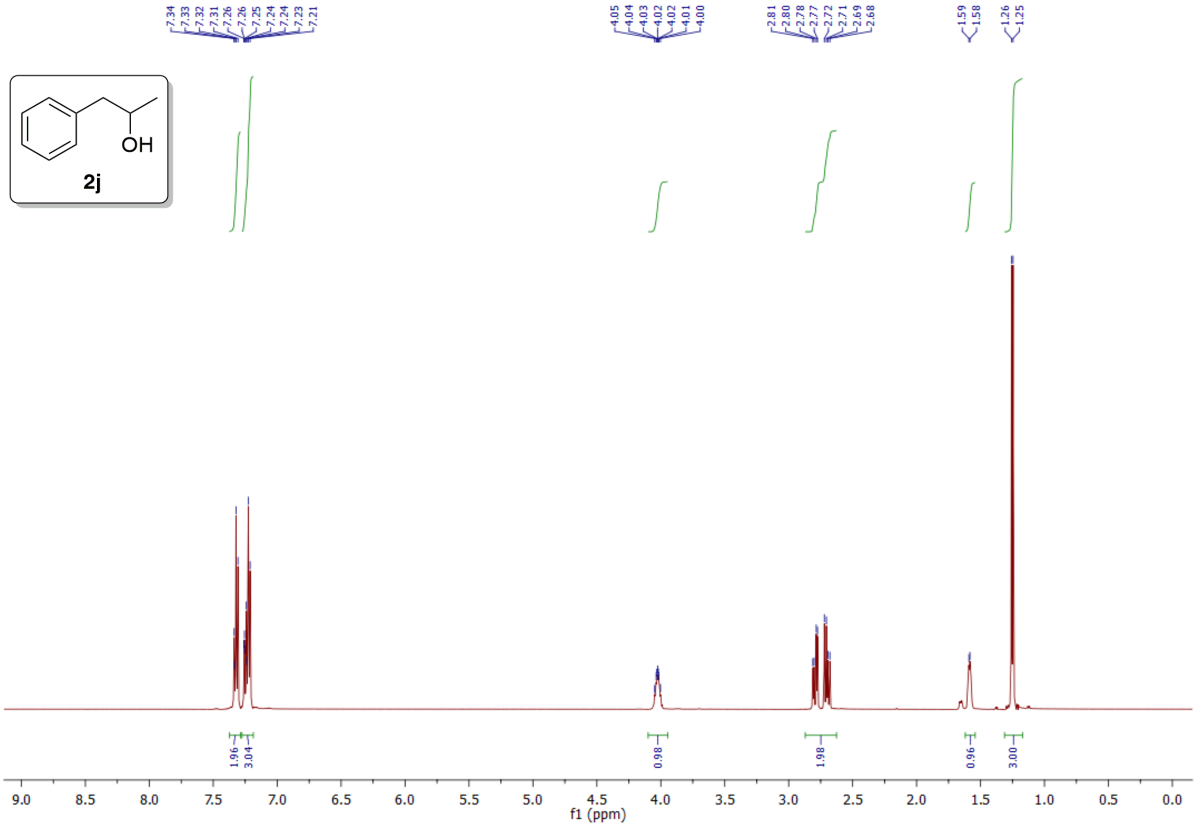

Supplementary Figure 42. $^{13}C\{^{1}H\}$ NMR spectrum of **2j** (126 MHz, $CDCl_3$)

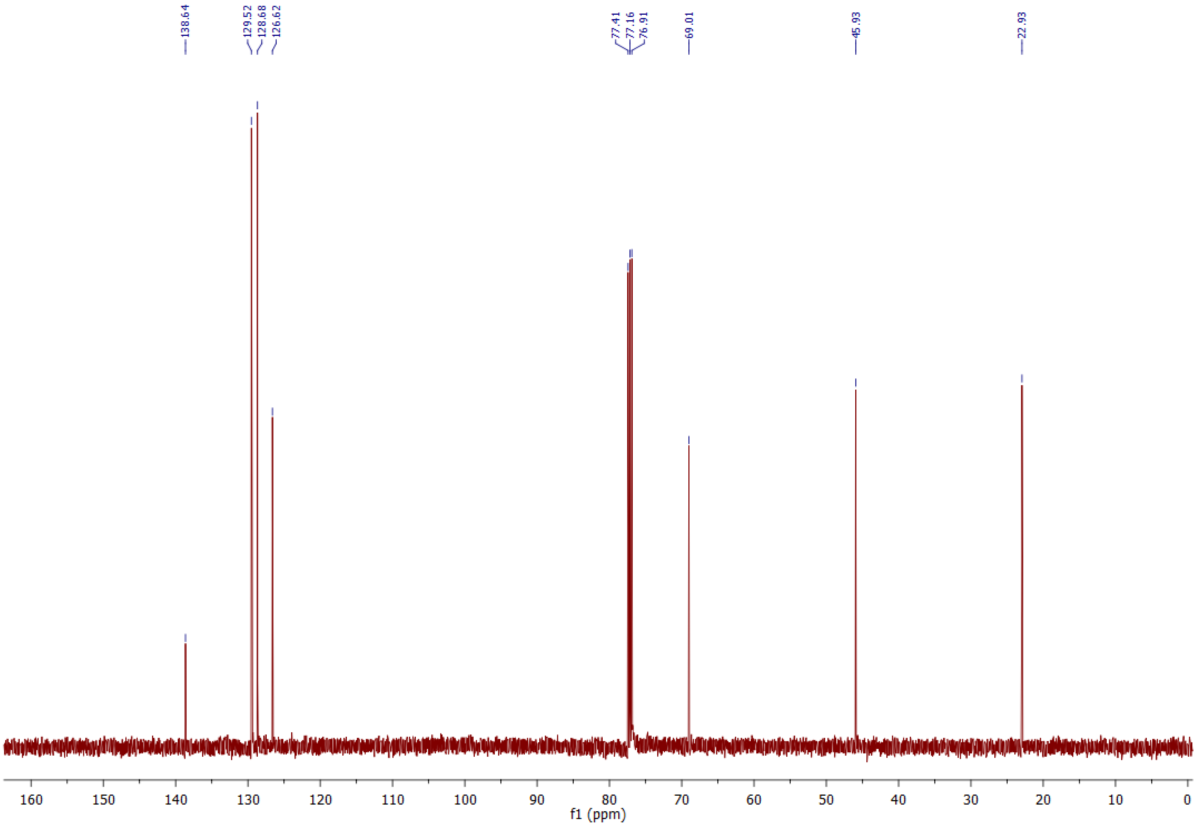

Supplementary Figure 43. FTMS spectrum of **2j** (ESI-TOF)

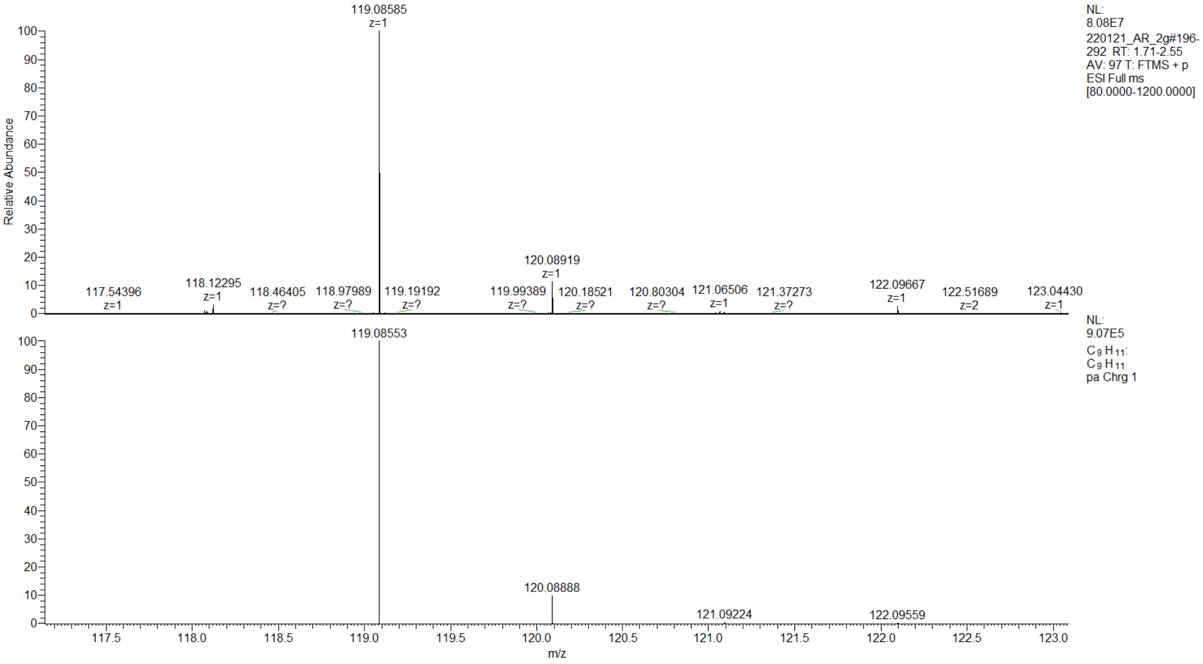

Supplementary Figure 44. ATR-FTIR spectrum of **2j** (neat)

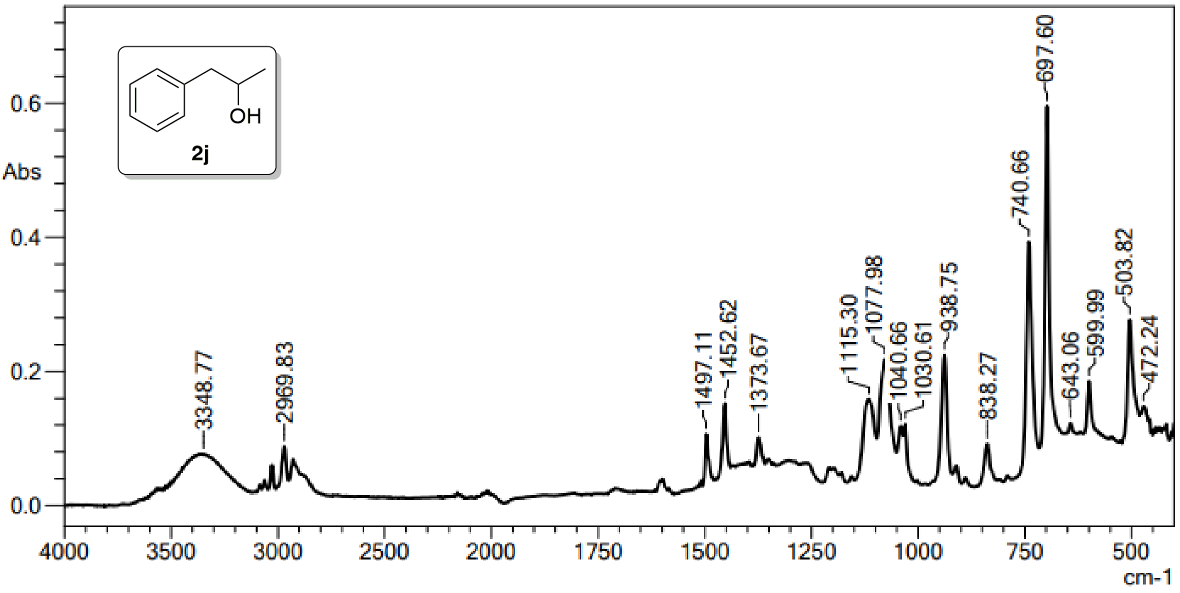

4-Phenylbutan-2-ol (2k)

Supplementary Figure 45. ^{1}H NMR spectrum of **2k** (500 MHz, $CDCl_3$)

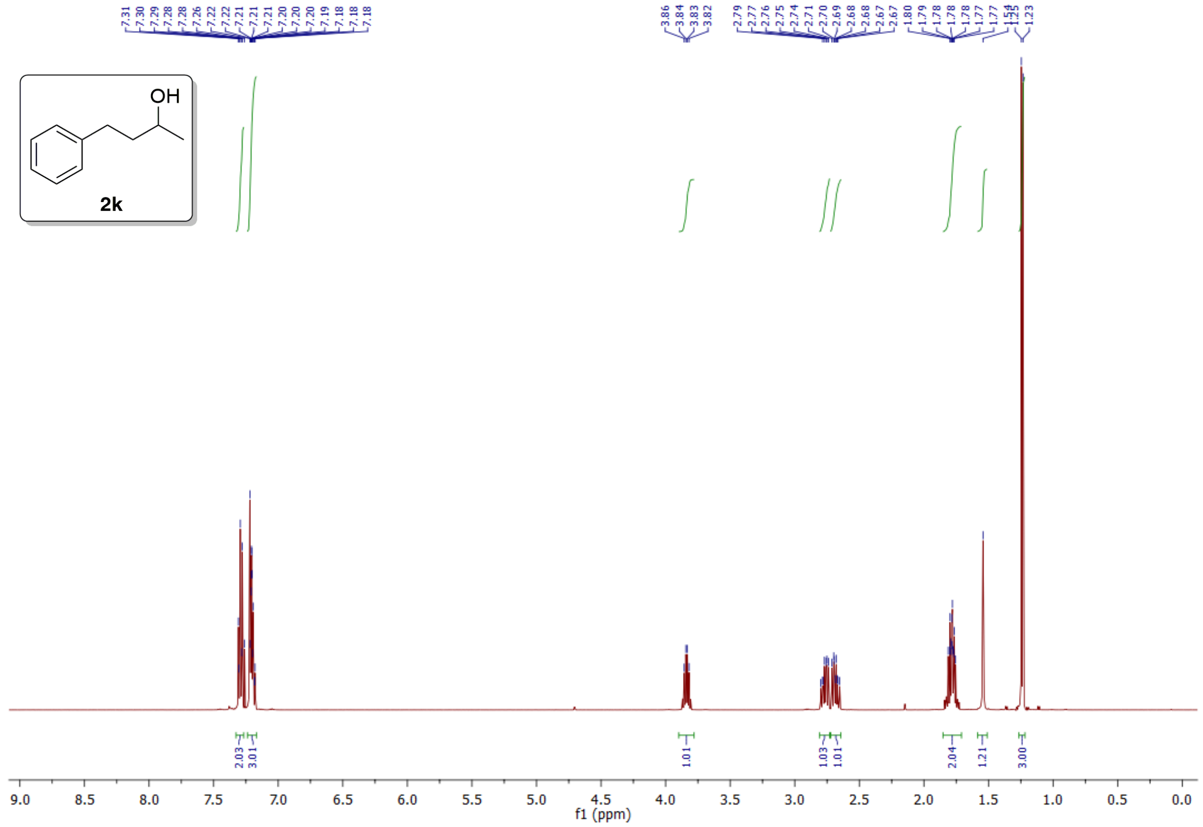

Supplementary Figure 46. $^{13}C\{^{1}H\}$ NMR spectrum of **2k** (126 MHz, $CDCl_3$)

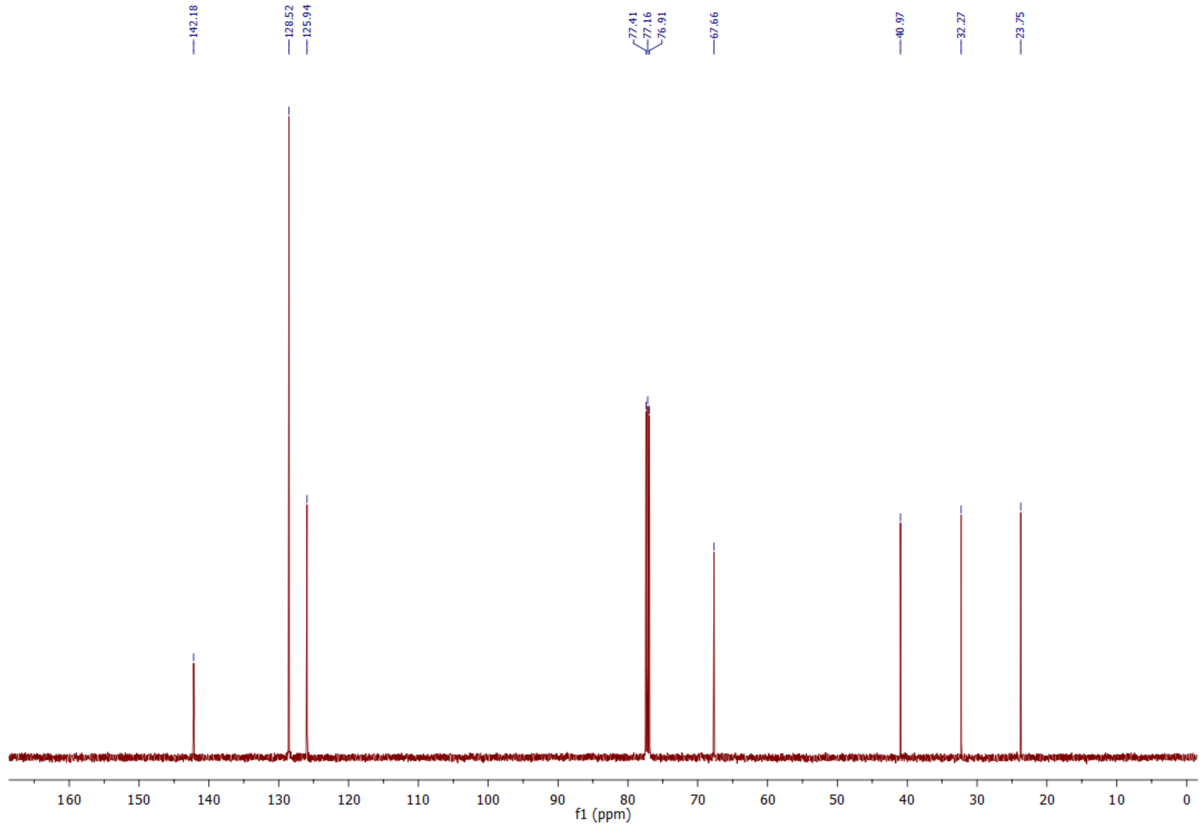

Supplementary Figure 47. FTMS spectrum of **2k** (ESI-TOF)

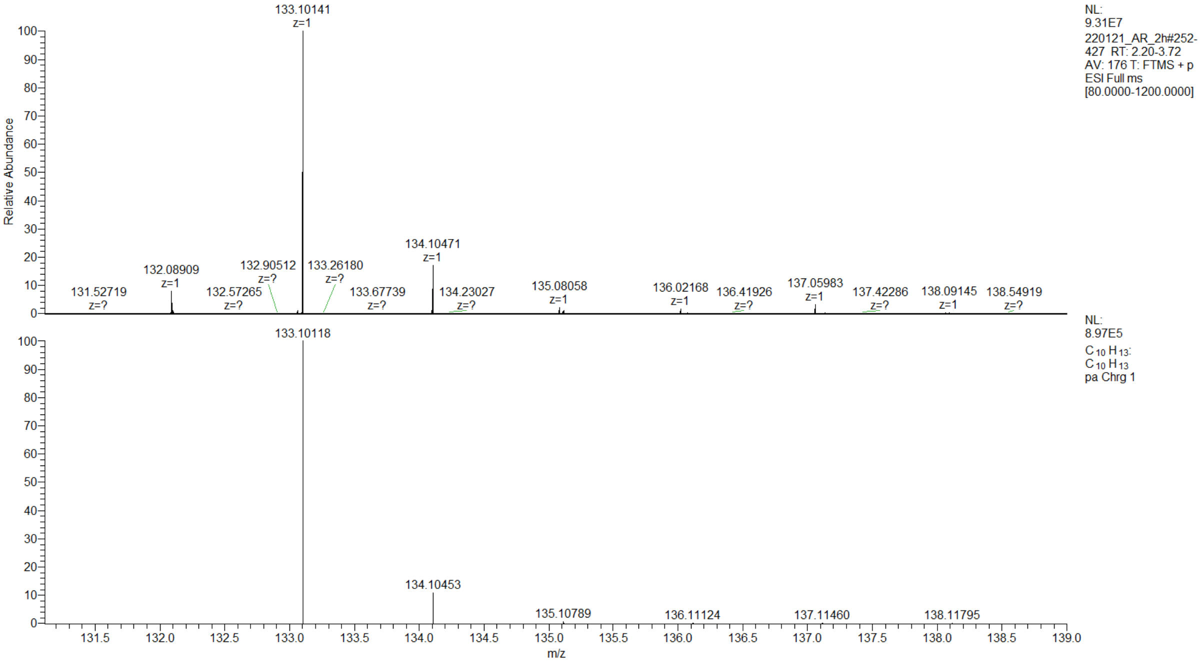

Supplementary Figure 48. ATR-FTIR spectrum of **2k** (neat)

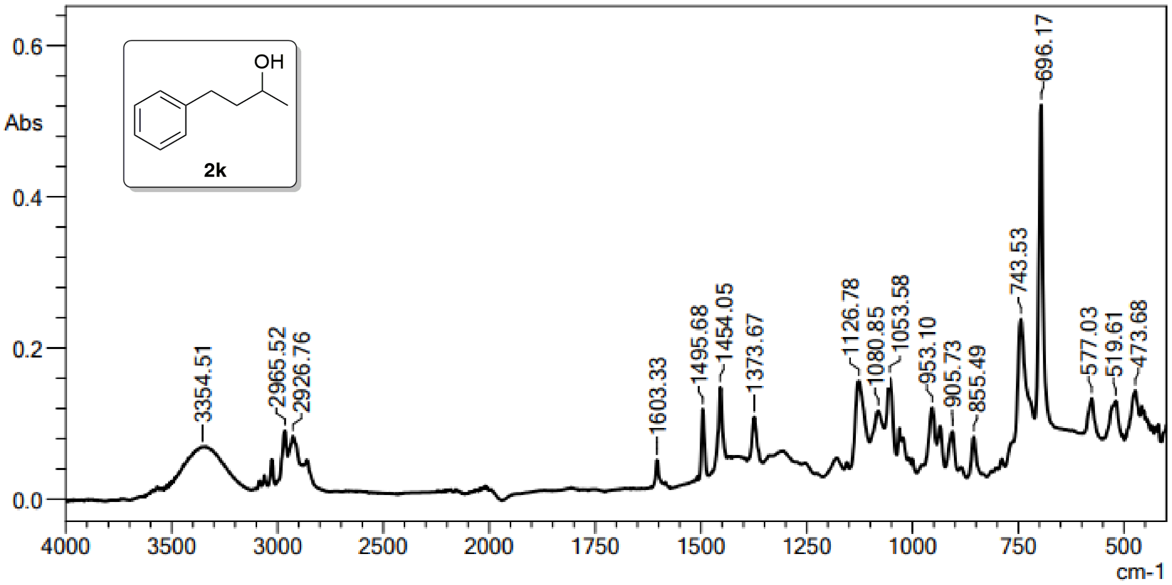

(3E)-4-Phenylbut-3-en-2-ol (2l)

Supplementary Figure 49. ^{1}H NMR spectrum of **2l** (500 MHz, $CDCl_3$)

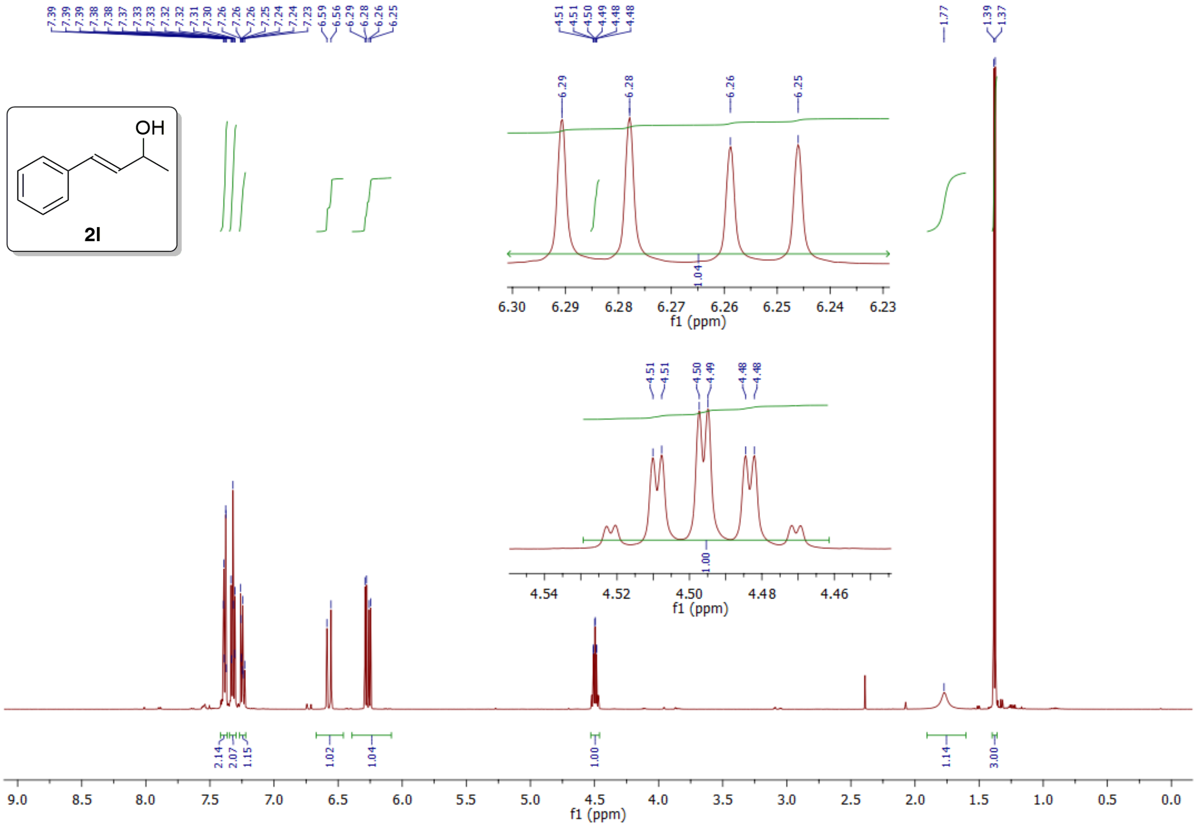

Supplementary Figure 50. $^{13}C\{^{1}H\}$ NMR spectrum of **2l** (126 MHz, $CDCl_3$)

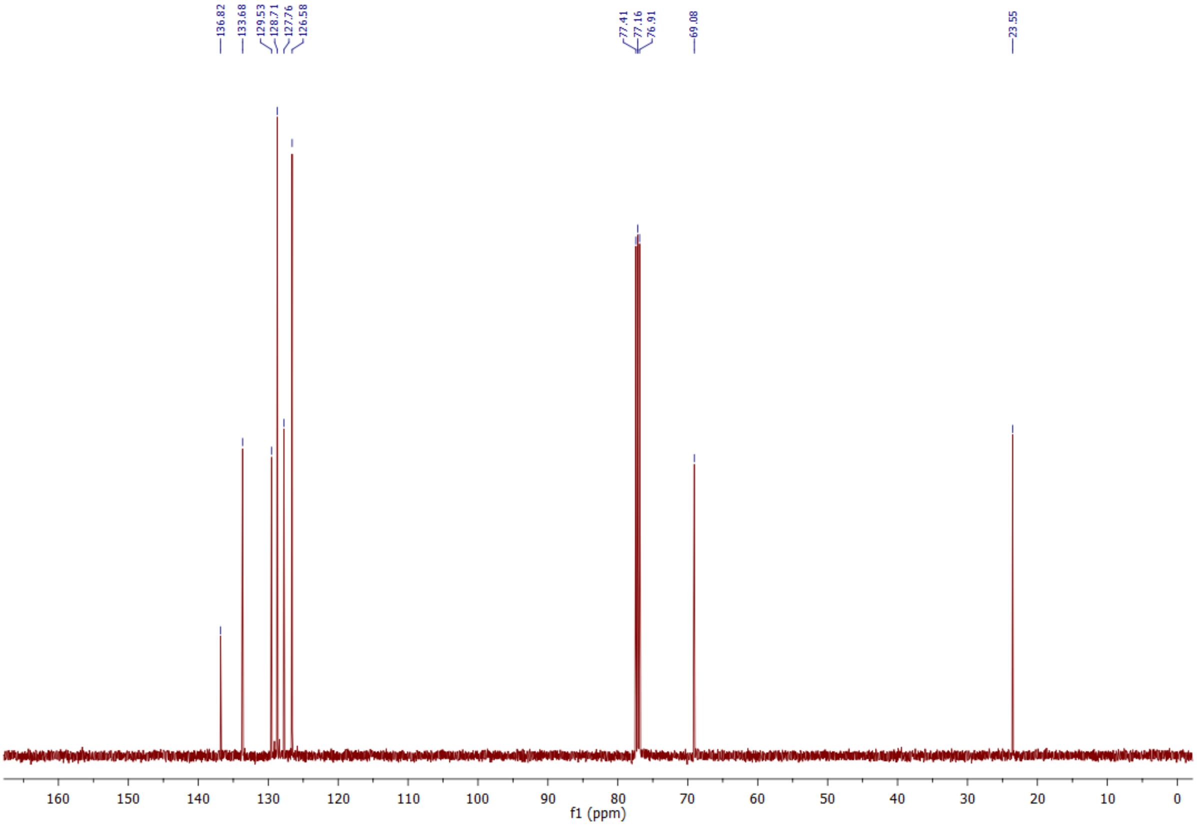

Supplementary Figure 51. FTMS spectrum of **2l** (ESI-TOF)

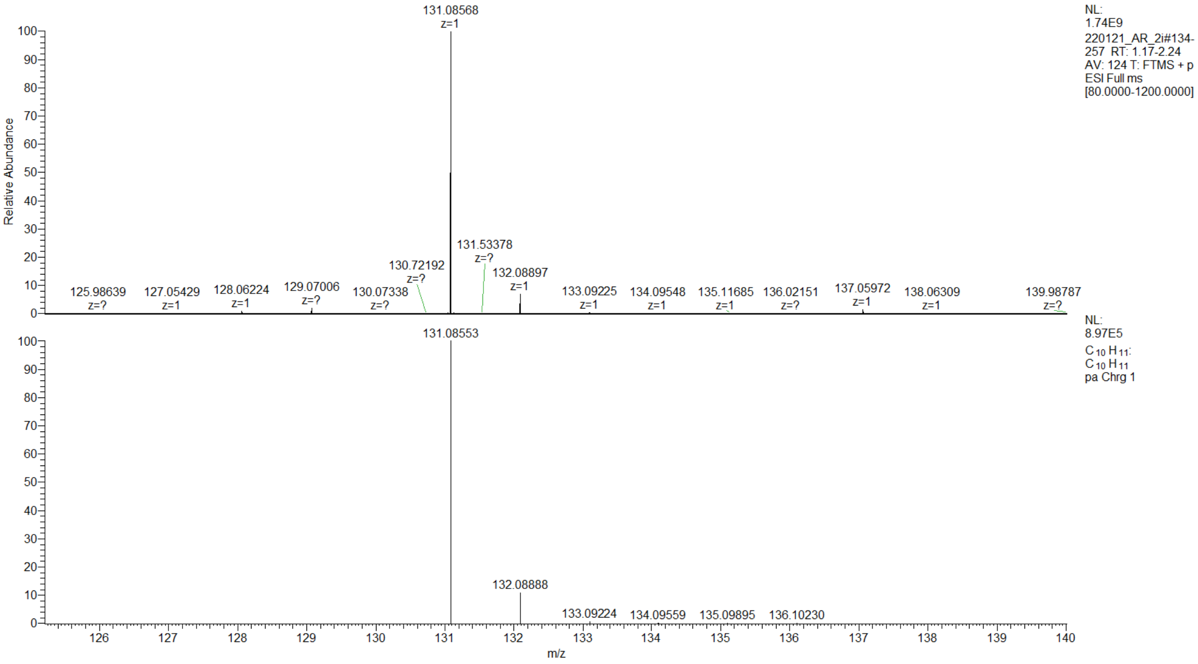

Supplementary Figure 52. ATR-FTIR spectrum of **2l** (neat)

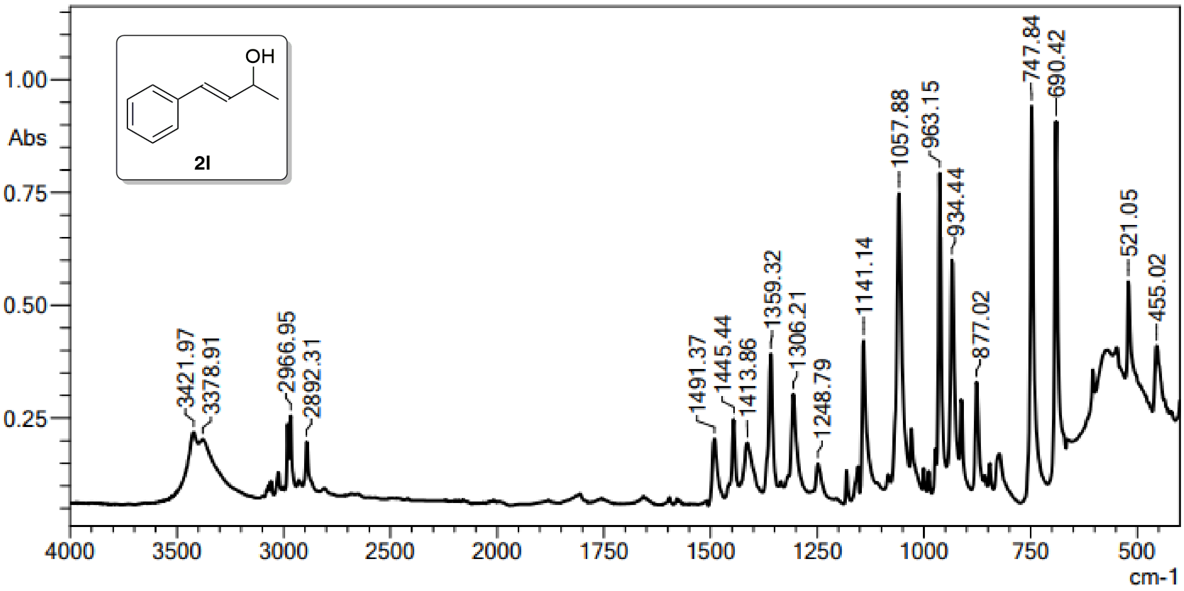

2-Chloro-1-phenylethanol (2m)

Supplementary Figure 53. ^{1}H NMR spectrum of **2m** (500 MHz, $CDCl_3$)

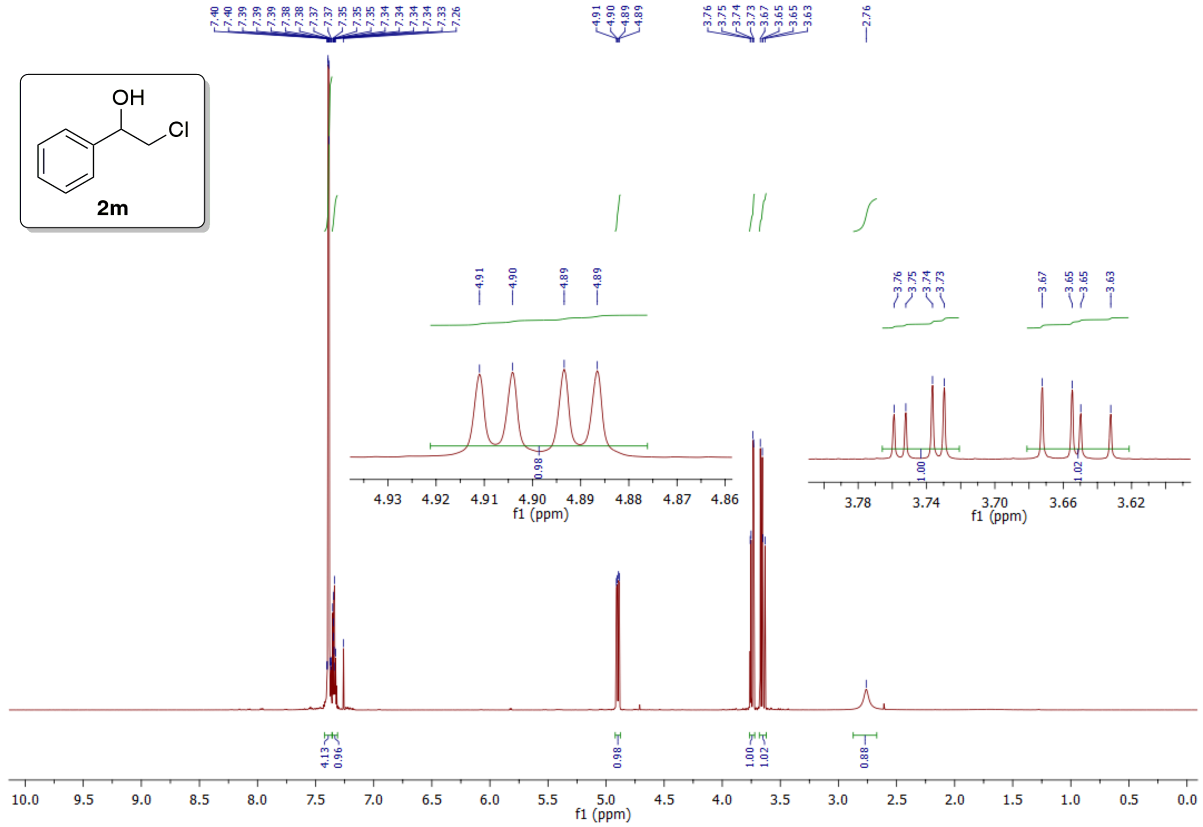

Supplementary Figure 54. $^{13}C\{^{1}H\}$ NMR spectrum of **2m** (126 MHz, $CDCl_3$)

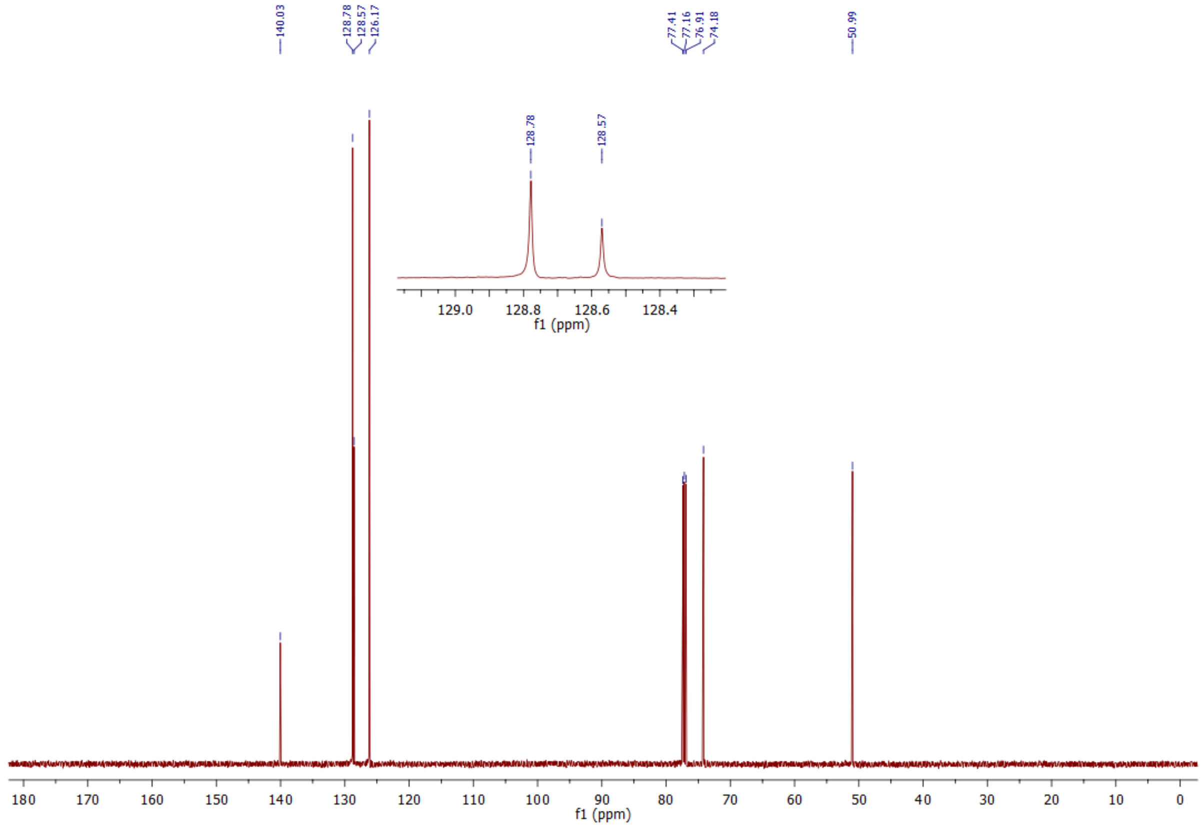

Supplementary Figure 55. FTMS spectrum of **2m** (ESI-TOF)

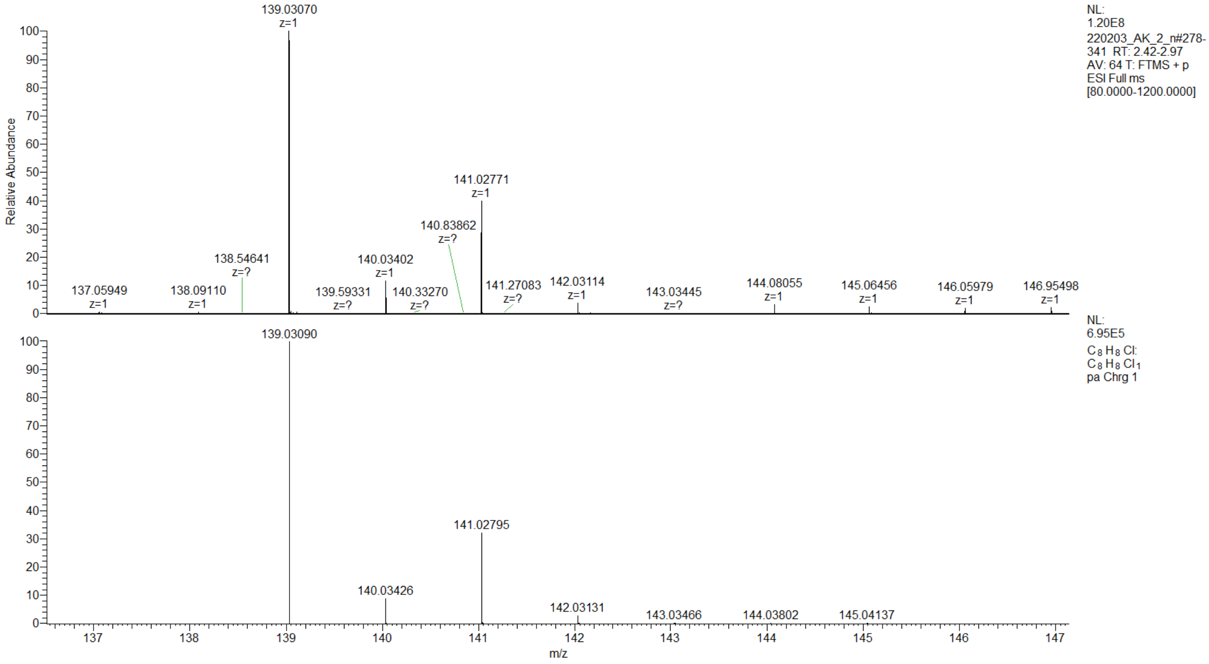

Supplementary Figure 56. ATR-FTIR spectrum of **2m** (neat)

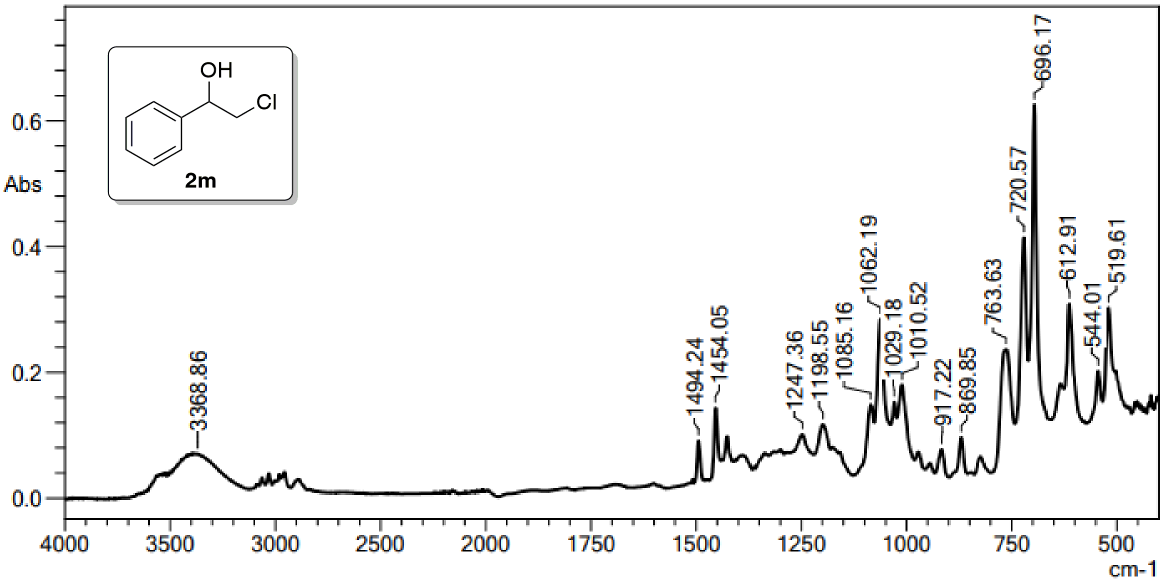

3-Chloro-1-phenylpropan-1-ol (2n)

Supplementary Figure 57. ^{1}H NMR spectrum of **2n** (500 MHz, $CDCl_3$)

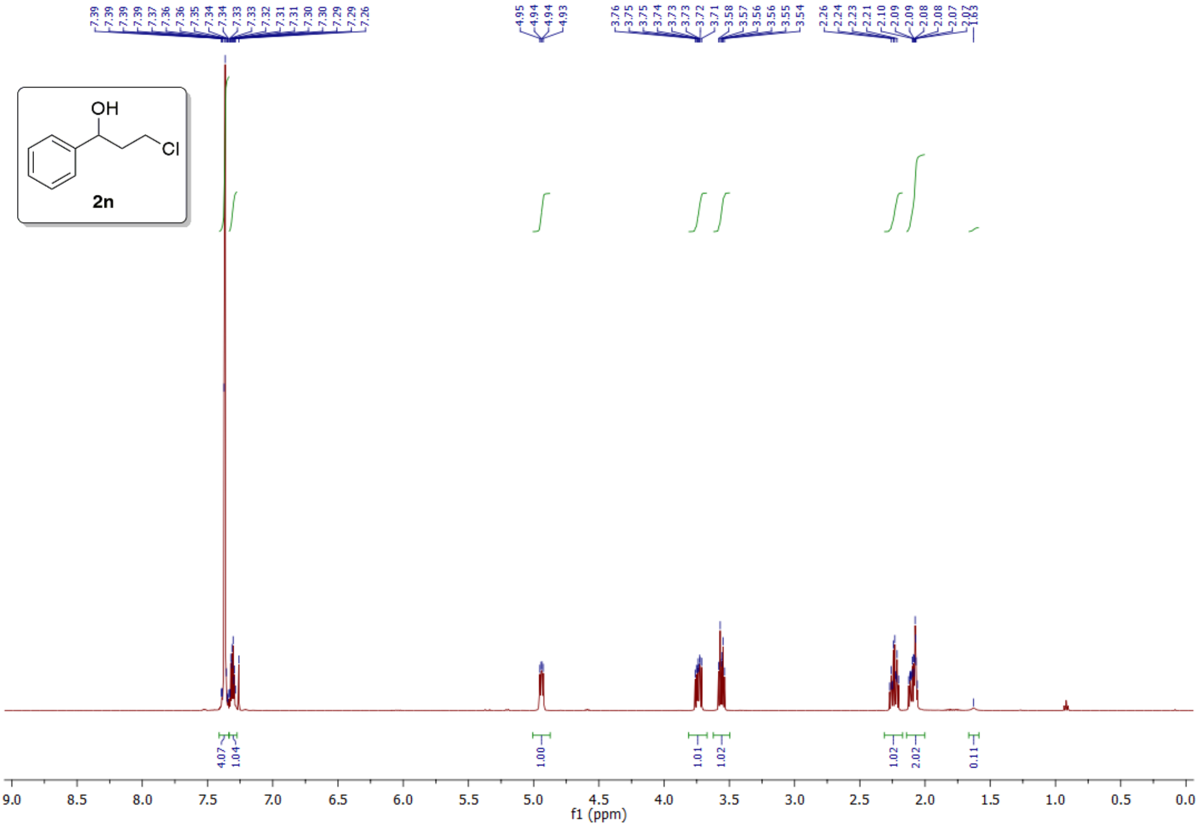

Supplementary Figure 58. $^{13}C\{^{1}H\}$ NMR spectrum of **2n** (126 MHz, $CDCl_3$)

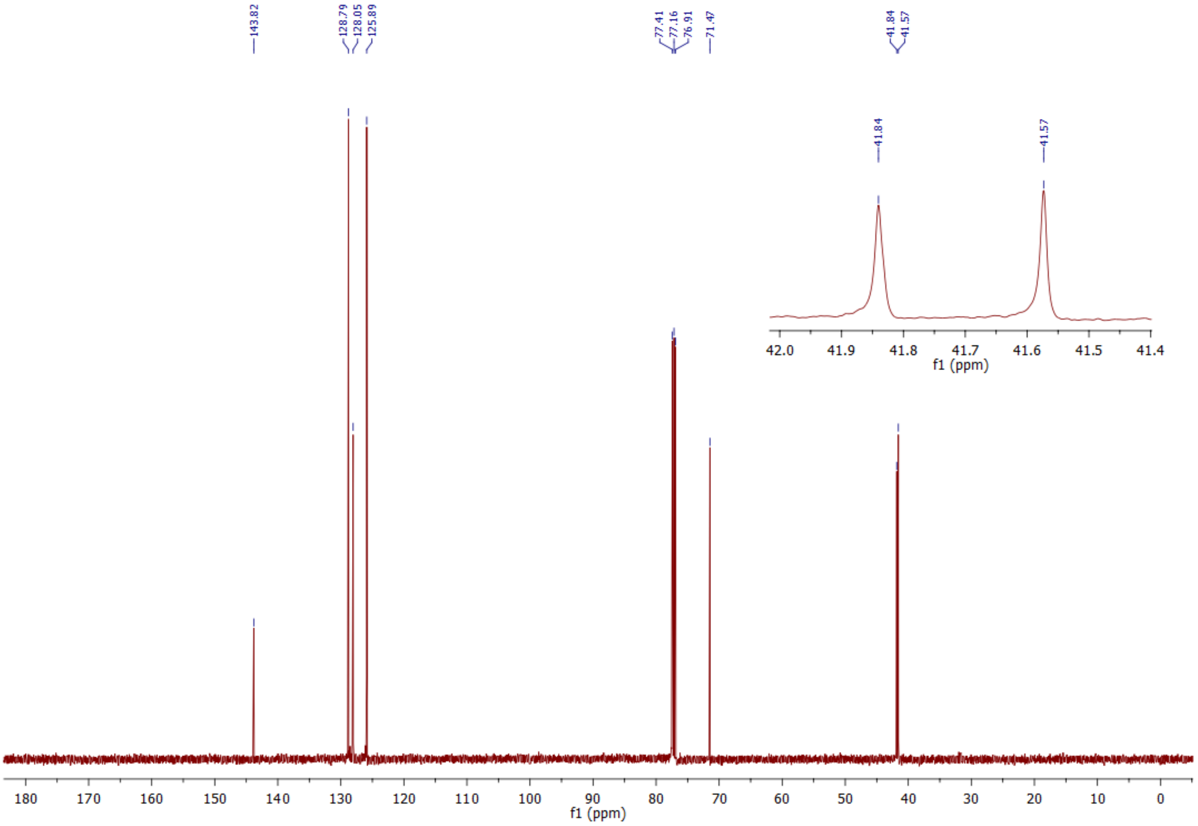

Supplementary Figure 59. FTMS spectrum of **2n** (ESI-TOF)

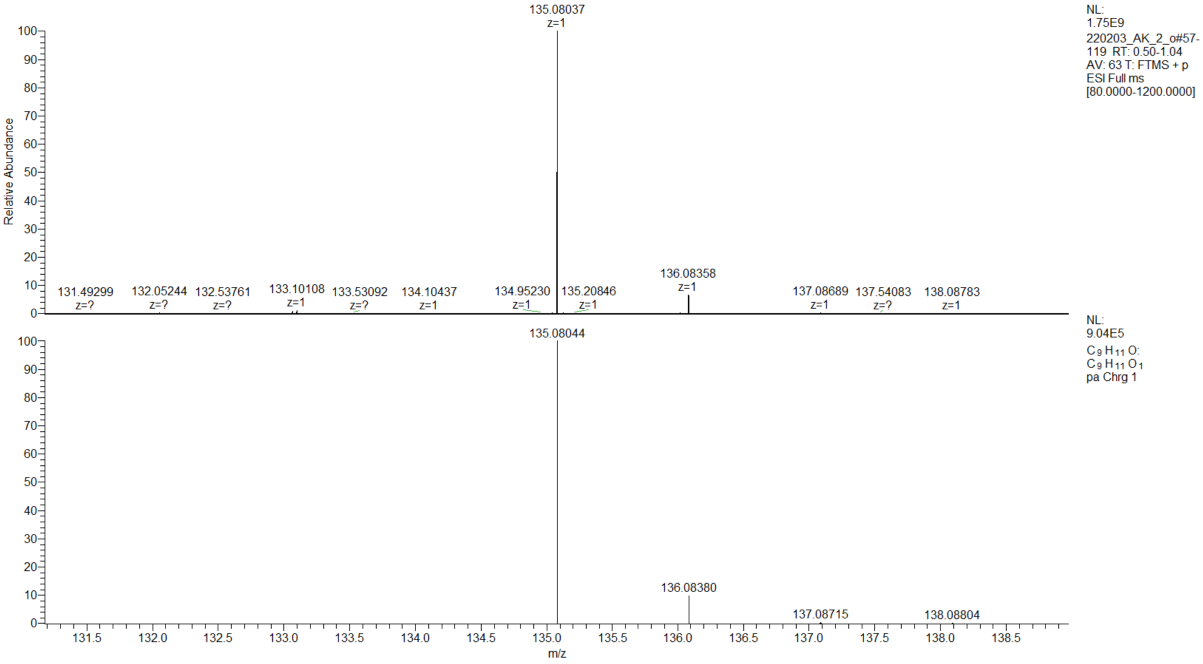

Supplementary Figure 60. ATR-FTIR spectrum of **2n** (neat)

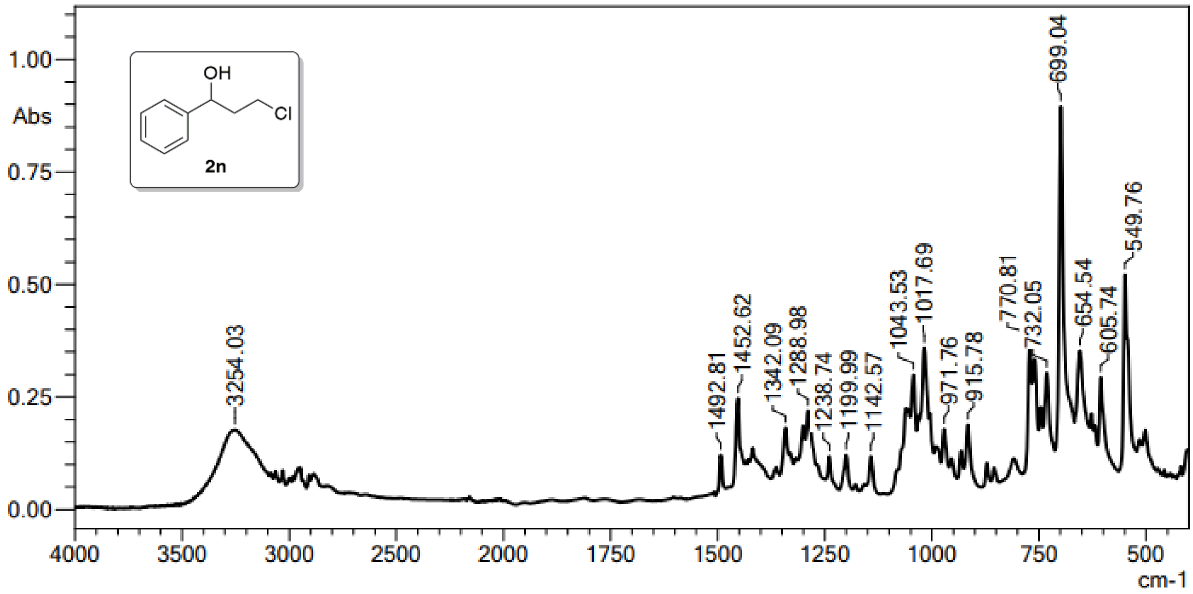

4-Chloro-1-phenylbutan-1-ol (2o)

Supplementary Figure 61. ^{1}H NMR spectrum of **2o** (500 MHz, $CDCl_3$)

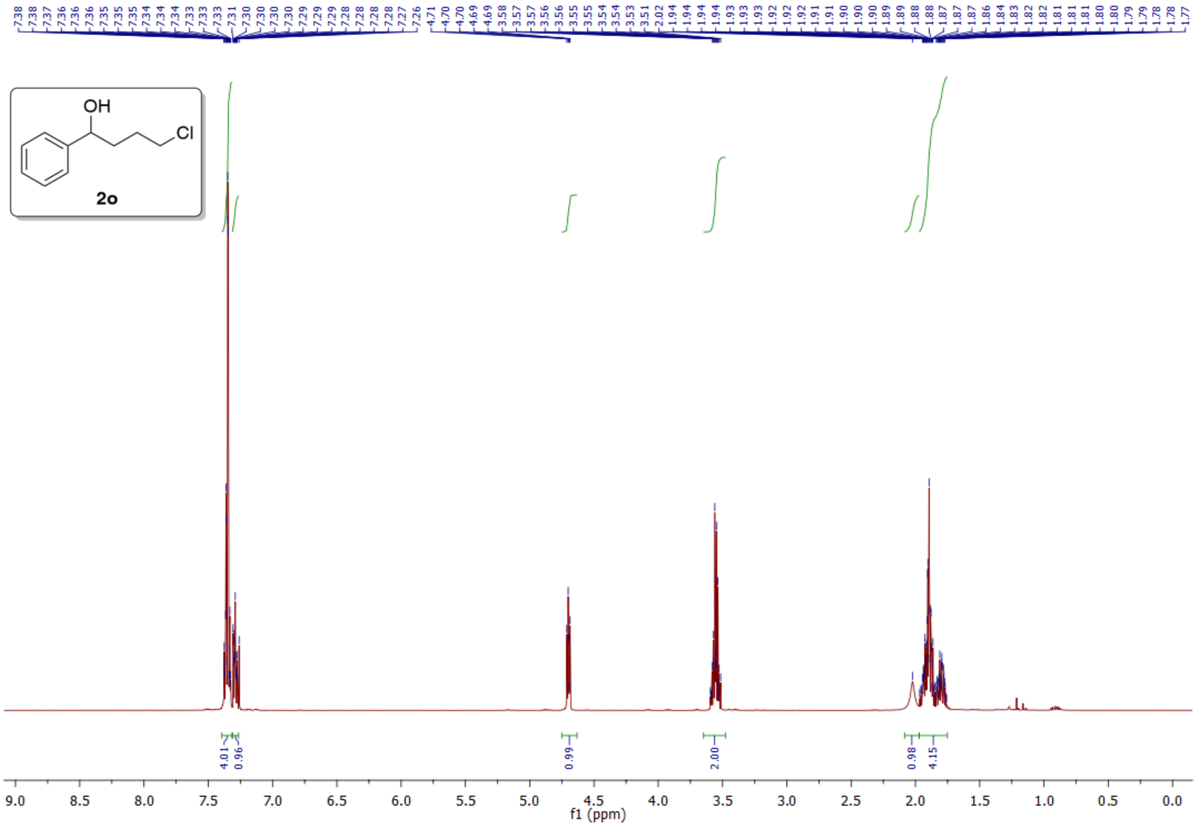

Supplementary Figure 62. $^{13}C\{^{1}H\}$ NMR spectrum of **2o** (126 MHz, $CDCl_3$)

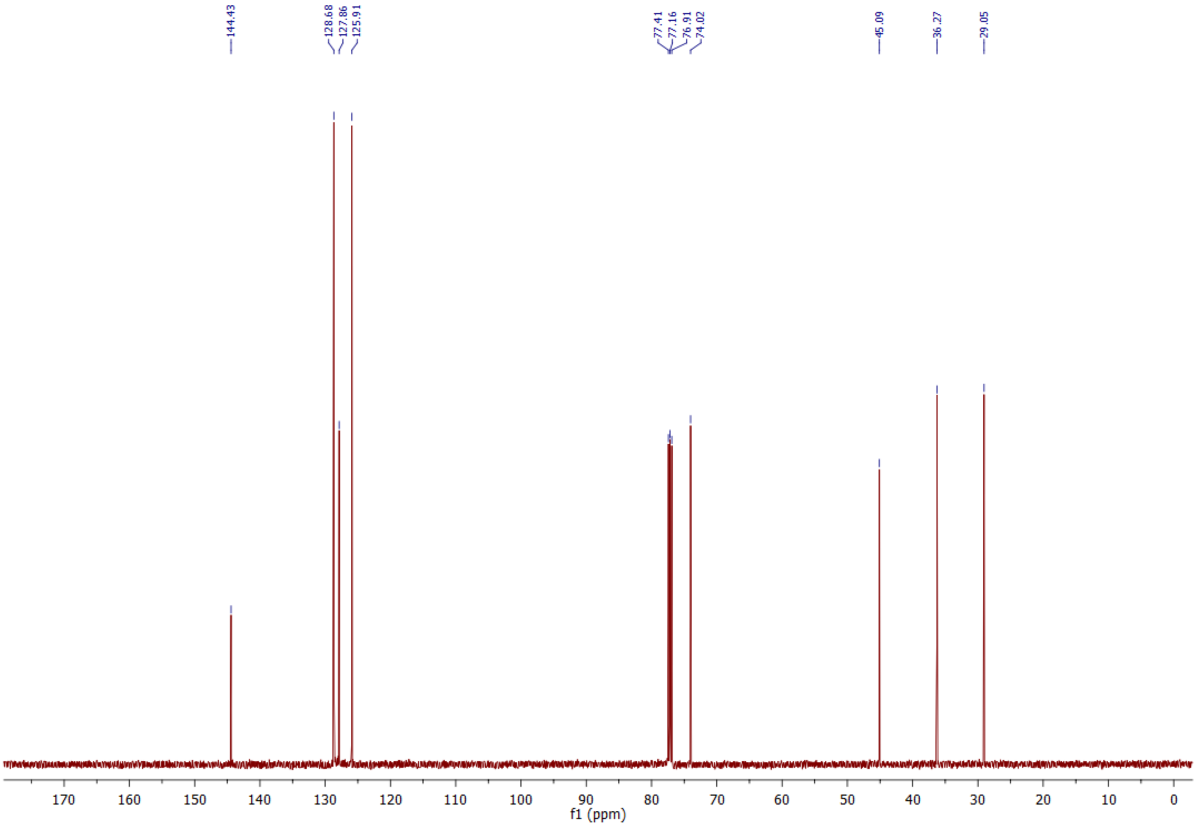

Supplementary Figure 63. FTMS spectrum of **2o** (ESI-TOF)

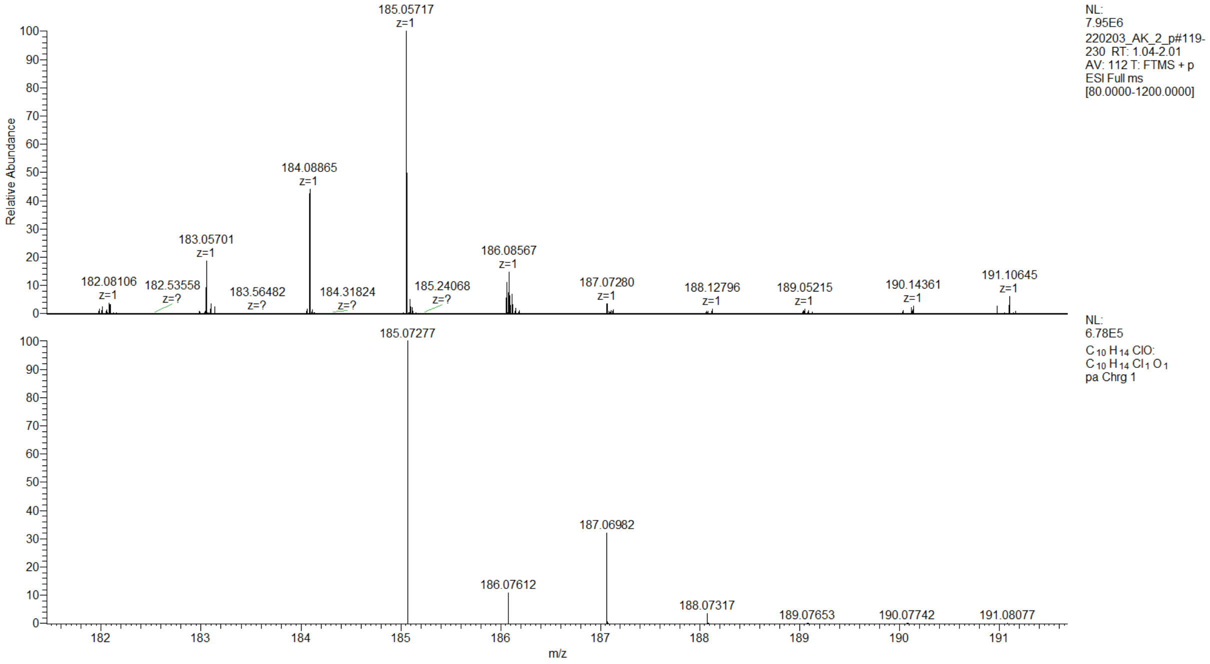

Supplementary Figure 64. ATR-FTIR spectrum of **2o** (neat)

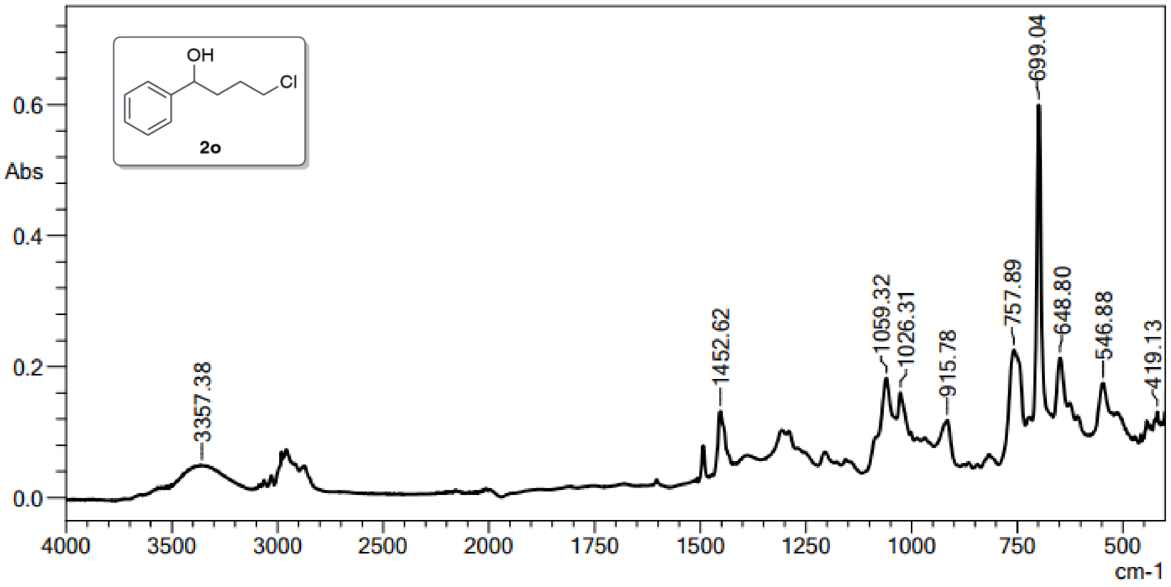

1-(Furan-2-yl)ethanol (2p)

Supplementary Figure 65. ^{1}H NMR spectrum of **2p** (500 MHz, $CDCl_3$)

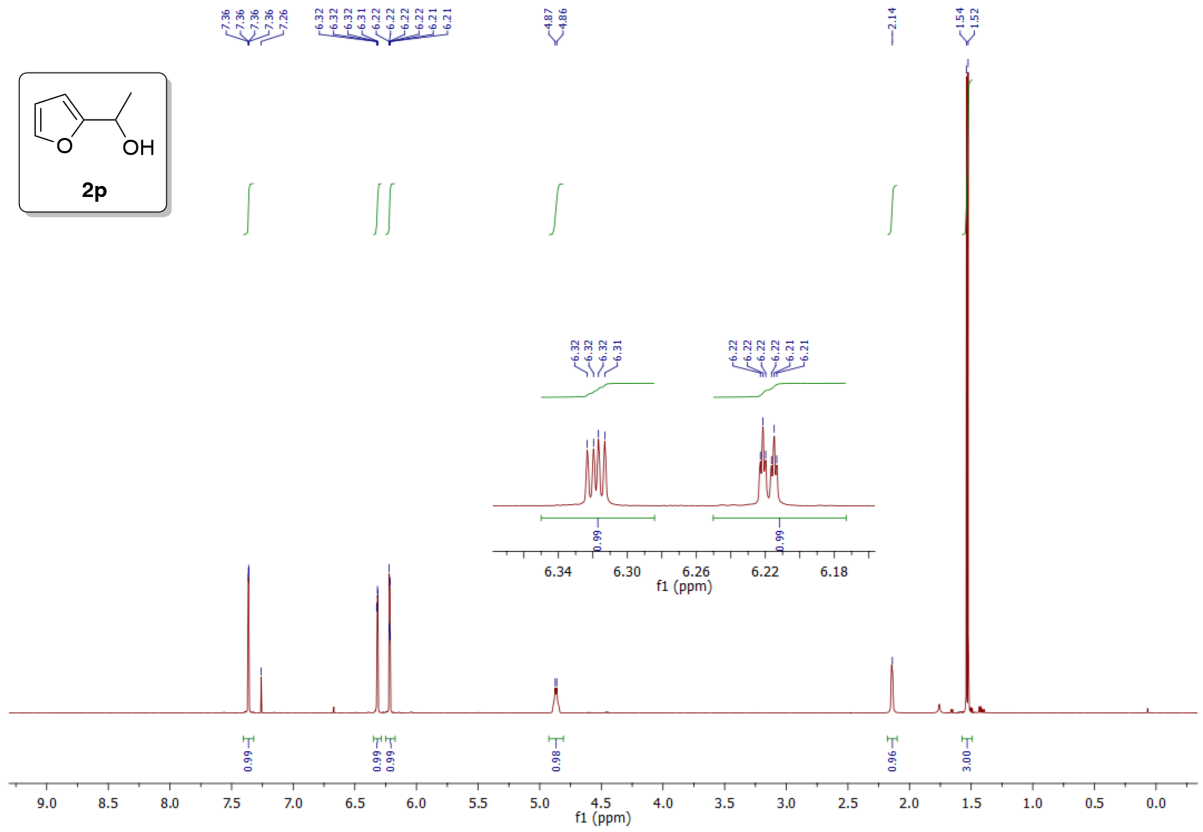

Supplementary Figure 66. ^{13}C{^{1}H} NMR spectrum of **2p** (126 MHz, $CDCl_3$)

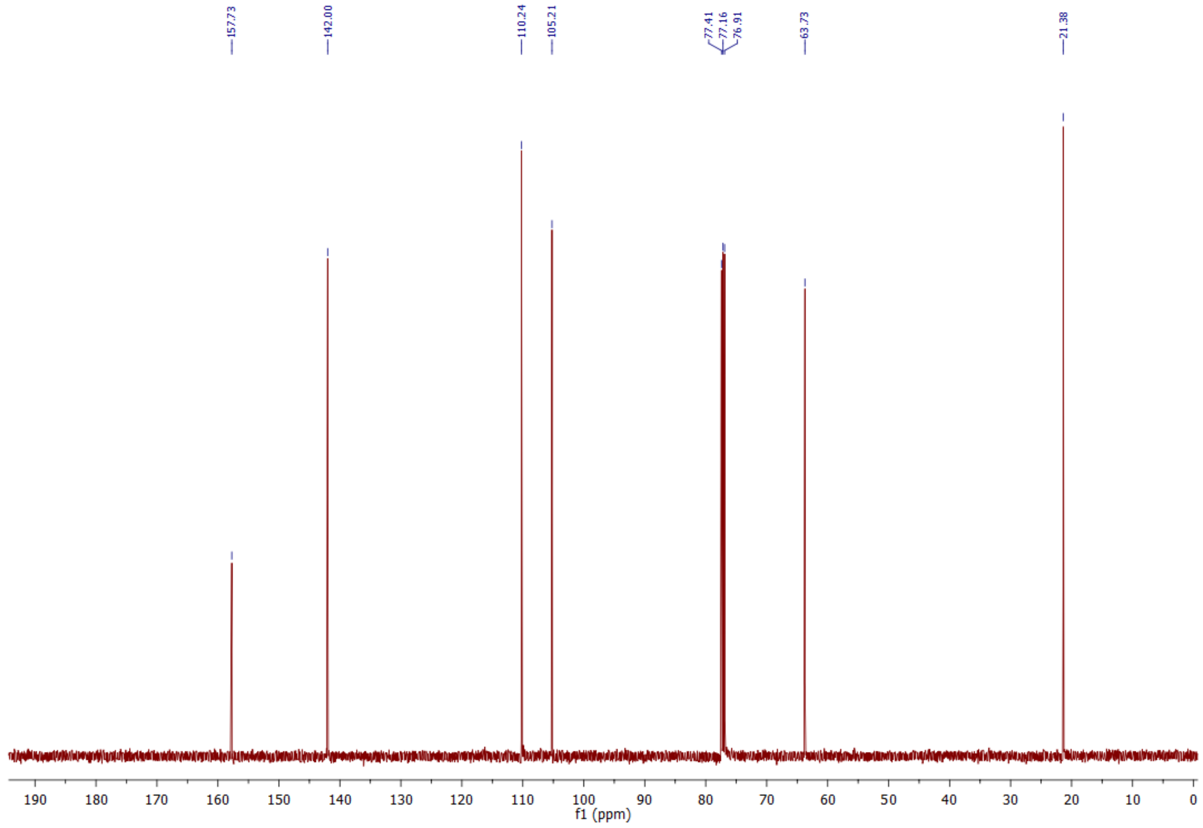

Supplementary Figure 67. FTMS spectrum of **2p** (ESI-TOF)

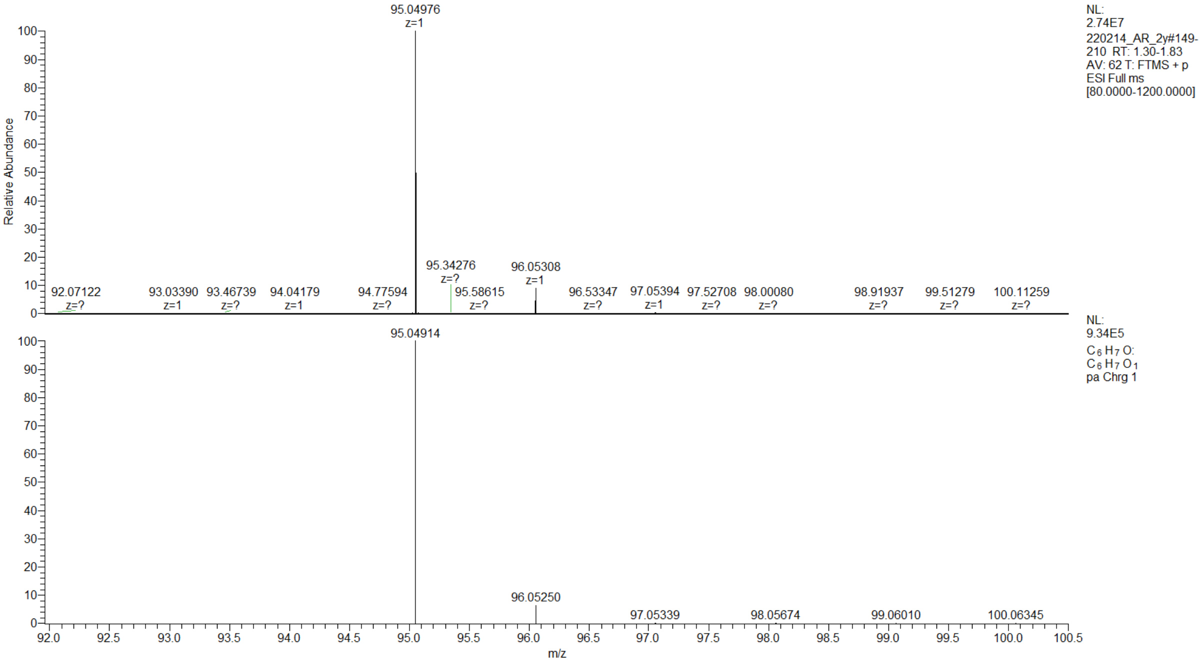

Supplementary Figure 68. ATR-FTIR spectrum of **2p** (neat)

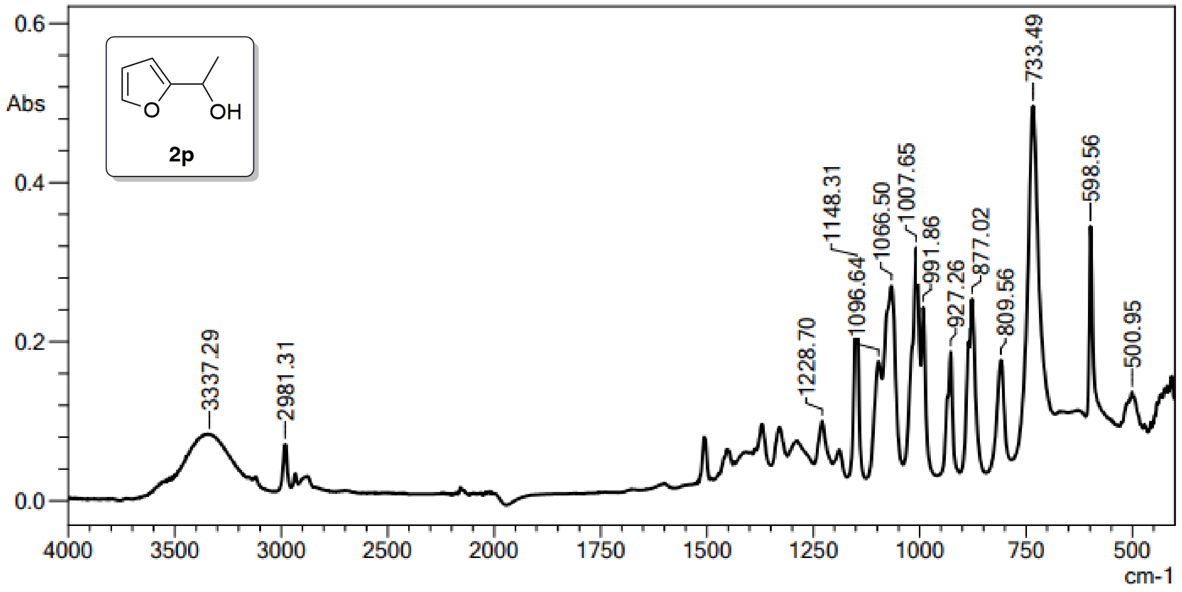

1-(Thiophen-2-yl)ethanol (2q)

Supplementary Figure 69. ^{1}H NMR spectrum of **2q** (500 MHz, $CDCl_3$)

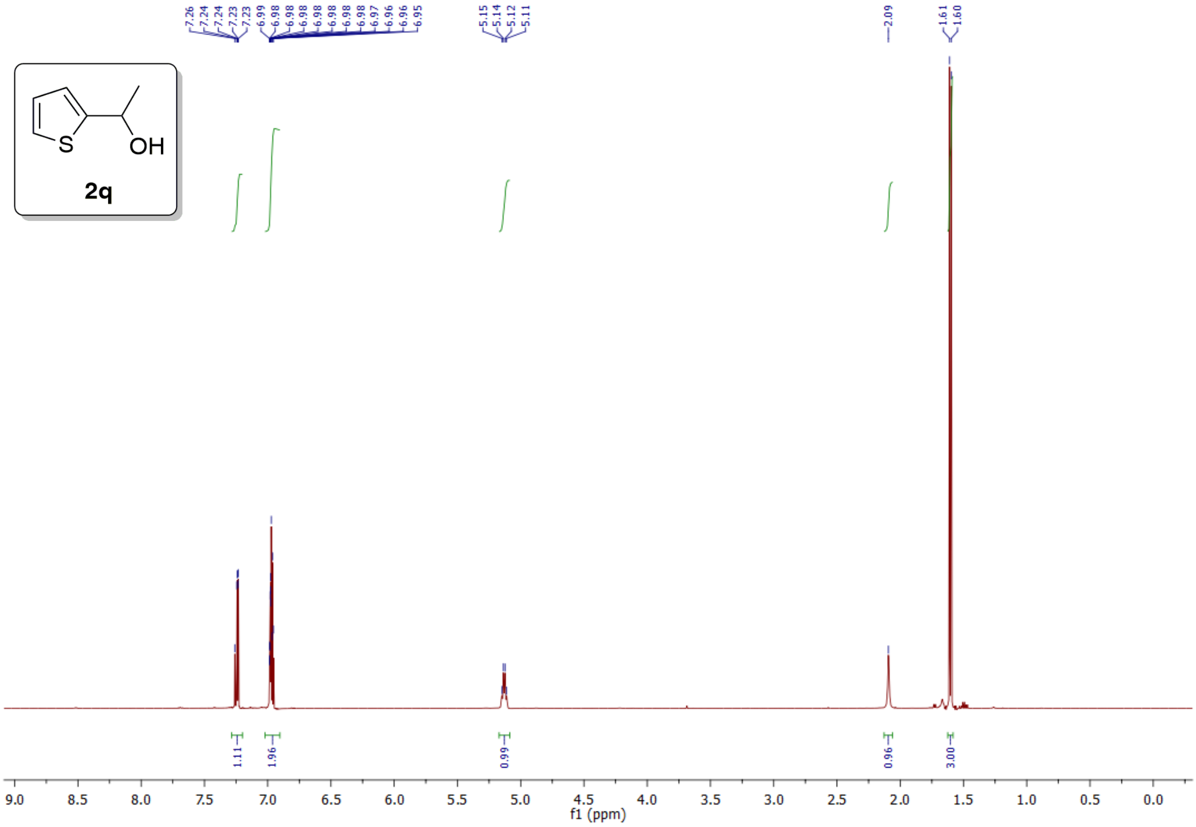

Supplementary Figure 70. $^{13}C\{^{1}H\}$ NMR spectrum of **2q** (126 MHz, $CDCl_3$)

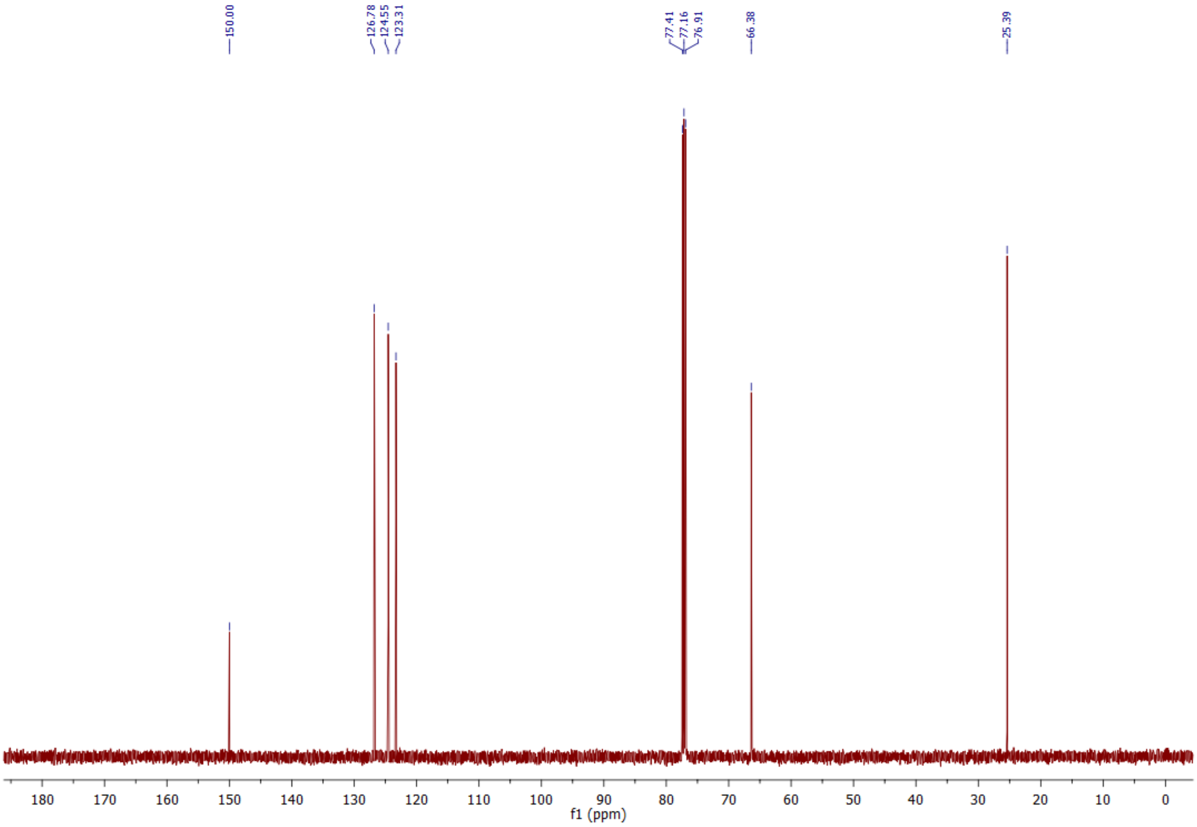

Supplementary Figure 71. FTMS spectrum of **2q** (ESI-TOF)

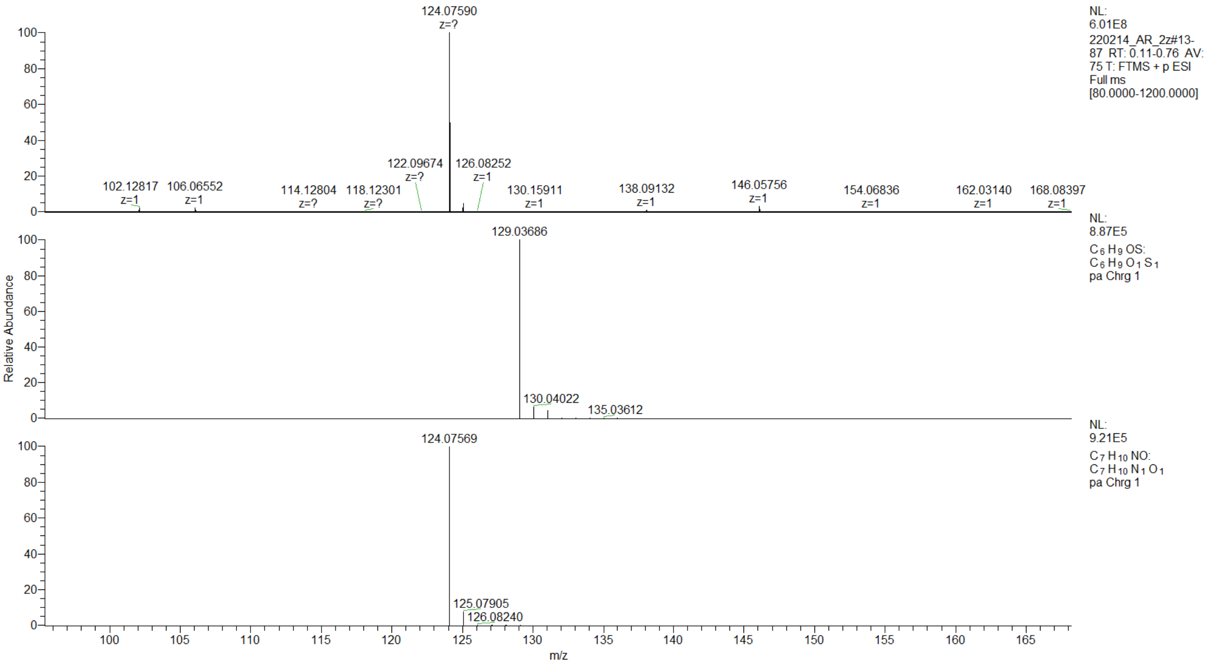

Supplementary Figure 72. ATR-FTIR spectrum of **2q** (neat)

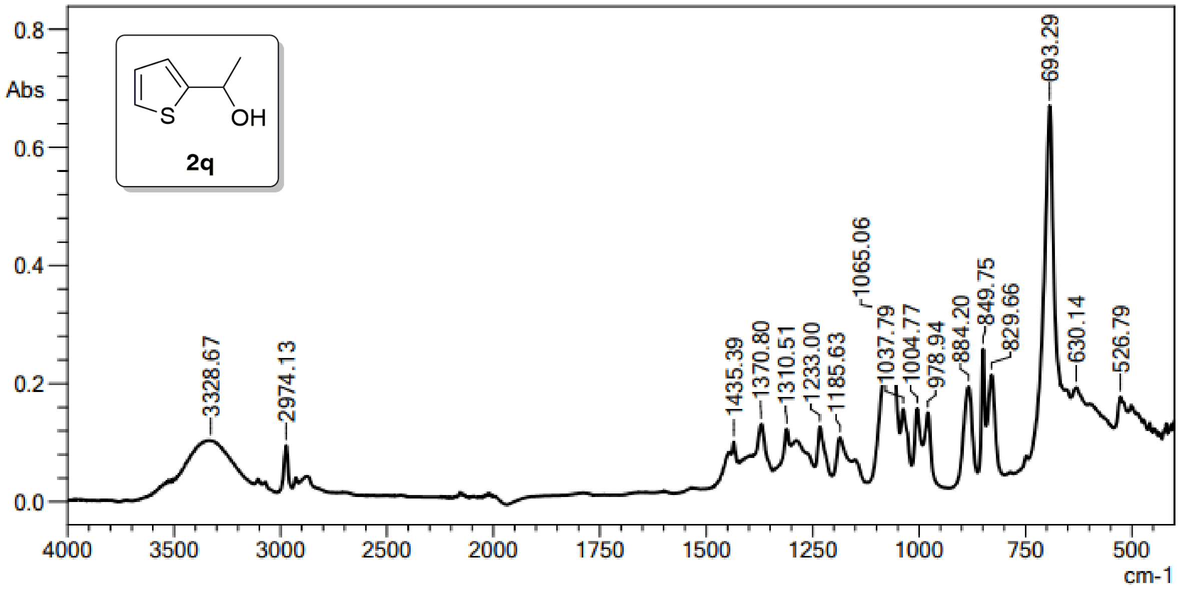

1-(Pyridin-2-yl)ethanol (2r)

Supplementary Figure 73. ^{1}H NMR spectrum of **2r** (500 MHz, $CDCl_3$)

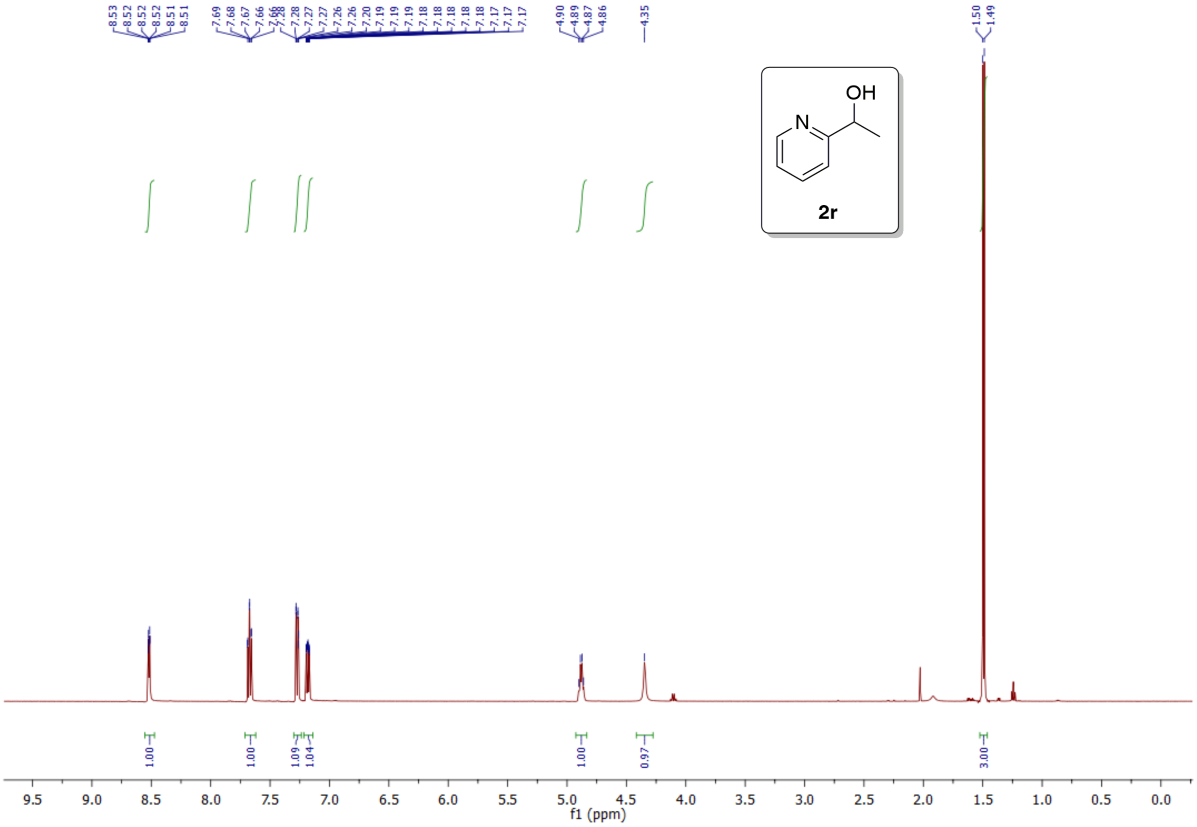

Supplementary Figure 74. $^{13}C\{^1H\}$ NMR spectrum of **2r** (126 MHz, $CDCl_3$)

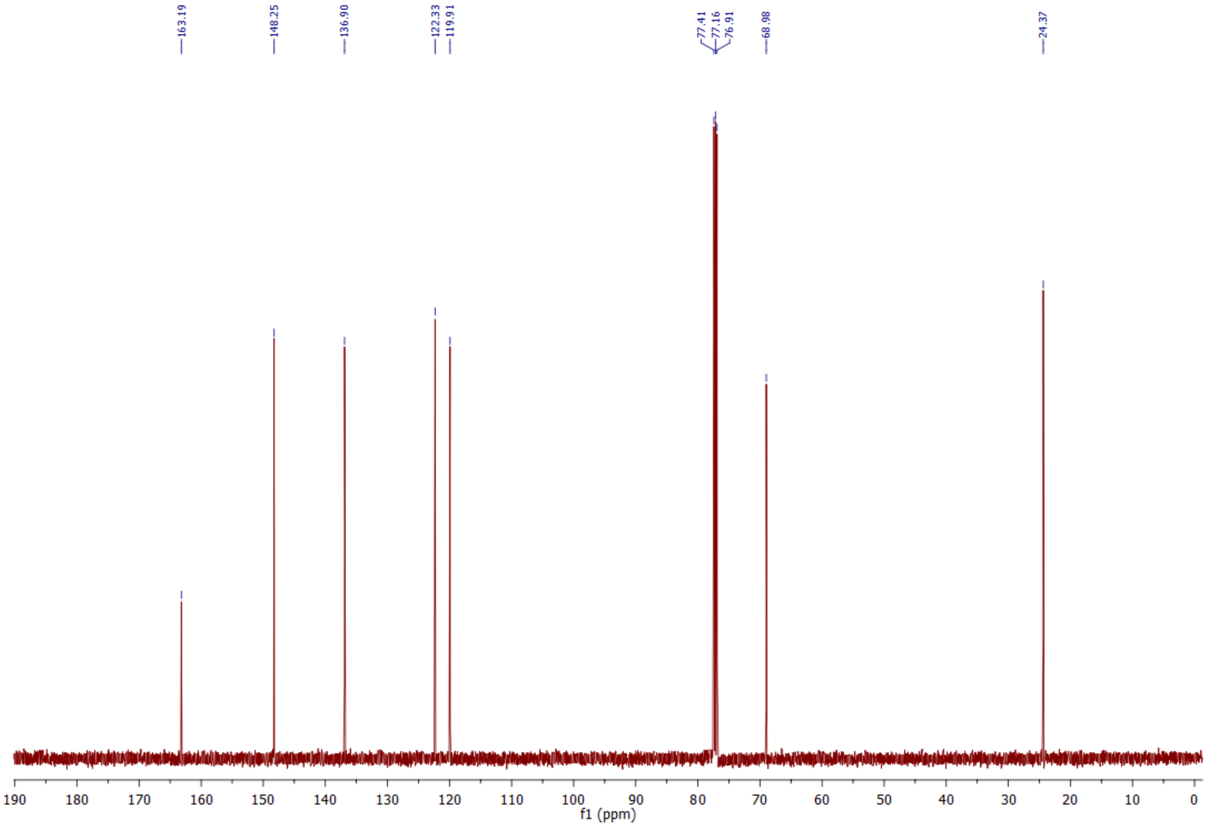

Supplementary Figure 75. FTMS spectrum of **2r** (ESI-TOF)

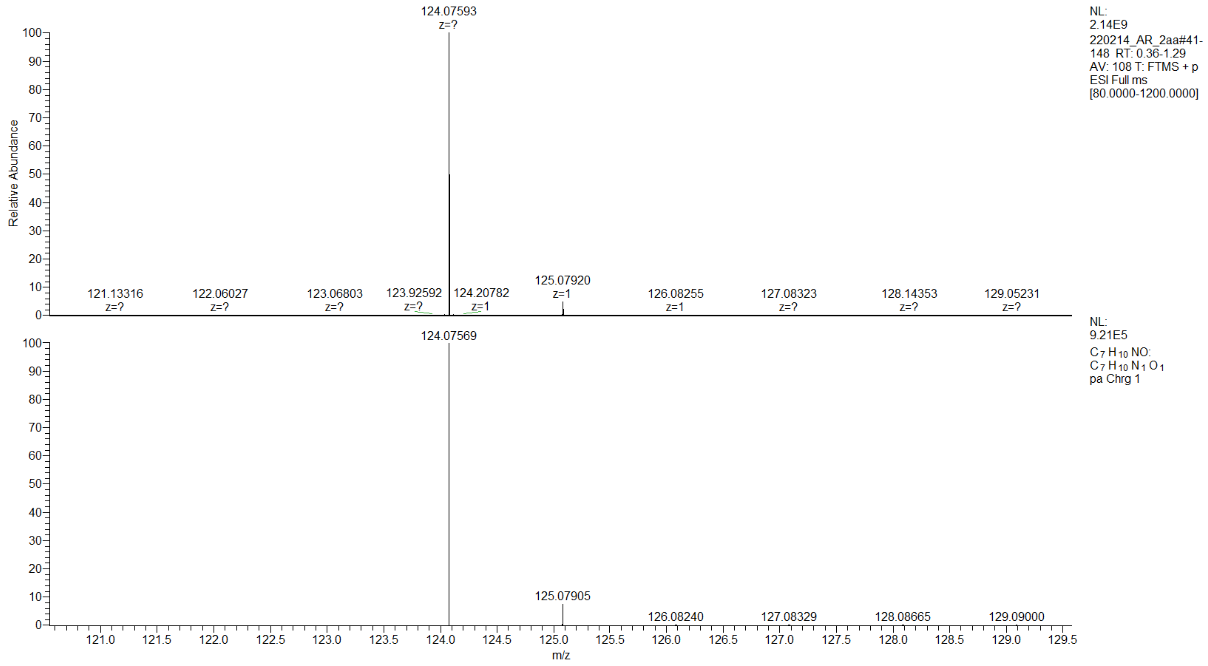

Supplementary Figure 76. ATR-FTIR spectrum of **2r** (neat)

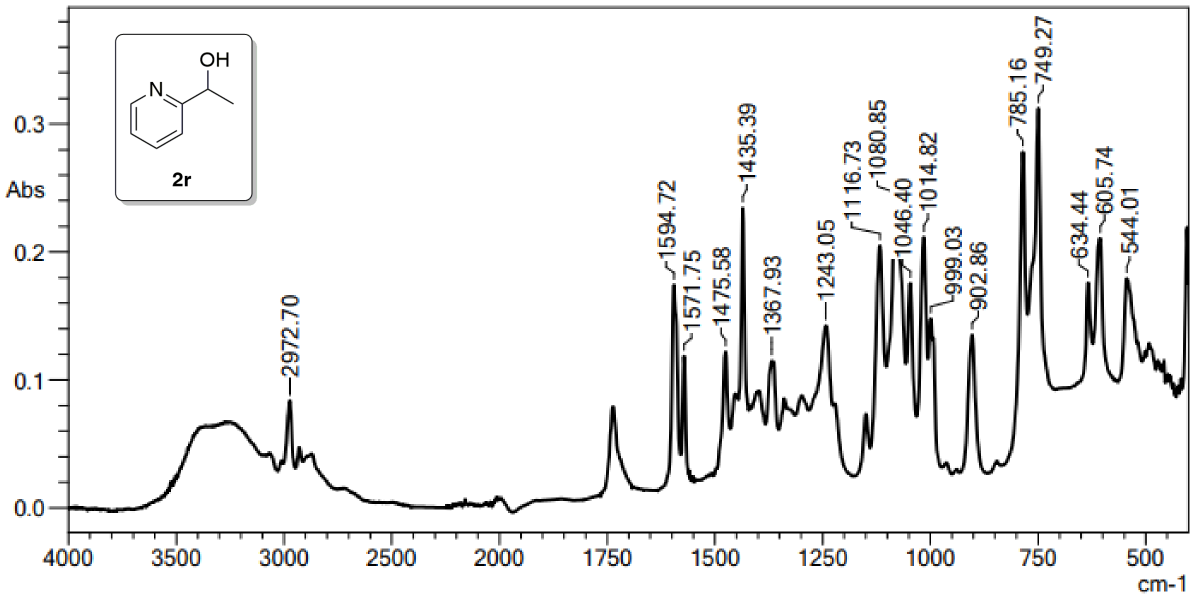

1-(1,3-Benzothiazol-2-ylsulfanyl)propan-2-ol (2s)

Supplementary Figure 77. ^{1}H NMR spectrum of **2s** (500 MHz, $CDCl_3$)

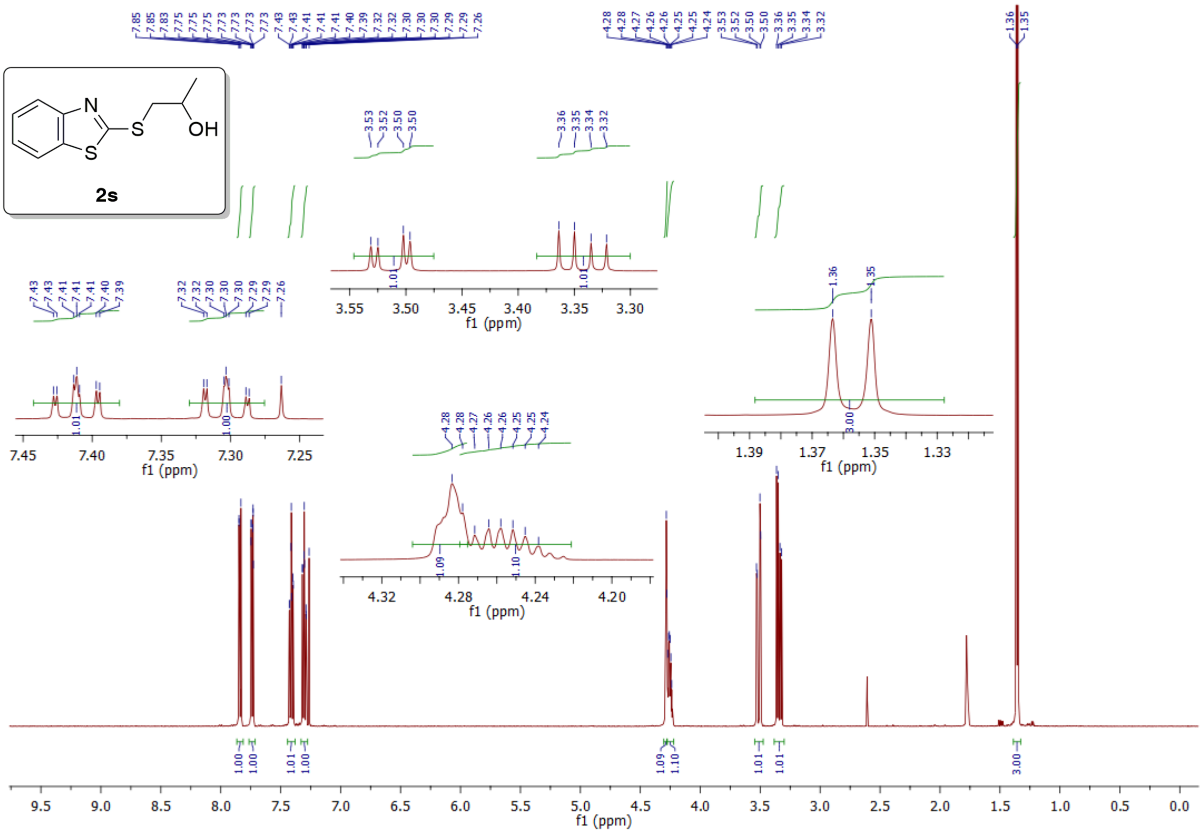

Supplementary Figure 78. $^{13}C\{^{1}H\}$ NMR spectrum of **2s** (126 MHz, $CDCl_3$)

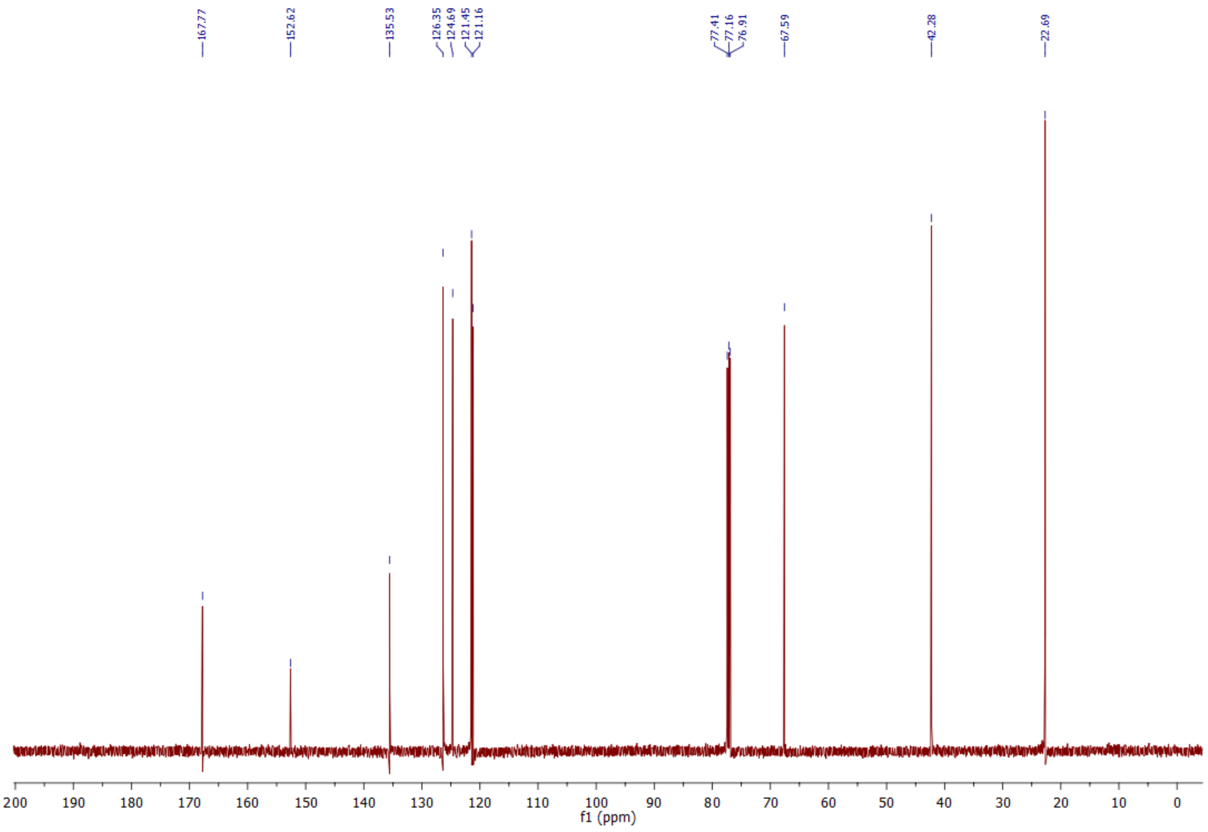

Supplementary Figure 79. FTMS spectrum of **2s** (ESI-TOF)

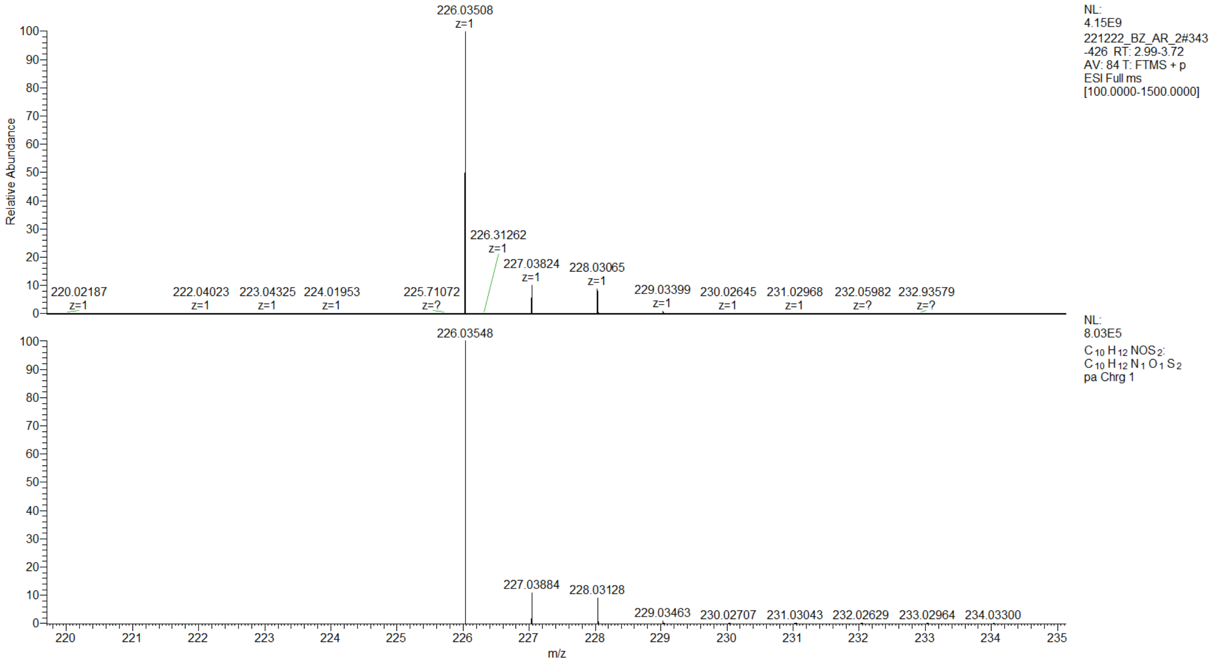

Supplementary Figure 80. ATR-FTIR spectrum of **2s** (neat)

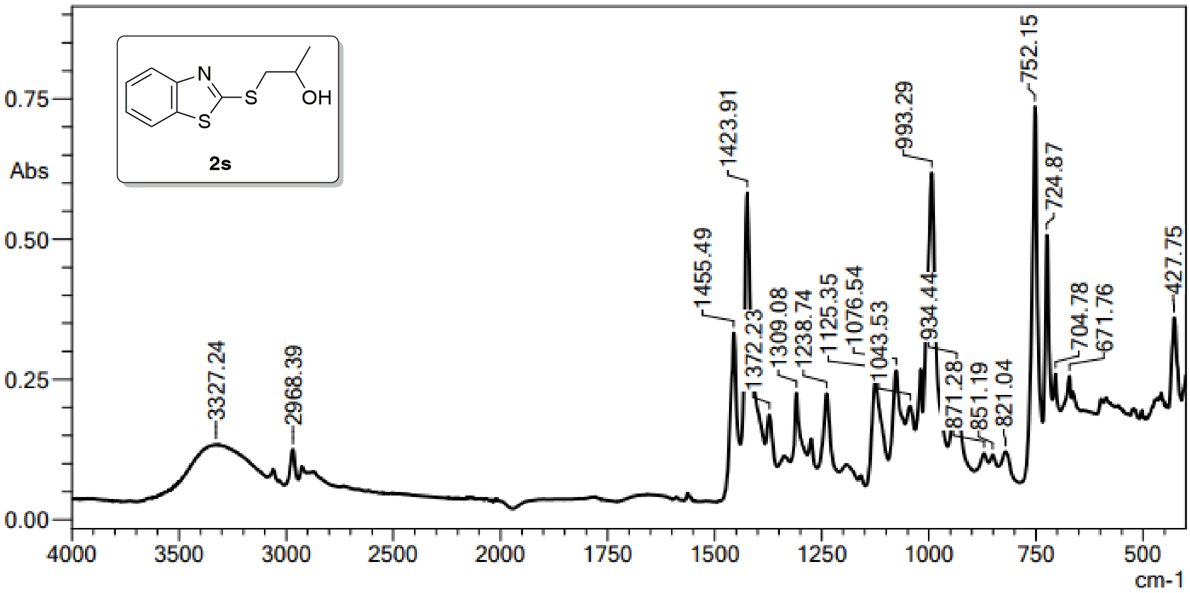

1-(10H-Phenothiazin-10-yl)propan-2-ol (2t)

Supplementary Figure 81. ^{1}H NMR spectrum of **2t** (500 MHz, $CDCl_3$)

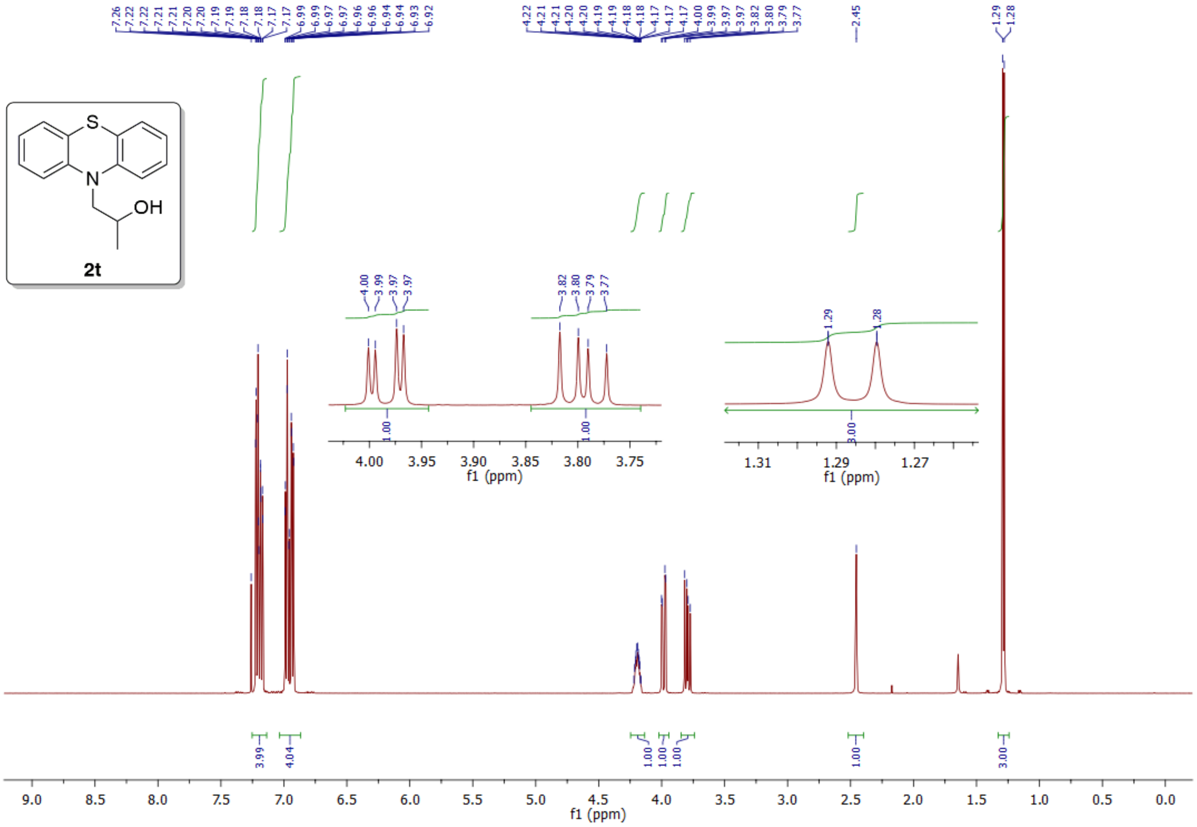

Supplementary Figure 82. ^{13}C{^{1}H} NMR spectrum of **2t** (126 MHz, $CDCl_3$)

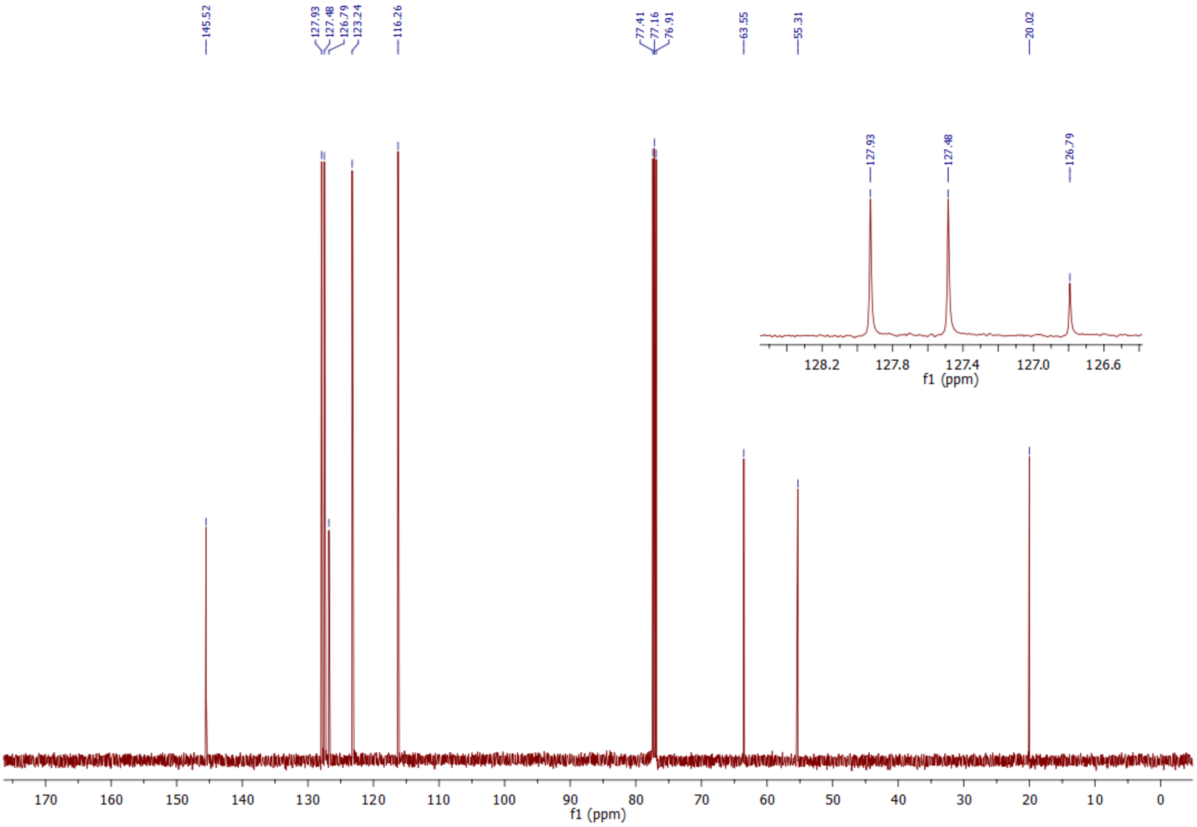

Supplementary Figure 83. FTMS spectrum of **2t** (ESI-TOF)

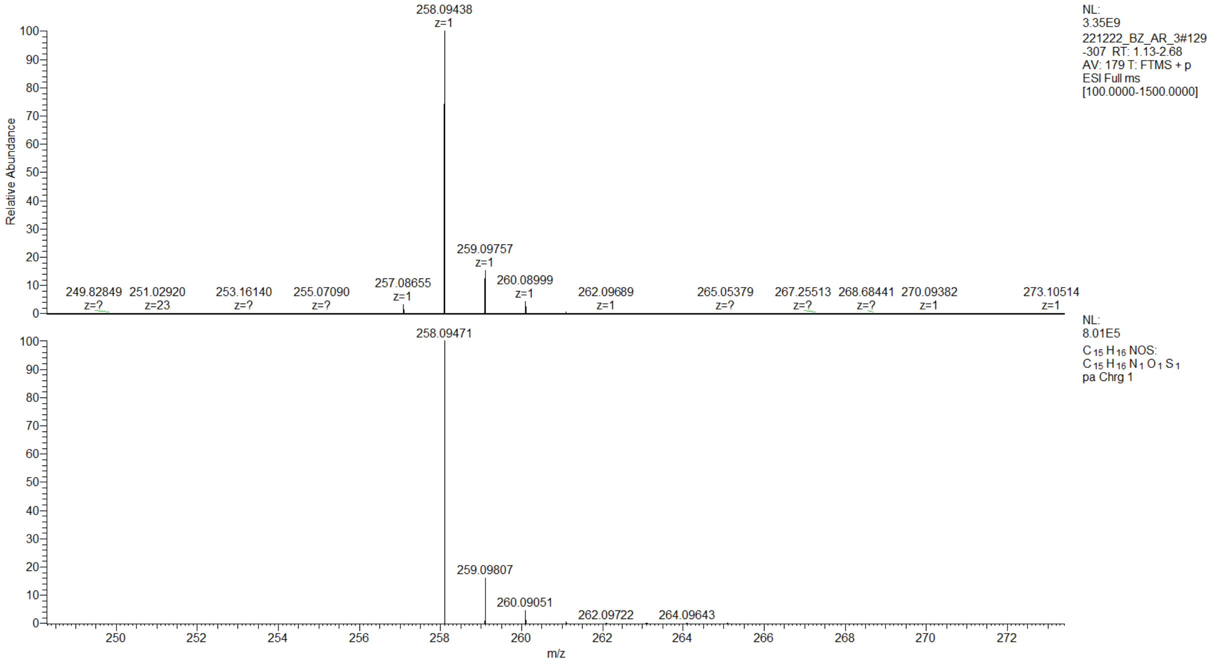

Supplementary Figure 84. ATR-FTIR spectrum of **2t** (neat)

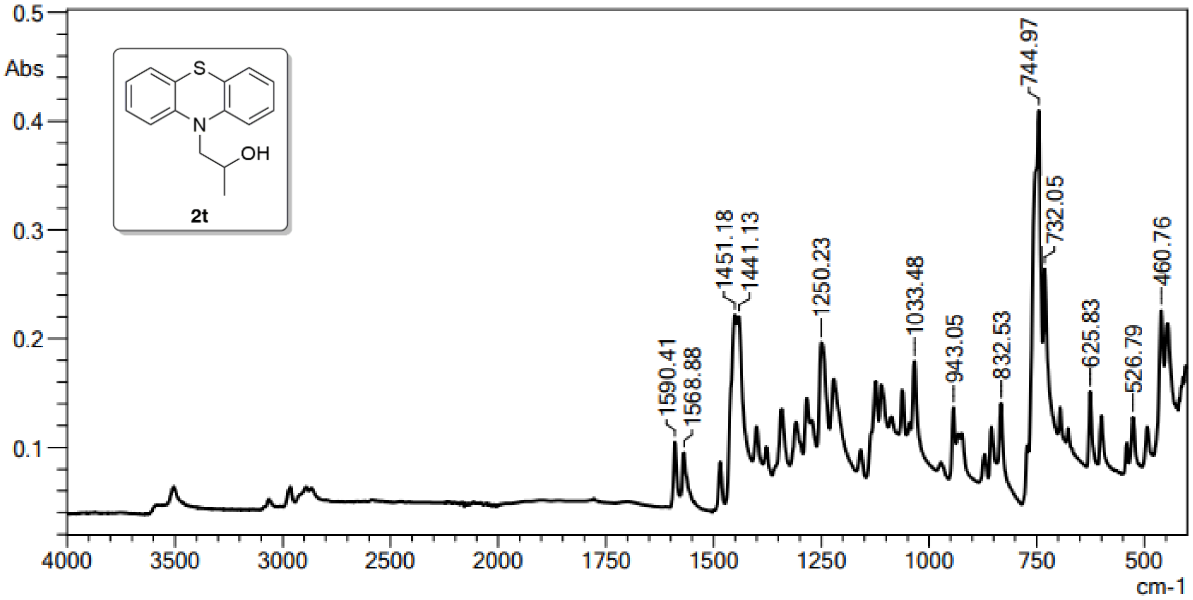

2-(2-Hydroxypropyl)-1H-isoindole-1,3(2H)-dione (2u)

Supplementary Figure 85. ^{1}H NMR spectrum of **2u** (500 MHz, $CDCl_3$)

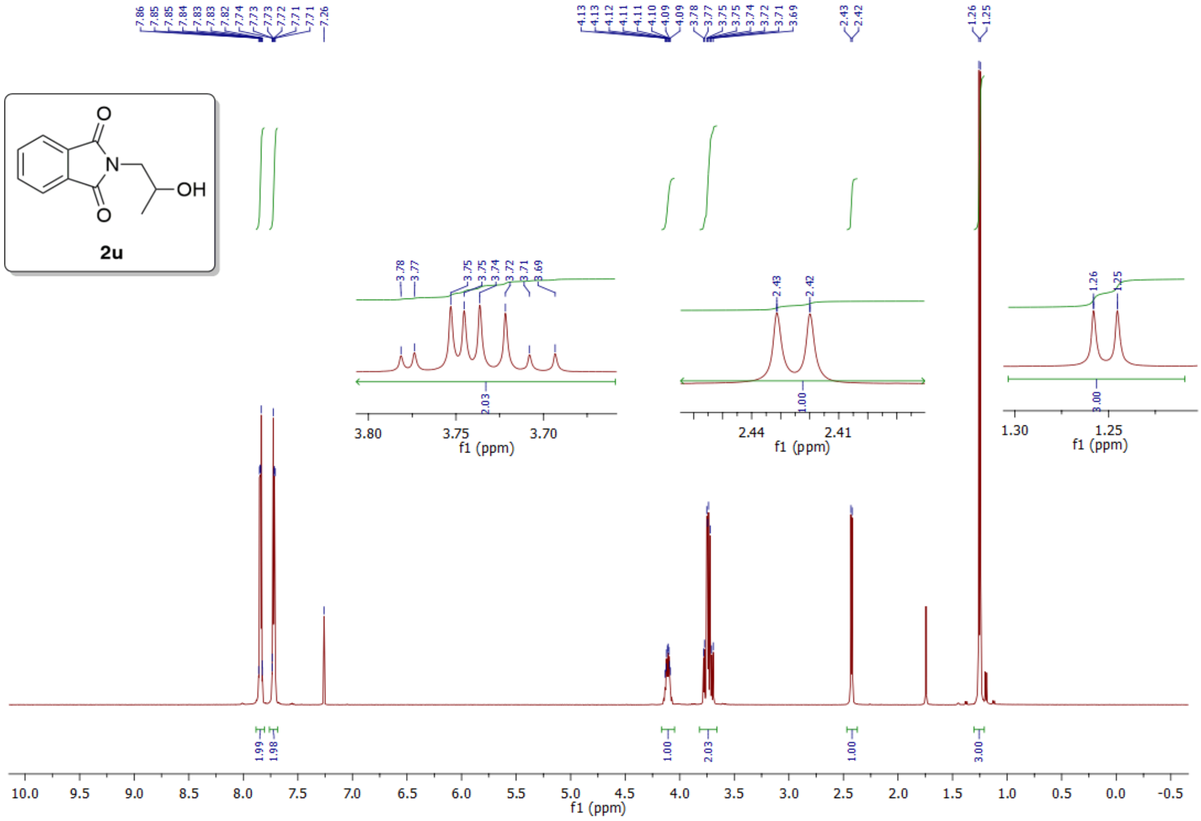

Supplementary Figure 86. ^{13}C{^{1}H} NMR spectrum of **2u** (126 MHz, $CDCl_3$)

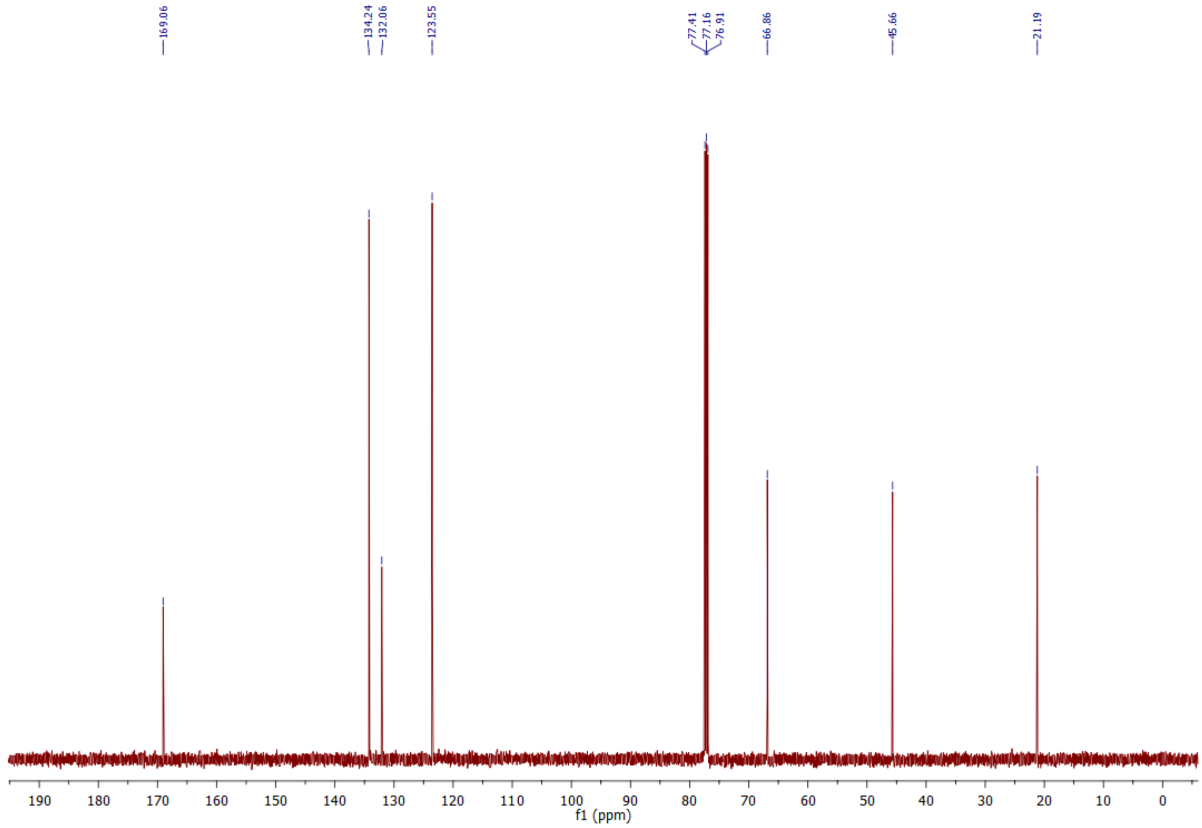

Supplementary Figure 87. FTMS spectrum of **2u** (ESI-TOF)

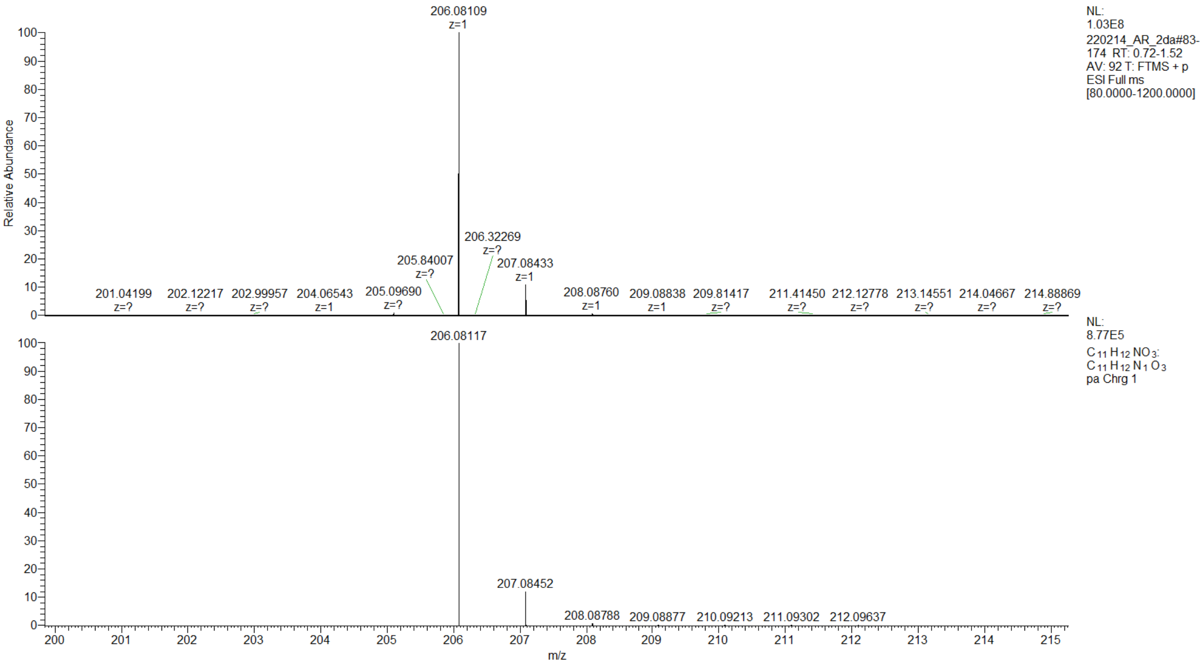

Supplementary Figure 88. ATR-FTIR spectrum of **2u** (neat)

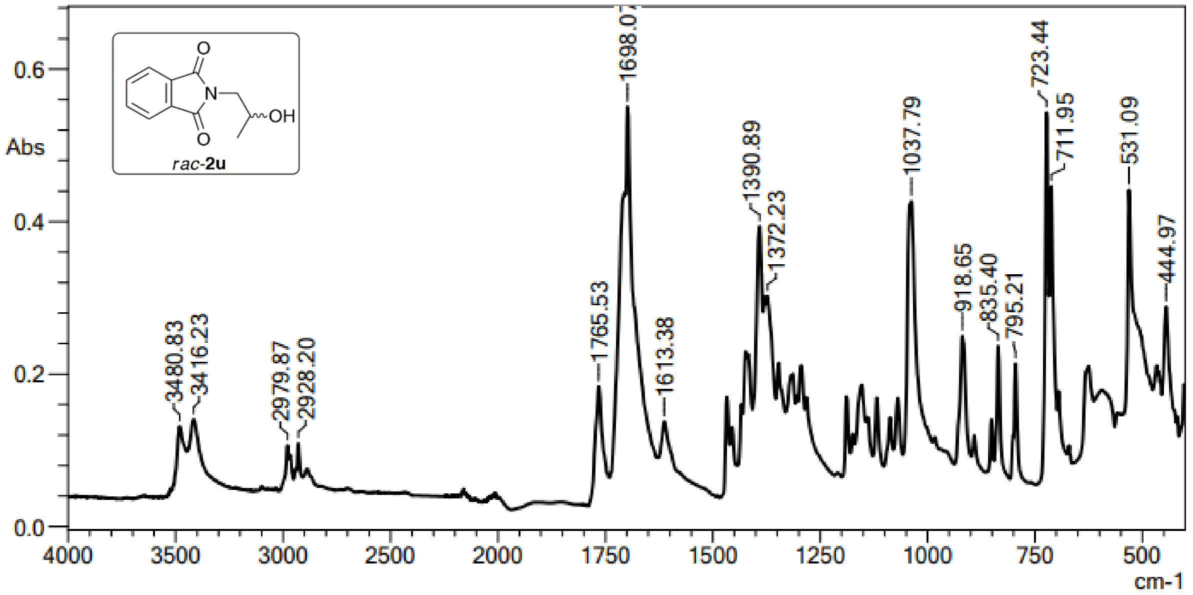

7-(2-Hydroxypropyl)-1,3-dimethyl-3,7-dihydro-1H-purine-2,6-dione (2v)

Supplementary Figure 89. ^{1}H NMR spectrum of **2v** (500 MHz, $CDCl_3$)

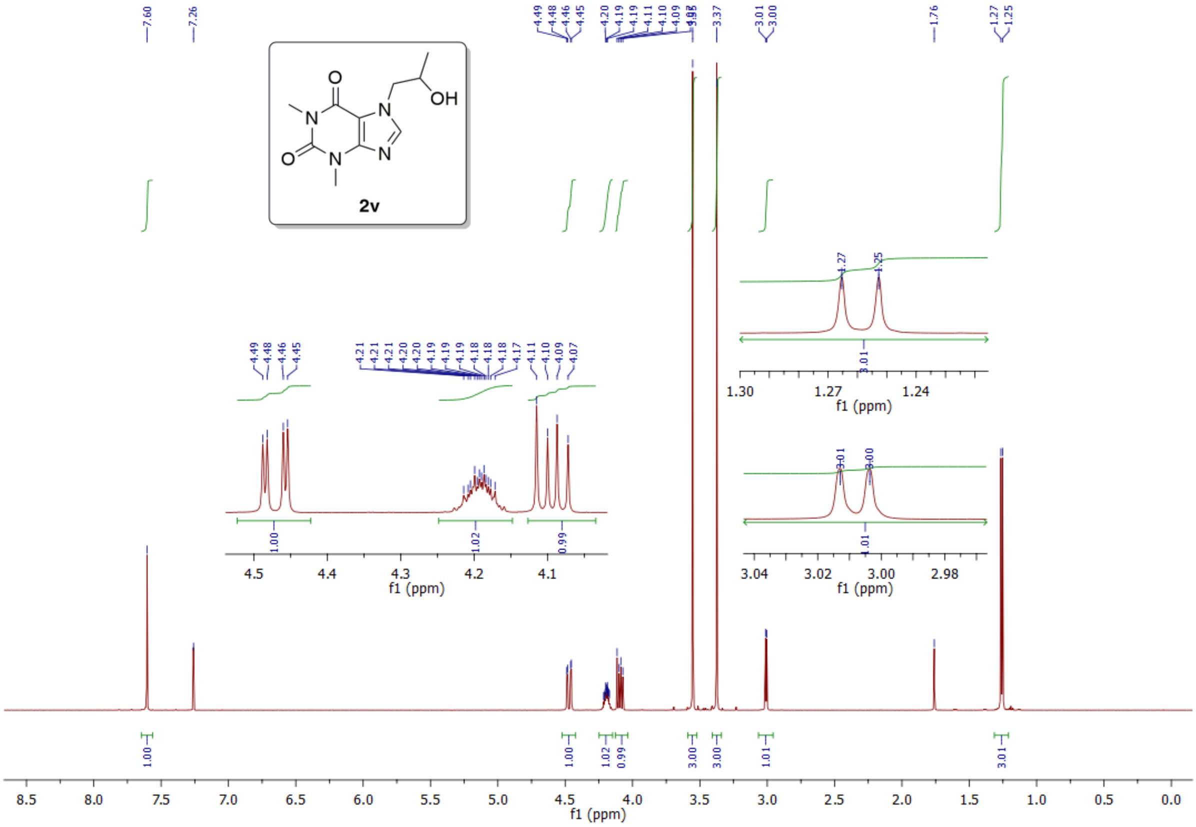

Supplementary Figure 90. $^{13}C\{^{1}H\}$ NMR spectrum of **2v** (126 MHz, $CDCl_3$)

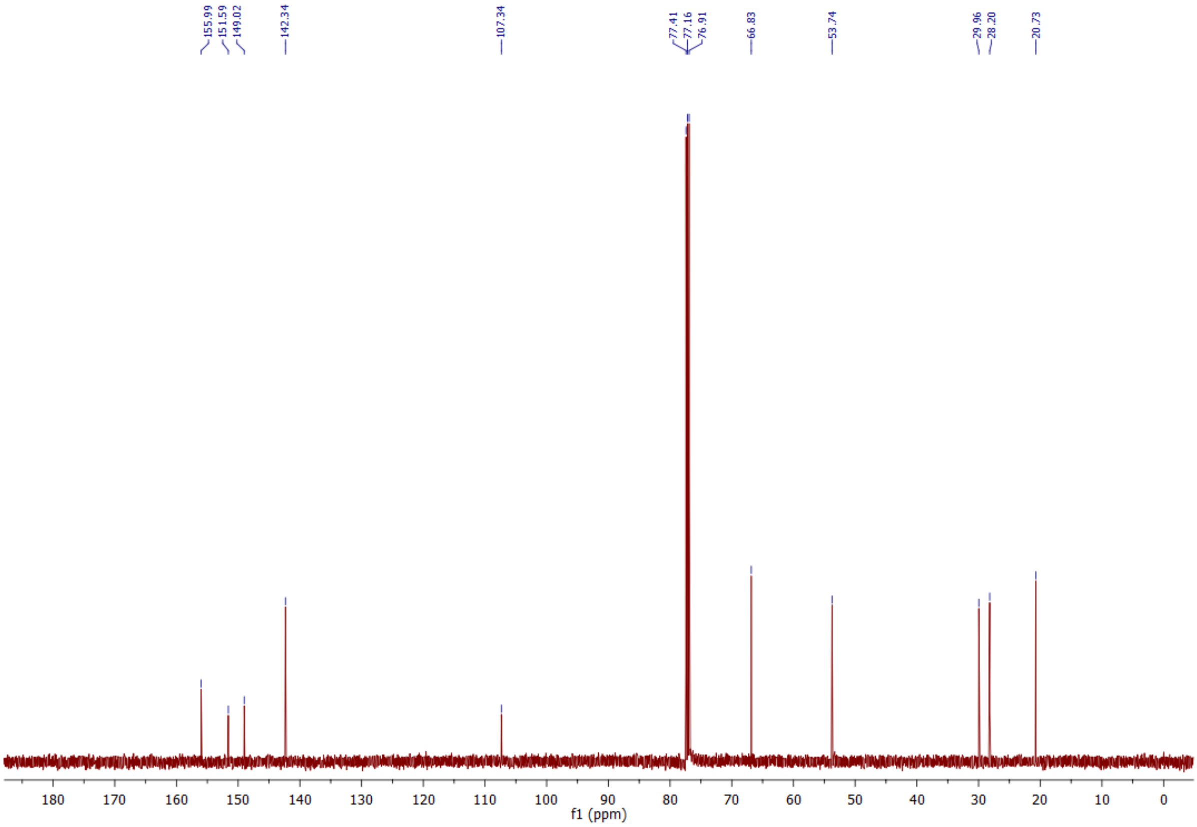

Supplementary Figure 91. FTMS spectrum of **2v** (ESI-TOF)

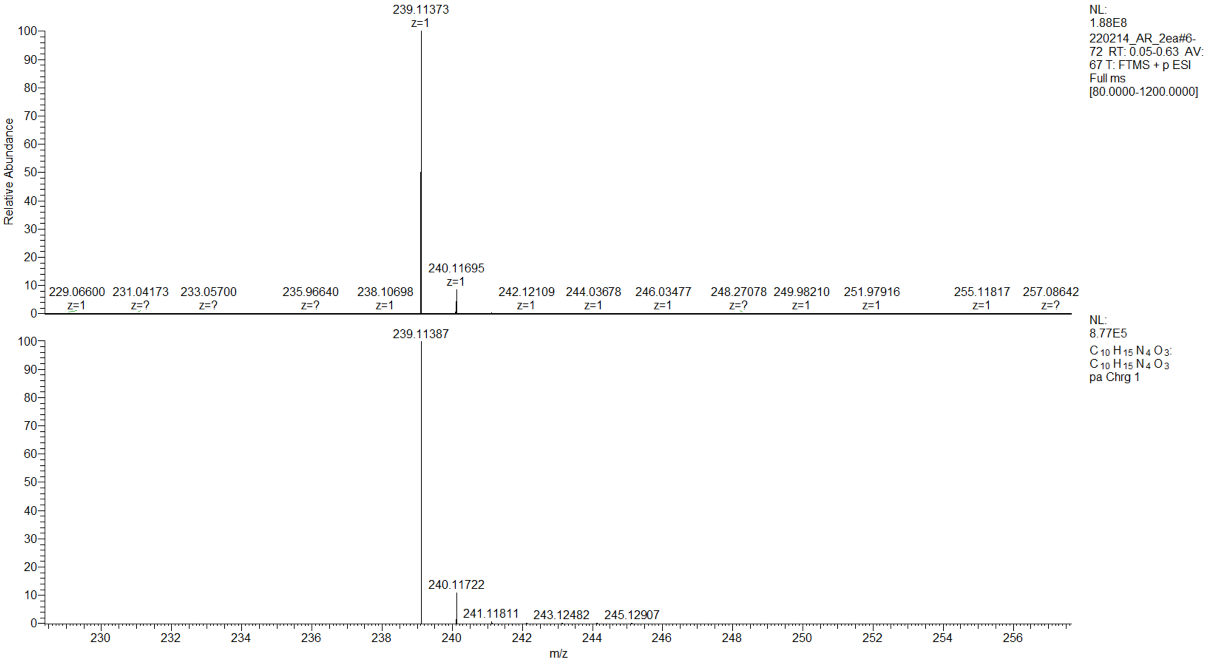

Supplementary Figure 92. ATR-FTIR spectrum of **2v** (neat)

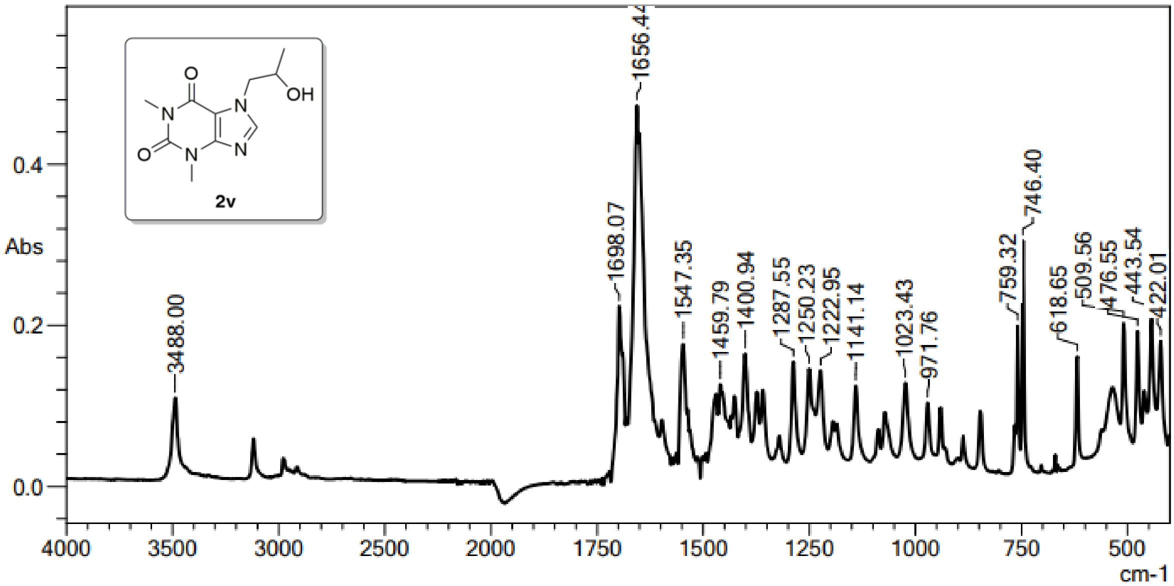

1-(6-Chloro-9H-purin-9-yl)propan-2-ol (2w)

Supplementary Figure 93. ^{1}H NMR spectrum of **2w** (500 MHz, $CDCl_3$)

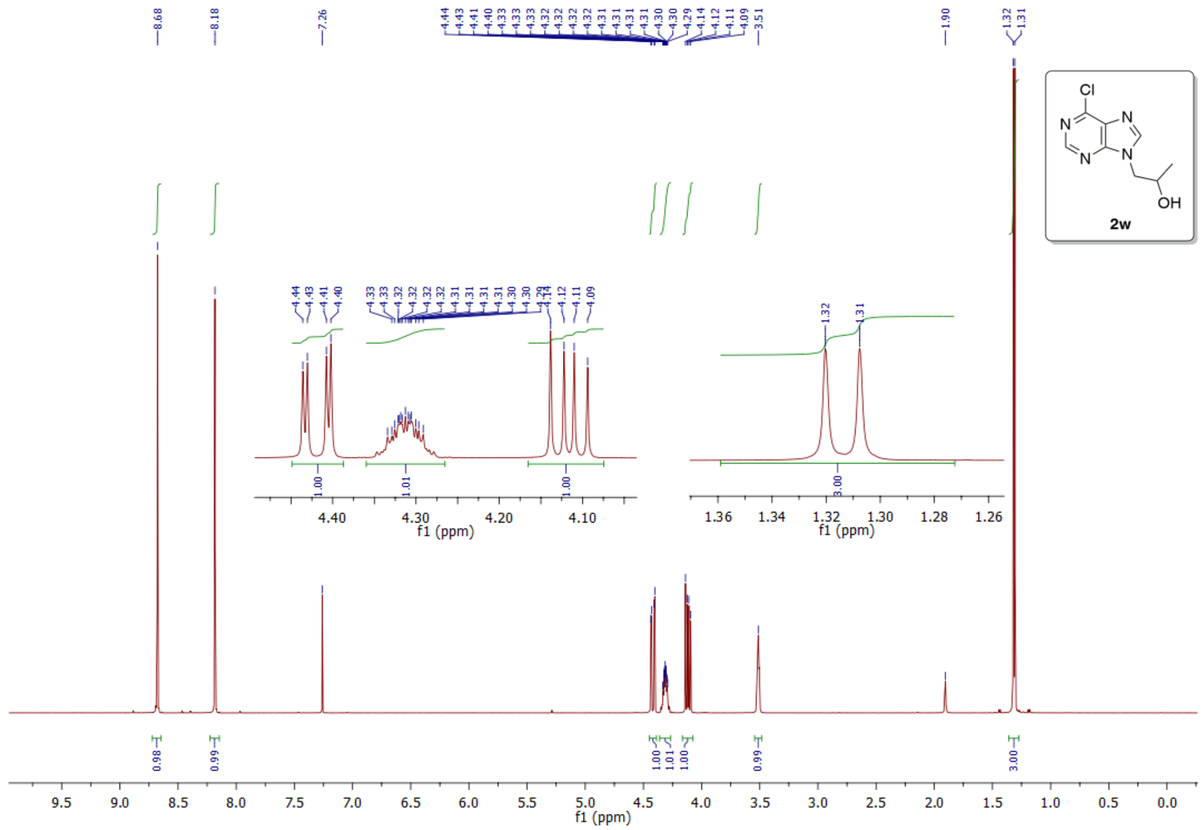

Supplementary Figure 94. $^{13}C\{^{1}H\}$ NMR spectrum of **2w** (126 MHz, $CDCl_3$)

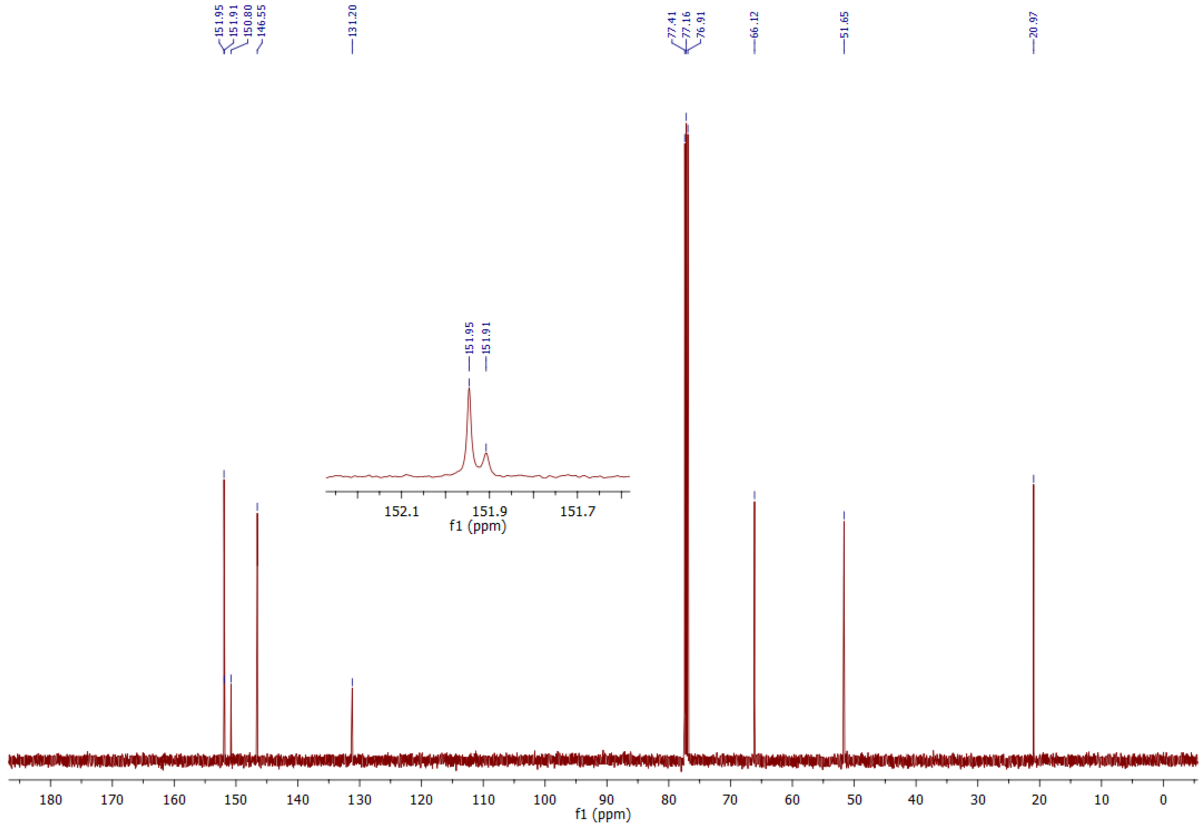

Supplementary Figure 95. FTMS spectrum of **2w** (ESI-TOF)

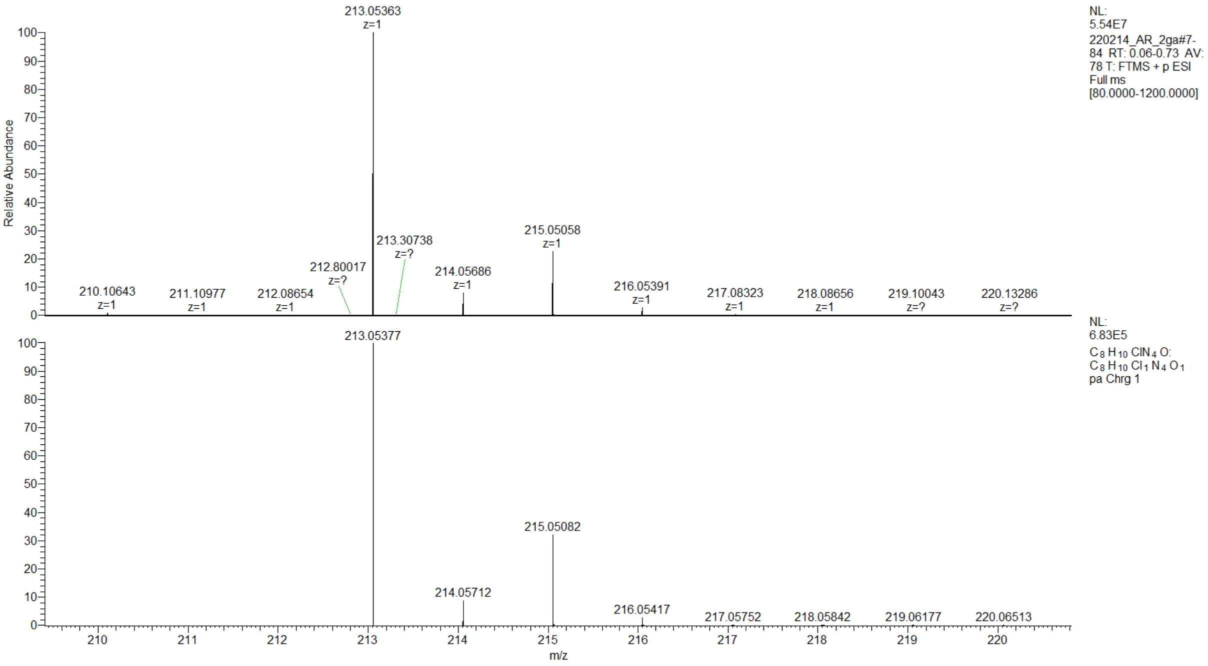

Supplementary Figure 96. ATR-FTIR spectrum of **2w** (neat)

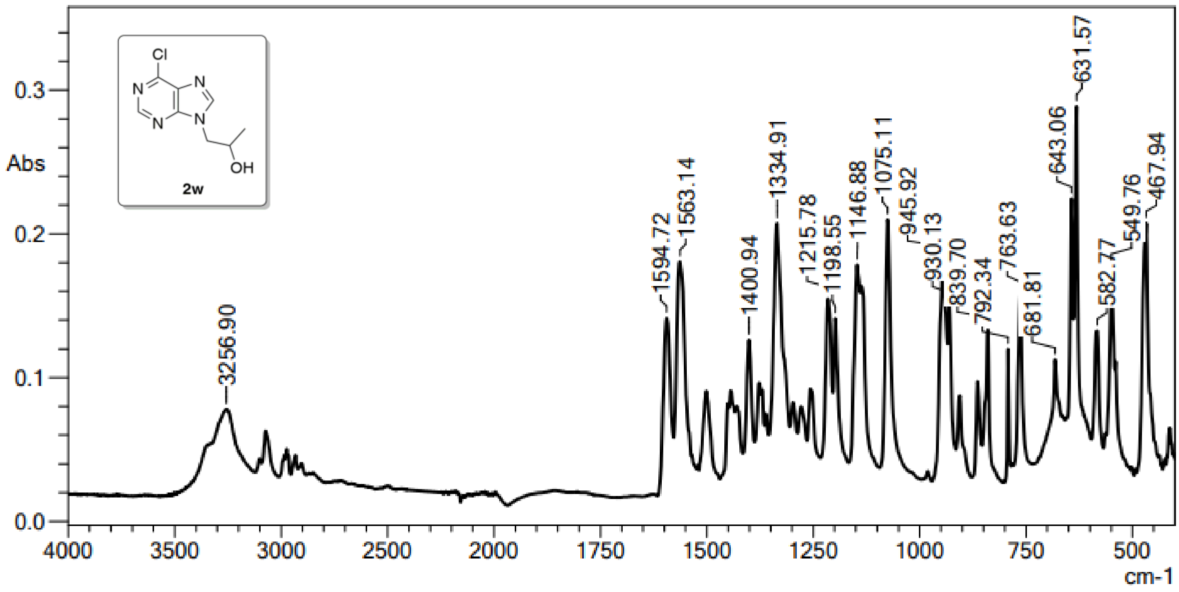

2-(3-Chloro-2-hydroxypropyl)-1H-isoindole-1,3(2H)-dione (2x)

Supplementary Figure 97. ^{1}H NMR spectrum of **2x** (500 MHz, $CDCl_3$)

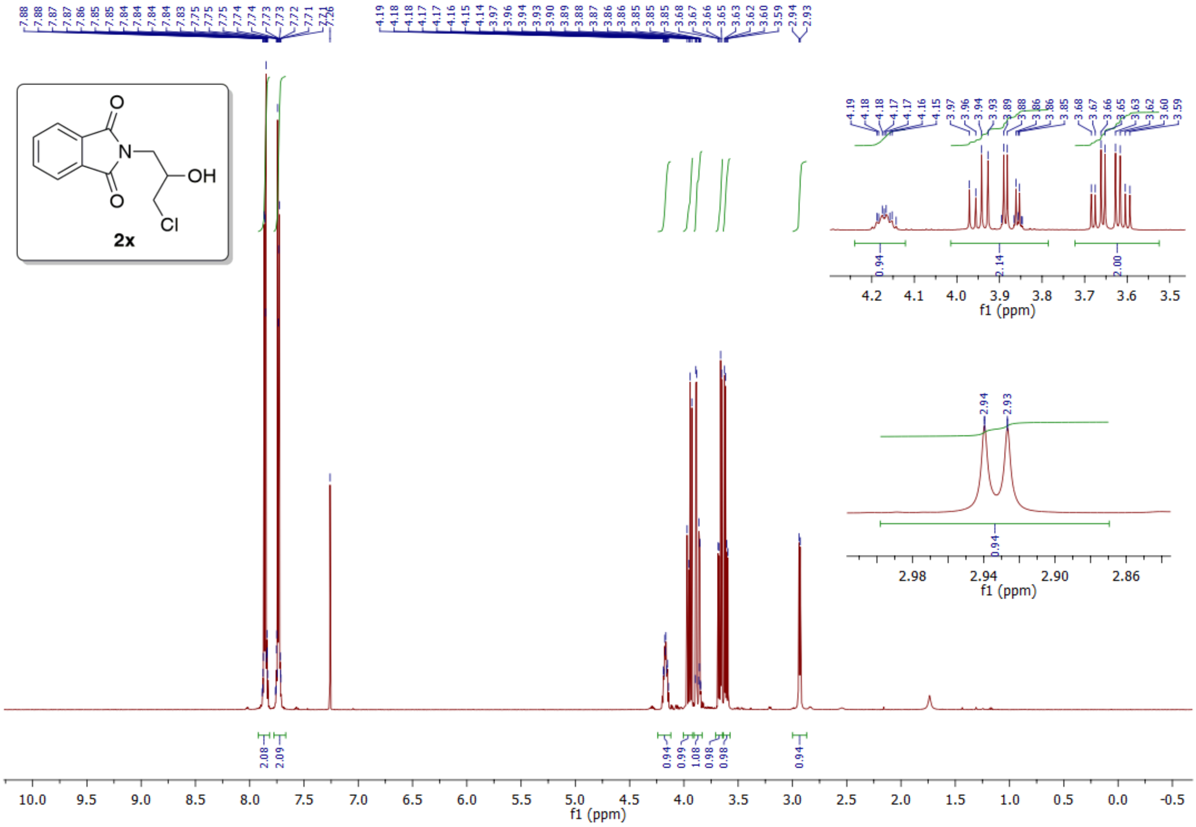

Supplementary Figure 98. $^{13}C\{^{1}H\}$ NMR spectrum of **2x** (126 MHz, $CDCl_3$)

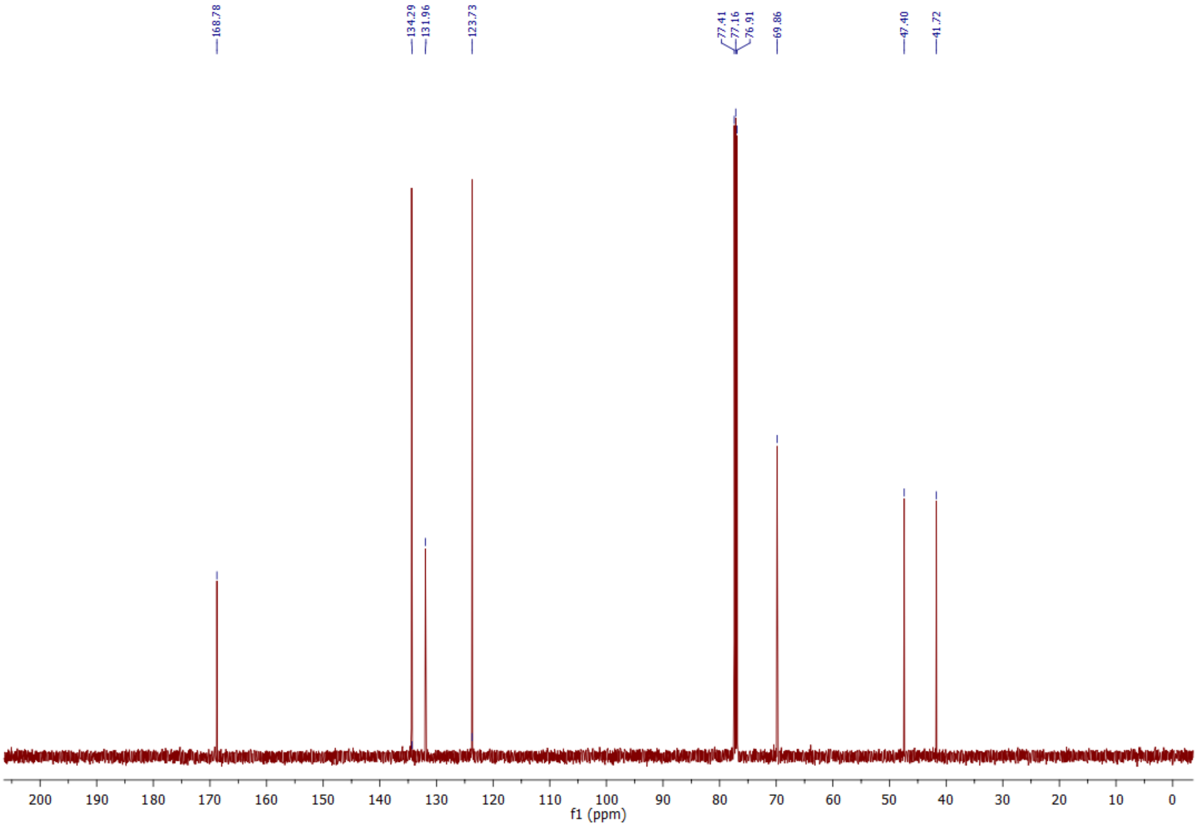

Supplementary Figure 99. FTMS spectrum of **2x** (ESI-TOF)

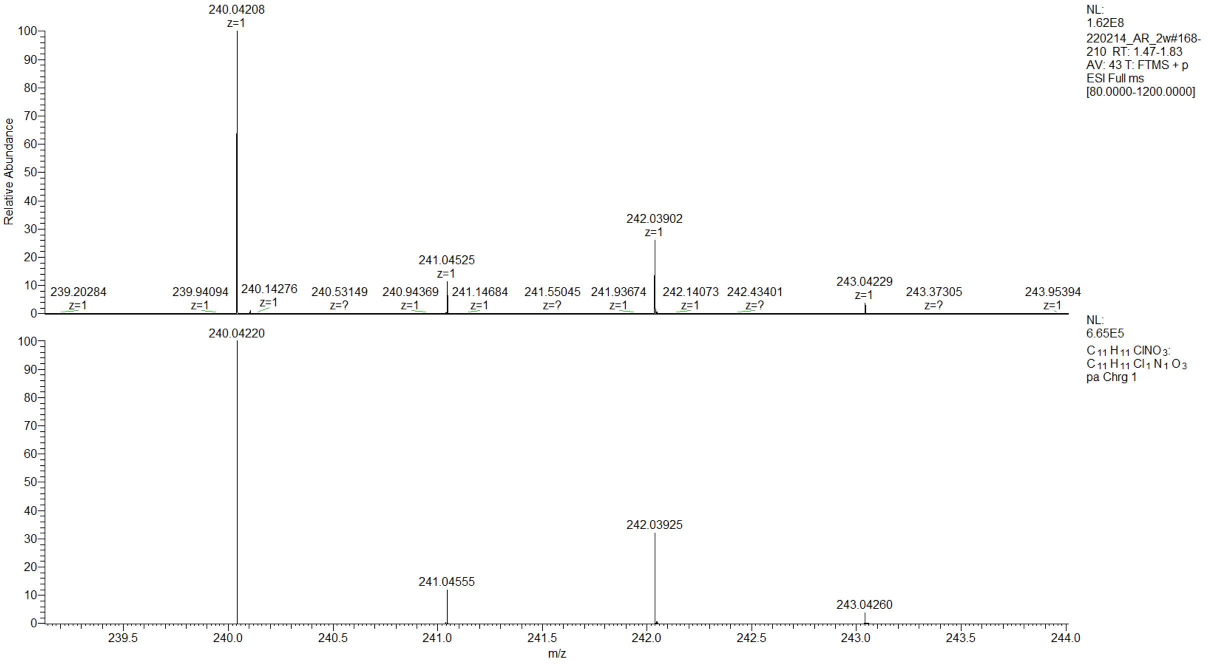

Supplementary Figure 100. ATR-FTIR spectrum of **2x** (neat)

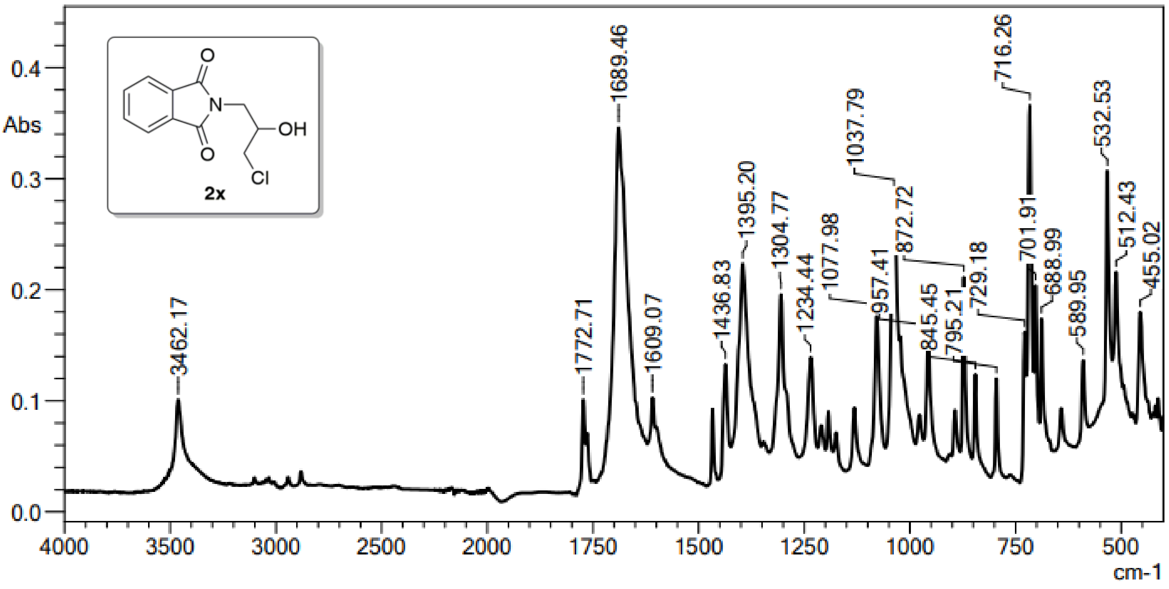

7-(3-Chloro-2-hydroxypropyl)-1,3-dimethyl-3,7-dihydro-1H-purine-2,6-dione (2y)

Supplementary Figure 101. ^{1}H NMR spectrum of **2y** (500 MHz, $CDCl_3$)

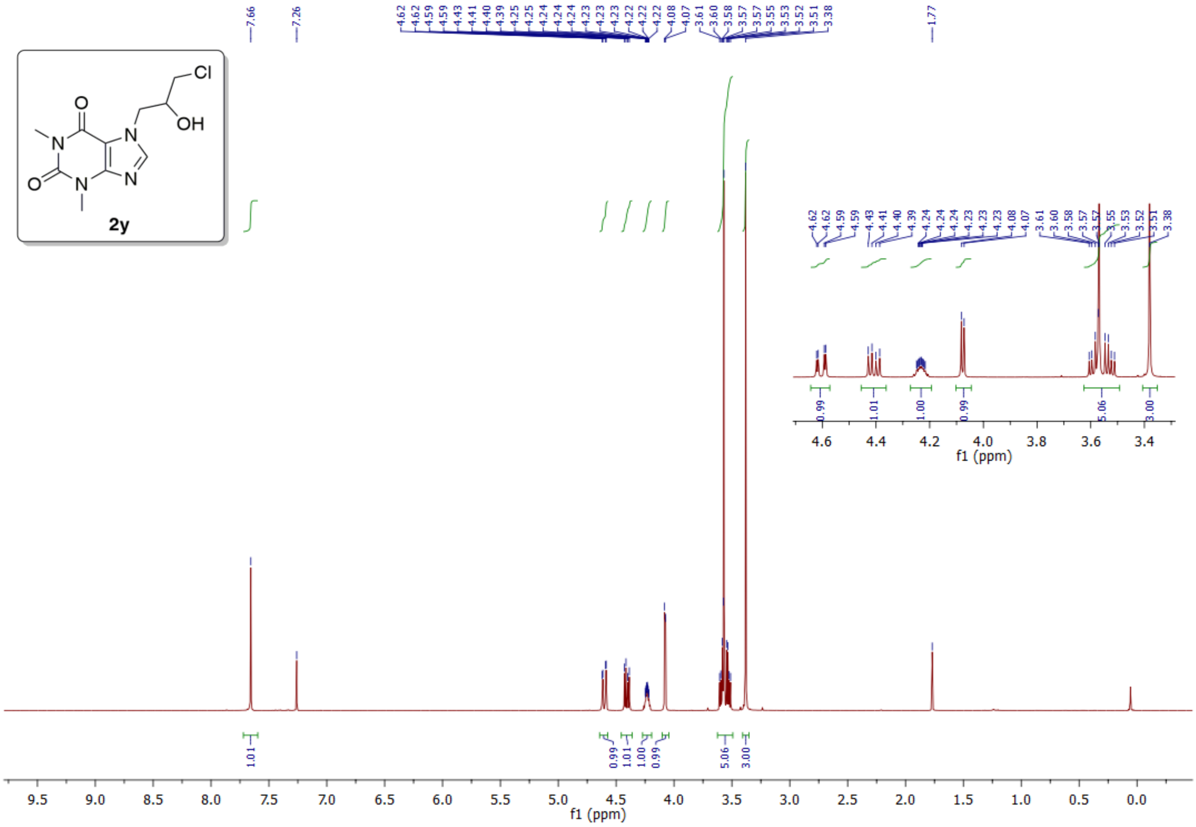

Supplementary Figure 102. $^{13}C\{^{1}H\}$ NMR spectrum of **2y** (126 MHz, $CDCl_3$)

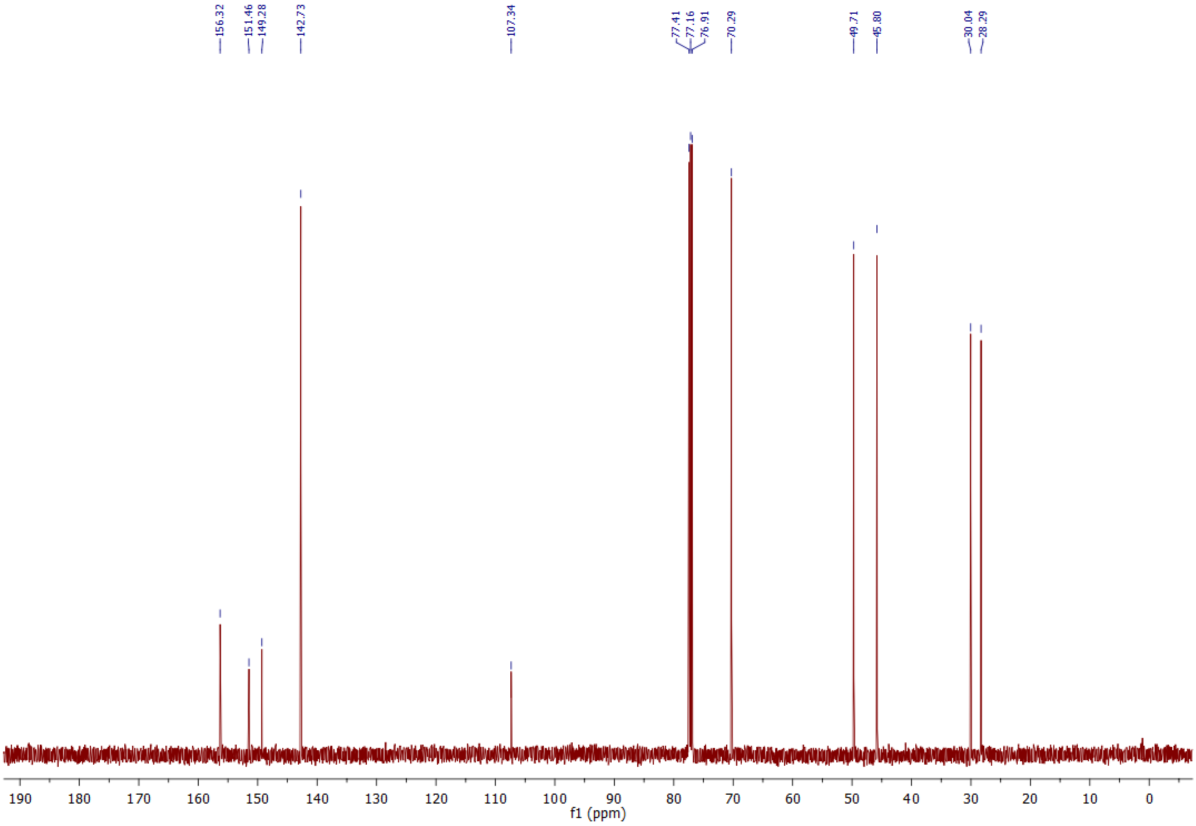

Supplementary Figure 103. FTMS spectrum of **2y** (ESI-TOF)

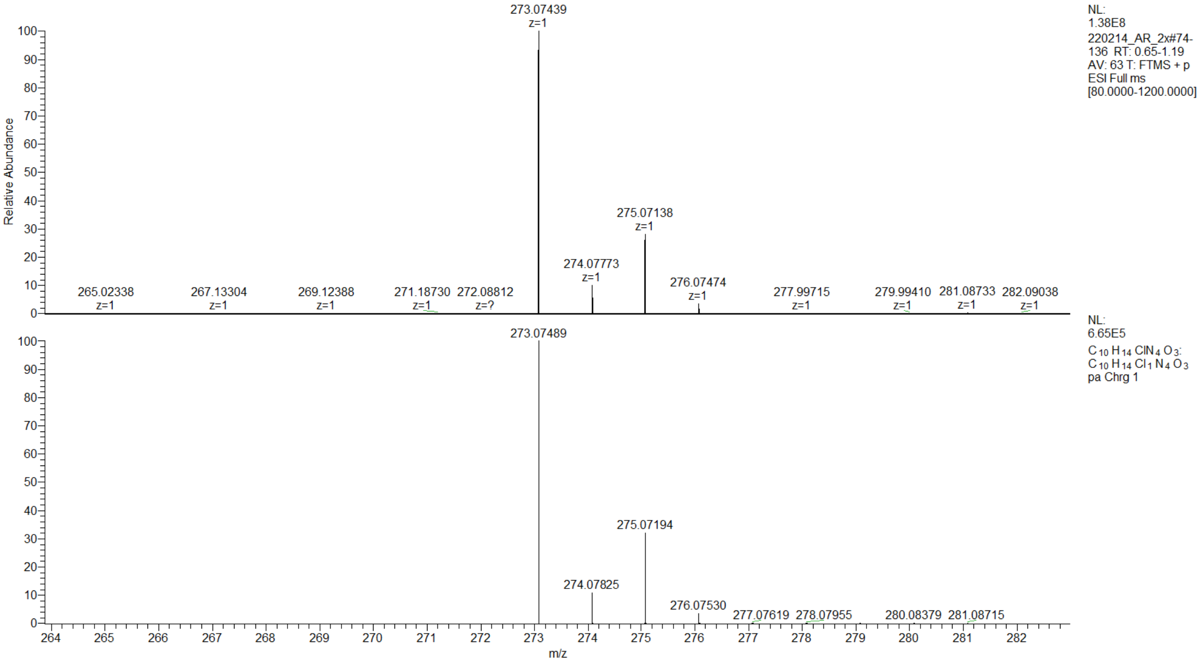

Supplementary Figure 104. ATR-FTIR spectrum of **2y** (neat)

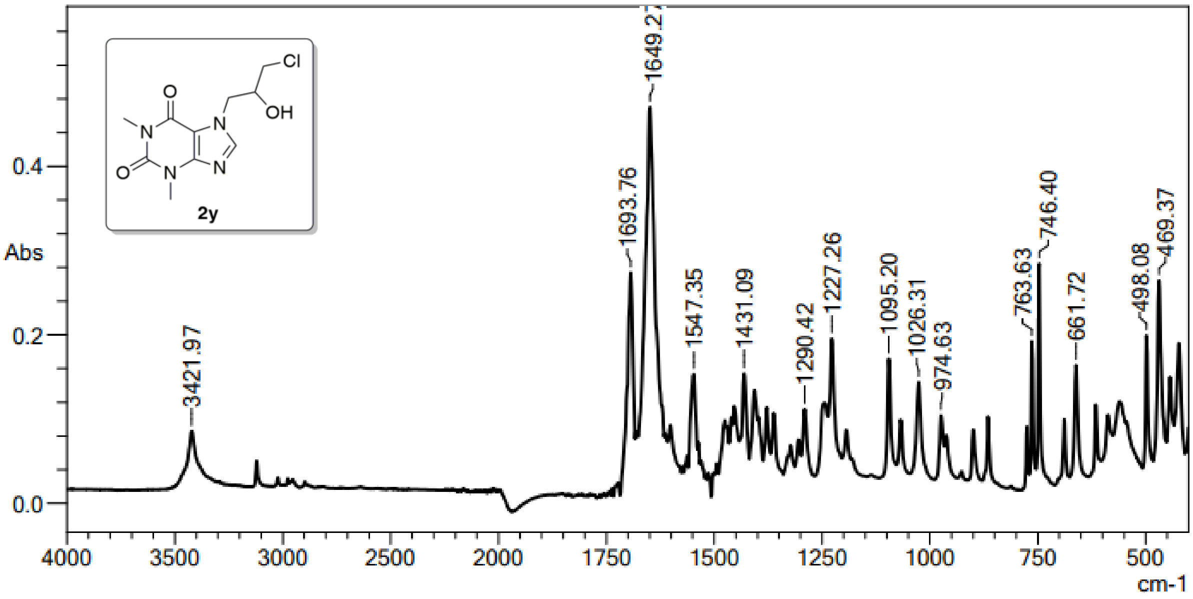

Methyl hydroxy(phenyl)acetate (2z)

Supplementary Figure 105. ^{1}H NMR spectrum of **2z** (500 MHz, $CDCl_3$)

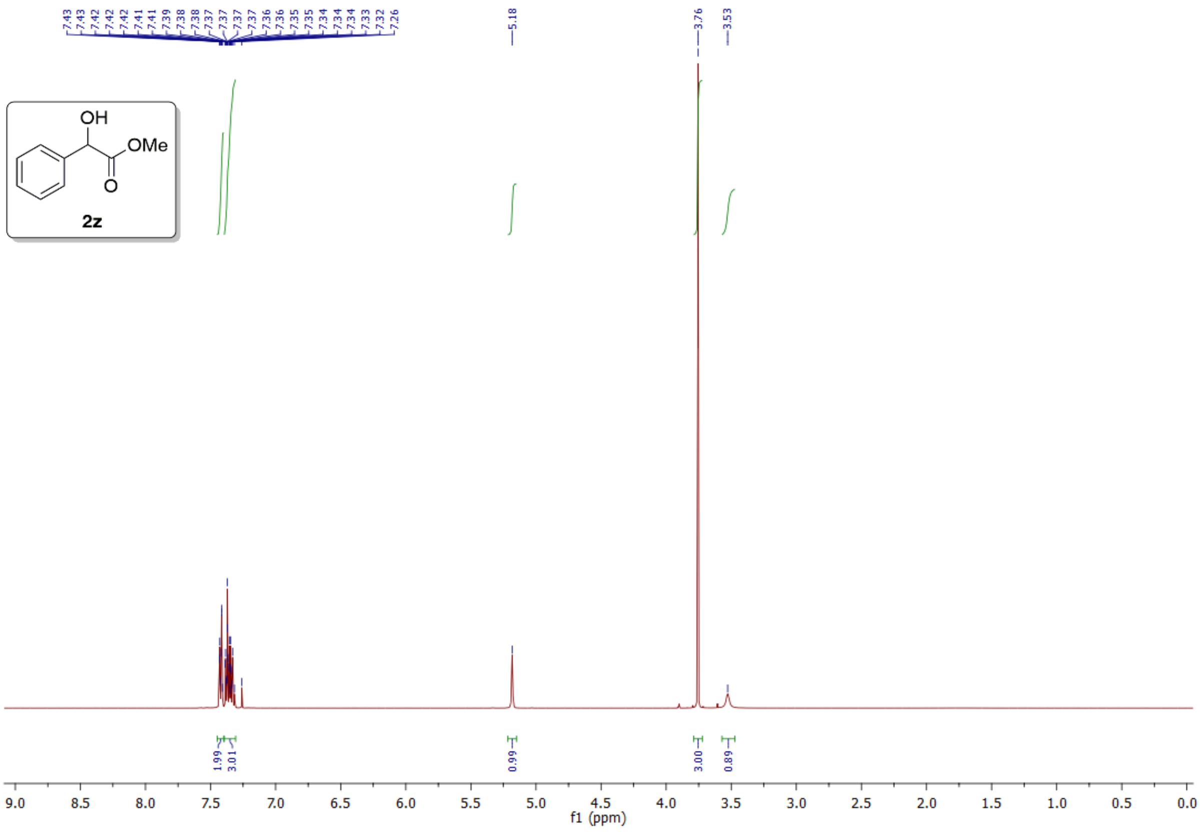

Supplementary Figure 106. $^{13}C\{^{1}H\}$ NMR spectrum of **2z** (126 MHz, $CDCl_3$)

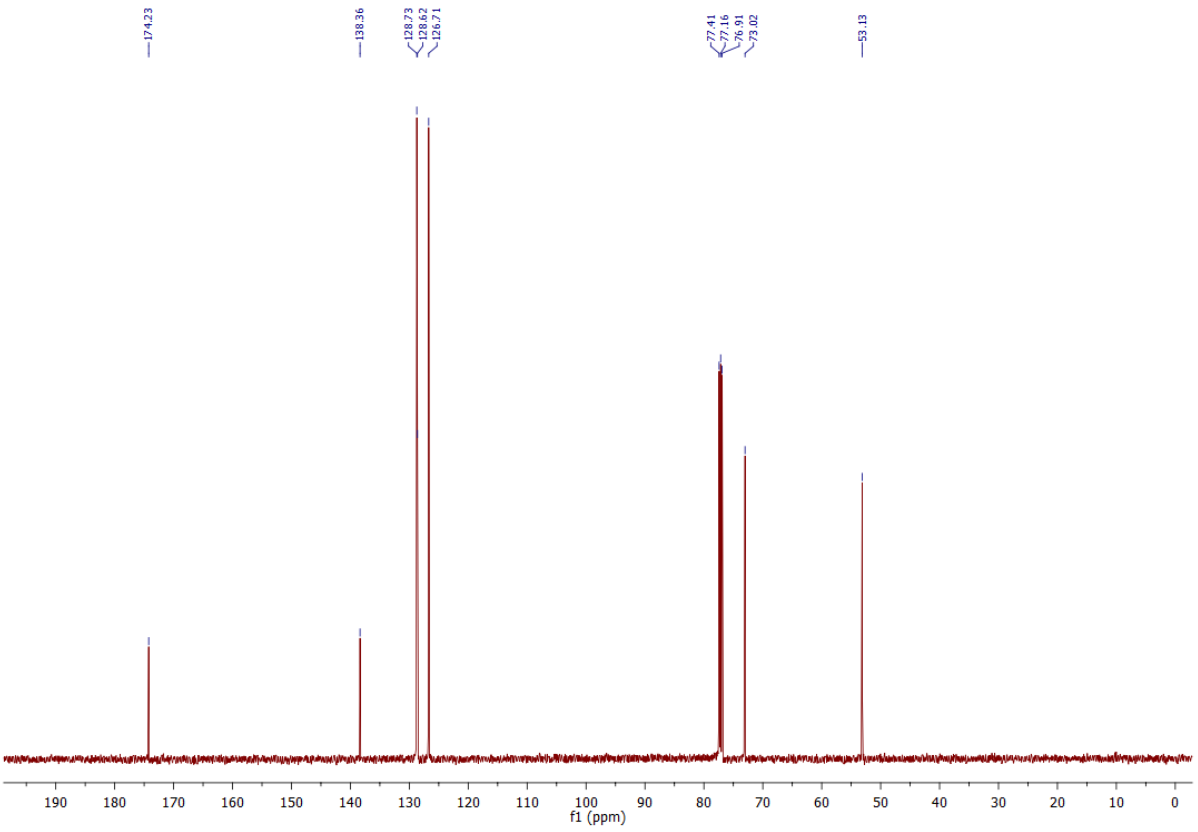

Supplementary Figure 107. FTMS spectrum of **2z** (ESI-TOF)

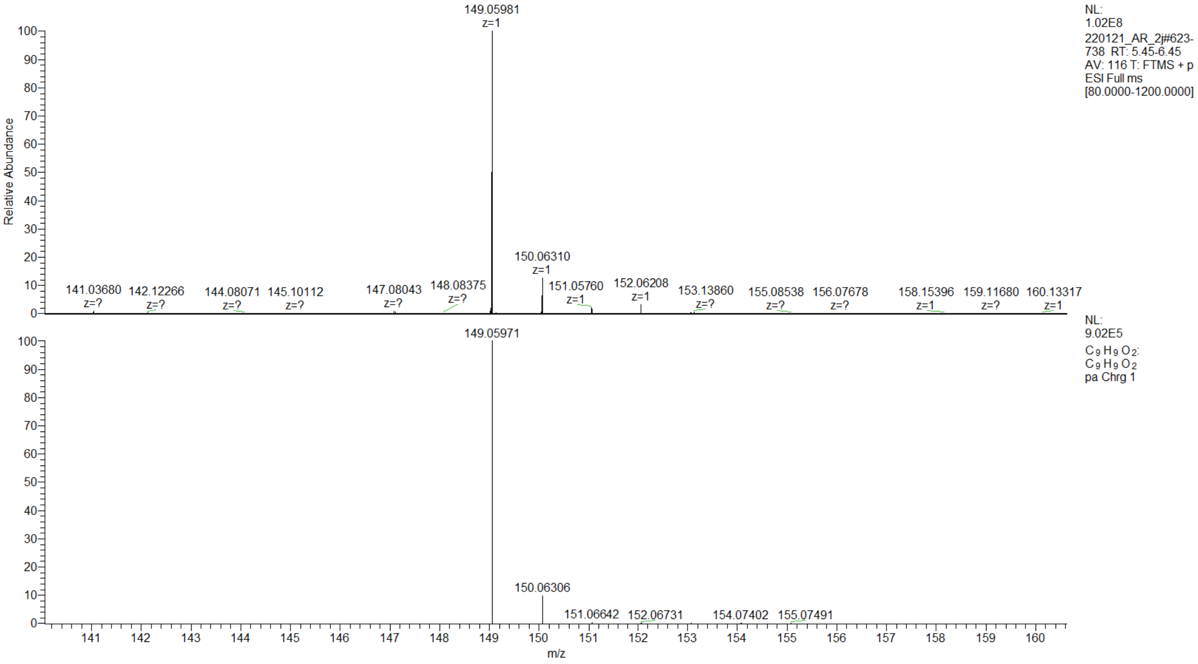

Supplementary Figure 108. ATR-FTIR spectrum of **2z** (neat)

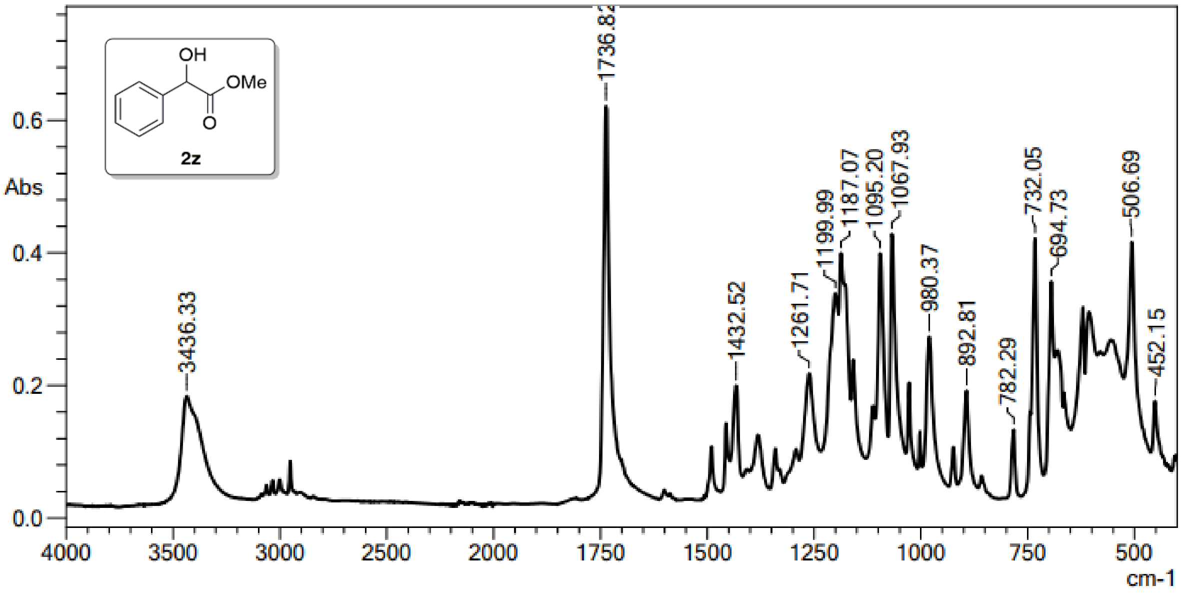

Methyl 2-hydroxy-3-phenylpropanoate (2aa)

Supplementary Figure 109. ^{1}H NMR spectrum of **2aa** (500 MHz, $CDCl_3$)

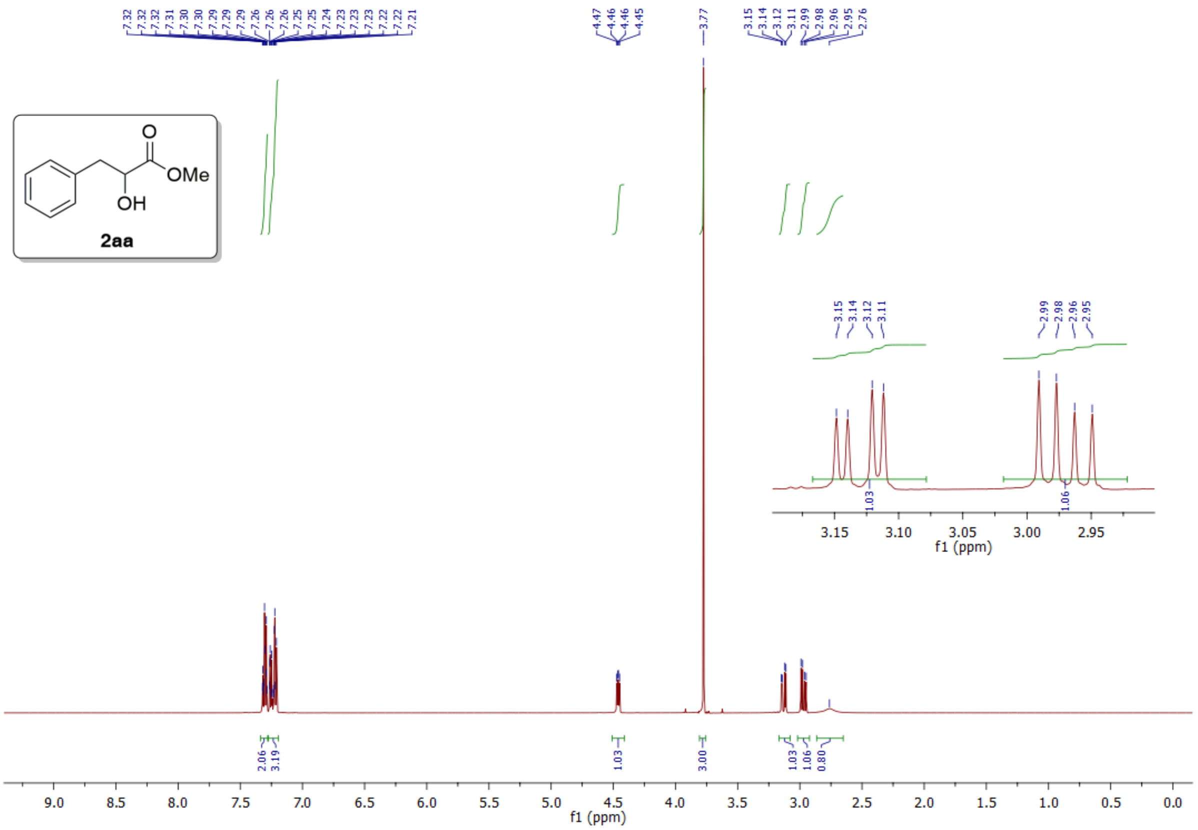

Supplementary Figure 110. $^{13}C\{^1H\}$ NMR spectrum of **2aa** (126 MHz, $CDCl_3$)

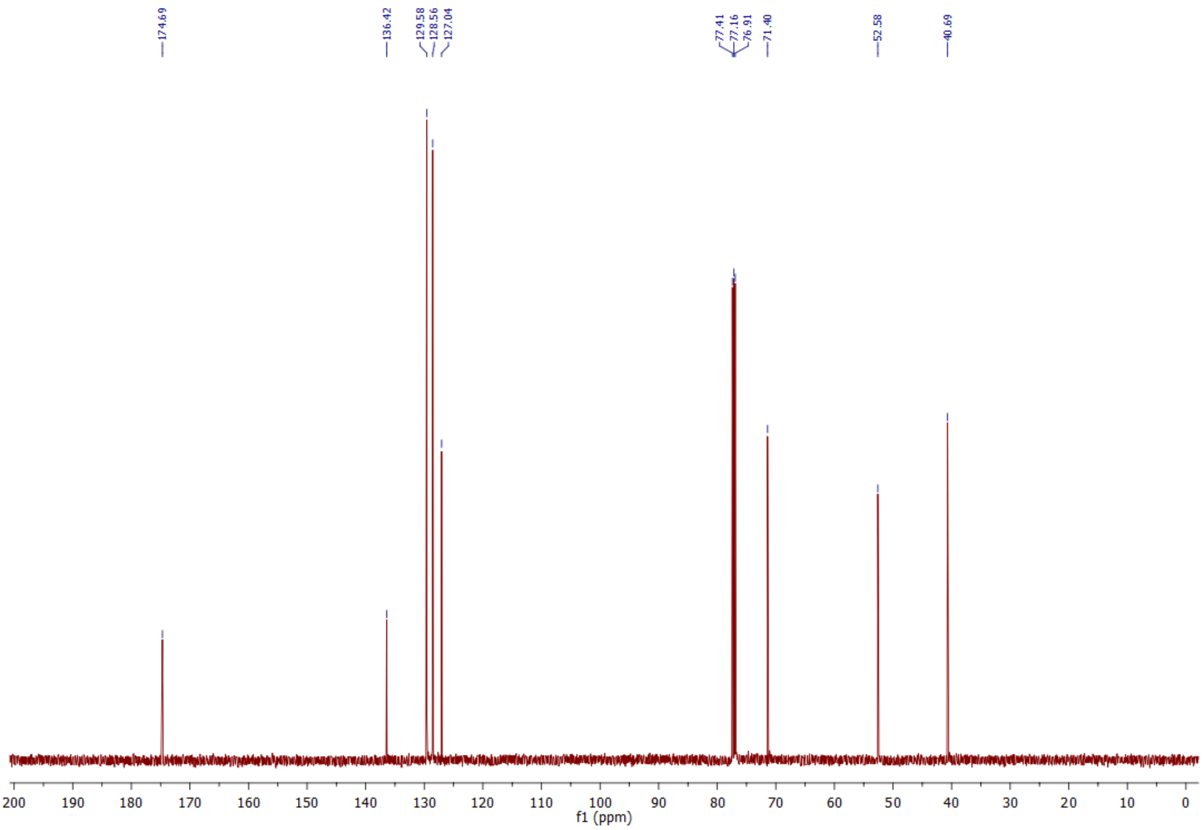

Supplementary Figure 111a. FTMS spectrum of **2aa** (ESI-TOF)

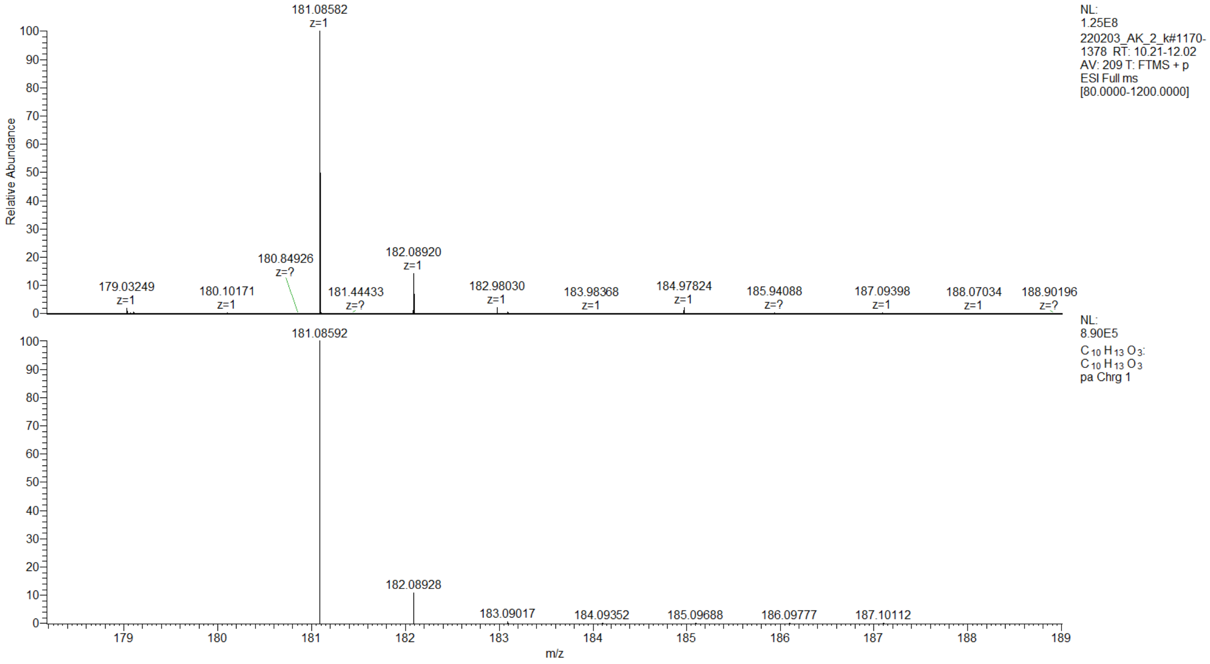

Supplementary Figure 111b. FTMS spectrum of **2aa** (ESI-TOF)

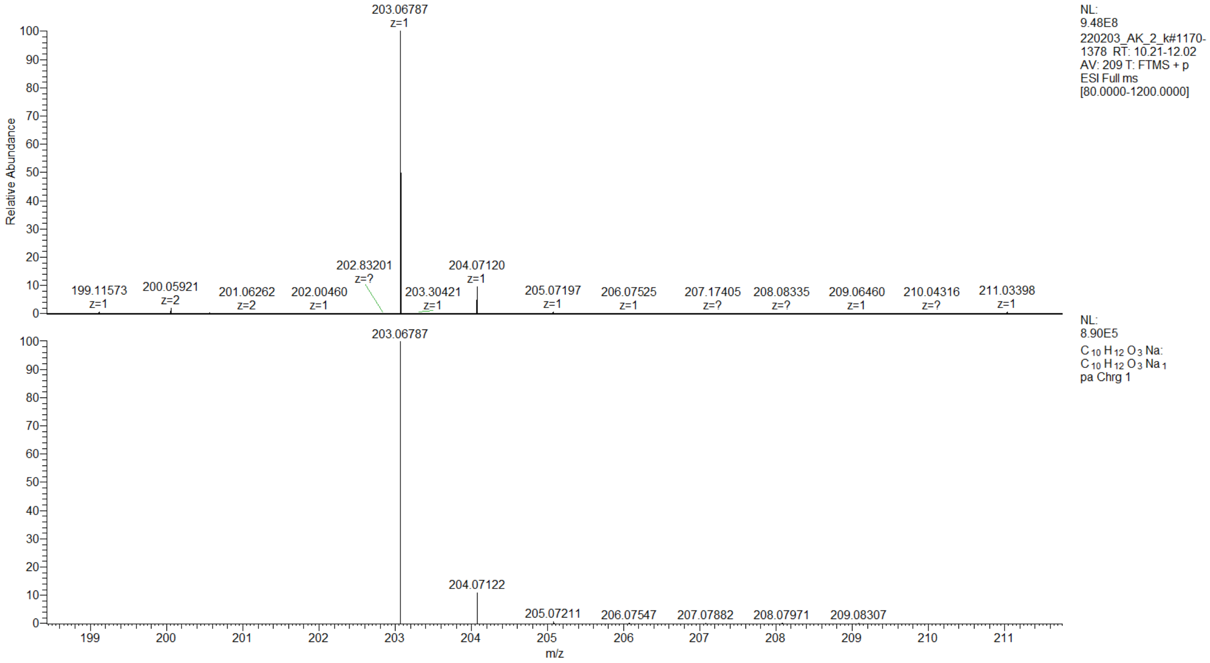

Supplementary Figure 112. ATR-FTIR spectrum of **2aa** (neat)

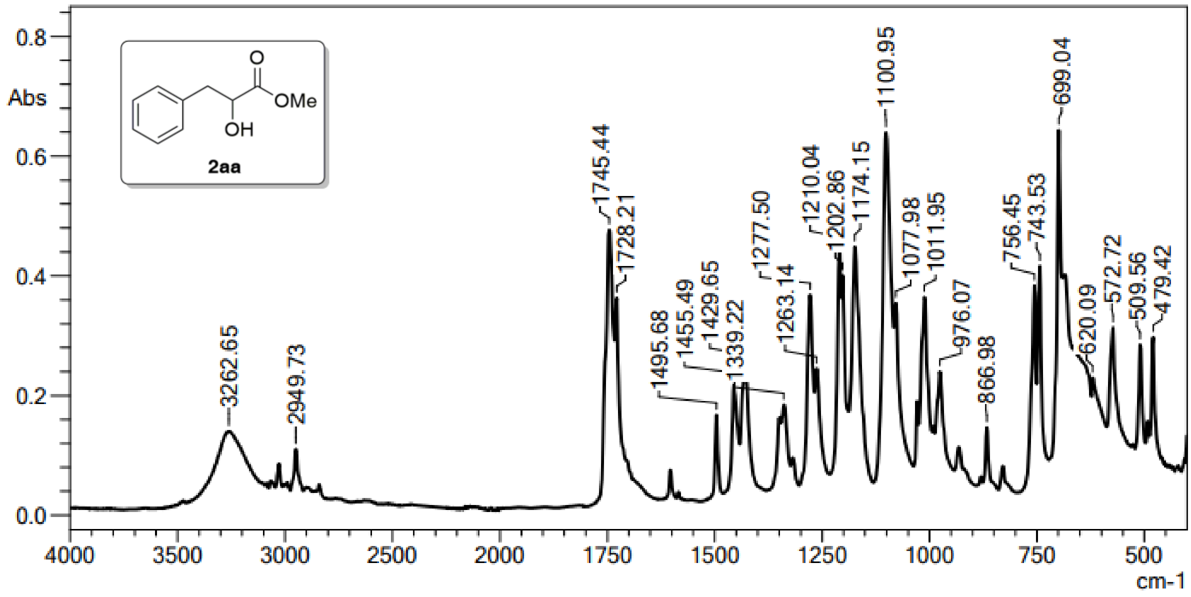

Ethyl 3-hydroxy-3-phenylpropanoate (2ab)

Supplementary Figure 113. ^{1}H NMR spectrum of **2ab** (500 MHz, $CDCl_3$)

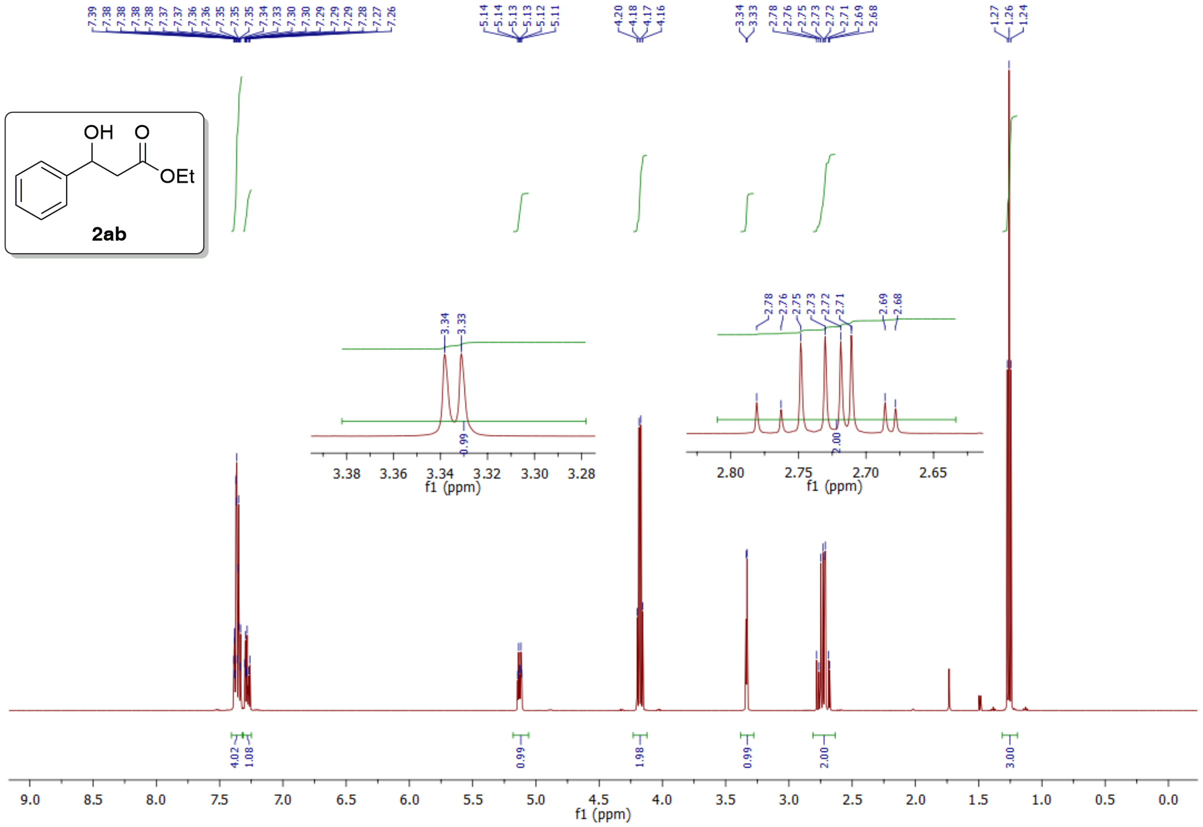

Supplementary Figure 114. ^{13}C{^{1}H} NMR spectrum of **2ab** (126 MHz, $CDCl_3$)

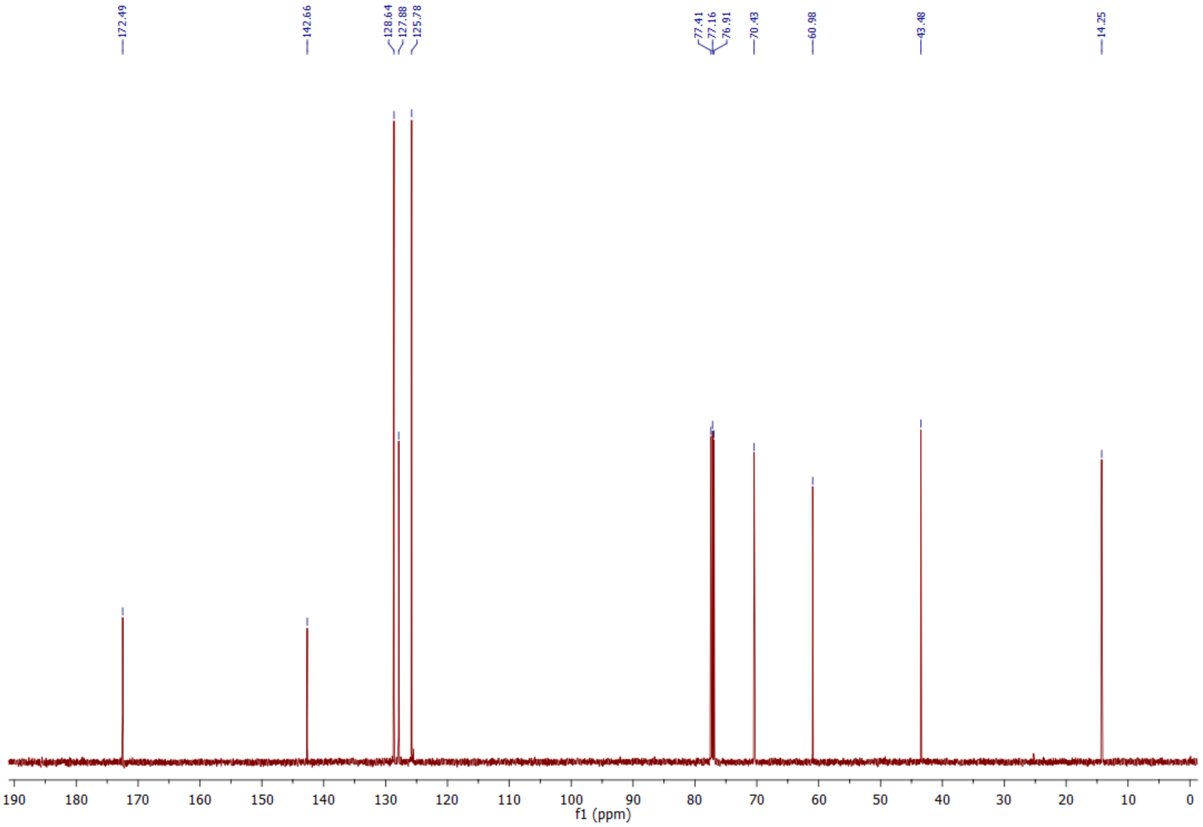

Supplementary Figure 115. FTMS spectrum of **2ab** (ESI-TOF)

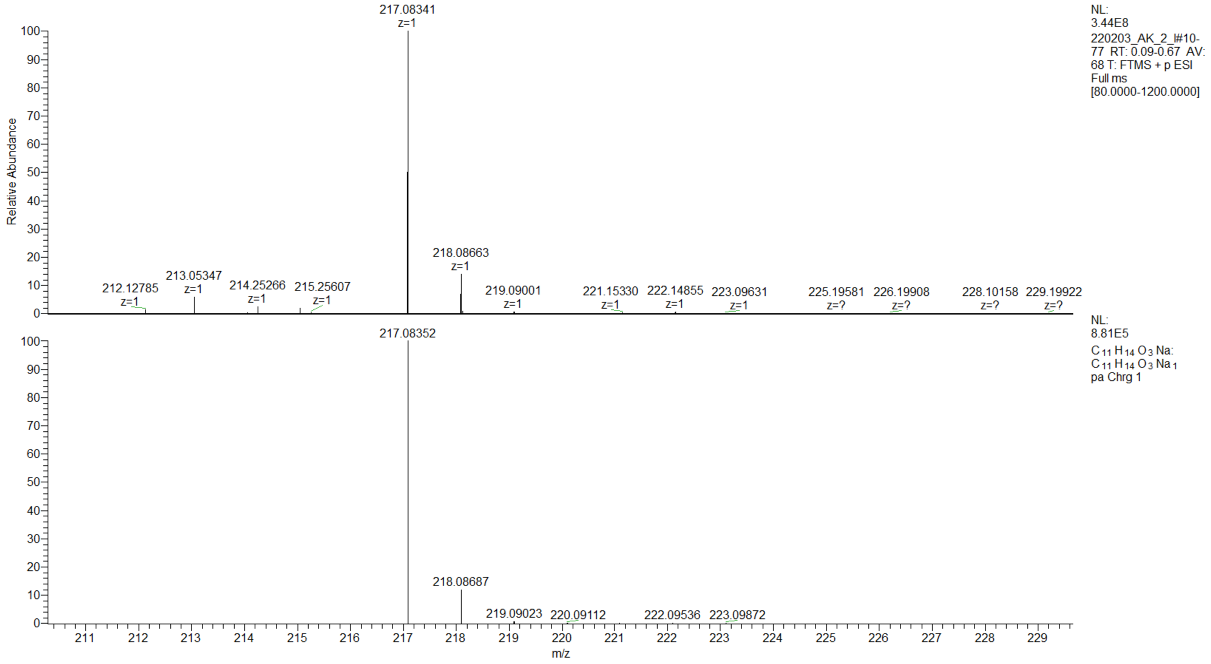

Supplementary Figure 116. ATR-FTIR spectrum of **2ab** (neat)

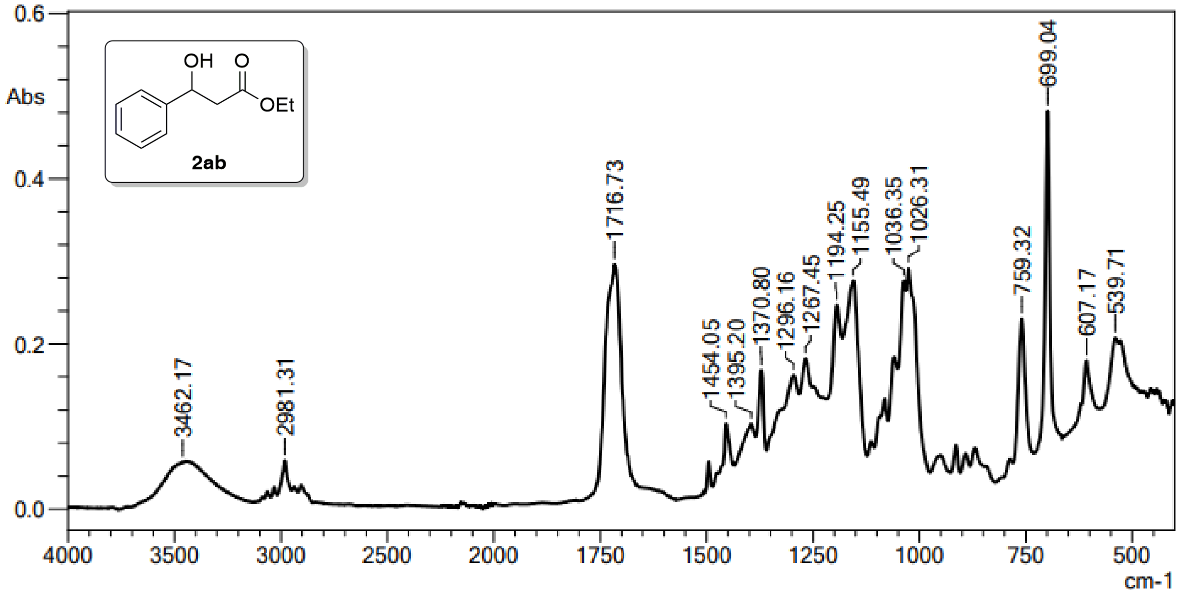

2-Methyl-1-phenylpropan-1-ol (2ac)

Supplementary Figure 117. ^{1}H NMR spectrum of **2ac** (500 MHz, $CDCl_3$)

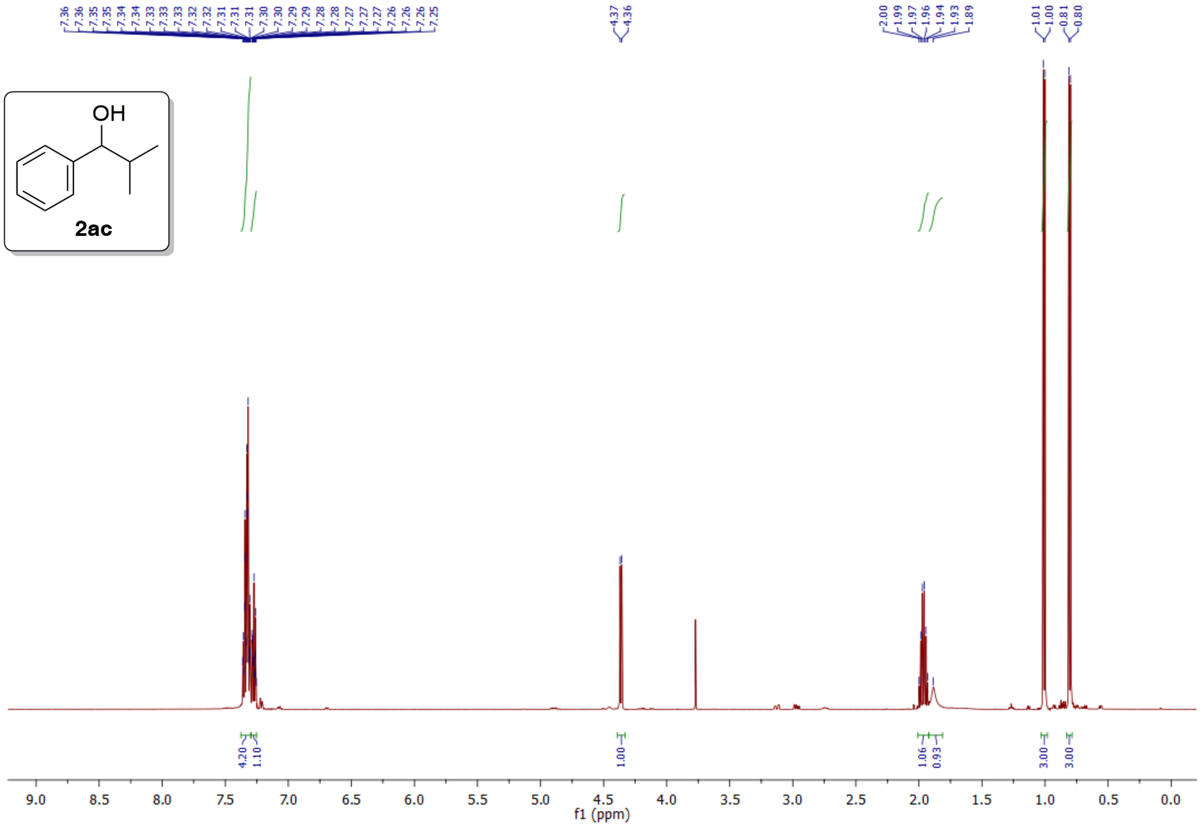

Supplementary Figure 118. $^{13}C\{^{1}H\}$ NMR spectrum of **2ac** (126 MHz, $CDCl_3$)

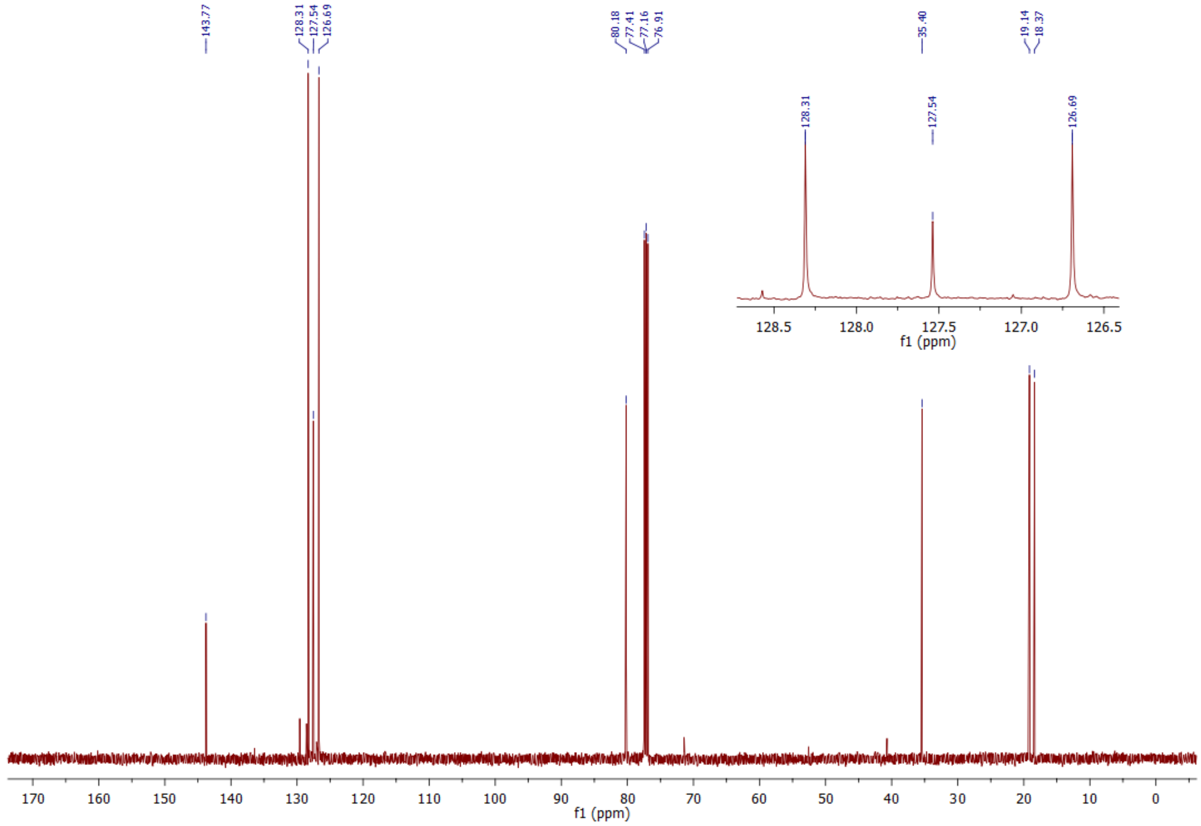

Supplementary Figure 119. FTMS spectrum of **2ac** (ESI-TOF)

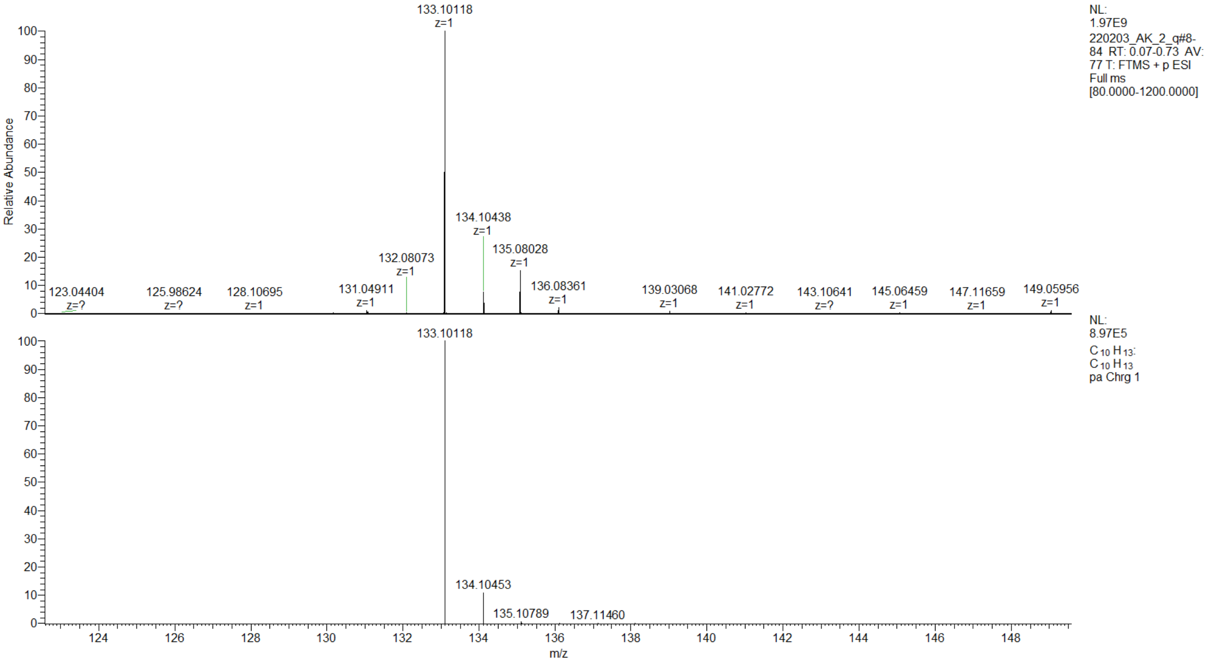

Supplementary Figure 120. ATR-FTIR spectrum of **2ac** (neat)

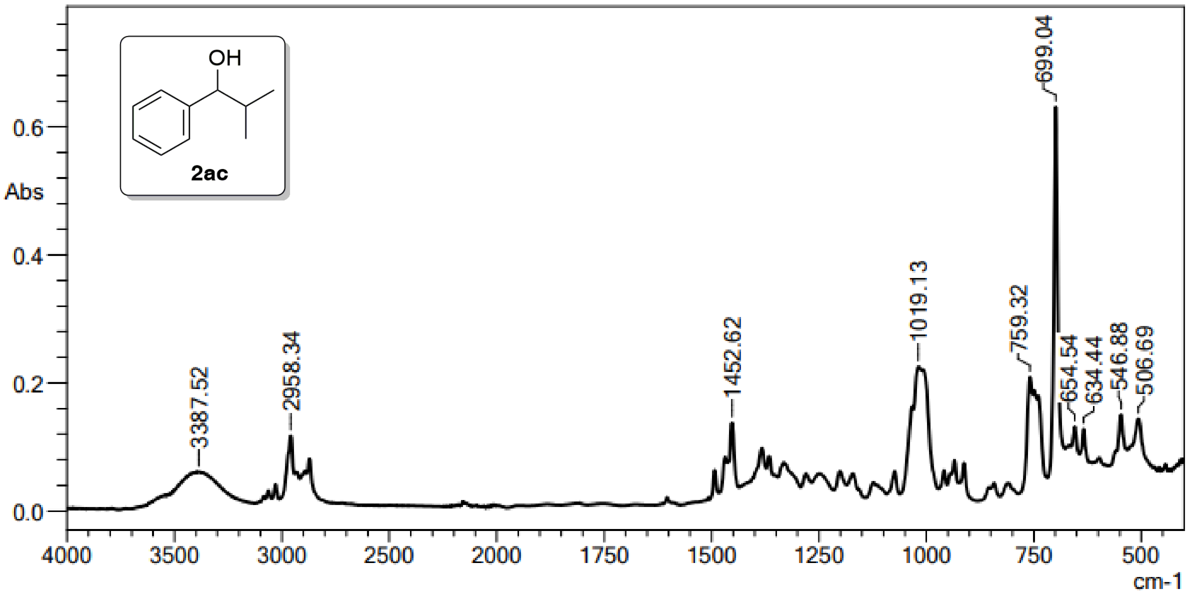

Cyclopropyl(phenyl)methanol (2ad)

Supplementary Figure 121. ^{1}H NMR spectrum of **2ad** (500 MHz, $CDCl_3$)

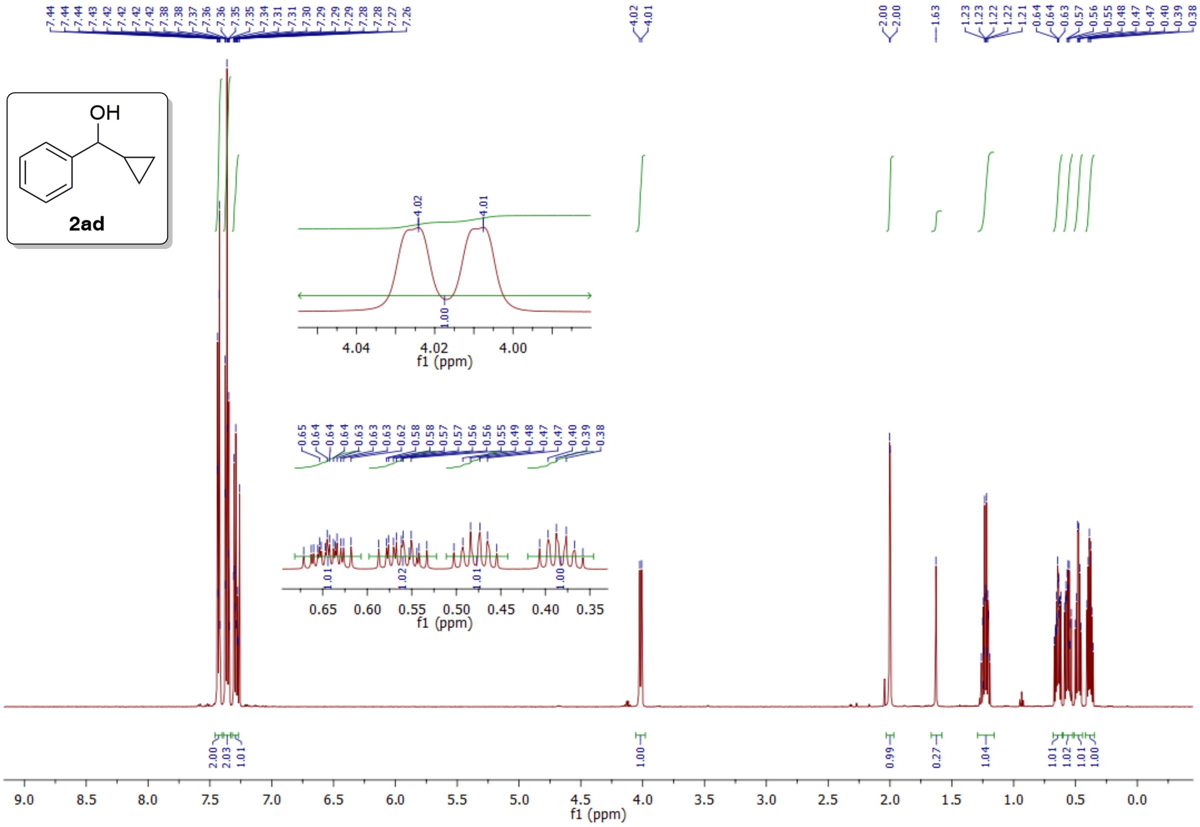

Supplementary Figure 122. $^{13}C\{^{1}H\}$ NMR spectrum of **2ad** (126 MHz, $CDCl_3$)

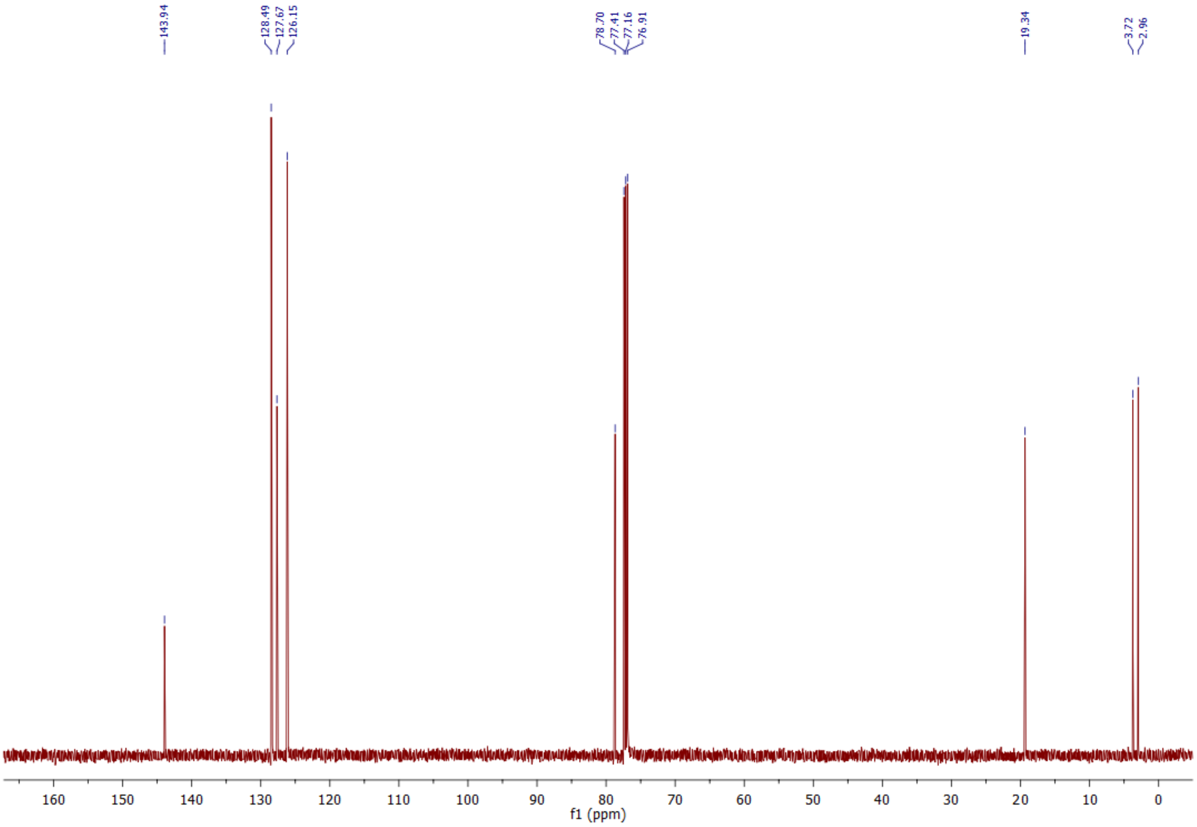

Supplementary Figure 123. FTMS spectrum of **2ad** (ESI-TOF)

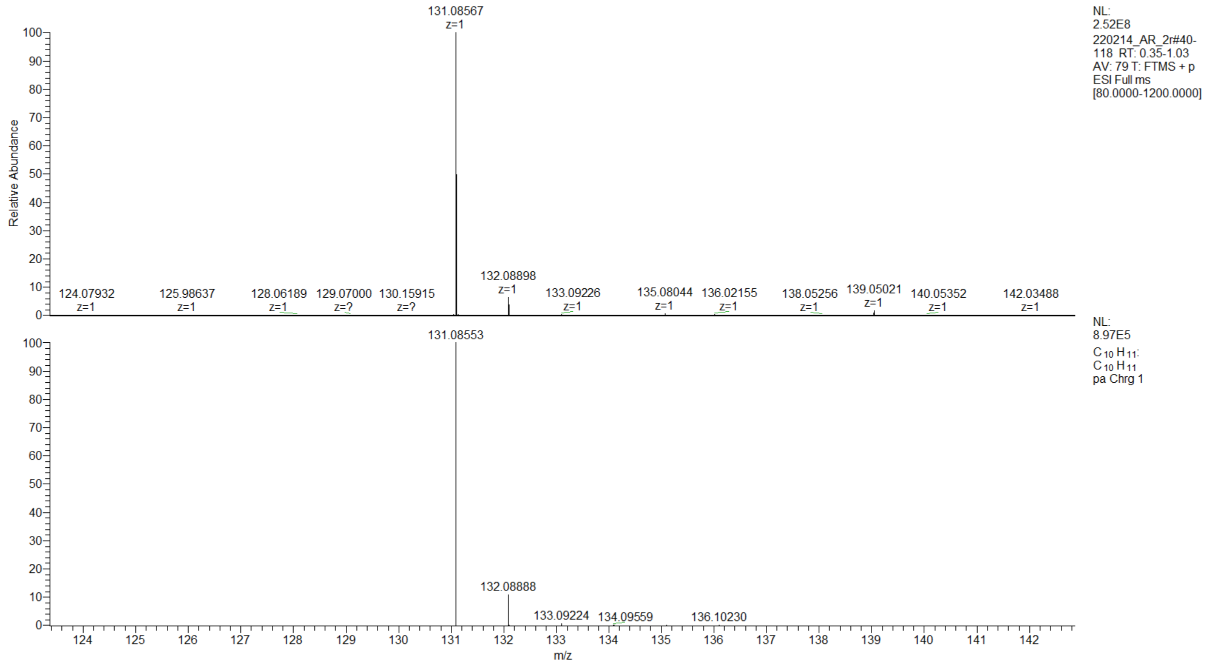

Supplementary Figure 124. ATR-FTIR spectrum of **2ad** (neat)

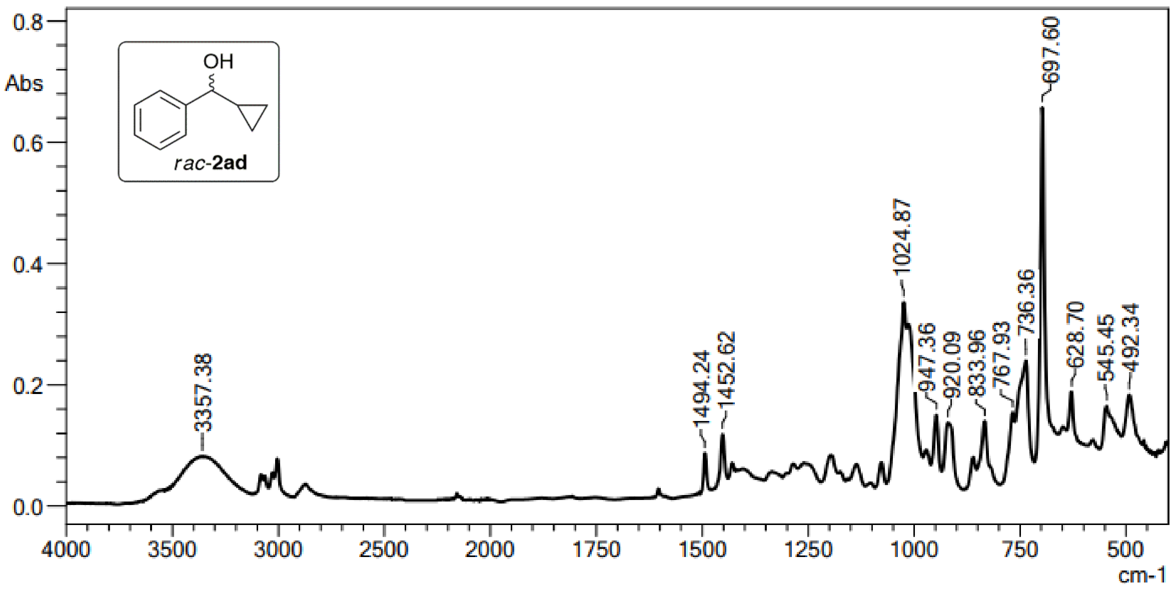

Cyclohexyl(phenyl)methanol (2ae)

Supplementary Figure 125. ^{1}H NMR spectrum of **2ae** (500 MHz, $CDCl_3$)

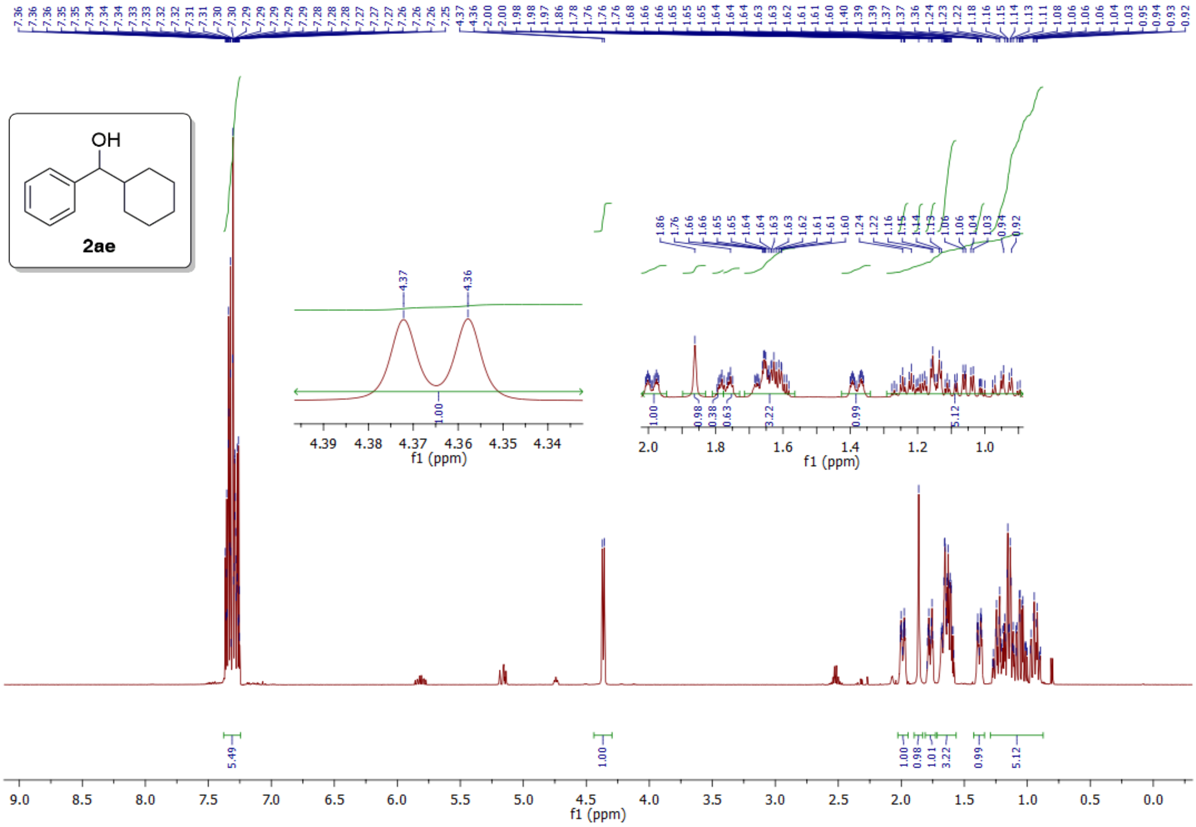

Supplementary Figure 126. $^{13}C\{^{1}H\}$ NMR spectrum of **2ae** (126 MHz, $CDCl_3$)

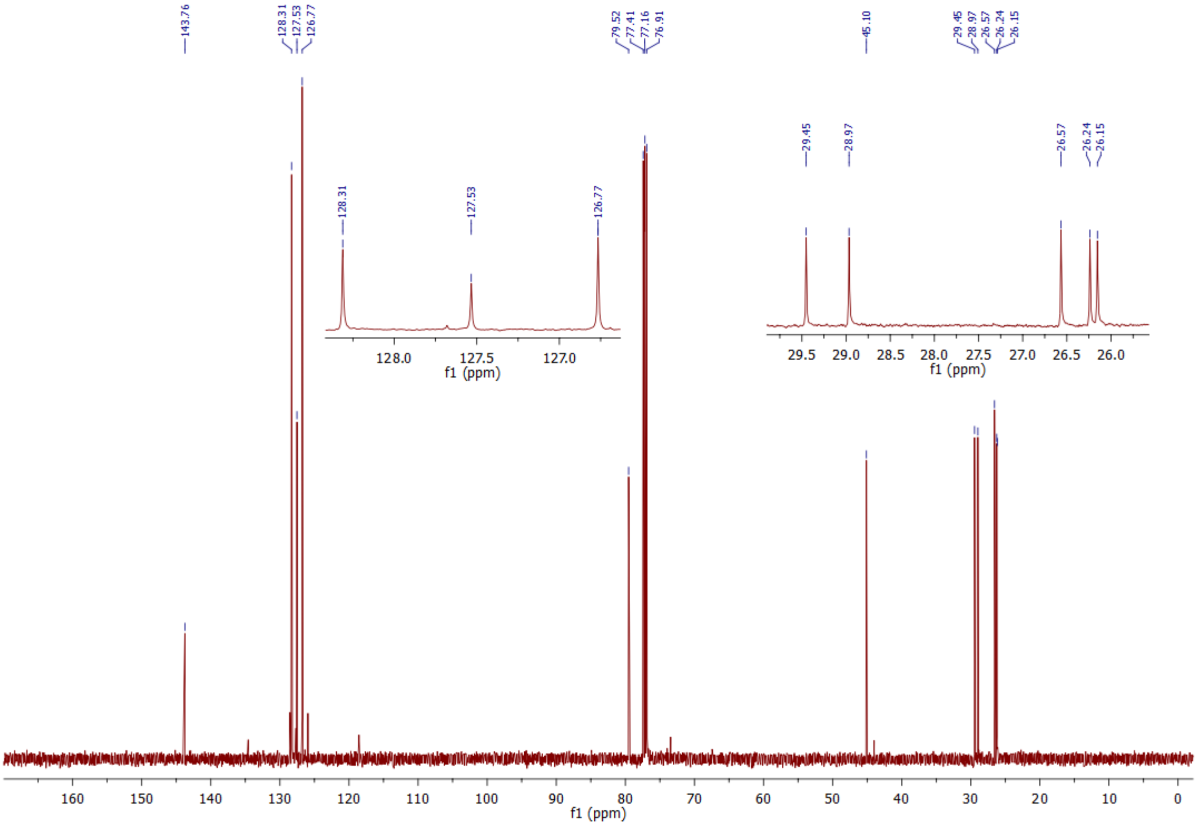

Supplementary Figure 127. FTMS spectrum of **2ae** (ESI-TOF)

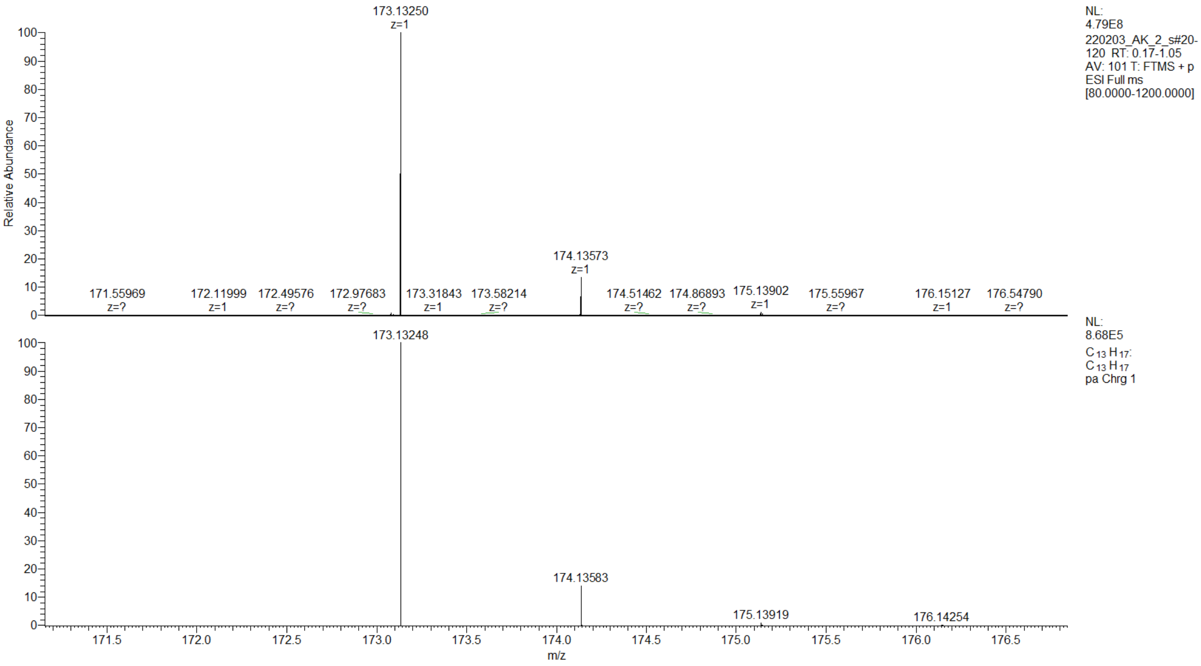

Supplementary Figure 128. ATR-FTIR spectrum of **2ae** (neat)

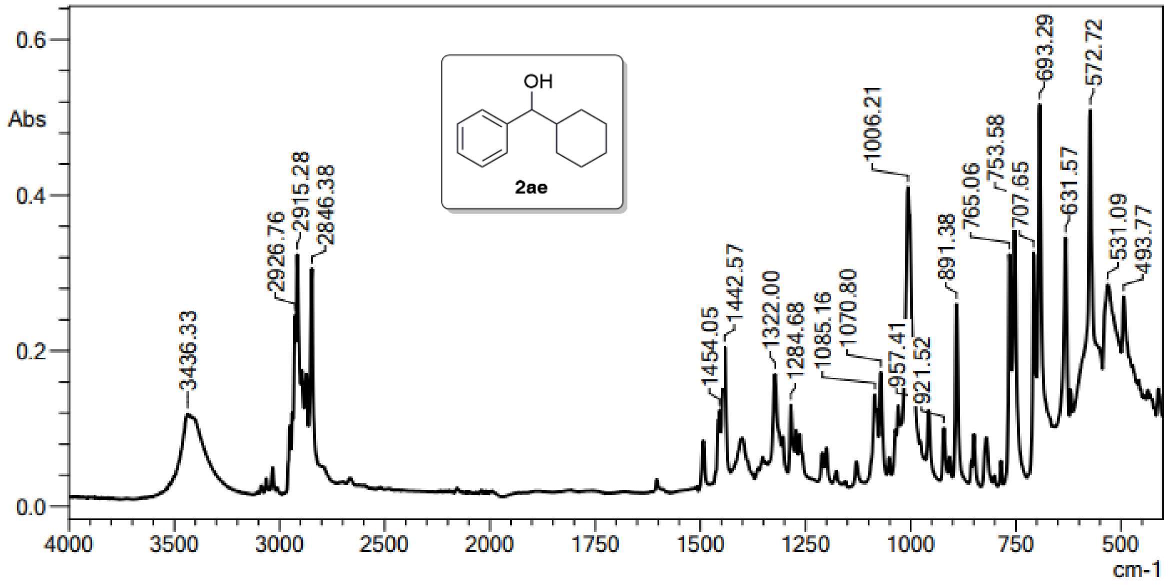

1,2-Diphenylethanol (2af)

Supplementary Figure 129. ^{1}H NMR spectrum of **2af** (500 MHz, $CDCl_3$)

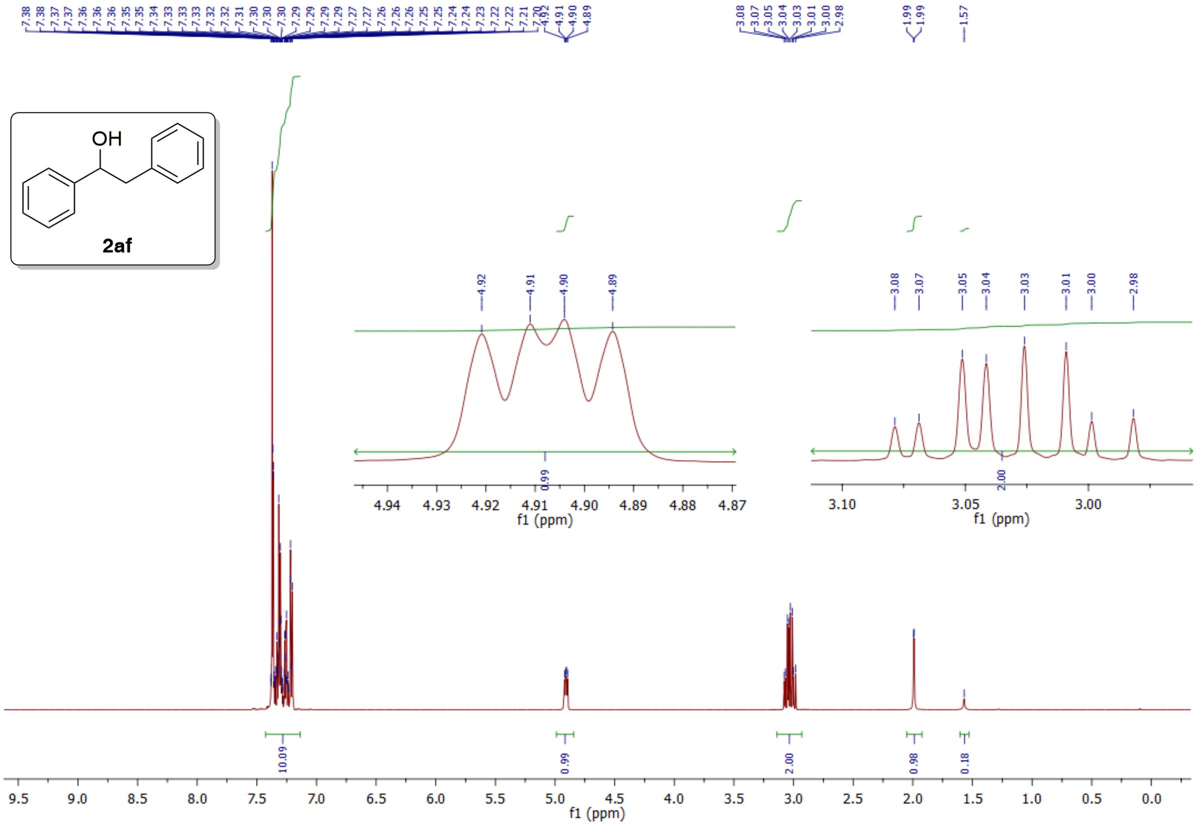

Supplementary Figure 130. $^{13}C\{^1H\}$ NMR spectrum of **2af** (126 MHz, $CDCl_3$)

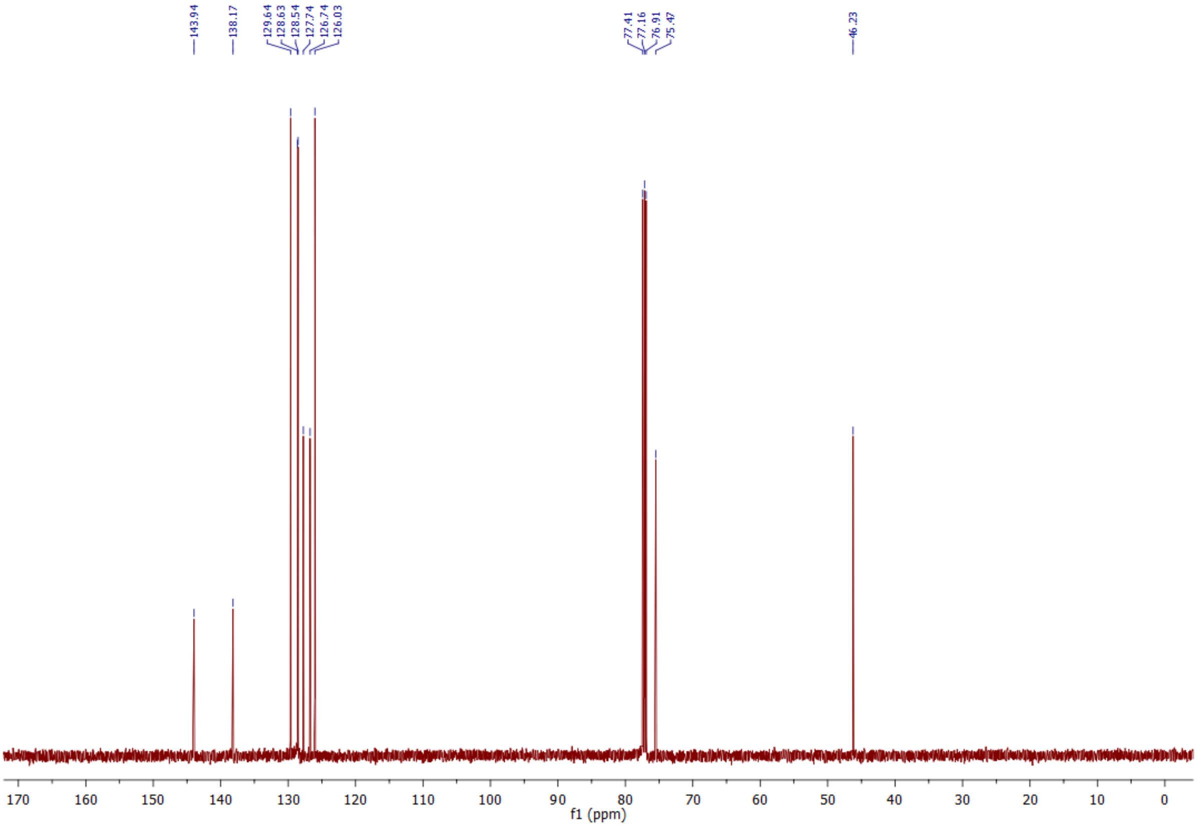

Supplementary Figure 131. FTMS spectrum of **2af** (ESI-TOF)

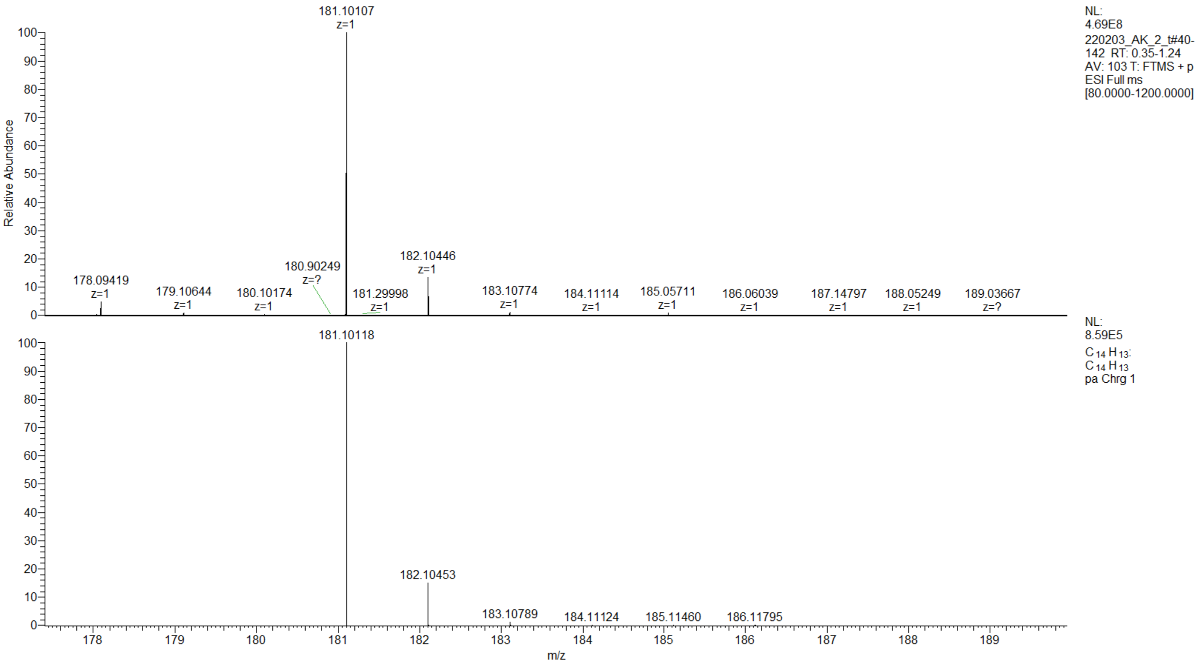

Supplementary Figure 132. ATR-FTIR spectrum of **2af** (neat)

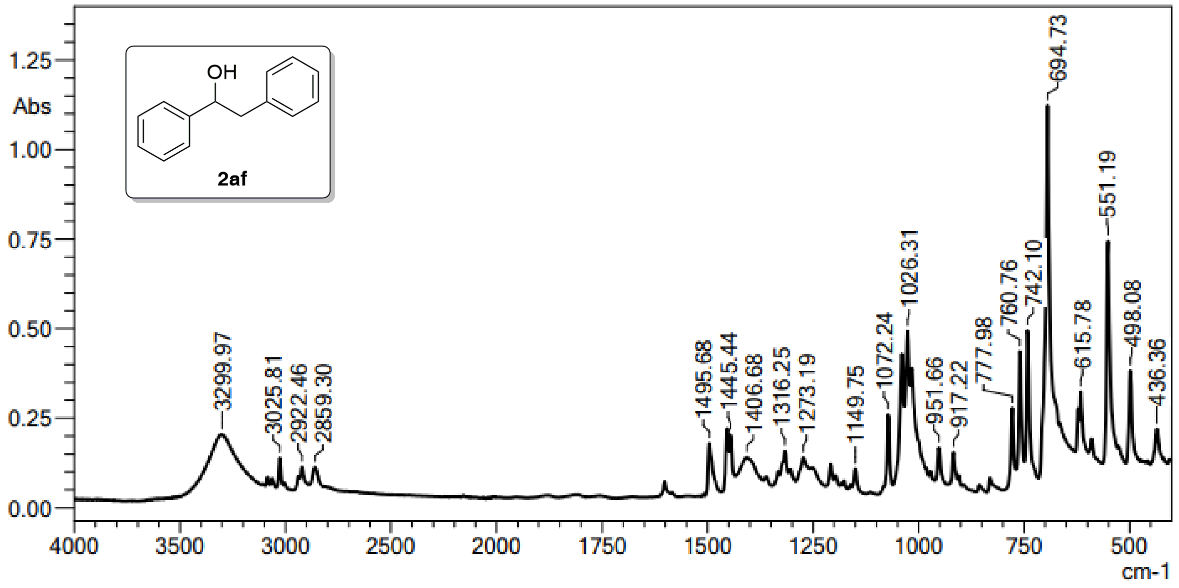

1-Phenylbutan-1-ol (2ag)

Supplementary Figure 133. ^{1}H NMR spectrum of **2ag** (500 MHz, $CDCl_3$)

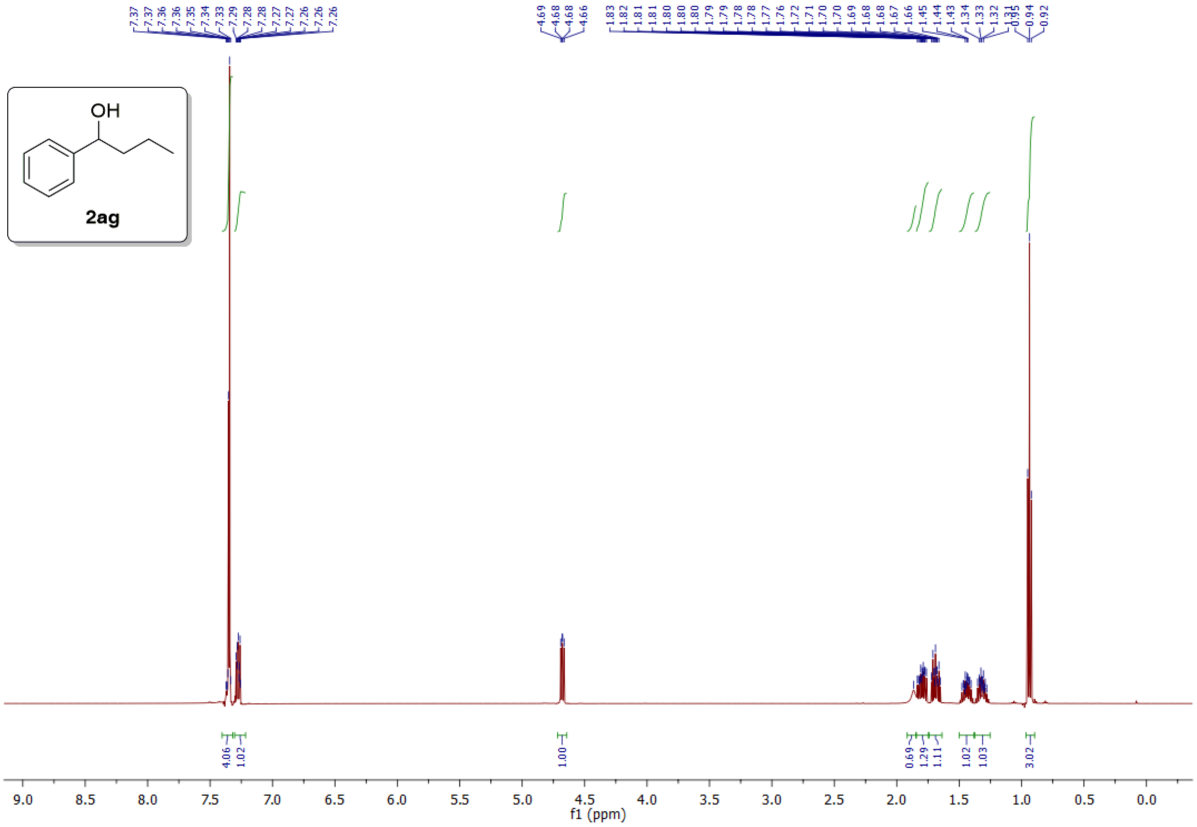

Supplementary Figure 134. $^{13}C\{^{1}H\}$ NMR spectrum of **2ag** (126 MHz, $CDCl_3$)

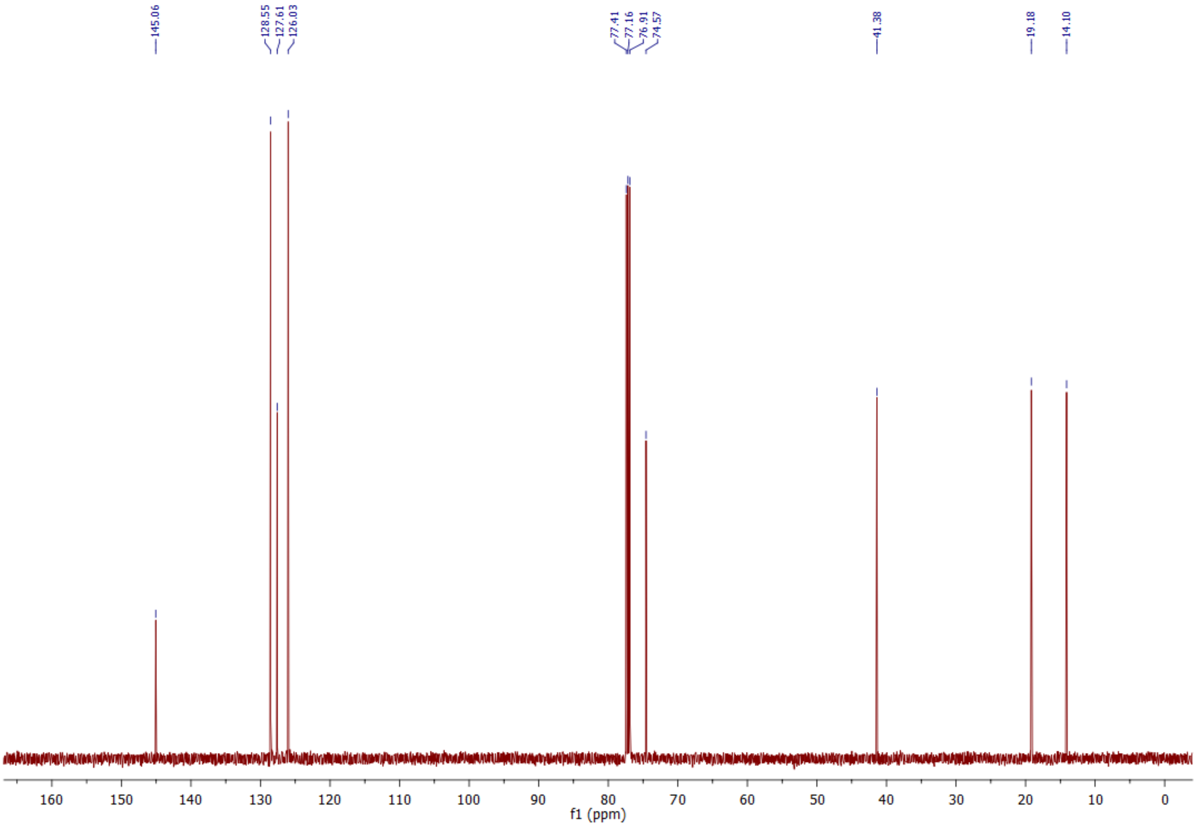

Supplementary Figure 135. FTMS spectrum of **2ag** (ESI-TOF)

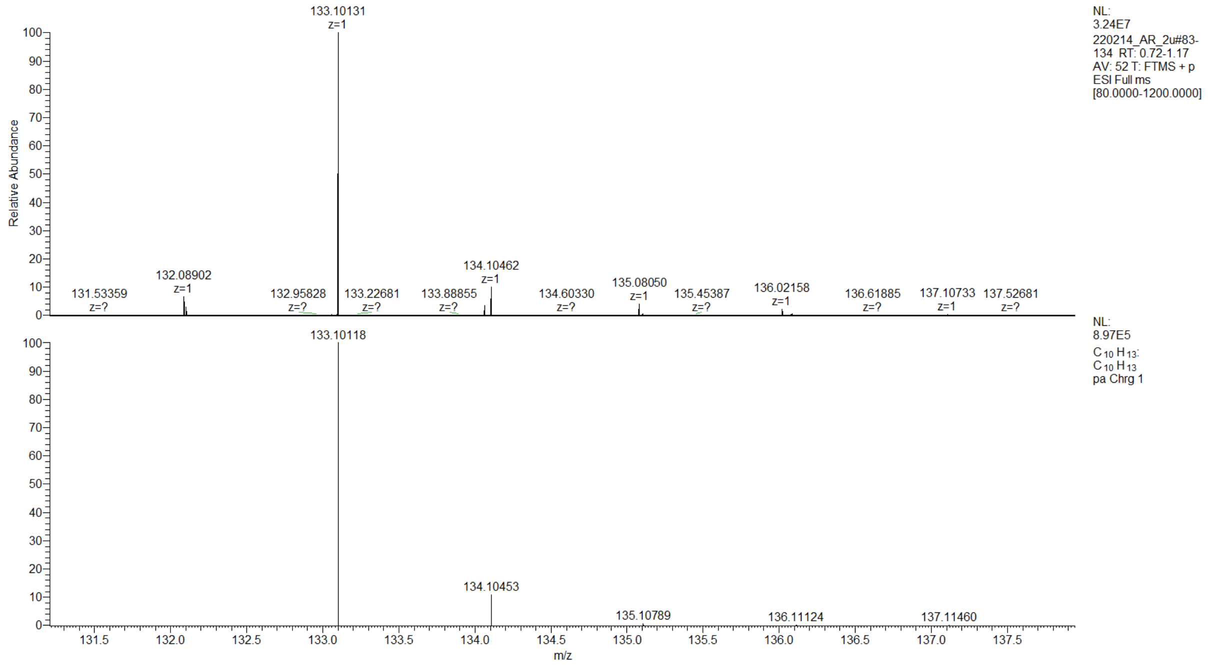

Supplementary Figure 136. ATR-FTIR spectrum of **2ag** (neat)

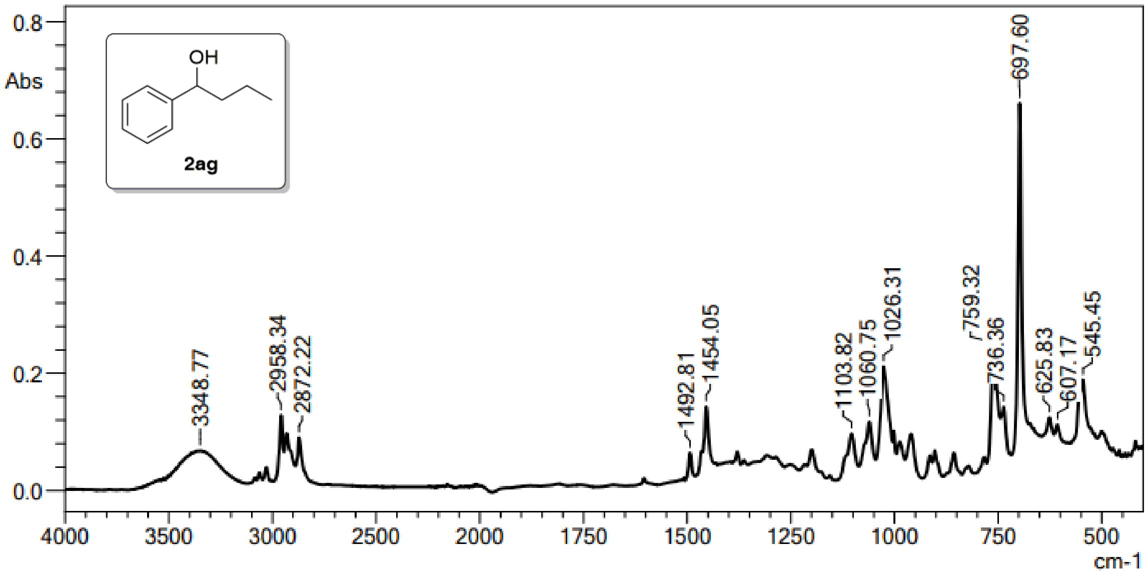

1-Phenylbut-3-en-1-ol (2ah)

Supplementary Figure 137. ^{1}H NMR spectrum of **2ah** (500 MHz, $CDCl_3$)

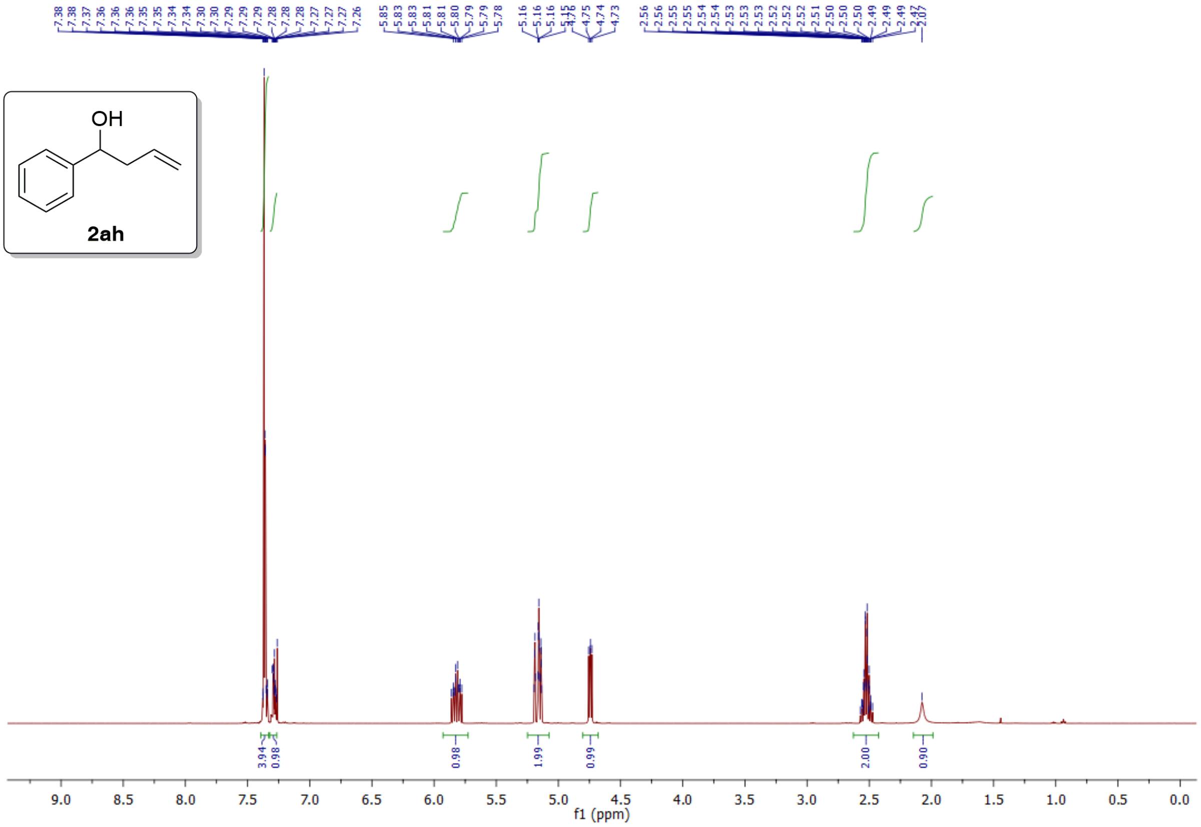

Supplementary Figure 138. $^{13}C\{^{1}H\}$ NMR spectrum of **2ah** (126 MHz, $CDCl_3$)

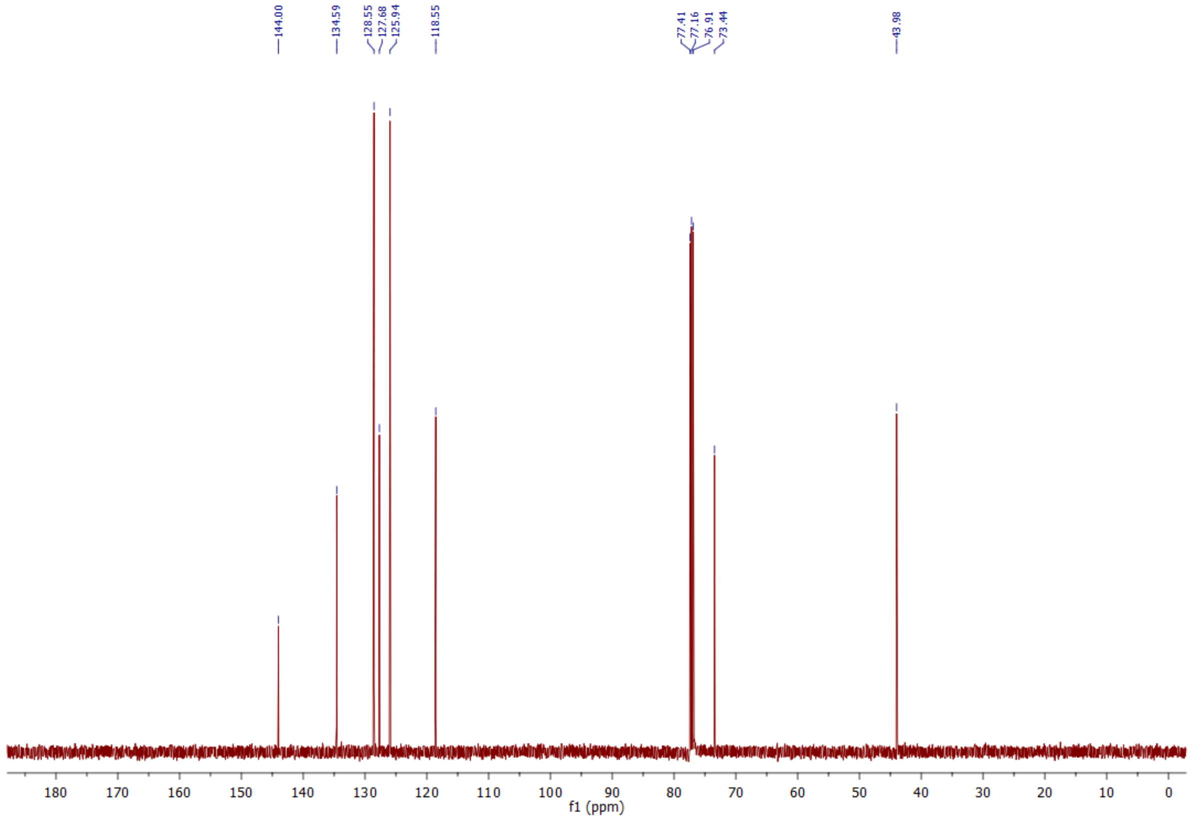

Supplementary Figure 139. FTMS spectrum of **2ah** (ESI-TOF)

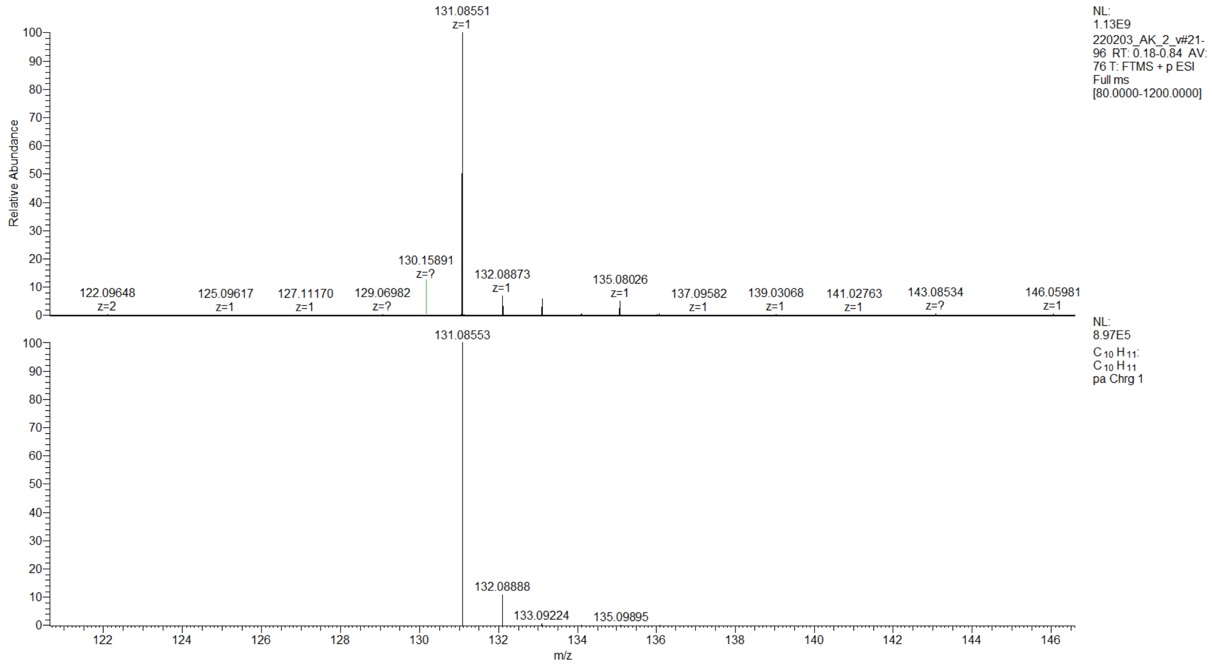

Supplementary Figure 140. ATR-FTIR spectrum of **2ah** (neat)

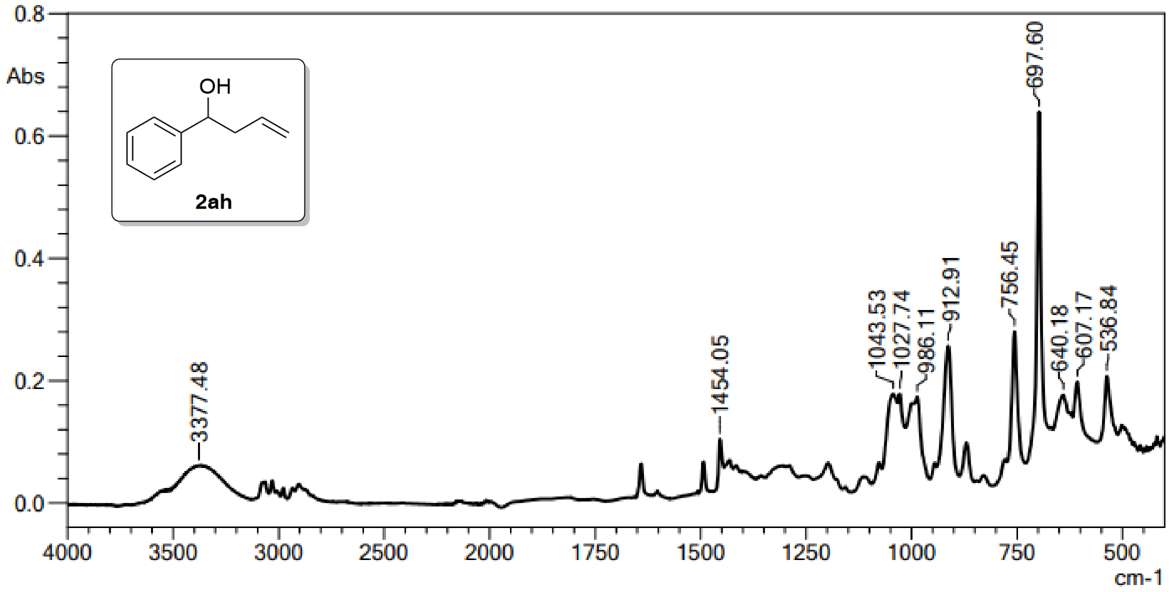

Supplement: Supplementary file 3 — Supplementary Data 1 [file 42004_2023_1013_MOESM3_ESM.pdf]
