# Supplementary material for: Biocatalytic characterization of an alcohol dehydrogenase variant deduced from Lactobacillus kefir in asymmetric hydrogen transfer
Source: Commun Chem. 2023 Oct 12;6:217. doi: 10.1038/s42004-023-01013-1 (PMC10570314; doi:10.1038/s42004-023-01013-1)
Supplement: Supplementary file 4 — Supplementary Data 2 [file 42004_2023_1013_MOESM4_ESM.pdf]

The Gaussian Input file for geometry optimization of 1-(6-chloro-9*H*-purin-9-yl)propan-2-one (**1w**) was performed using the standard set of parameters (theory: B3LYP, basis: 6-31G(d), charge: 0, and multiplicity: 1). The list of the Cartesian coordinates for the optimized structure of the ligand molecule **1w** is as follows:

%NProcShared=2
#n B3LYP/6-31G(d) Opt

Cartesian coordinates for the optimized structure of 1-(6-chloro-9*H*-purin-9-yl)propan-2-one (**1w**):

0 1

N	-0.20750	-2.39510	0.64830
C	0.72310	-1.49120	0.42350
N	0.19810	-0.22850	0.37190
C	-1.14900	-0.32860	0.57450
N	-2.05120	0.66750	0.59720
C	-3.29560	0.19920	0.83590
N	-3.68220	-1.07560	1.02640
C	-2.71480	-2.01150	0.97910
C	-1.38630	-1.68430	0.74990
Cl	-3.13920	-3.63600	1.20750
C	0.93930	0.99120	0.15780
C	1.38240	1.12130	-1.27660
C	0.33660	1.48050	-2.29910
O	2.55100	0.90840	-1.60330
H	1.77950	-1.69470	0.28800
H	-4.08460	0.94560	0.87980
H	0.32970	1.85520	0.43850
H	1.81510	0.97330	0.81520
H	0.81930	1.68110	-3.25980
H	-0.19390	2.38200	-1.98310
H	-0.36440	0.65160	-2.42020
