# Supplementary material for: Biocatalytic characterization of an alcohol dehydrogenase variant deduced from Lactobacillus kefir in asymmetric hydrogen transfer
Source: Commun Chem. 2023 Oct 12;6:217. doi: 10.1038/s42004-023-01013-1 (PMC10570314; doi:10.1038/s42004-023-01013-1)
Supplement: Supplementary file 5 — Supplementary Data 3 [file 42004_2023_1013_MOESM5_ESM.pdf]

Protein and DNA sequences of alcohol dehydrogenase variant deduced from *Lactobacillus kefir* (Lk-ADH Prince).

Native amino acid sequence.

MTDRLKGKVAIVTGGTLGIGLAIADKFVEEGAKVVITGRHADVGEKAAKSIGGTDVI
RFVQHDASDEAGWTKLFDTTEEAFGPVTTVVNNAGIAVSKSVEDTTTEEWRKLLSV
NLDGVFFGTRLGIQRMKNKGLGASIINMSSIFGLVGDPTLGAYNASKGAVRIMSKSA
ALDCALKDYDVRVNTVHPGCIKTPLVDDLEGAEEMMSQRTKTPMGHIGEPNDIAWI
CVYLASDESKFATGAEFVVDGGYTAQ

Amino acid sequence including Strep-Tag as used in this study.

MASWSHPQFEKGAETMTDRLKGKVAIVTGGTLGIGLAIADKFVEEGAKVVITGRHA
DVGEKAAKSIGGTDVIRFVQHDASDEAGWTKLFDTTEEAFGPVTTVVNNAGIAVSKS
VEDTTTEEWRKLLSVNLDGVFFGTRLGIQRMKNKGLGASIINMSSIFGLVGDPTLGAY
NASKGAVRIMSKSAALDCALKDYDVRVNTVHPGCIKTPLVDDLEGAEEMMSQRTKT
PMGHIGEPNDIAWICVYLASDESKFATGAEFVVDGGYTAQ

Codon-optimized DNA sequence used.

atggctagctggagccacccgcagttcgaaaaaggcgccgagaccATGACCGATCGTCTGAAAGGTAAAG
TTGCAATTGTTACCGGTGGCACCTTAGGTATTGGTCTGGCAATTGCAGATAAATTT
GTTGAAGAAGGTGCCAAAGTTGTTATTACCGGTCGTCATGCAGATGTTGGTGAAA
AAGCAGCAAAAAGCATTGGTGGCACCGATGTTATTCGTTTTGTTCAGCATGATGC
AAGTGATGAAGCAGGTTGGACCAAACTGTTTGATACCACCGAAGAAGCATTTGGT
CCGGTTACCACCGTTGTTAATAATGCAGGTATTGCAGTTAGCAAGAGCGTTGAAG
ATACCACCACAGAAGAATGGCGTAAACTGCTGAGCGTTAATCTGGATGGTGTTTT
TTTTGGCACCCGTCTGGGTATTCAGCGTATGAAAAACAAAGGTCTGGGTGCCAGC
ATTATCAATATGAGCAGCATTTTTGGTCTGGTTGGTGATCCGACACTGGGTGCAT
ATAATGCAAGCAAAGGTGCAGTTCGTATTATGAGCAAAAGCGCAGCACTGGATT
GTGCACTGAAAGATTATGATGTTCGTGTGAATACCGTTCATCCGGGTTGTATTAA
AACACCGCTGGTTGATGATCTGGAAGGTGCCGAAGAAATGATGAGCCAGCGTAC
CAAAACACCGATGGGTCATATTGGTGAACCGAATGATATTGCCTGGATTTGTGTT
TATCTGGCCAGTGATGAAAGTAAATTTGCGACCGGTGCCGAATTTGTTGTTGATG
GTGGTTATACCGCACAGTAAggtctctgatatctaactaagcttgacctg
