# Supplementary material for: Biocatalytic characterization of an alcohol dehydrogenase variant deduced from Lactobacillus kefir in asymmetric hydrogen transfer
Source: Commun Chem. 2023 Oct 12;6:217. doi: 10.1038/s42004-023-01013-1 (PMC10570314; doi:10.1038/s42004-023-01013-1)
Supplement: Supplementary file 6 — Supplementary Data 4 [file 42004_2023_1013_MOESM6_ESM.pdf]

The copies of HPLC chromatograms.

Supplementary Figure 141. HPLC of *rac*-2a on Chiralpak AD-H at 25 °C

Conditions: *n*-hexane-2-PrOH-DEA (78:22:0.1, v/v); f=1.0 mL/min; λ=273 nm; *p*=5.5 MPa

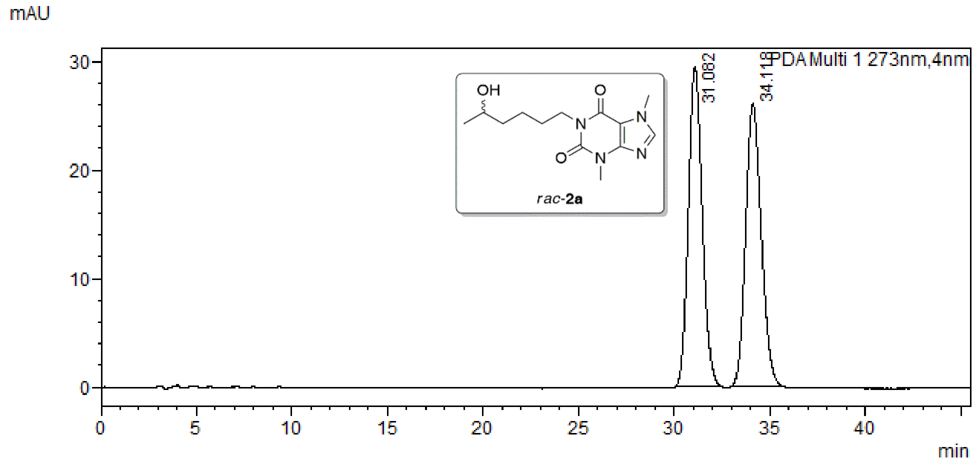

Peak Table

PDA Ch1 273nm

Peak#	Ret. Time	Area	Height	Height%	Area%
1	31.082	1505440	29469	53.080	50.138
2	34.118	1497143	26049	46.920	49.862
Total		3002583	55518	100.000	100.000

Supplementary Figure 142. HPLC of (*R*)-2a on Chiralpak AD-H at 25 °C

Conditions: *n*-hexane-2-PrOH-DEA (78:22:0.1, v/v); f=1.0 mL/min; λ=273 nm; *p*=5.5 MPa

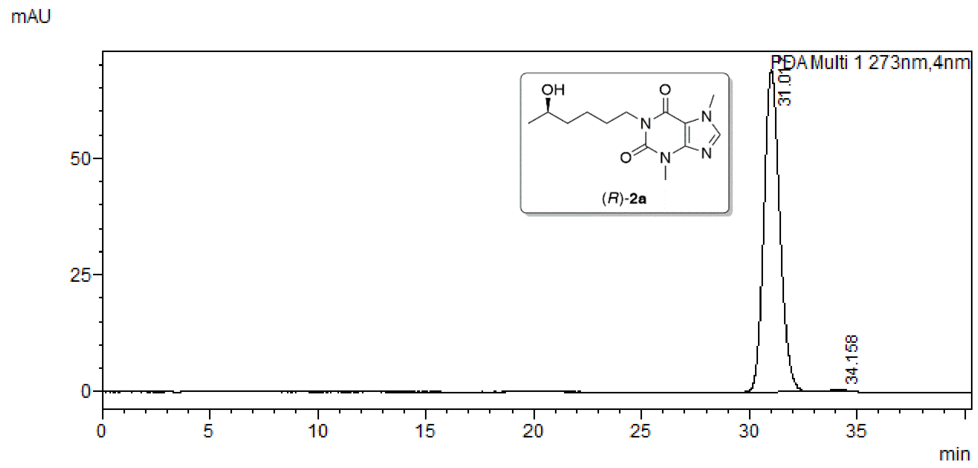

Peak Table

PDA Ch1 273nm

Peak#	Ret. Time	Area	Height	Height%	Area%
1	31.017	3552976	68874	99.334	99.369
2	34.158	22565	462	0.666	0.631
Total		3575541	69336	100.000	100.000

Supplementary Figure 143. HPLC of *rac*-2b on Chiralcel OD-H at 30 °C

Conditions: *n*-hexane-2-PrOH (97:3, v/v); f=1.0 mL/min; λ=210 nm; *p*=4.4 MPa

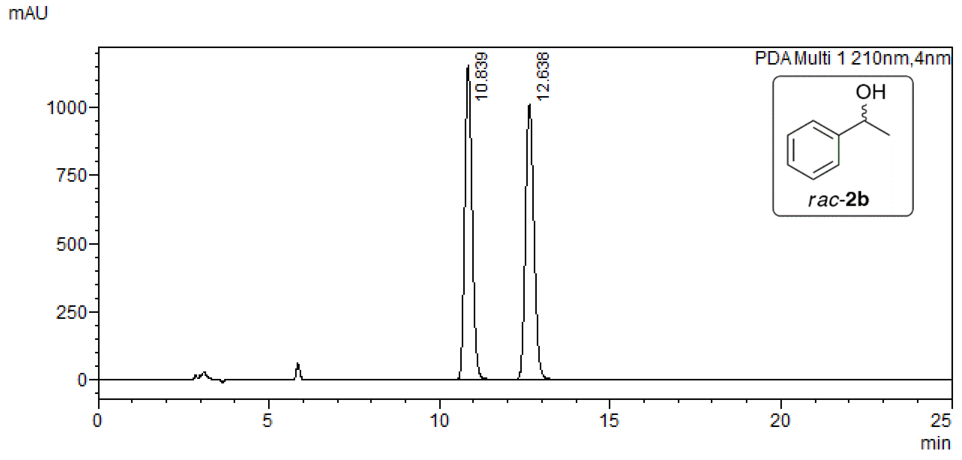

Peak Table

PDA Ch1 210nm

Peak#	Ret. Time	Area	Height	Height%	Area%
1	10.839	17291179	1152729	53.329	49.622
2	12.638	17554399	1008811	46.671	50.378
Total		34845578	2161540	100.000	100.000

Supplementary Figure 144. HPLC of commercial (*S*)-1-phenyl ethanol [(*S*)-2b] (>99% ee) on Chiralcel OD-H at 30 °C

Conditions: *n*-hexane-2-PrOH (97:3, v/v); f=1.0 mL/min; λ=210 nm; *p*=4.4 MPa

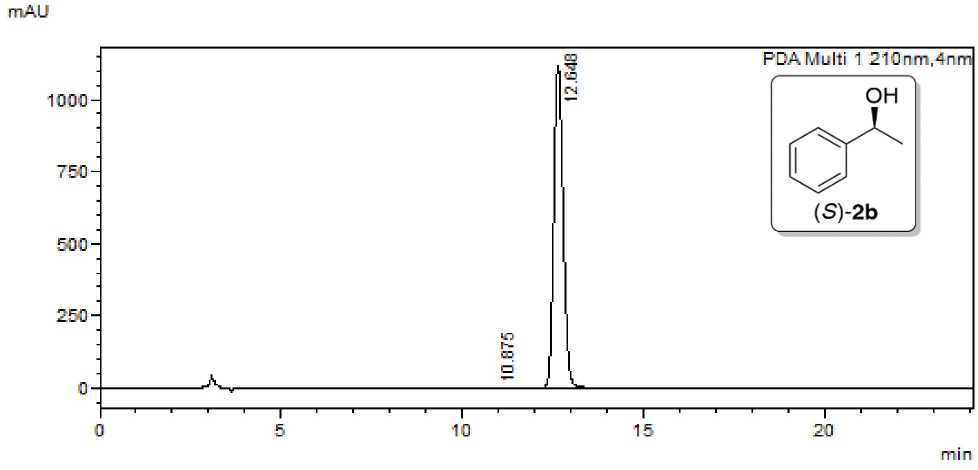

Peak Table

PDA Ch1 210nm

Peak#	Ret. Time	Area	Height	Height%	Area%
1	10.875	39280	2210	0.198	0.195
2	12.648	20132480	1113834	99.802	99.805
Total		20171760	1116044	100.000	100.000

Supplementary Figure 145. HPLC of *rac*-2b (after enzymatic reaction) on Chiralcel OD-H at 30 °C

Conditions: *n*-hexane-2-PrOH (97:3, v/v); f=1.0 mL/min; λ=210 nm; *p*=4.4 MPa

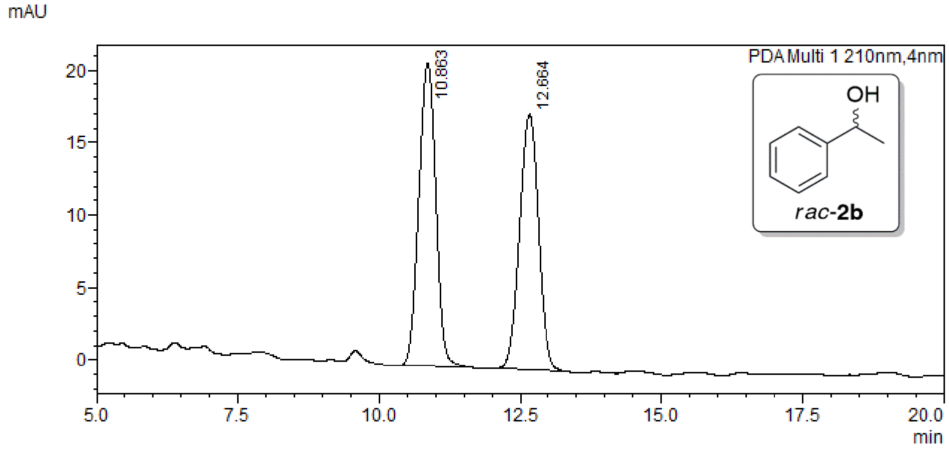

Peak Table

PDA Ch1 210nm

Peak#	Ret. Time	Area	Height	Height%	Area%
1	10.863	435889	20892	54.269	51.392
2	12.664	412271	17605	45.731	48.608
Total		848160	38497	100.000	100.000

Supplementary Figure 146. HPLC of *rac*-2c on Chiralcel OD-H at 30 °C

Conditions: *n*-hexane-2-PrOH (98:2, v/v); f=1.0 mL/min; λ=220 nm; *p*=4.4 MPa

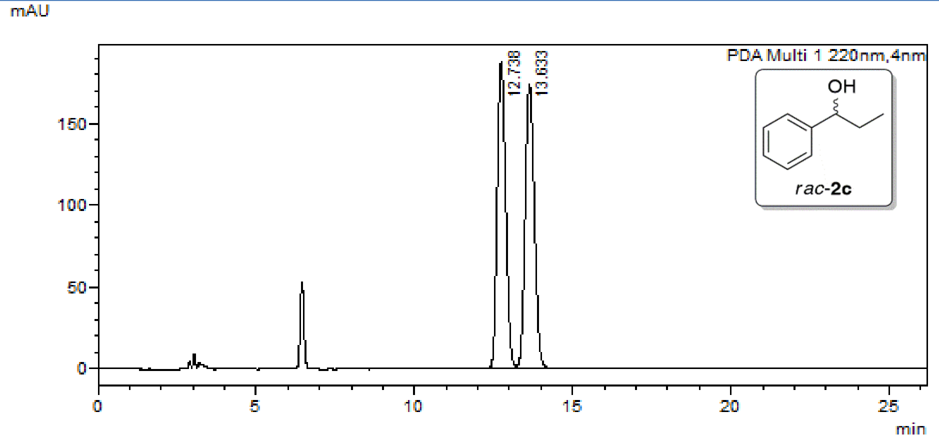

Peak Table

PDA Ch1 220nm

Peak#	Ret. Time	Area	Height	Height%	Area%
1	12.738	3453257	186863	51.831	49.656
2	13.633	3501162	173662	48.169	50.344
Total		6954419	360524	100.000	100.000

Supplementary Figure 147. HPLC of commercial (*S*)-1-phenyl-1-propanol [(*S*)-2c] (>99% ee) on Chiralcel OD-H at 30 °C

Conditions: *n*-hexane-2-PrOH (98:2, v/v); f=1.0 mL/min; λ=220 nm; *p*=4.4 MPa

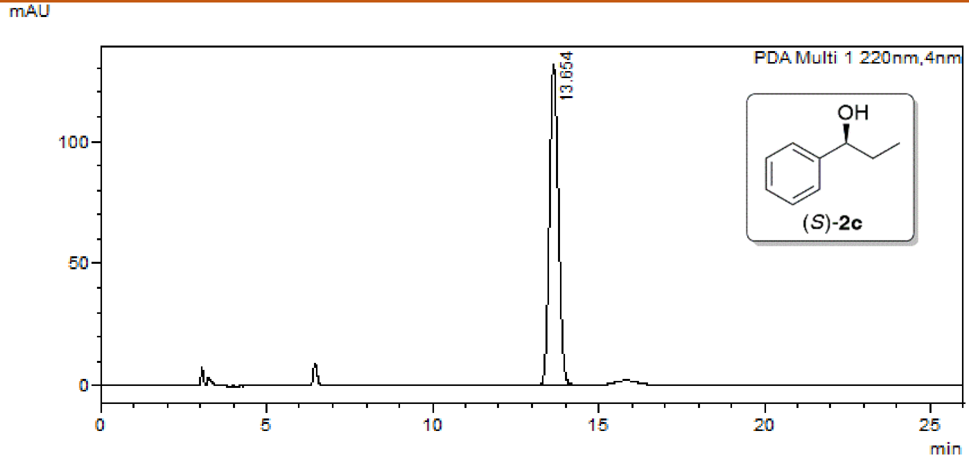

Peak Table

PDA Ch1 220nm

Peak#	Ret. Time	Area	Height	Height%	Area%
1	13.654	2488193	130850	100.000	100.000
Total		2488193	130850	100.000	100.000

Supplementary Figure 148. HPLC of *rac*-2c (after enzymatic reaction) on Chiralcel OD-H at 30 °C

Conditions: *n*-hexane-2-PrOH (98:2, v/v); f=1.0 mL/min; λ=220 nm; *p*=4.4 MPa

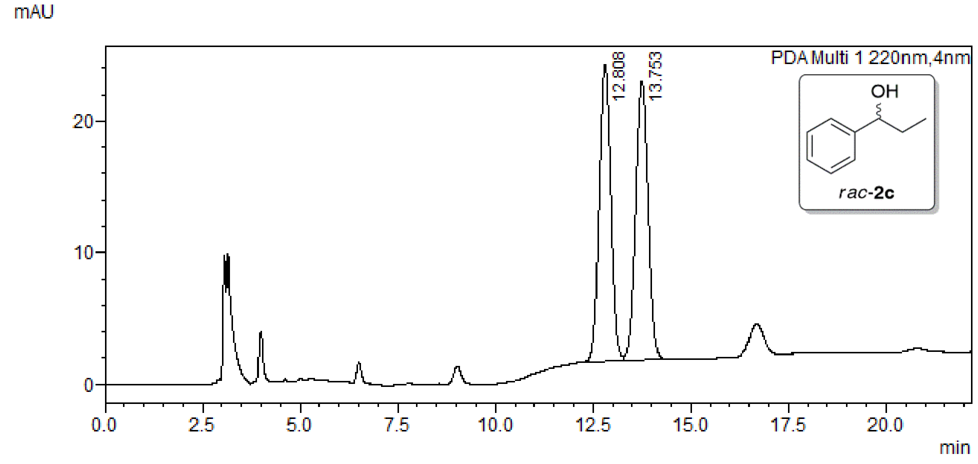

Peak Table

PDA Ch1 220nm

Peak#	Ret. Time	Area	Height	Height%	Area%
1	12.808	466608	22452	51.479	49.557
2	13.753	474953	21162	48.521	50.443
Total		941560	43614	100.000	100.000

Supplementary Figure 149. HPLC of *rac*-2d on Chiralcel OJ-H at 30 °C

Conditions: *n*-hexane-2-PrOH (90:10, v/v); f=1.0 mL/min; λ=222 nm; *p*=4.5 MPa

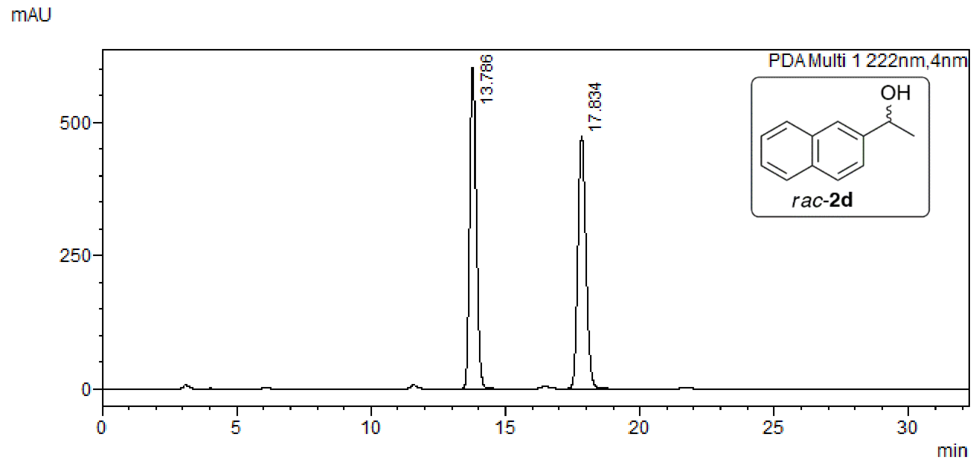

Peak Table

PDA Ch1 222nm

Peak#	Ret. Time	Area	Height	Height%	Area%
1	13.786	9940681	601710	55.938	49.767
2	17.834	10033767	473963	44.062	50.233
Total		19974447	1075673	100.000	100.000

Supplementary Figure 150. HPLC of commercial (*S*)-α-methyl-2-naphtalenemethanol [(*S*)-2d] (98% ee) on Chiralcel OJ-H at 30 °C

Conditions: *n*-hexane-2-PrOH (90:10, v/v); f=1.0 mL/min; λ=222 nm; *p*=4.5 MPa

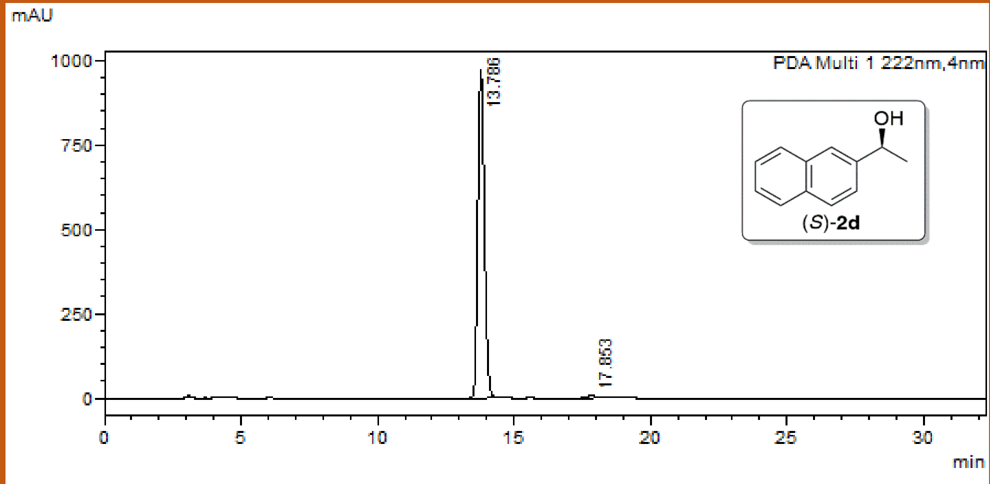

Peak Table

PDA Ch1 222nm

Peak#	Ret. Time	Area	Height	Height%	Area%
1	13.786	16145496	969798	99.197	99.024
2	17.853	159099	7850	0.803	0.976
Total		16304596	977647	100.000	100.000

Supplementary Figure 151. HPLC of (*R*)-2d (after enzymatic reaction) on Chiralcel OJ-H at 30 °C

Conditions: *n*-hexane-2-PrOH (90:10, v/v); f=1.0 mL/min; λ=222 nm; *p*=4.5 MPa

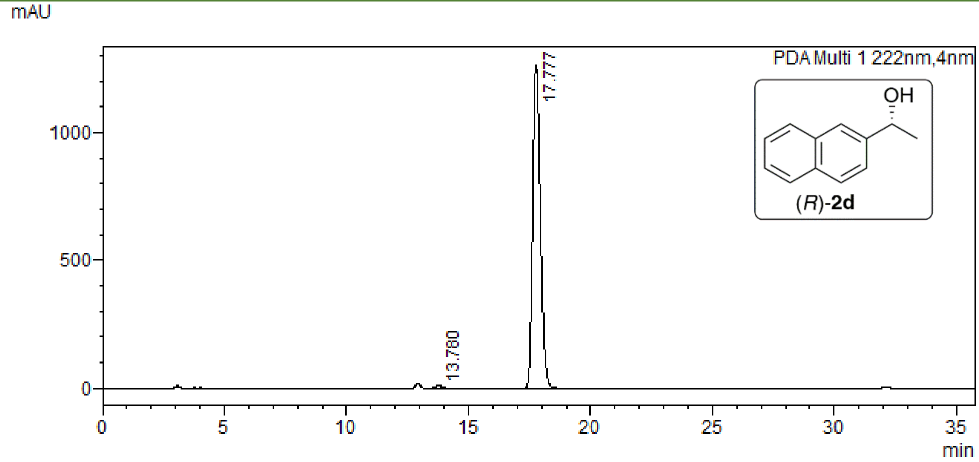

Peak Table

PDA Ch1 222nm

Peak#	Ret. Time	Area	Height	Height%	Area%
1	13.780	202275	12747	0.999	0.723
2	17.777	27767298	1263577	99.001	99.277
Total		27969573	1276323	100.000	100.000

Supplementary Figure 152. HPLC of *rac*-2e on Chiralcel OD-H at 30 °C

Conditions: *n*-hexane-2-PrOH (98:2, v/v); f=1.0 mL/min; λ=205 nm; *p*=4.4 MPa

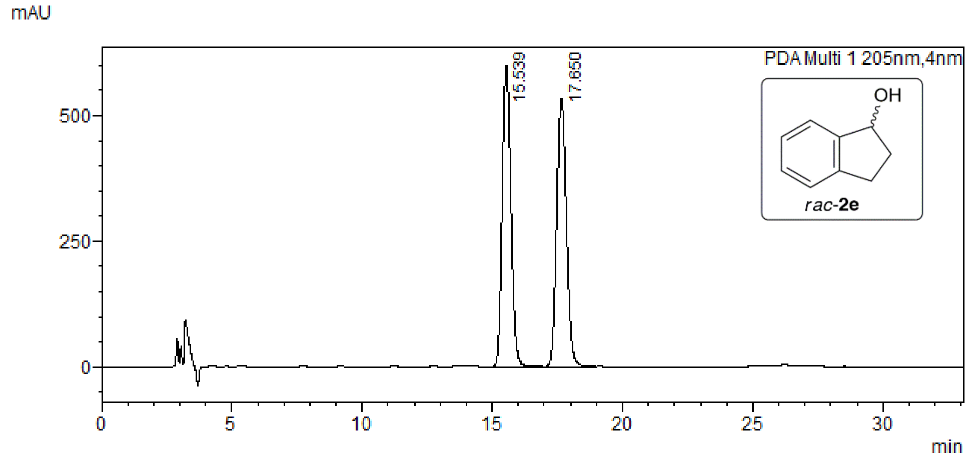

Peak Table

PDA Ch1 205nm

Peak#	Ret. Time	Area	Height	Height%	Area%
1	15.539	13486919	598567	52.909	50.011
2	17.650	13480835	532743	47.091	49.989
Total		26967754	1131309	100.000	100.000

Supplementary Figure 153. HPLC of commercial (*R*)-1-indanol [(*R*)-2e] (>99% ee) on Chiralcel OD-H at 30 °C

Conditions: *n*-hexane-2-PrOH (98:2, v/v); f=1.0 mL/min; λ=205 nm; *p*=4.4 MPa

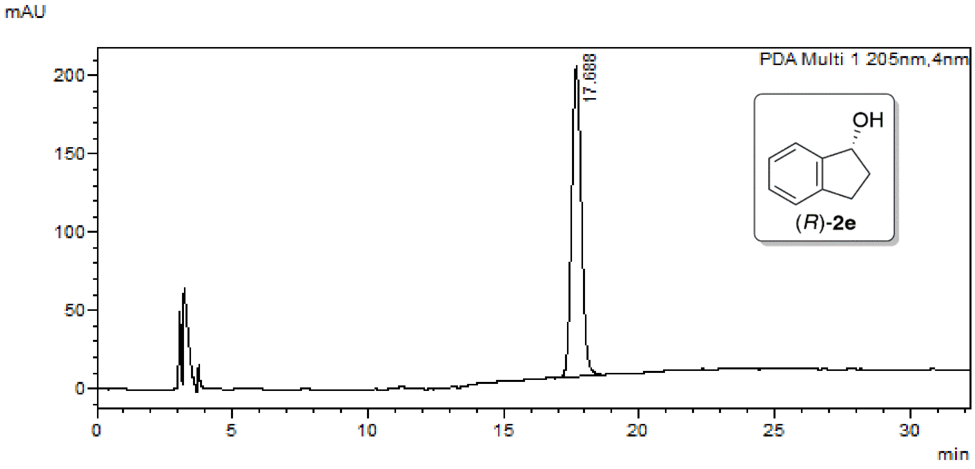

Peak Table

PDA Ch1 205nm

Peak#	Ret. Time	Area	Height	Height%	Area%
1	17.688	4938467	198354	100.000	100.000
Total		4938467	198354	100.000	100.000

Supplementary Figure 154. HPLC of *rac*-2e (after enzymatic reaction) on Chiralcel OD-H at 30 °C

Conditions: *n*-hexane-2-PrOH (98:2, v/v); f=1.0 mL/min; λ=205 nm; *p*=4.4 MPa

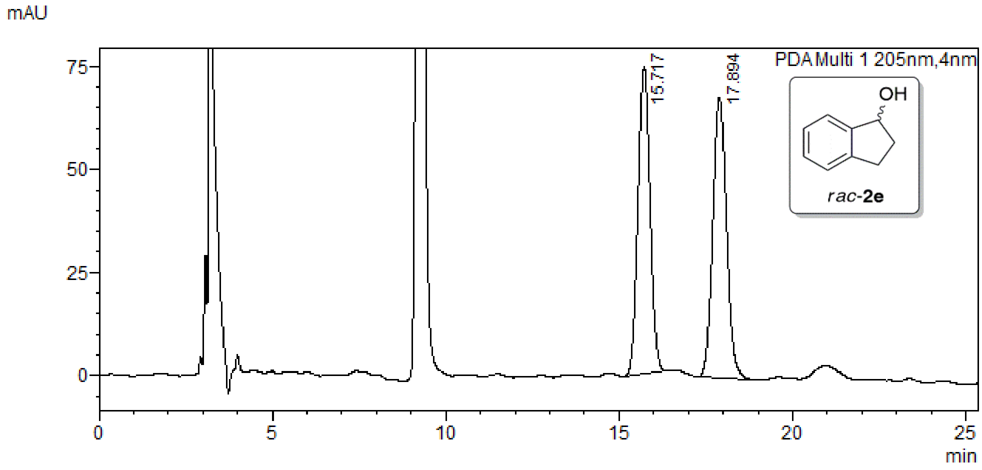

Peak Table

PDA Ch1 205nm

Peak#	Ret. Time	Area	Height	Height%	Area%
1	15.717	1803568	74419	52.195	49.112
2	17.894	1868752	68158	47.805	50.888
Total		3672320	142577	100.000	100.000

Supplementary Figure 155. HPLC of *rac*-2f on Chiralcel OJ-H at 30 °C

Conditions: *n*-hexane-2-PrOH (90:10, v/v); f=0.8 mL/min; λ=220 nm; *p*=3.6 MPa

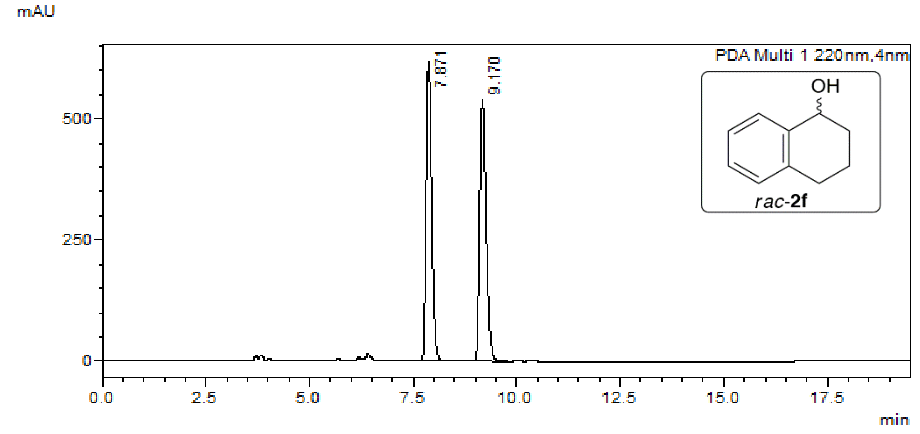

Peak Table

PDA Ch1 220nm

Peak#	Ret. Time	Area	Height	Height%	Area%
1	7.871	5819296	616676	53.410	49.580
2	9.170	5917858	537941	46.590	50.420
Total		11737154	1154616	100.000	100.000

Supplementary Figure 156. HPLC of commercial (*R*)-1,2,3,4-tetrahydro-1-napthol [(*R*)-2f] (>99% ee) on Chiralcel OJ-H at 30 °C

Conditions: *n*-hexane-2-PrOH (90:10, v/v); f=0.8 mL/min; λ=220 nm; *p*=3.6 MPa

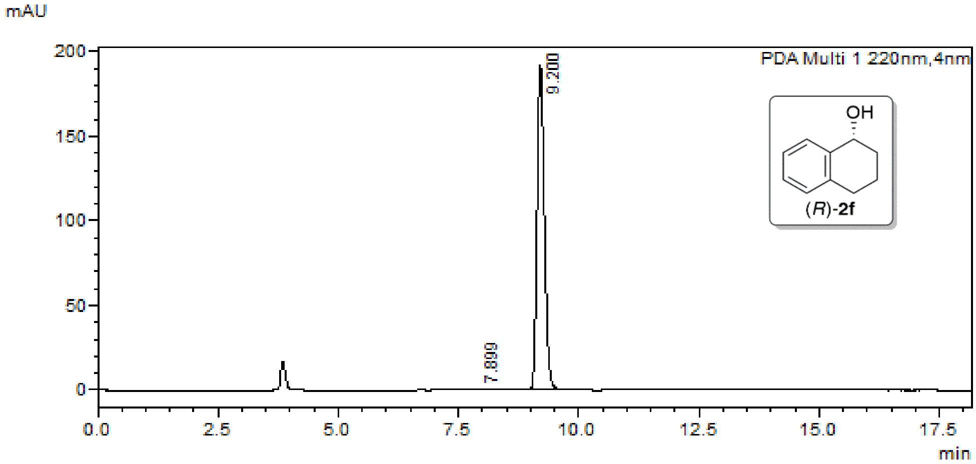

Peak Table

PDA Ch1 220nm

Peak#	Ret. Time	Area	Height	Height%	Area%
1	7.899	10420	722	0.376	0.521
2	9.200	1989238	191204	99.624	99.479
Total		1999659	191926	100.000	100.000

Supplementary Figure 157. HPLC of *rac*-2f (after enzymatic reaction) on Chiralcel OJ-H at 30 °C

Conditions: *n*-hexane-2-PrOH (90:10, v/v); f=0.8 mL/min; λ=220 nm; *p*=3.6 MPa

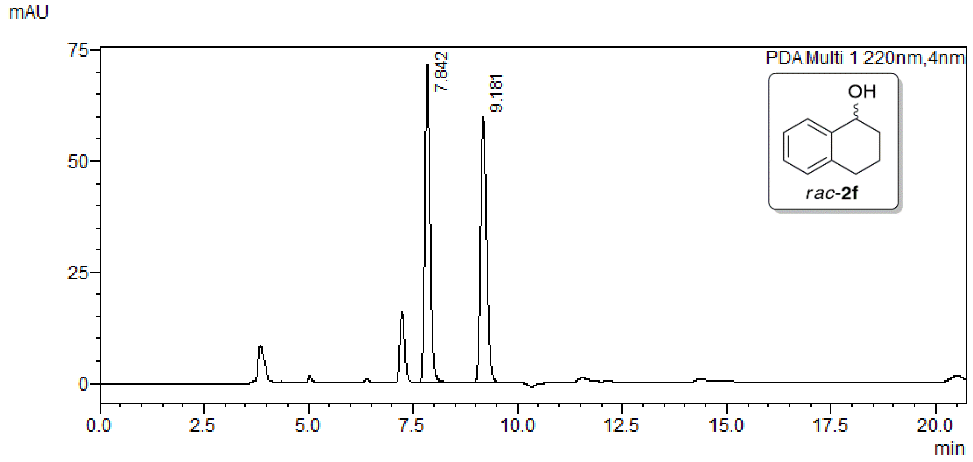

Peak Table

PDA Ch1 220nm

Peak#	Ret. Time	Area	Height	Height%	Area%
1	7.842	620371	71349	54.469	50.245
2	9.181	614316	59642	45.531	49.755
Total		1234687	130991	100.000	100.000

Supplementary Figure 158. HPLC of *rac*-2g on Chiralcel OD-H at 30 °C

Conditions: *n*-hexane-2-PrOH (98:2, v/v); f=0.8 mL/min; λ=210 nm; *p*=3.5 MPa

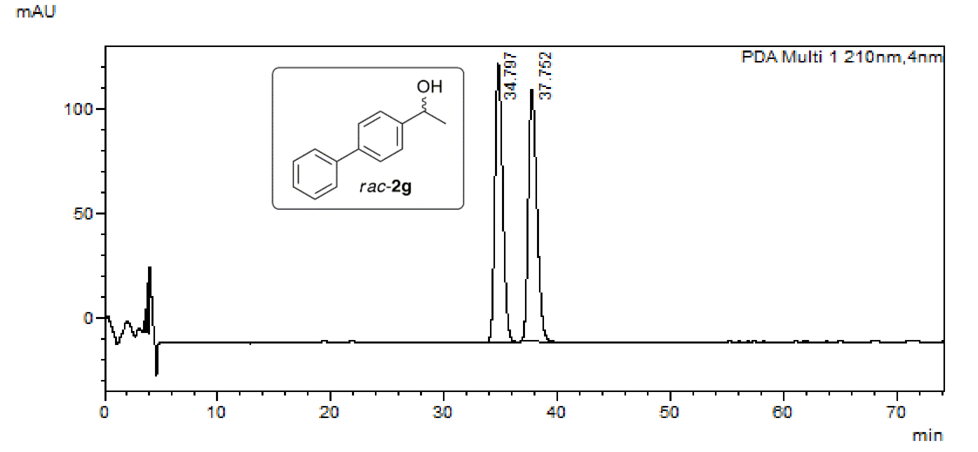

Peak Table

PDA Ch1 210nm

Peak#	Ret. Time	Area	Height	Height%	Area%
1	34.797	6304103	132711	52.423	49.834
2	37.752	6346220	120442	47.577	50.166
Total		12650323	253153	100.000	100.000

Supplementary Figure 159. HPLC of (*R*)-2g on Chiralcel OD-H at 30 °C

Conditions: *n*-hexane-2-PrOH (98:2, v/v); f=0.8 mL/min; λ=210 nm; *p*=3.5 MPa

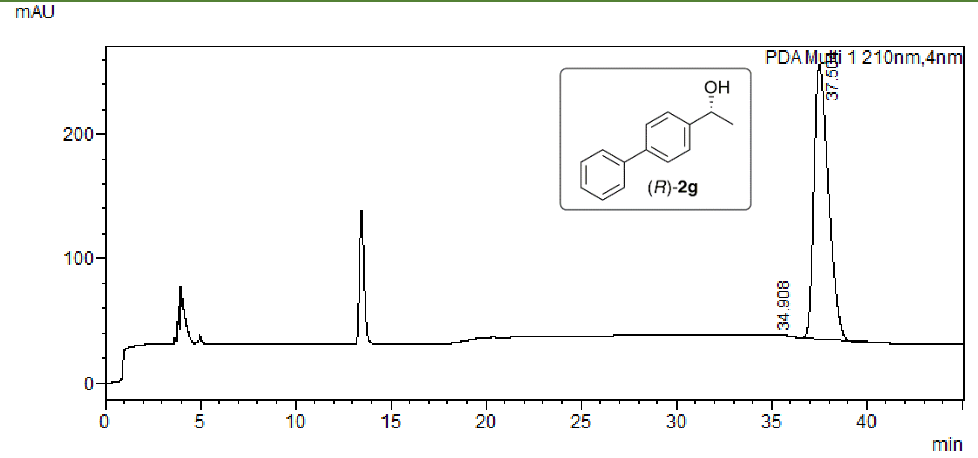

Peak Table

PDA Ch1 210nm

Peak#	Ret. Time	Area	Height	Height%	Area%
1	34.908	12398	371	0.168	0.105
2	37.504	11774340	220561	99.832	99.895
Total		11786738	220932	100.000	100.000

Supplementary Figure 160. HPLC of *rac*-2h on Chiralcel OD-H at 30 °C

Conditions: *n*-hexane-2-PrOH (95:5, v/v); f=0.9 mL/min; λ=210 nm; *p*=4.1 MPa

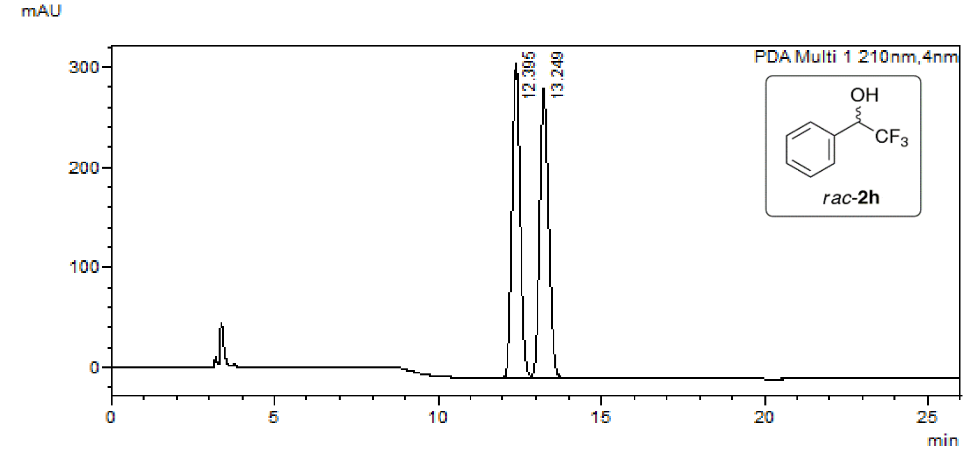

Peak Table

PDA Ch1 210nm

Peak#	Ret. Time	Area	Height	Height%	Area%
1	12.395	5509195	313286	51.937	49.819
2	13.249	5549241	289914	48.063	50.181
Total		11058436	603201	100.000	100.000

Supplementary Figure 161. HPLC of (*S*)-2h on Chiralcel OD-H at 30 °C

Conditions: *n*-hexane-2-PrOH (95:5, v/v); f=0.9 mL/min; λ=210 nm; *p*=4.1 MPa

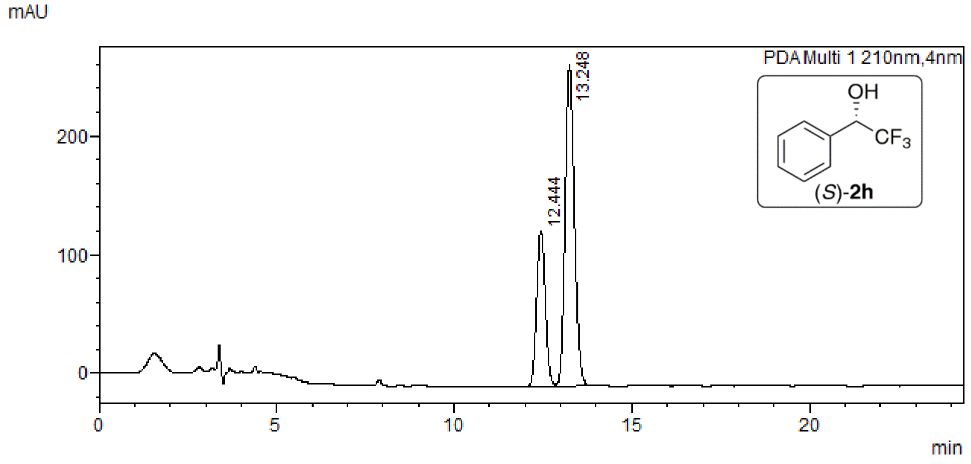

Peak Table

PDA Ch1 210nm

Peak#	Ret. Time	Area	Height	Height%	Area%
1	12.444	2134572	130377	32.565	30.455
2	13.248	4874388	269987	67.435	69.545
Total		7008960	400363	100.000	100.000

Supplementary Figure 162. HPLC of *rac*-2i on Chiralcel OD-H at 30 °C

Conditions: *n*-hexane-2-PrOH (98:2, v/v); f=0.8 mL/min; λ=210 nm; *p*=3.5 MPa

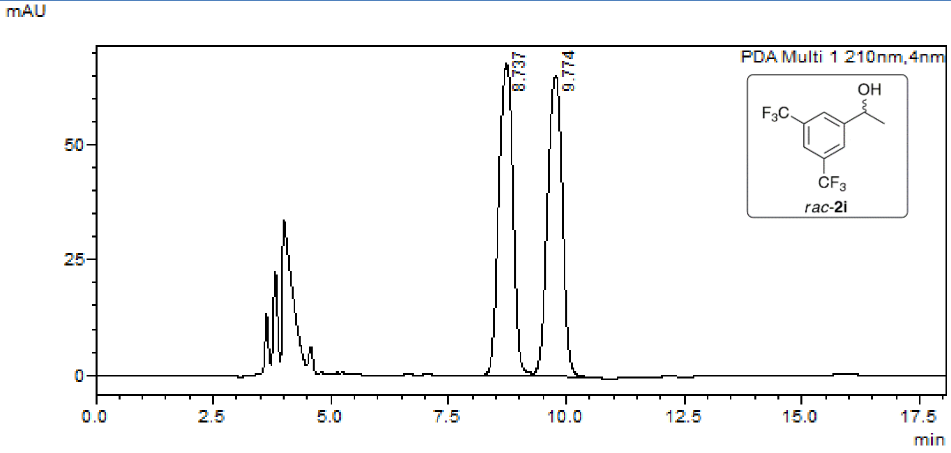

Peak Table

PDA Ch1 210nm

Peak#	Ret. Time	Area	Height	Height%	Area%
1	8.737	1430425	67618	50.769	49.642
2	9.774	1451039	65570	49.231	50.358
Total		2881464	133188	100.000	100.000

Supplementary Figure 163. HPLC of (*R*)-2i on Chiralcel OD-H at 30 °C

Conditions: *n*-hexane-2-PrOH (98:2, v/v); f=0.8 mL/min; λ=210 nm; *p*=3.5 MPa

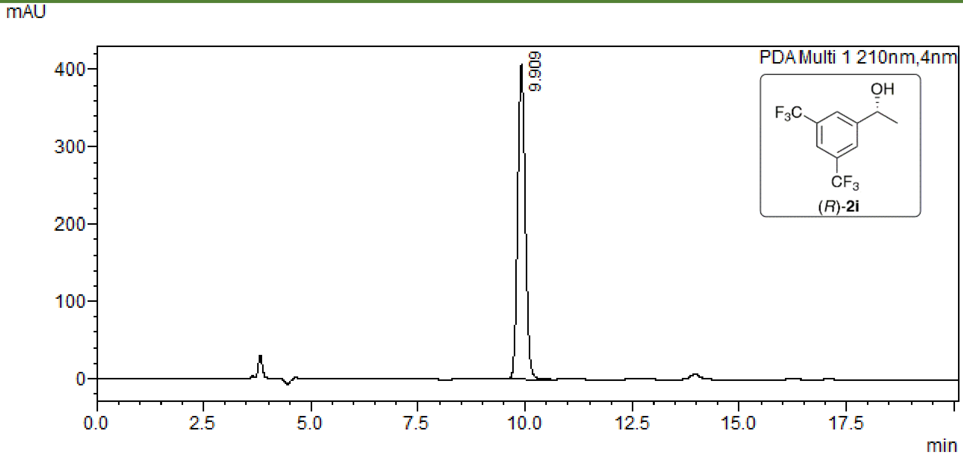

Peak Table

PDA Ch1 210nm

Peak#	Ret. Time	Area	Height	Height%	Area%
1	9.909	5067386	407279	100.000	100.000
Total		5067386	407279	100.000	100.000

Supplementary Figure 164. HPLC of *rac*-2j on Chiralcel OD-H at 30 °C

Conditions: *n*-hexane-2-PrOH (99:1, v/v); f=0.8 mL/min; λ=205 nm; *p*=3.5 MPa

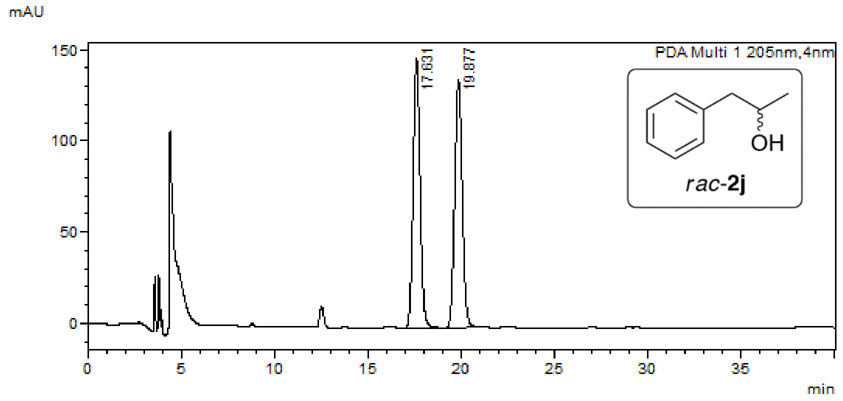

Peak Table

PDA Ch1 205nm

Peak#	Ret. Time	Area	Height	Height%	Area%
1	17.631	3903035	147796	52.085	50.101
2	19.877	3887308	135963	47.915	49.899
Total		7790344	283759	100.000	100.000

Supplementary Figure 165. HPLC of commercial (*R*)-1-phenyl-2-propanol [(*R*)-2j] (>99% ee) on Chiralcel OD-H at 30 °C

Conditions: *n*-hexane-2-PrOH (99:1, v/v); f=0.8 mL/min; λ=205 nm; *p*=3.5 MPa

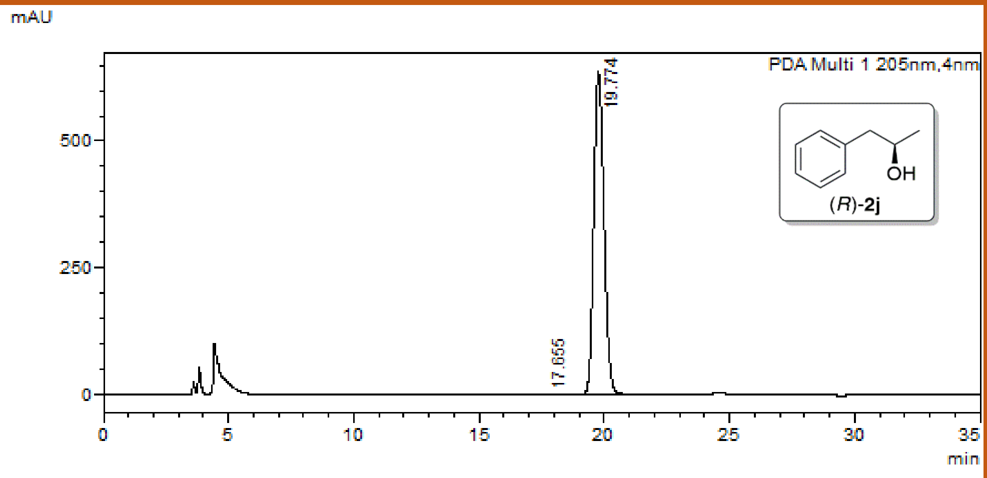

Peak Table

PDA Ch1 205nm

Peak#	Ret. Time	Area	Height	Height%	Area%
1	17.655	37979	1398	0.218	0.210
2	19.774	18071422	638649	99.782	99.790
Total		18109401	640047	100.000	100.000

Supplementary Figure 166. HPLC of (*R*)-2j on Chiralcel OD-H at 30 °C

Conditions: *n*-hexane-2-PrOH (99:1, v/v); f=0.8 mL/min; λ=205 nm; *p*=3.5 MPa

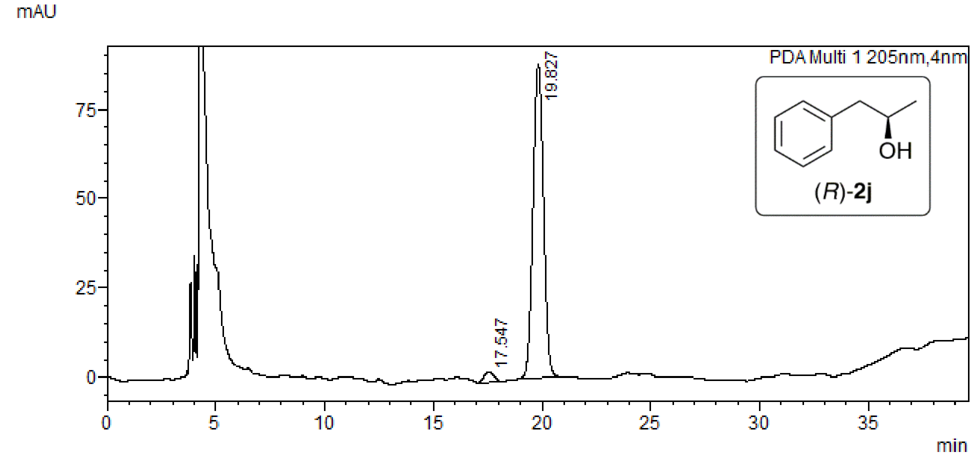

Peak Table

PDA Ch1 205nm

Peak#	Ret. Time	Area	Height	Height%	Area%
1	17.547	90792	2913	3.214	2.963
2	19.827	2973382	87717	96.786	97.037
Total		3064174	90630	100.000	100.000

Supplementary Figure 167. HPLC of *rac*-2k on Chiralcel OD-H at 30 °C

Conditions: *n*-hexane-2-PrOH (90:10, v/v); f=0.8 mL/min; λ=210 nm; *p*=3.7 MPa

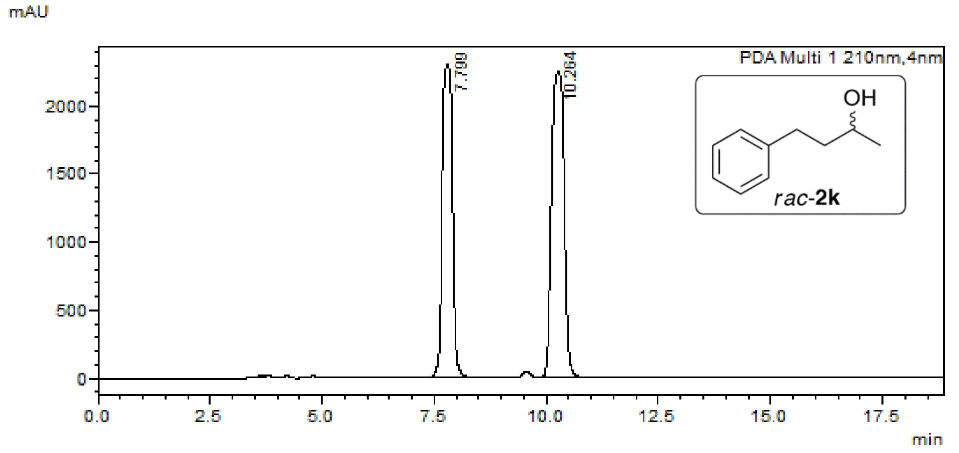

Peak Table

PDA Ch1 210nm

Peak#	Ret. Time	Area	Height	Height%	Area%
1	7.799	36783040	2300726	50.529	45.668
2	10.264	43760595	2252513	49.471	54.332
Total		80543635	4553239	100.000	100.000

Supplementary Figure 168. HPLC of commercial (*R*)-1-phenyl-3-butanol [(*R*)-2k] (>99% ee) on Chiralcel OD-H at 30 °C

Conditions: *n*-hexane-2-PrOH (90:10, v/v); f=0.8 mL/min; λ=210 nm; *p*=3.7 MPa

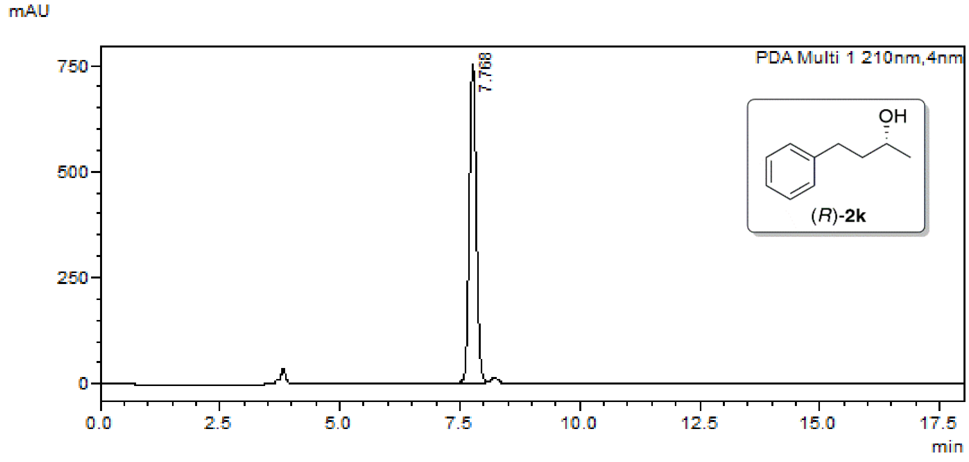

Peak Table

PDA Ch1 210nm

Peak#	Ret. Time	Area	Height	Height%	Area%
1	7.768	7657811	750129	100.000	100.000
Total		7657811	750129	100.000	100.000

Supplementary Figure 169. HPLC of (*R*)-2k on Chiralcel OD-H at 30 °C

Conditions: *n*-hexane-2-PrOH (90:10, v/v); f=0.8 mL/min; λ=210 nm; *p*=3.7 MPa

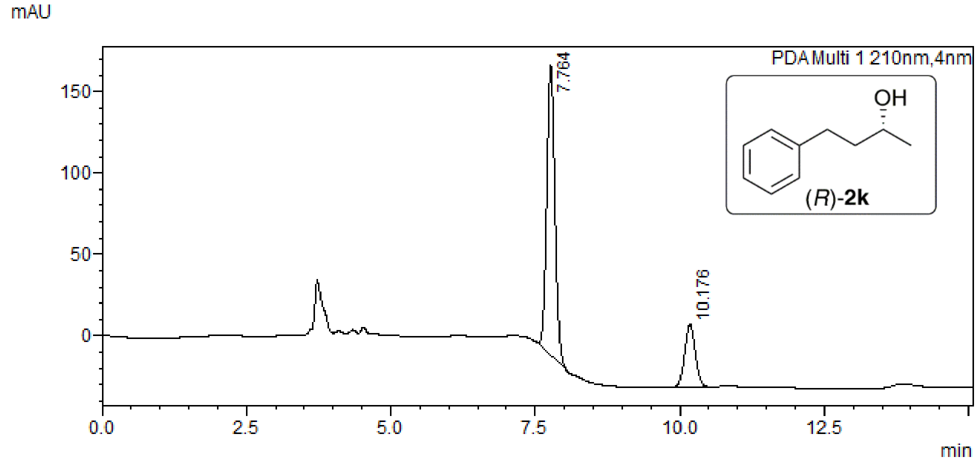

Peak Table

PDA Ch1 210nm

Peak#	Ret. Time	Area	Height	Height%	Area%
1	7.764	1773069	177841	82.112	77.538
2	10.176	513642	38742	17.888	22.462
Total		2286711	216583	100.000	100.000

Supplementary Figure 170. HPLC of *rac*-2l on Chiralcel OD-H at 30 °C

Conditions: *n*-hexane-2-PrOH (95:5, v/v); f=0.9 mL/min; λ=210 nm; *p*=4.1 MPa

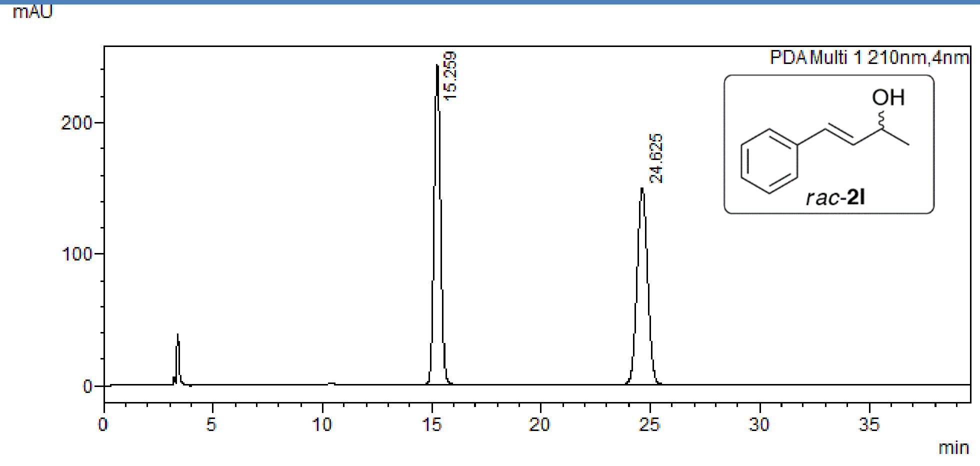

Peak Table

PDA Ch1 210nm

Peak#	Ret. Time	Area	Height	Height%	Area%
1	15.259	5050565	242793	61.887	50.013
2	24.625	5048006	149522	38.113	49.987
Total		10098571	392315	100.000	100.000

Supplementary Figure 171. HPLC of (*R*)-2l on Chiralcel OD-H at 30 °C

Conditions: *n*-hexane-2-PrOH (95:5, v/v); f=0.9 mL/min; λ=210 nm; *p*=4.1 MPa

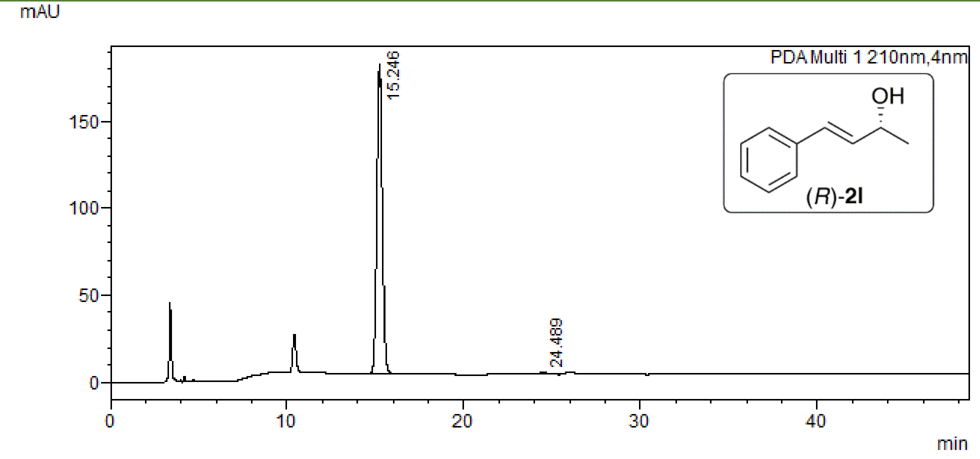

Peak Table

PDA Ch1 210nm

Peak#	Ret. Time	Area	Height	Height%	Area%
1	15.246	3806956	177932	99.393	99.111
2	24.489	34149	1087	0.607	0.889
Total		3841104	179019	100.000	100.000

Supplementary Figure 172. HPLC of *rac*-2m on Chiralcel OD-H at 30 °C

Conditions: *n*-hexane-2-PrOH (98:2, v/v); f=1.0 mL/min; λ=220 nm; *p*=4.4 MPa

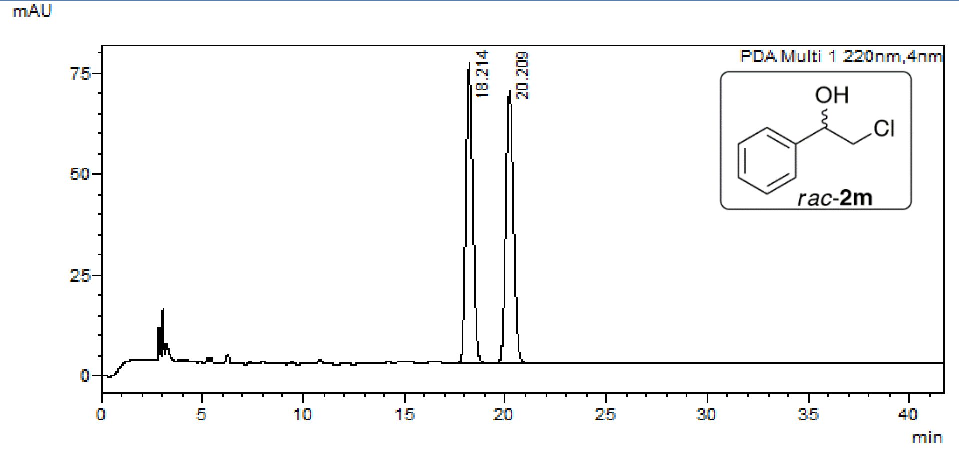

Peak Table

PDA Ch1 220nm

Peak#	Ret. Time	Area	Height	Height%	Area%
1	18.214	1778096	74350	52.465	49.944
2	20.209	1782112	67364	47.535	50.056
Total		3560208	141713	100.000	100.000

Supplementary Figure 173. HPLC of (*S*)-2m on Chiralcel OD-H at 30 °C

Conditions: *n*-hexane-2-PrOH (98:2, v/v); f=1.0 mL/min; λ=220 nm; *p*=4.4 MPa

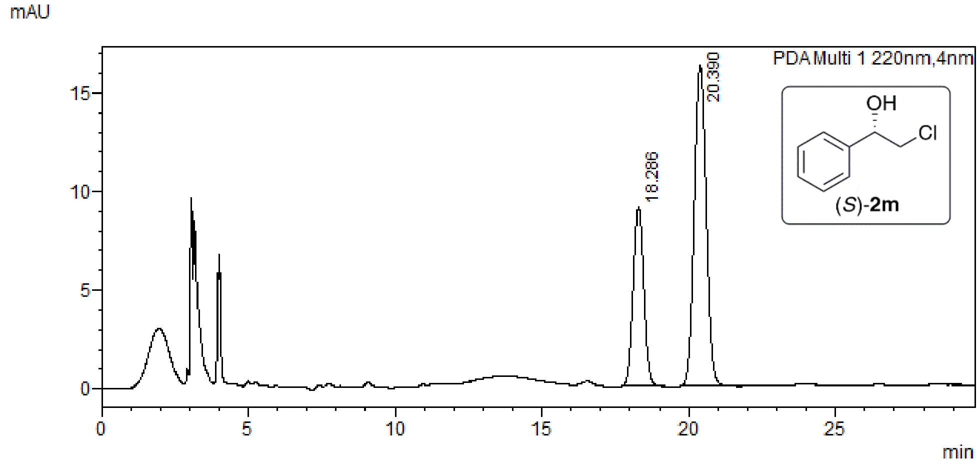

Peak Table

PDA Ch1 220nm

Peak#	Ret. Time	Area	Height	Height%	Area%
1	18.286	224132	9021	35.788	33.242
2	20.390	450117	16186	64.212	66.758
Total		674249	25208	100.000	100.000

Supplementary Figure 174. HPLC of *rac*-2n on Chiralcel OD-H at 30 °C

Conditions: *n*-hexane-2-PrOH (95:5, v/v); f=0.9 mL/min; λ=210 nm; *p*=4.1 MPa

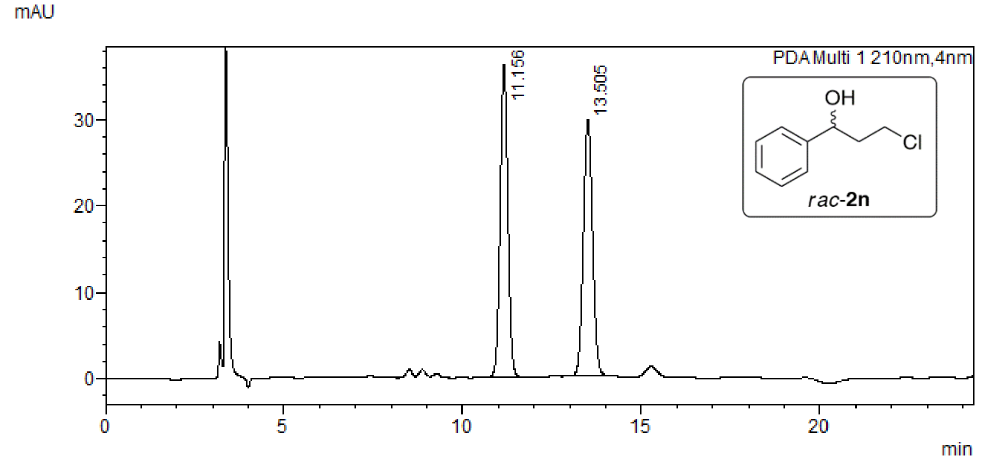

Peak Table

PDA Ch1 210nm

Peak#	Ret. Time	Area	Height	Height%	Area%
1	11.156	549539	36101	54.920	50.552
2	13.505	537536	29633	45.080	49.448
Total		1087075	65734	100.000	100.000

Supplementary Figure 175. HPLC of (*R*)-2n on Chiralcel OD-H at 30 °C

Conditions: *n*-hexane-2-PrOH (95:5, v/v); f=0.9 mL/min; λ=210 nm; *p*=4.1 MPa

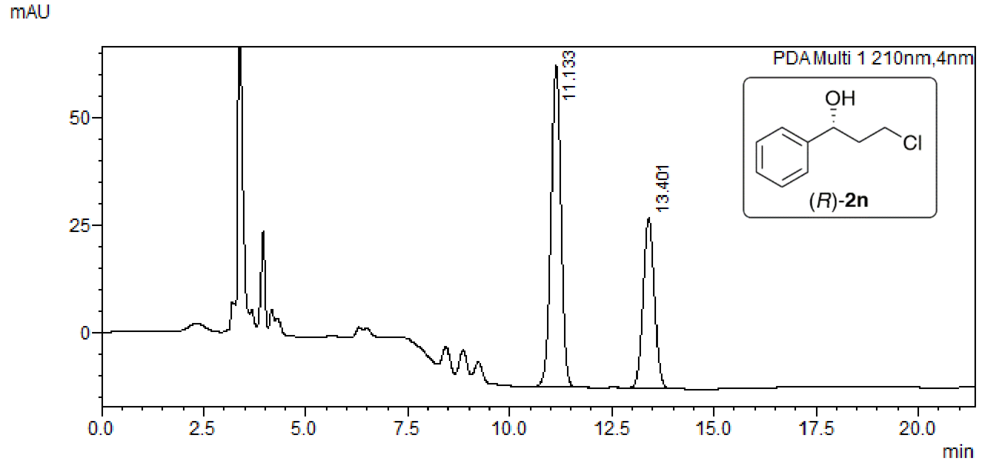

Peak Table

PDA Ch1 210nm

Peak#	Ret. Time	Area	Height	Height%	Area%
1	11.133	1273808	74850	65.389	62.623
2	13.401	760293	39619	34.611	37.377
Total		2034102	114469	100.000	100.000

Supplementary Figure 176. HPLC of *rac*-2o on Chiralcel OD-H at 30 °C

Conditions: *n*-hexane-2-PrOH (98:2, v/v); f=1.0 mL/min; λ=210 nm; *p*=4.4 MPa

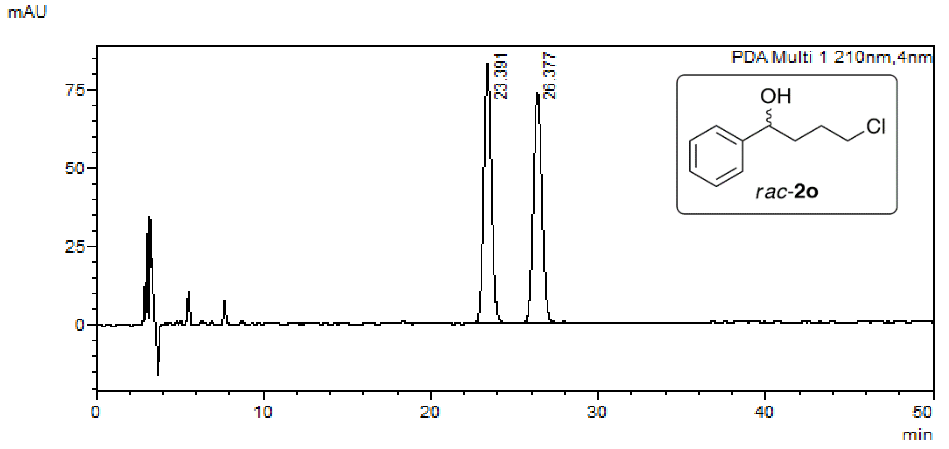

Peak Table

PDA Ch1 210nm

Peak#	Ret. Time	Area	Height	Height%	Area%
1	23.391	2655892	82395	52.952	49.865
2	26.377	2670284	73208	47.048	50.135
Total		5326176	155603	100.000	100.000

Supplementary Figure 177. HPLC of (*R*)-2o on Chiralcel OD-H at 30 °C

Conditions: *n*-hexane-2-PrOH (98:2, v/v); f=1.0 mL/min; λ=210 nm; *p*=4.4 MPa

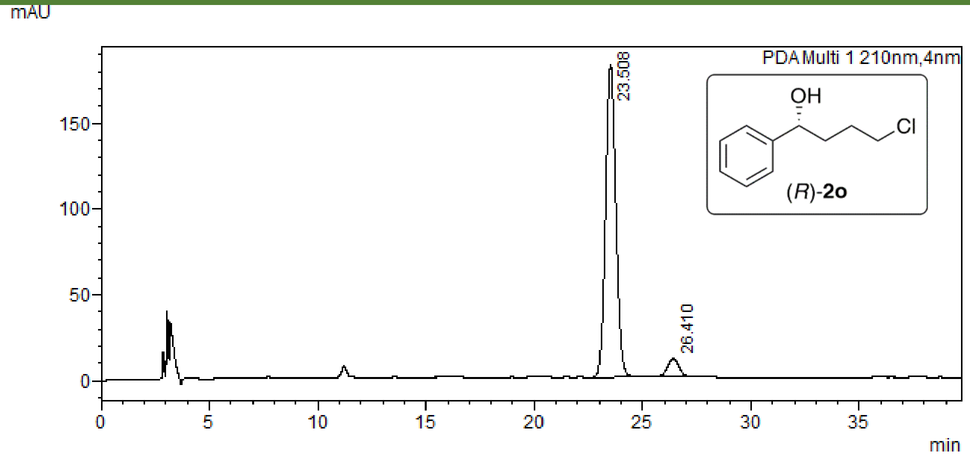

Peak Table

PDA Ch1 210nm

Peak#	Ret. Time	Area	Height	Height%	Area%
1	23.508	6019973	182018	94.549	93.971
2	26.410	386212	10493	5.451	6.029
Total		6406185	192512	100.000	100.000

Supplementary Figure 178. HPLC of *rac*-2p on Chiralcel OJ-H at 30 °C

Conditions: *n*-hexane-2-PrOH (95:5, v/v); f=0.8 mL/min; λ=220 nm; *p*=3.4 MPa

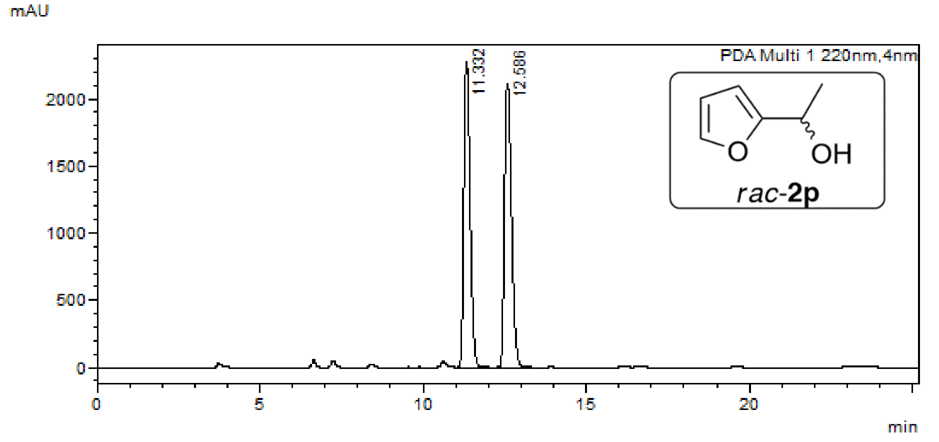

Peak Table

PDA Ch1 220nm

Peak#	Ret. Time	Area	Height	Height%	Area%
1	11.332	30914198	2268863	51.791	49.435
2	12.586	31620869	2111971	48.209	50.565
Total		62535067	4380834	100.000	100.000

Supplementary Figure 179. HPLC of commercial (*S*)-1-(2-furyl)ethanol [(*S*)-2p] (>99% ee) on Chiralcel OJ-H at 30 °C

Conditions: *n*-hexane-2-PrOH (95:5, v/v); f=0.8 mL/min; λ=220 nm; *p*=3.4 MPa

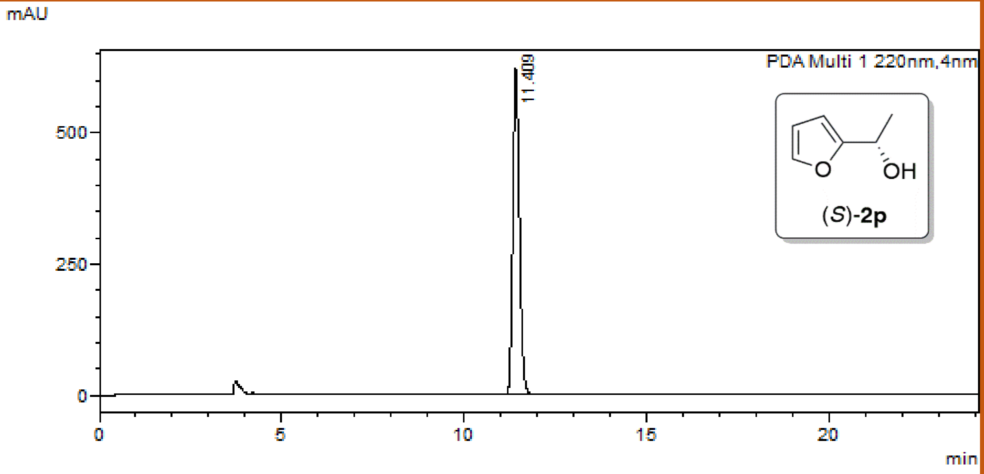

Peak Table

PDA Ch1 220nm

Peak#	Ret. Time	Area	Height	Height%	Area%
1	11.409	7876555	620294	100.000	100.000
Total		7876555	620294	100.000	100.000

Supplementary Figure 180. HPLC of (*R*)-2p on Chiralcel OJ-H at 30 °C

Conditions: *n*-hexane-2-PrOH (95:5, v/v); f=0.8 mL/min; λ=220 nm; *p*=3.4 MPa

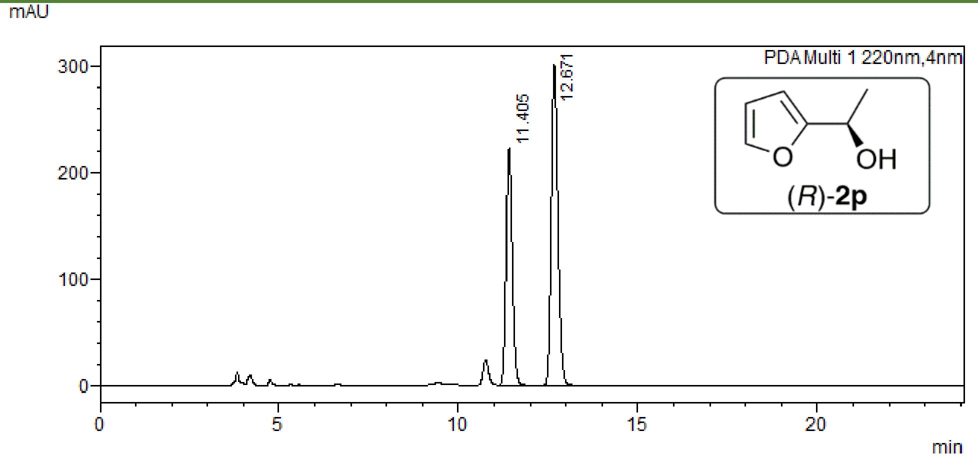

Peak Table

PDA Ch1 220nm

Peak#	Ret. Time	Area	Height	Height%	Area%
1	11.405	2588105	221723	42.449	39.840
2	12.671	3908111	300610	57.551	60.160
Total		6496216	522333	100.000	100.000

Supplementary Figure 181. HPLC of *rac*-2q on Chiralcel OJ-H at 30 °C

Conditions: *n*-hexane-2-PrOH (95:5, v/v); f=0.8 mL/min; λ=233 nm; *p*=3.4 MPa

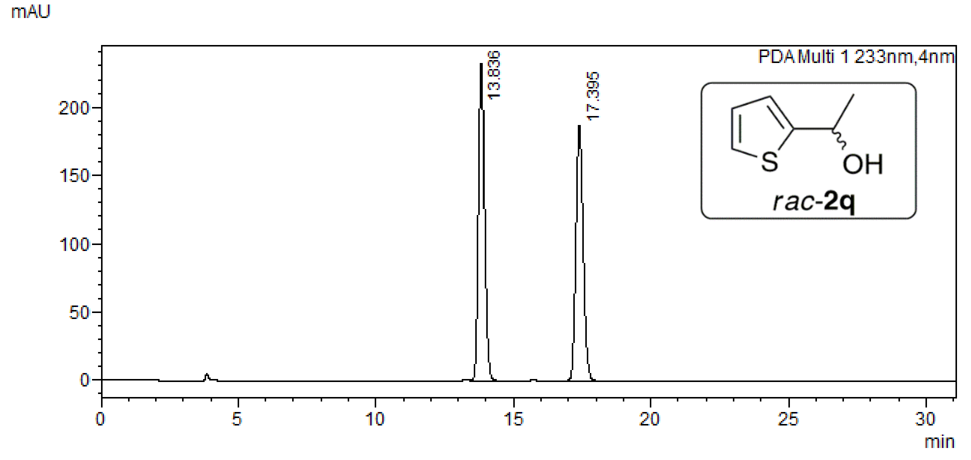

Peak Table

PDA Ch1 233nm

Peak#	Ret. Time	Area	Height	Height%	Area%
1	13.836	3455203	231777	55.367	50.052
2	17.395	3448073	186844	44.633	49.948
Total		6903277	418621	100.000	100.000

Supplementary Figure 182. HPLC of *rac*-2q (after enzymatic reaction) on Chiralcel OJ-H at 30 °C

Conditions: *n*-hexane-2-PrOH (95:5, v/v); f=0.8 mL/min; λ=233 nm; *p*=3.4 MPa

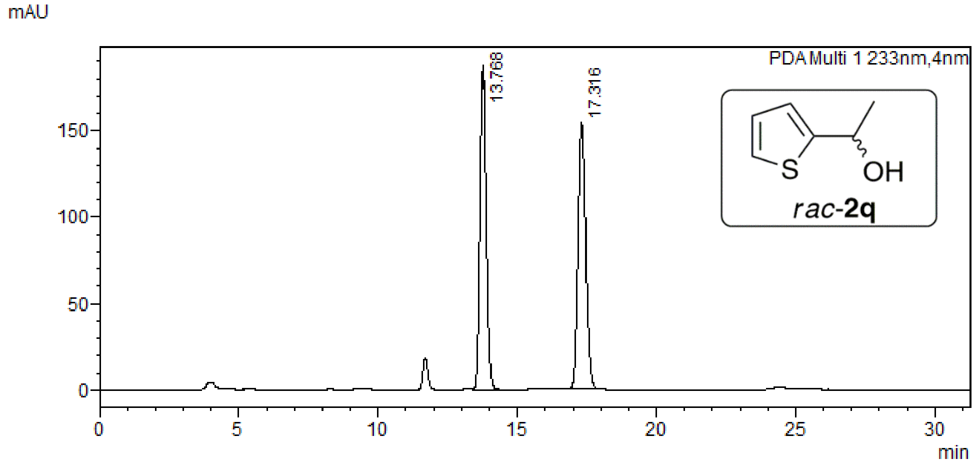

Peak Table

PDA Ch1 233nm

Peak#	Ret. Time	Area	Height	Height%	Area%
1	13.768	2960197	187308	54.892	50.120
2	17.316	2946013	153923	45.108	49.880
Total		5906210	341231	100.000	100.000

Supplementary Figure 183. HPLC of *rac*-2r on Lux i-Cellulose 5 at 30 °C

Conditions: *n*-hexane-2-PrOH (99:1, v/v); f=1.0 mL/min; λ=258 nm; *p*=3.7 MPa

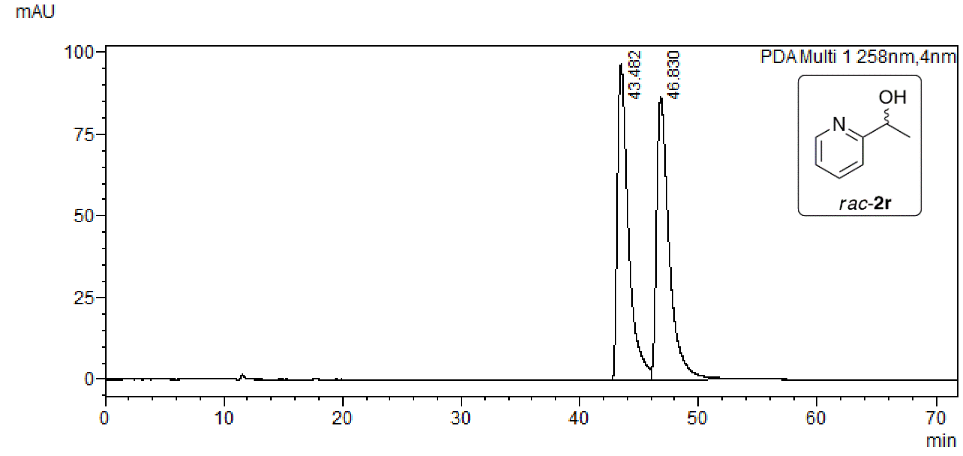

Peak Table

PDA Ch1 258nm

Peak#	Ret. Time	Area	Height	Height%	Area%
1	43.482	6251105	96475	52.741	49.255
2	46.830	6440333	86449	47.259	50.745
Total		12691438	182924	100.000	100.000

Supplementary Figure 184. HPLC of (*R*)-2r on Lux i-Cellulose 5 at 30 °C

Conditions: *n*-hexane-2-PrOH (99:1, v/v); f=1.0 mL/min; λ=258 nm; *p*=3.7 MPa

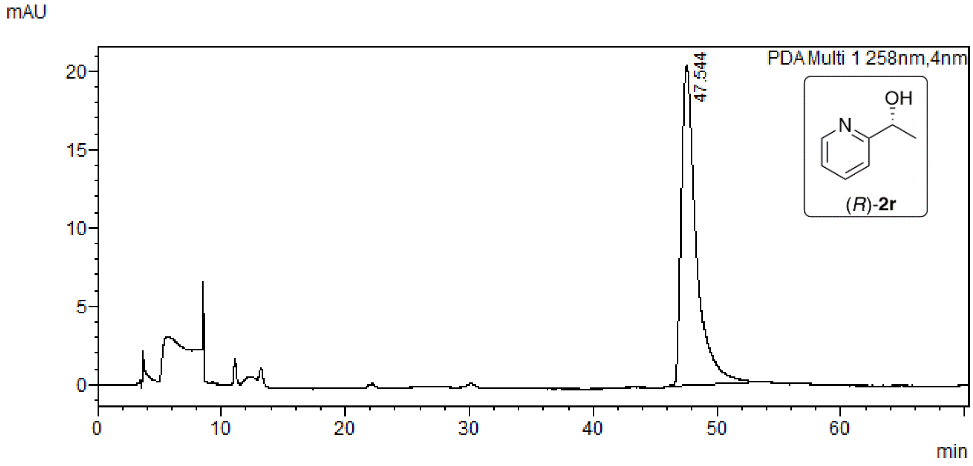

Peak Table

PDA Ch1 258nm

Peak#	Ret. Time	Area	Height	Height%	Area%
1	47.544	1730960	20440	100.000	100.000
Total		1730960	20440	100.000	100.000

Supplementary Figure 185. HPLC of *rac*-2s on Chiralcel OD-H at 30 °C

Conditions: *n*-hexane-2-PrOH (95:5, v/v); f=0.7 mL/min; λ=225 nm; *p*=3.1 MPa

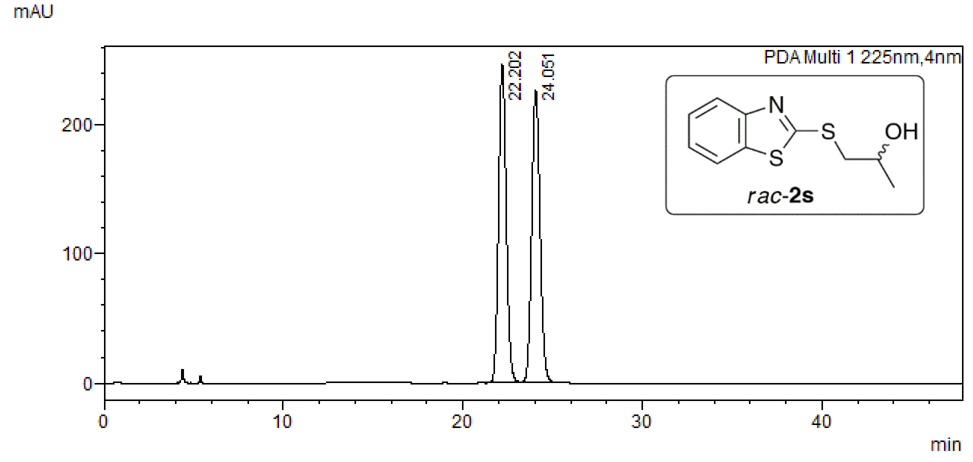

Peak Table

PDA Ch1 225nm

Peak#	Ret. Time	Area	Height	Height%	Area%
1	22.202	7364392	246354	52.069	49.894
2	24.051	7395826	226772	47.931	50.106
Total		14760218	473127	100.000	100.000

Supplementary Figure 186. HPLC of (*R*)-2s on Chiralcel OD-H at 30 °C

Conditions: *n*-hexane-2-PrOH (95:5, v/v); f=0.7 mL/min; λ=225 nm; *p*=3.1 MPa

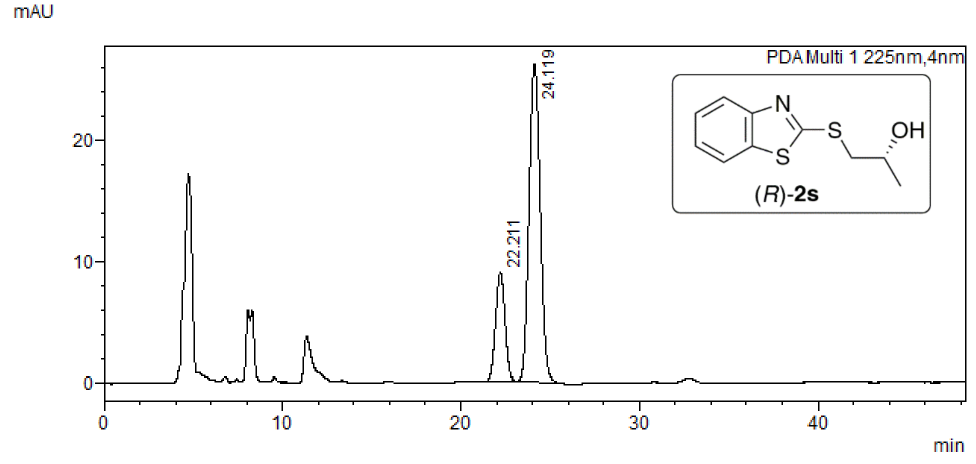

Peak Table

PDA Ch1 225nm

Peak#	Ret. Time	Area	Height	Height%	Area%
1	22.211	330578	8950	25.541	22.427
2	24.119	1143437	26090	74.459	77.573
Total		1474015	35040	100.000	100.000

Supplementary Figure 187. HPLC of *rac*-2t on Chiralcel OD-H at 30 °C

Conditions: *n*-hexane-2-PrOH (90:10, v/v); f=0.8 mL/min; λ=254 nm; *p*=3.7 MPa

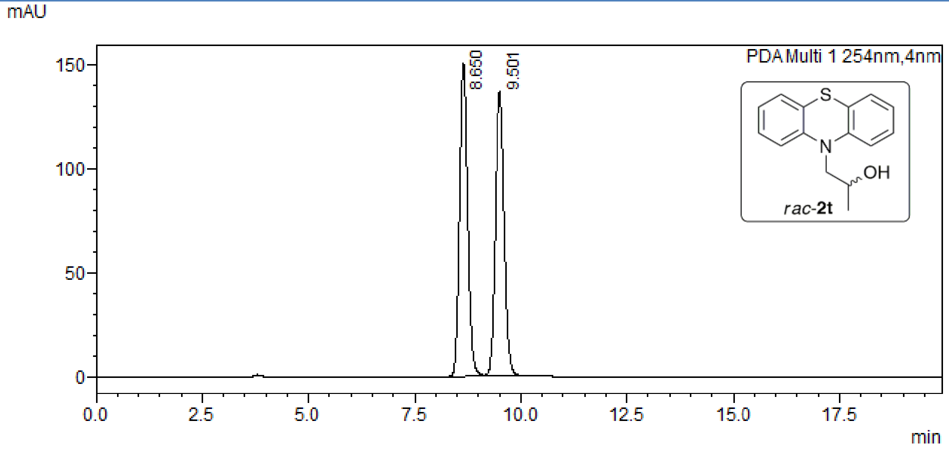

Peak Table

PDA Ch1 254nm

Peak#	Ret. Time	Area	Height	Height%	Area%
1	8.650	1985948	150560	52.403	49.929
2	9.501	1991633	136754	47.597	50.071
Total		3977581	287314	100.000	100.000

Supplementary Figure 188. HPLC of *rac*-2u on Chiralcel OD-H at 30 °C

Conditions: *n*-hexane-2-PrOH (90:10, v/v); f=0.8 mL/min; λ=216 nm; *p*=3.7 MPa

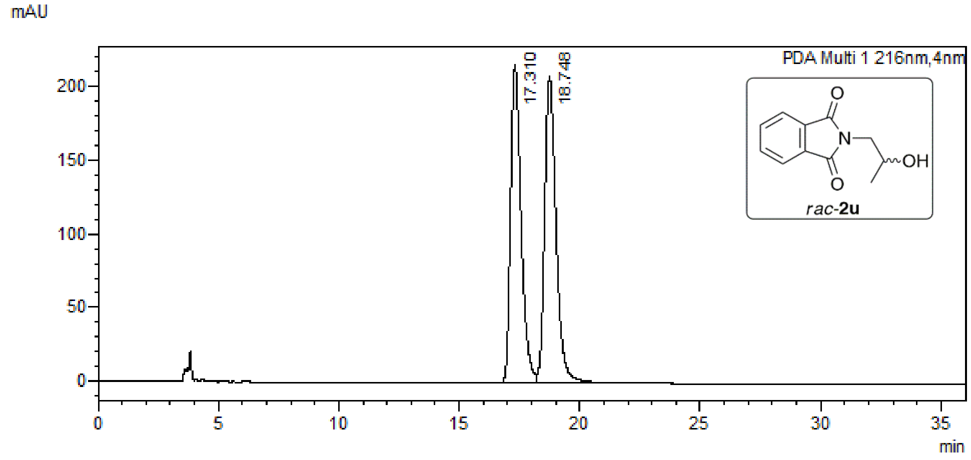

Peak Table

PDA Ch1 216nm

Peak#	Ret. Time	Area	Height	Height%	Area%
1	17.310	6533958	215507	50.890	49.096
2	18.748	6774482	207972	49.110	50.904
Total		13308440	423479	100.000	100.000

Supplementary Figure 189. HPLC of (*R*)-2u on Chiralcel OD-H at 30 °C

Conditions: *n*-hexane-2-PrOH (90:10, v/v); f=0.8 mL/min; λ=216 nm; *p*=3.7 MPa

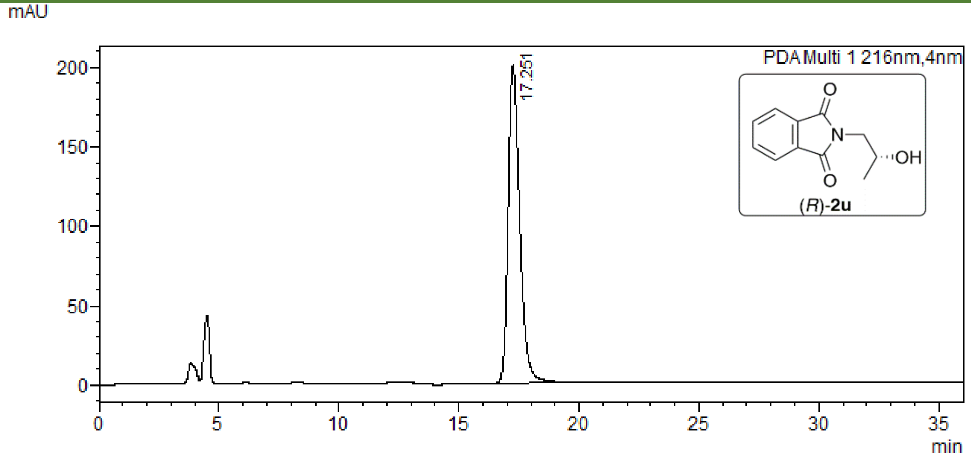

Peak Table

PDA Ch1 216nm

Peak#	Ret. Time	Area	Height	Height%	Area%
1	17.251	6764745	200300	100.000	100.000
Total		6764745	200300	100.000	100.000

Supplementary Figure 190. HPLC of *rac*-2v on Chiralpak AD-H at 25 °C

Conditions: *n*-hexane-2-PrOH (78:22, v/v); f=1.0 mL/min; λ=273 nm; *p*=5.5 MPa

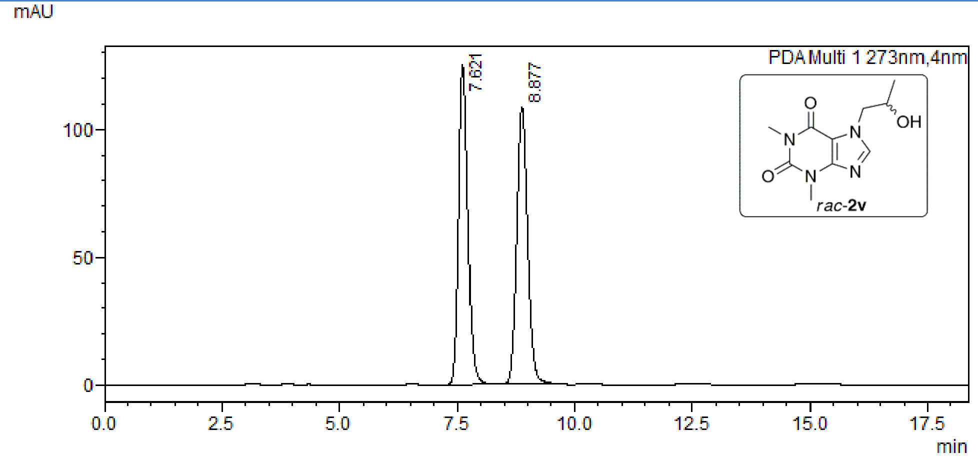

Peak Table

PDA Ch1 273nm

Peak#	Ret. Time	Area	Height	Height%	Area%
1	7.621	1722456	125291	53.541	49.400
2	8.877	1764320	108716	46.459	50.600
Total		3486776	234008	100.000	100.000

Supplementary Figure 191. HPLC of (*R*)-2v on Chiralpak AD-H at 25 °C

Conditions: *n*-hexane-2-PrOH (78:22, v/v); f=1.0 mL/min; λ=273 nm; *p*=5.5 MPa

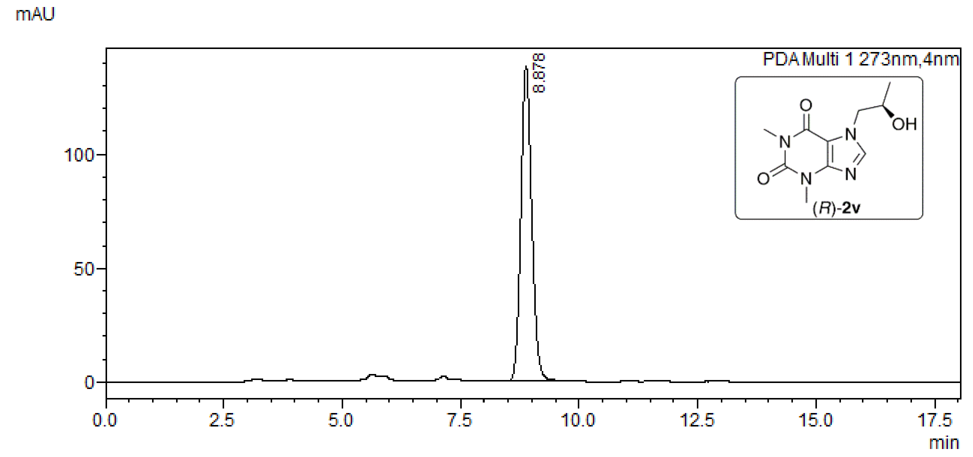

Peak Table

PDA Ch1 273nm

Peak#	Ret. Time	Area	Height	Height%	Area%
1	8.878	2217073	138194	100.000	100.000
Total		2217073	138194	100.000	100.000

Supplementary Figure 192. HPLC of *rac*-2w on Chiralcel OJ-H at 30 °C

Conditions: *n*-hexane-2-PrOH (90:10, v/v); f=1.0 mL/min; λ=264 nm; *p*=4.5 MPa

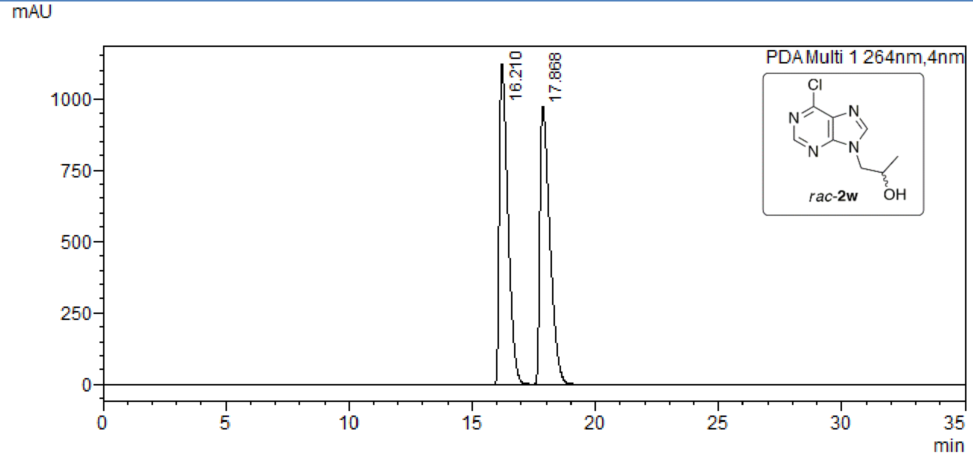

Peak Table

PDA Ch1 264nm

Peak#	Ret. Time	Area	Height	Height%	Area%
1	16.210	27661596	1119207	53.492	49.959
2	17.868	27706959	973091	46.508	50.041
Total		55368556	2092298	100.000	100.000

Supplementary Figure 193. HPLC of (*R*)-2w on Chiralcel OJ-H at 30 °C

Conditions: *n*-hexane-2-PrOH (90:10, v/v); f=1.0 mL/min; λ=264 nm; *p*=4.5 MPa

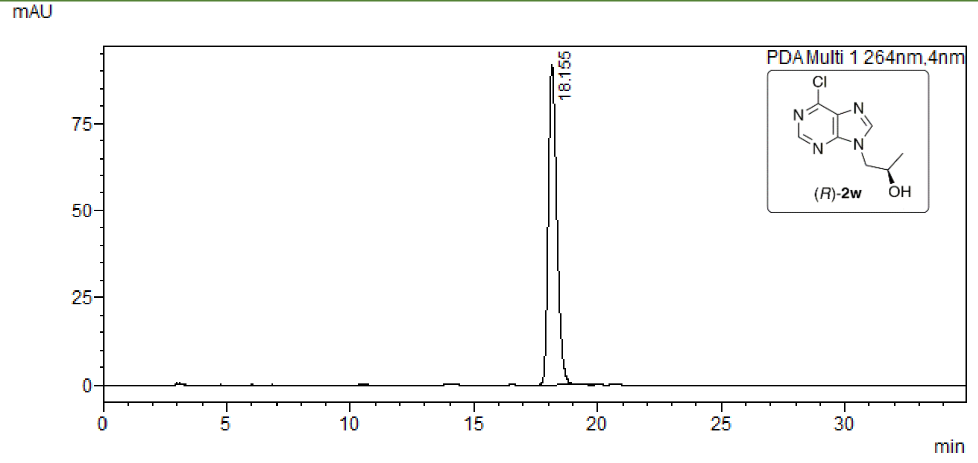

Peak Table

PDA Ch1 264nm

Peak#	Ret. Time	Area	Height	Height%	Area%
1	18.155	2222316	91630	100.000	100.000
Total		2222316	91630	100.000	100.000

Supplementary Figure 194. HPLC of *rac*-2x on Chiralcel OD-H at 30 °C

Conditions: *n*-hexane-2-PrOH (90:10, v/v); f=0.8 mL/min; λ=220 nm; *p*=3.7 MPa

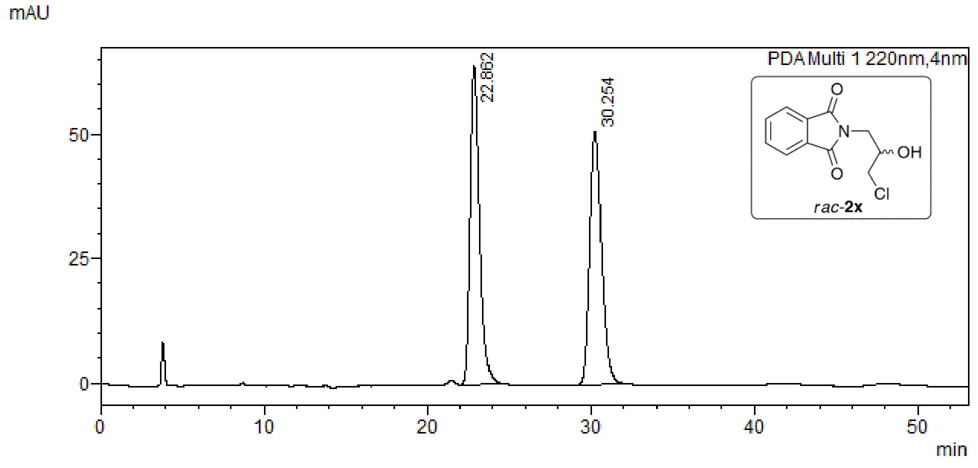

Peak Table

PDA Ch1 220nm

Peak#	Ret. Time	Area	Height	Height%	Area%
1	22.862	2596038	63889	55.734	50.349
2	30.254	2560010	50744	44.266	49.651
Total		5156047	114633	100.000	100.000

Supplementary Figure 195. HPLC of (*S*)-2x on Chiralcel OD-H at 30 °C

Conditions: *n*-hexane-2-PrOH (90:10, v/v); f=0.8 mL/min; λ=220 nm; *p*=3.7 MPa

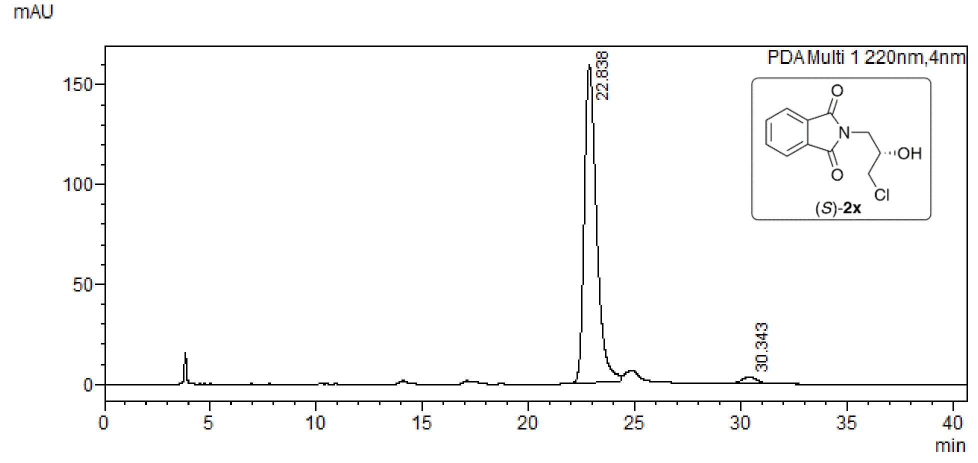

Peak Table

PDA Ch1 220nm

Peak#	Ret. Time	Area	Height	Height%	Area%
1	22.838	6492723	159406	97.974	97.677
2	30.343	154386	3296	2.026	2.323
Total		6647109	162702	100.000	100.000

Supplementary Figure 196. HPLC of *rac*-2y on Chiralpak AD-H at 25 °C

Conditions: *n*-hexane-2-PrOH (78:22, v/v); f=0.3 mL/min; λ=273 nm; *p*=1.6 MPa

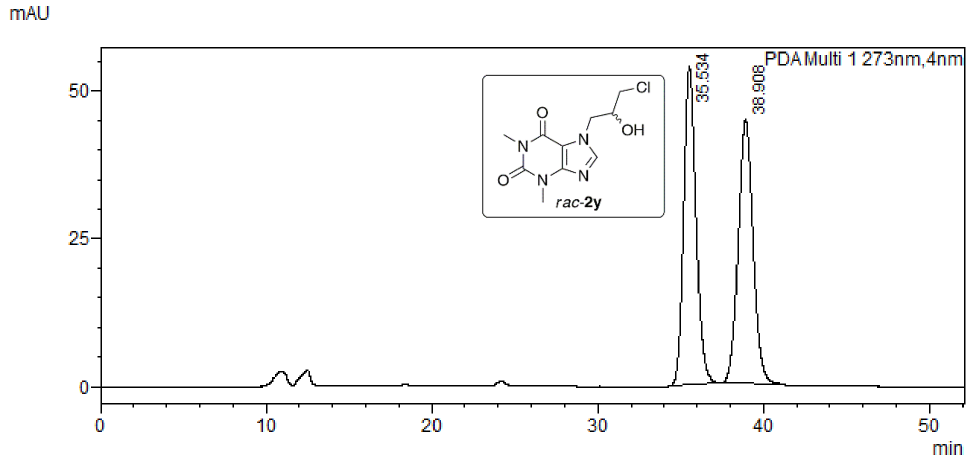

Peak Table

PDA Ch1 273nm

Peak#	Ret. Time	Area	Height	Height%	Area%
1	35.534	2788823	53856	54.680	50.025
2	38.908	2786037	44636	45.320	49.975
Total		5574860	98492	100.000	100.000

Supplementary Figure 197. HPLC of (*S*)-2y on Chiralpak AD-H at 25 °C

Conditions: *n*-hexane-2-PrOH (78:22, v/v); f=0.3 mL/min; λ=273 nm; *p*=1.6 MPa

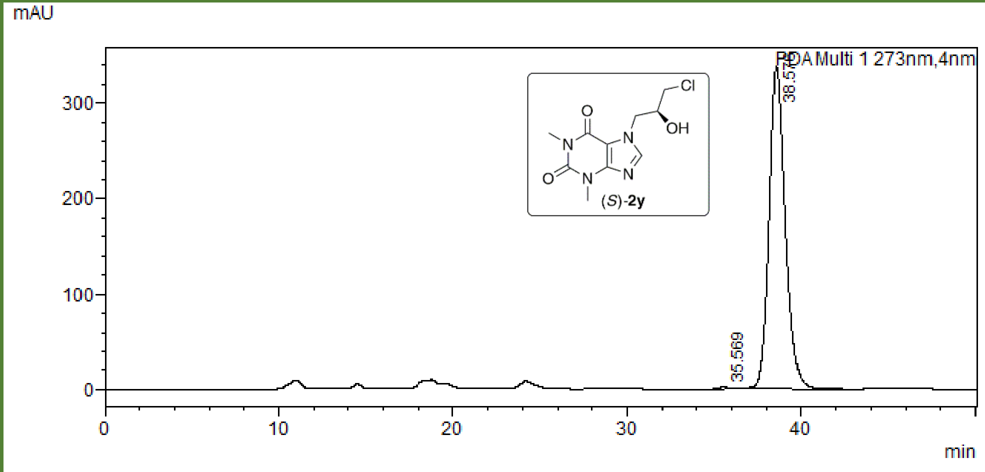

Peak Table

PDA Ch1 273nm

Peak#	Ret. Time	Area	Height	Height%	Area%
1	35.569	78515	1783	0.525	0.360
2	38.576	21744118	338208	99.475	99.640
Total		21822633	339991	100.000	100.000

Supplementary Figure 198. HPLC of *rac*-2z on Chiralcel OD-H at 30 °C

Conditions: *n*-hexane-2-PrOH (90:10, v/v); f=0.8 mL/min; λ=225 nm; *p*=3.7 MPa

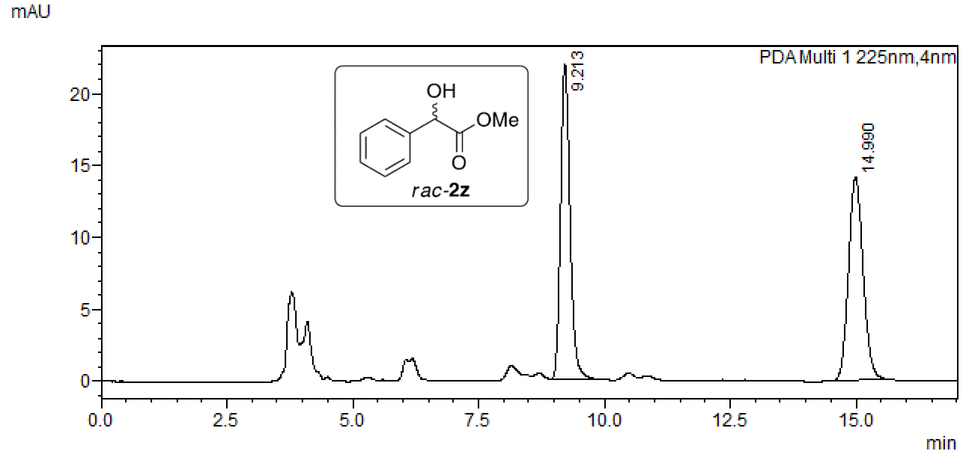

Peak Table

PDA Ch1 225nm

Peak#	Ret. Time	Area	Height	Height%	Area%
1	9.213	285333	21864	60.834	49.986
2	14.990	285492	14076	39.166	50.014
Total		570825	35940	100.000	100.000

Supplementary Figure 199. HPLC of (*S*)-2z on Chiralcel OD-H at 30 °C

Conditions: *n*-hexane-2-PrOH (90:10, v/v); f=0.8 mL/min; λ=225 nm; *p*=3.7 MPa

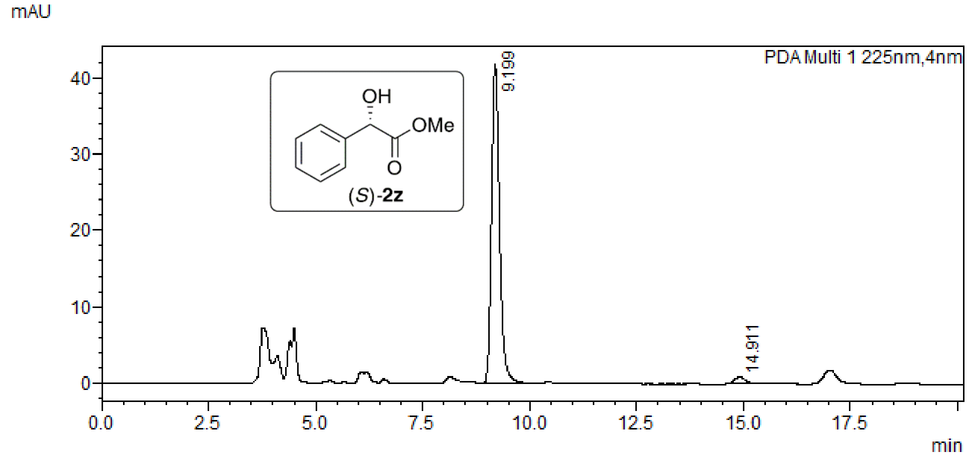

Peak Table

PDA Ch1 225nm

Peak#	Ret. Time	Area	Height	Height%	Area%
1	9.199	545896	41654	98.020	97.355
2	14.911	14834	841	1.980	2.645
Total		560729	42495	100.000	100.000

Supplementary Figure 200. HPLC of *rac*-2aa on Chiralcel OJ-H at 30 °C

Conditions: *n*-hexane-2-PrOH (90:10, v/v); f=0.5 mL/min; λ=210 nm; *p*=2.2 MPa

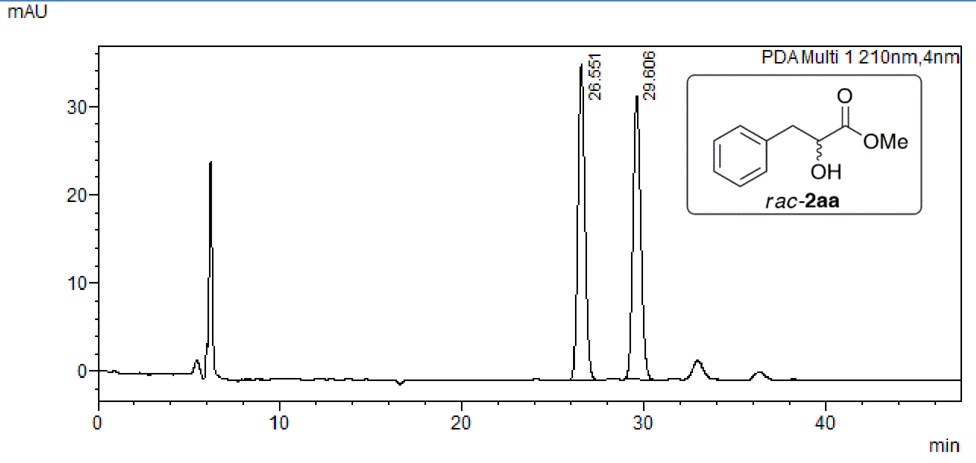

Peak Table

PDA Ch1 210nm

Peak#	Ret. Time	Area	Height	Height%	Area%
1	26.551	964749	35621	52.641	49.922
2	29.606	967764	32047	47.359	50.078
Total		1932513	67667	100.000	100.000

Supplementary Figure 201. HPLC of (*S*)-2aa on Chiralcel OJ-H at 30 °C

Conditions: *n*-hexane-2-PrOH (90:10, v/v); f=0.5 mL/min; λ=210 nm; *p*=2.2 MPa

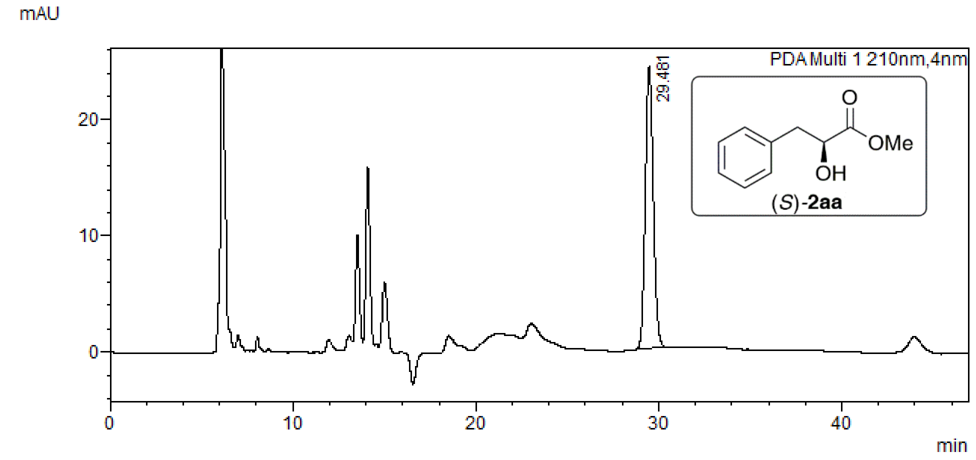

Peak Table

PDA Ch1 210nm

Peak#	Ret. Time	Area	Height	Height%	Area%
1	29.481	737964	24230	100.000	100.000
Total		737964	24230	100.000	100.000

Supplementary Figure 202. HPLC of *rac*-2ab on Chiralcel OD-H at 30 °C

Conditions: *n*-hexane-2-PrOH (90:10, v/v); f=0.8 mL/min; λ=210 nm; *p*=3.7 MPa

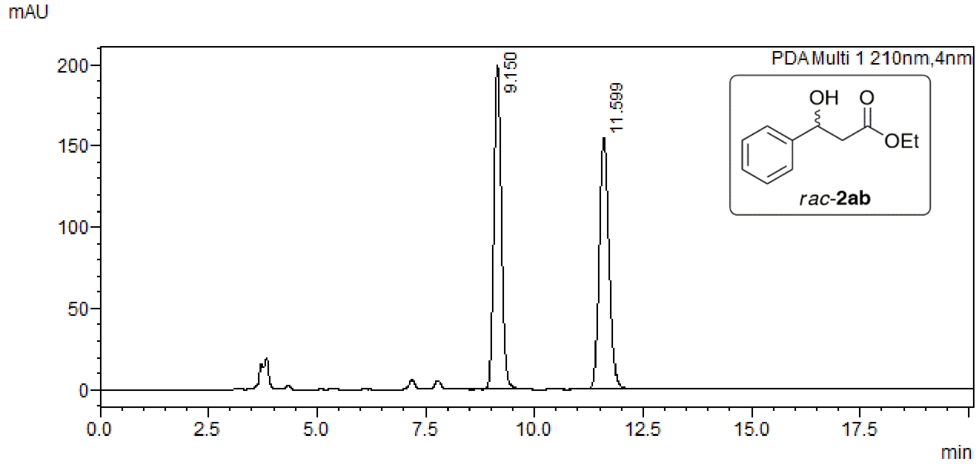

Peak Table

PDA Ch1 210nm

Peak#	Ret. Time	Area	Height	Height%	Area%
1	9.150	2449125	199204	56.251	50.052
2	11.599	2444078	154932	43.749	49.948
Total		4893203	354137	100.000	100.000

Supplementary Figure 203. HPLC of (*R*)-2ab on Chiralcel OD-H at 30 °C

Conditions: *n*-hexane-2-PrOH (90:10, v/v); f=0.8 mL/min; λ=210 nm; *p*=3.7 MPa

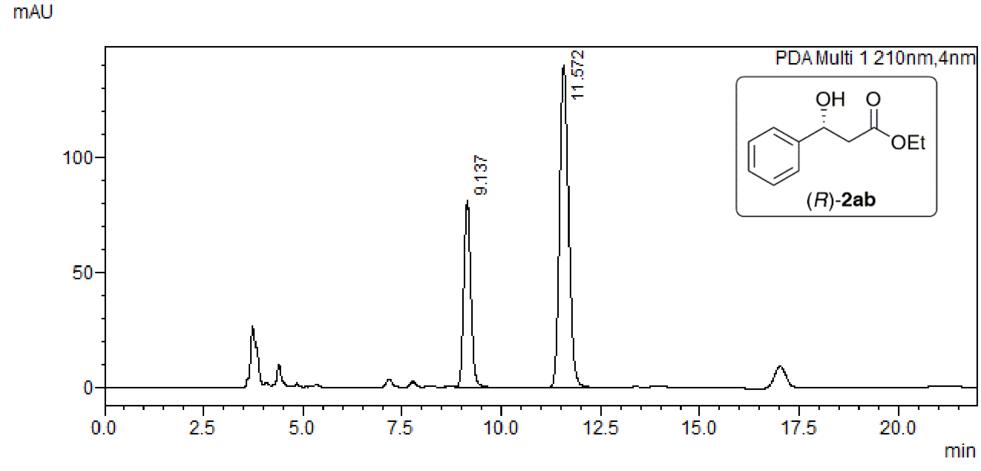

Peak Table

PDA Ch1 210nm

Peak#	Ret. Time	Area	Height	Height%	Area%
1	9.137	1013018	81130	36.705	31.422
2	11.572	2210911	139904	63.295	68.578
Total		3223929	221034	100.000	100.000

Supplementary Figure 204. HPLC of *rac*-2ac on Chiralcel OD-H at 30 °C

Conditions: *n*-hexane-2-PrOH (98:2, v/v); f=1.0 mL/min; λ=210 nm; *p*=4.4 MPa

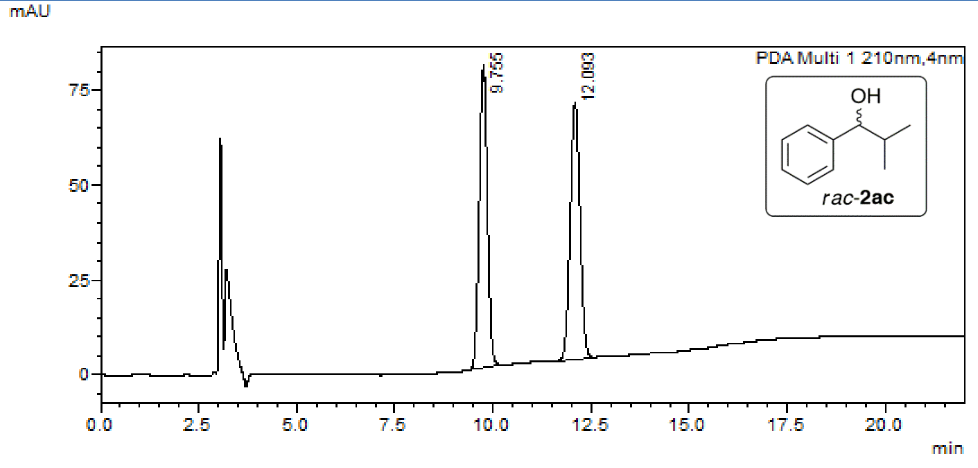

Peak Table

PDA Ch1 210nm

Peak#	Ret. Time	Area	Height	Height%	Area%
1	9.755	1160716	79616	54.029	49.906
2	12.093	1165089	67741	45.971	50.094
Total		2325805	147357	100.000	100.000

Supplementary Figure 205. HPLC of (*R*)-2ac on Chiralcel OD-H at 30 °C

Conditions: *n*-hexane-2-PrOH (98:2, v/v); f=1.0 mL/min; λ=210 nm; *p*=4.4 MPa

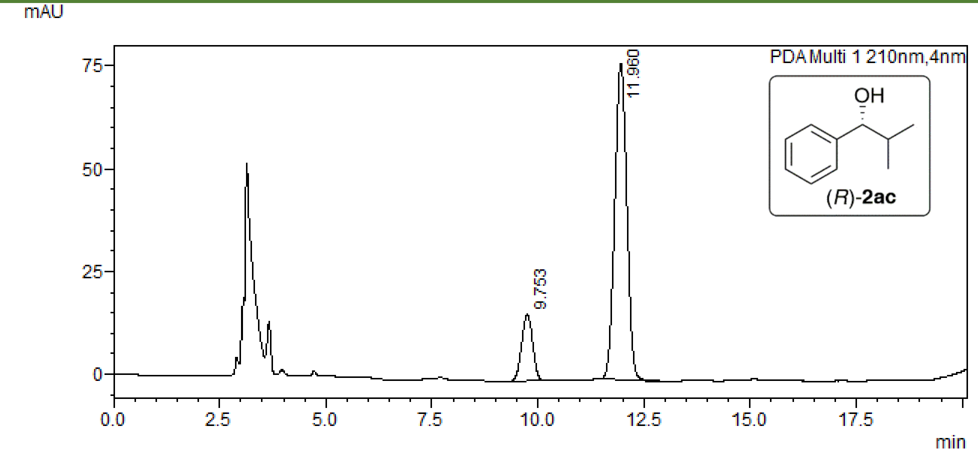

Peak Table

PDA Ch1 210nm

Peak#	Ret. Time	Area	Height	Height%	Area%
1	9.753	290439	16121	17.346	16.064
2	11.960	1517608	76817	82.654	83.936
Total		1808048	92938	100.000	100.000

Supplementary Figure 206. HPLC of *rac*-2ad on Chiralcel OJ-H at 30 °C

Conditions: *n*-hexane-2-PrOH (98:2, v/v); f=1.0 mL/min; λ=204 nm; *p*=4.3 MPa

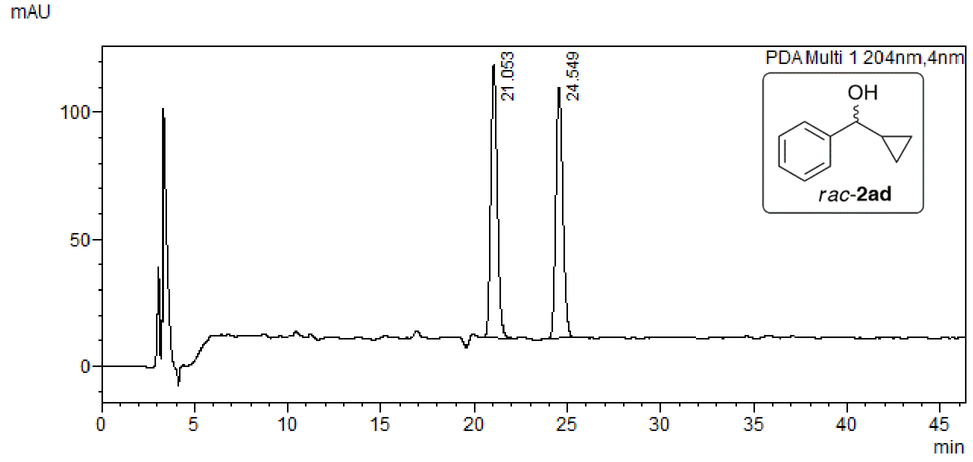

Peak Table

PDA Ch1 204nm

Peak#	Ret. Time	Area	Height	Height%	Area%
1	21.053	2643789	107438	52.079	50.063
2	24.549	2637183	98858	47.921	49.937
Total		5280972	206296	100.000	100.000

Supplementary Figure 207. HPLC of (*R*)-2ad on Chiralcel OJ-H at 30 °C

Conditions: *n*-hexane-2-PrOH (98:2, v/v); f=1.0 mL/min; λ=204 nm; *p*=4.3 MPa

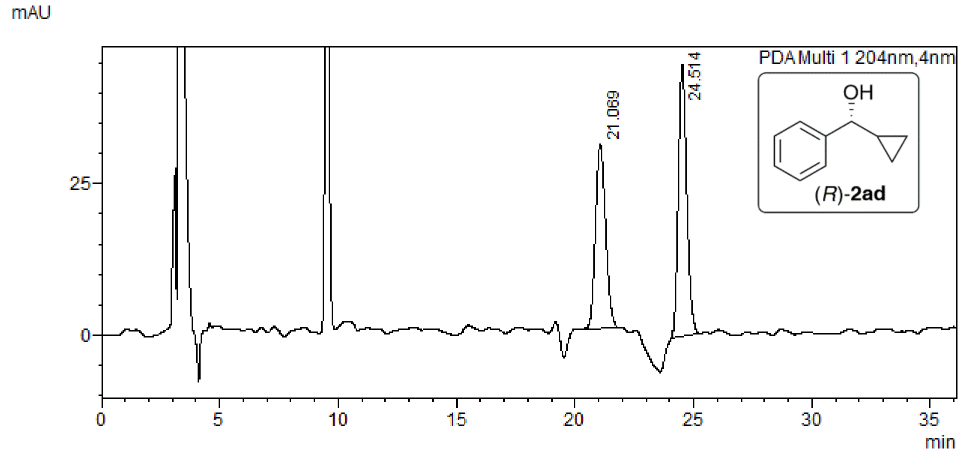

Peak Table

PDA Ch1 204nm

Peak#	Ret. Time	Area	Height	Height%	Area%
1	21.069	910443	30337	40.387	45.086
2	24.514	1108903	44778	59.613	54.914
Total		2019346	75115	100.000	100.000

Supplementary Figure 208. HPLC of *rac*-2ae on Chiralcel OD-H at 30 °C

Conditions: *n*-hexane-2-PrOH (95:5, v/v); f=0.9 mL/min; λ=210 nm; *p*=4.1 MPa

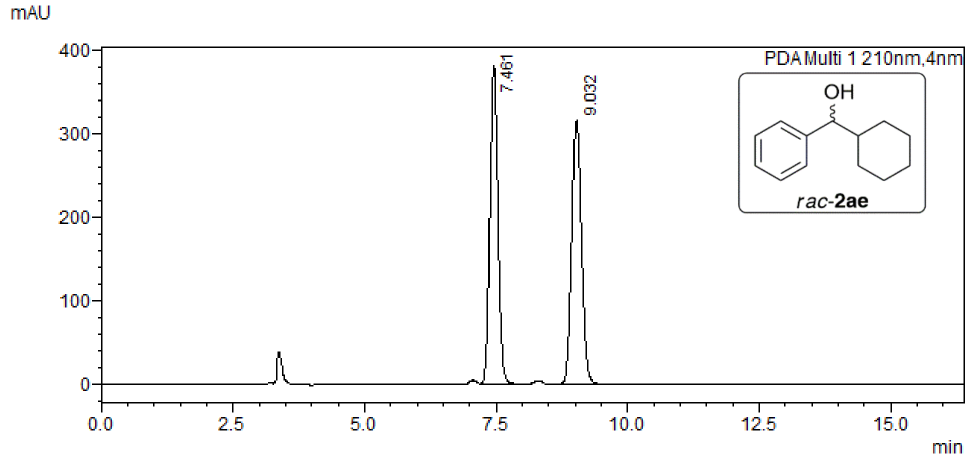

Peak Table

PDA Ch1 210nm

Peak#	Ret. Time	Area	Height	Height%	Area%
1	7.461	4062568	381787	54.711	50.072
2	9.032	4050923	316039	45.289	49.928
Total		8113491	697826	100.000	100.000

Supplementary Figure 209. HPLC of *rac*-2af on Chiralcel OD-H at 30 °C

Conditions: *n*-hexane-2-PrOH (95:5, v/v); f=0.9 mL/min; λ=210 nm; *p*=4.1 MPa

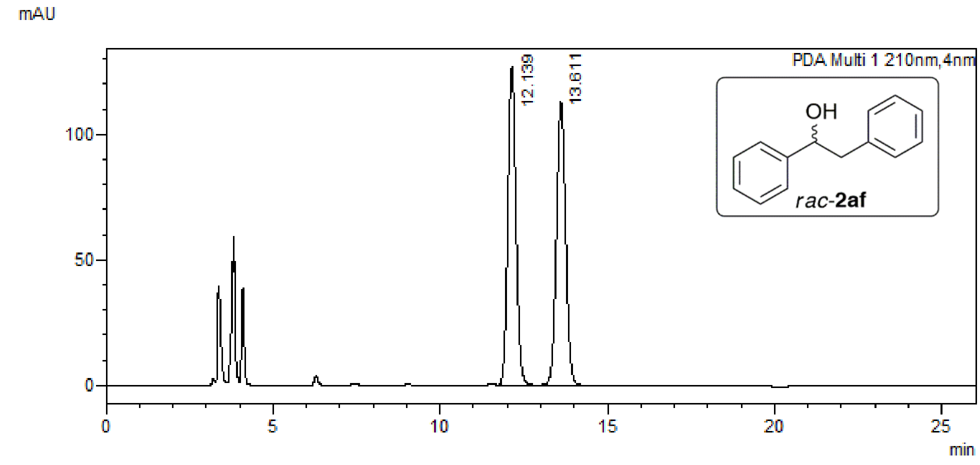

Peak Table

PDA Ch1 210nm

Peak#	Ret. Time	Area	Height	Height%	Area%
1	12.139	2150967	126525	52.859	49.963
2	13.611	2154120	112837	47.141	50.037
Total		4305087	239362	100.000	100.000

Supplementary Figure 210. HPLC of (*S*)-2af on Chiralcel OD-H at 30 °C

Conditions: *n*-hexane-2-PrOH (95:5, v/v); f=0.9 mL/min; λ=210 nm; *p*=4.1 MPa

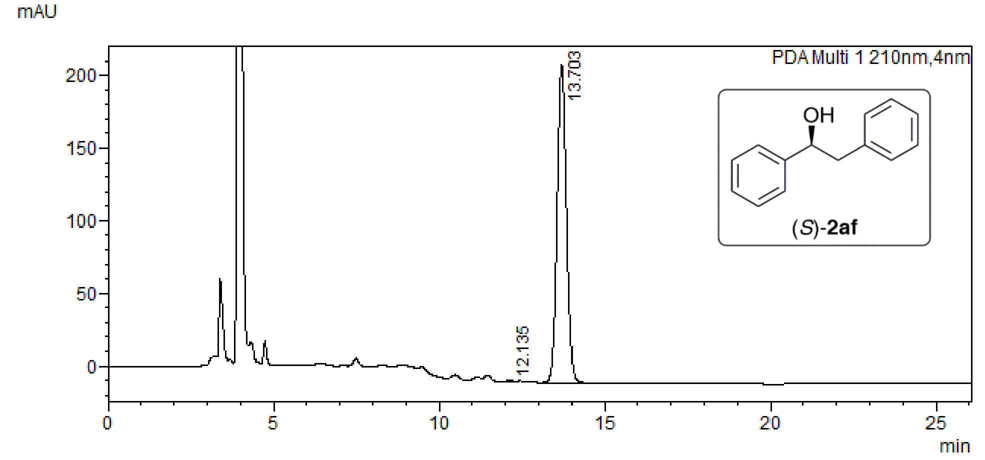

Peak Table

PDA Ch1 210nm

Peak#	Ret. Time	Area	Height	Height%	Area%
1	12.135	6783	518	0.236	0.157
2	13.703	4299775	218744	99.764	99.843
Total		4306558	219262	100.000	100.000

Supplementary Figure 211. HPLC of *rac*-2ag on Chiralcel OJ-H at 30 °C

Conditions: *n*-hexane-2-PrOH (97:3, v/v); f=1.0 mL/min; λ=220 nm; *p*=4.3 MPa

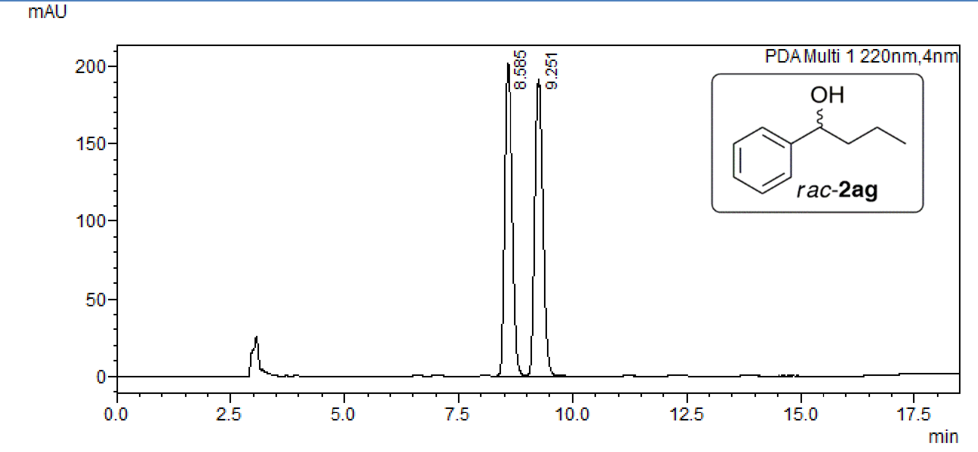

Peak Table

PDA Ch1 220nm

Peak#	Ret. Time	Area	Height	Height%	Area%
1	8.585	2325693	201705	51.359	49.799
2	9.251	2344423	191029	48.641	50.201
Total		4670116	392734	100.000	100.000

Supplementary Figure 212. HPLC of commercial (*R*)-1-phenyl-1-butanol [(*R*)-2ag] (>99% ee) on Chiralcel OJ-H at 30 °C

Conditions: *n*-hexane-2-PrOH (97:3, v/v); f=1.0 mL/min; λ=220 nm; *p*=4.3 MPa

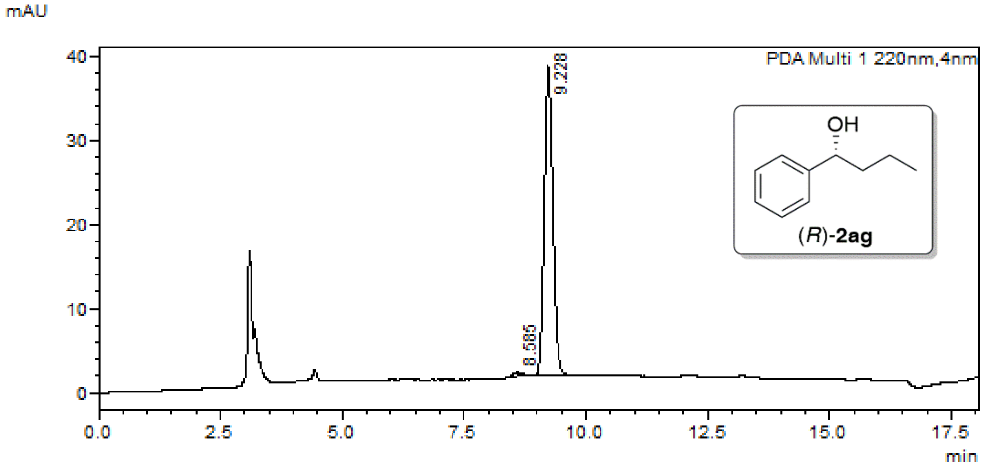

Peak Table

PDA Ch1 220nm

Peak#	Ret. Time	Area	Height	Height%	Area%
1	8.585	5945	506	1.361	1.298
2	9.228	452080	36700	98.639	98.702
Total		458025	37207	100.000	100.000

Supplementary Figure 213. HPLC of (*S*)-2ag on Chiralcel OJ-H at 30 °C

Conditions: *n*-hexane-2-PrOH (97:3, v/v); f=1.0 mL/min; λ=220 nm; *p*=4.3 MPa

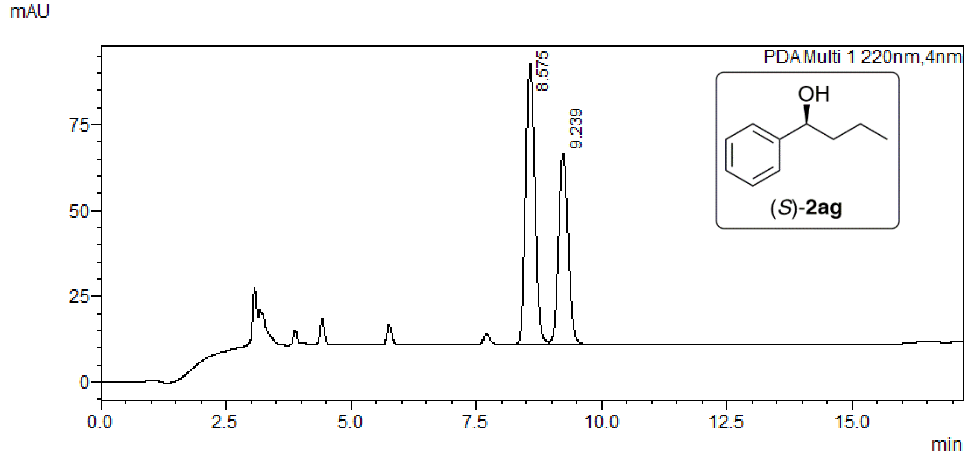

Peak Table

PDA Ch1 220nm

Peak#	Ret. Time	Area	Height	Height%	Area%
1	8.575	1027391	81424	59.414	58.599
2	9.239	725864	55622	40.586	41.401
Total		1753255	137045	100.000	100.000

Supplementary Figure 214. HPLC of *rac*-2ah on Chiralcel OD-H at 30 °C

Conditions: *n*-hexane-2-PrOH (98:2, v/v); f=1.0 mL/min; λ=220 nm; *p*=4.4 MPa

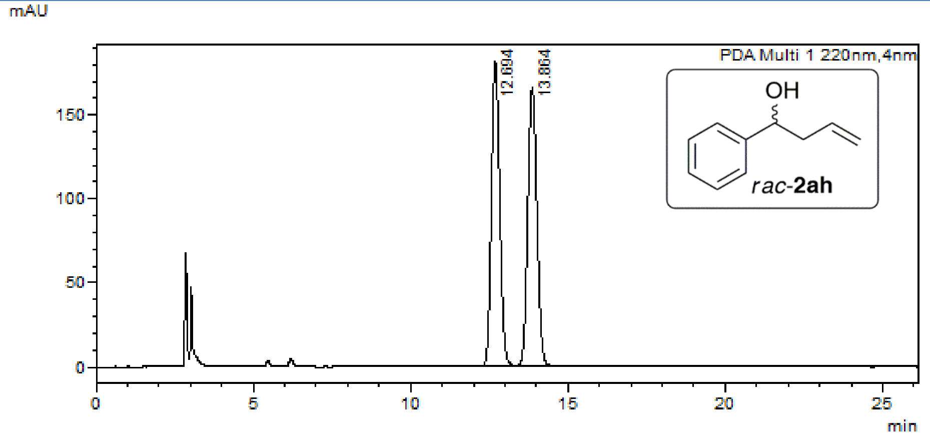

Peak Table

PDA Ch1 220nm

Peak#	Ret. Time	Area	Height	Height%	Area%
1	12.694	3358462	181017	52.069	49.992
2	13.864	3359502	166629	47.931	50.008
Total		6717963	347646	100.000	100.000

Supplementary Figure 215. HPLC of (*R*)-2ah on Chiralcel OD-H at 30 °C

Conditions: *n*-hexane-2-PrOH (98:2, v/v); f=1.0 mL/min; λ=220 nm; *p*=4.4 MPa

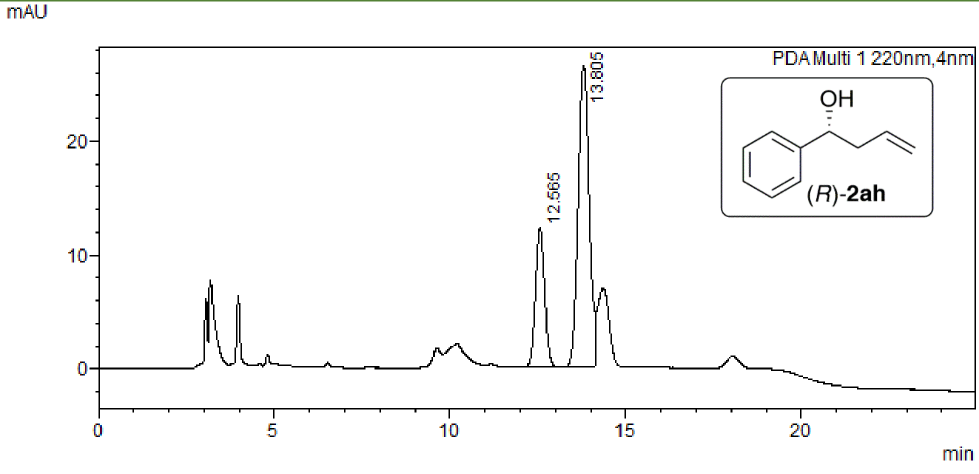

Peak Table

PDA Ch1 220nm

Peak#	Ret. Time	Area	Height	Height%	Area%
1	12.565	224071	12269	31.686	28.018
2	13.805	575673	26451	68.314	71.982
Total		799744	38720	100.000	100.000
