# Supplementary material for: Biocatalytic characterization of an alcohol dehydrogenase variant deduced from Lactobacillus kefir in asymmetric hydrogen transfer
Source: Commun Chem. 2023 Oct 12;6:217. doi: 10.1038/s42004-023-01013-1 (PMC10570314; doi:10.1038/s42004-023-01013-1)
Supplement: Supplementary file 7 — Reporting Summary [file 42004_2023_1013_MOESM7_ESM.pdf]

nature portfolio

Corresponding author(s): Paweł Borowiecki

Last updated by author(s): Sep 12, 2023

Reporting Summary

Nature Portfolio wishes to improve the reproducibility of the work that we publish. This form provides structure for consistency and transparency in reporting. For further information on Nature Portfolio policies, see our Editorial Policies and the Editorial Policy Checklist.

Statistics

For all statistical analyses, confirm that the following items are present in the figure legend, table legend, main text, or Methods section.

n/a	Confirmed	
☒	☐	The exact sample size (*n*) for each experimental group/condition, given as a discrete number and unit of measurement
☒	☐	A statement on whether measurements were taken from distinct samples or whether the same sample was measured repeatedly
☒	☐	The statistical test(s) used AND whether they are one- or two-sided<br>*Only common tests should be described solely by name; describe more complex techniques in the Methods section.*
☒	☐	A description of all covariates tested
☒	☐	A description of any assumptions or corrections, such as tests of normality and adjustment for multiple comparisons
☒	☐	A full description of the statistical parameters including central tendency (e.g. means) or other basic estimates (e.g. regression coefficient) AND variation (e.g. standard deviation) or associated estimates of uncertainty (e.g. confidence intervals)
☒	☐	For null hypothesis testing, the test statistic (e.g. *F*, *t*, *r*) with confidence intervals, effect sizes, degrees of freedom and *P* value noted<br>*Give P values as exact values whenever suitable.*
☒	☐	For Bayesian analysis, information on the choice of priors and Markov chain Monte Carlo settings
☒	☐	For hierarchical and complex designs, identification of the appropriate level for tests and full reporting of outcomes
☒	☐	Estimates of effect sizes (e.g. Cohen's *d*, Pearson's *r*), indicating how they were calculated

Our web collection on statistics for biologists contains articles on many of the points above.

Software and code

Policy information about availability of computer code

Data collection	NMR data were collected on a Spectrometr Varian NMR System 500 MHz (Varian, Inc., Palo Alto, CA, USA); HPLC analyses were performed on Shimadzu Nexera-i (LC-2040C 3D); Fourier Transform Mass Spectrometry (FTMS) spectra were recorded on Q Exactive Hybrid Quadrupole-Orbitrap Mass Spectrometer; Fourier transform infrared (FTIR) spectra of neat samples were recorded on an IRSpiritTM Fourier Transform Infrared Spectrophotometer Instrument with a DLATGS detector from Shimadzu Corp. equipped with the QATRTM-S single-reflection attenuated total reflectance (ATR) measurement attachment with a monolithic diamond crystal stage (high-throughput) as prism material and a pressure clamp; Tunnel statistics calculated with CAVER Analyst 2.0 software. Docking calculations were performed using AutoDock Vina vs. 1.1.2 program. Optimization of the lignad structure for docking calculations was performed using Avogadro vs. 1.2.0. Ligands and proteins for docking were initially prepared using UCSF Chimera vs. 1.11.2
Data analysis	Raw spectroscopic (NMR) data (FID files) have been processed with MestReNova (Product version: 6.0.2-5475); The PyMOL Molecular Graphics System software, vs. 1.3, Schrödinger, LLC (http://www.pymol.org/) was used to generate the figures for the enzyme crystal structures and the docked ligands; The program BIOVIA Discovery Studio Visualizer 20.1.0.19295 (Dassault Systèmes Biovia Corp.; https://www.3ds.com) was used to calculate/generate 2D protein-ligand interactions map. Analysis of HPLC crude data was performed using Shimadzu's LabSolutions CS software.

For manuscripts utilizing custom algorithms or software that are central to the research but not yet described in published literature, software must be made available to editors and reviewers. We strongly encourage code deposition in a community repository (e.g. GitHub). See the Nature Portfolio guidelines for submitting code & software for further information.

Data

Policy information about availability of data

All manuscripts must include a data availability statement. This statement should provide the following information, where applicable:

- Accession codes, unique identifiers, or web links for publicly available datasets
- A description of any restrictions on data availability
- For clinical datasets or third party data, please ensure that the statement adheres to our policy

All data collected within the published article are deposited in the main manuscript and/or Supplementary Data files. The data that support the findings of this study are available from the corresponding author on reasonable request.

Research involving human participants, their data, or biological material

Policy information about studies with human participants or human data. See also policy information about sex, gender (identity/presentation), and sexual orientation and race, ethnicity and racism.

Reporting on sex and gender	N/A - this study did not involve human research participants.
Reporting on race, ethnicity, or other socially relevant groupings	N/A
Population characteristics	N/A
Recruitment	N/A
Ethics oversight	N/A

Note that full information on the approval of the study protocol must also be provided in the manuscript.

Field-specific reporting

Please select the one below that is the best fit for your research. If you are not sure, read the appropriate sections before making your selection.

☒ Life sciences ☐ Behavioural & social sciences ☐ Ecological, evolutionary & environmental sciences

For a reference copy of the document with all sections, see nature.com/documents/nr-reporting-summary-flat.pdf

Life sciences study design

All studies must disclose on these points even when the disclosure is negative.

Sample size	N/A
Data exclusions	No data were excluded from our study.
Replication	All experiments were performed in triplicate and all the attempts were conducted with successful reproducibility.
Randomization	We did not used any randomization protocols during our studies since we followed standard synthetic organic methodology.
Blinding	All the experiments were performed using blind trials.

Reporting for specific materials, systems and methods

We require information from authors about some types of materials, experimental systems and methods used in many studies. Here, indicate whether each material, system or method listed is relevant to your study. If you are not sure if a list item applies to your research, read the appropriate section before selecting a response.

Materials & experimental systems

n/a	Involved in the study
☒	☐ Antibodies
☒	☐ Eukaryotic cell lines
☒	☐ Palaeontology and archaeology
☒	☐ Animals and other organisms
☒	☐ Clinical data
☒	☐ Dual use research of concern
☒	☐ Plants

Methods

n/a	Involved in the study
☒	☐ ChIP-seq
☒	☐ Flow cytometry
☒	☐ MRI-based neuroimaging
